# Dynamic evolution and biogenesis of small RNAs during sex reversal

**DOI:** 10.1038/srep09999

**Published:** 2015-05-06

**Authors:** Jie Liu, Majing Luo, Yue Sheng, Qiang Hong, Hanhua Cheng, Rongjia Zhou

**Affiliations:** 1 Department of Genetics, College of Life Sciences, Wuhan University, Wuhan 430072, P. R. China

## Abstract

Understanding origin, evolution and functions of small RNA (sRNA) genes has been a great challenge in the past decade. Molecular mechanisms underlying sexual reversal in vertebrates, particularly sRNAs involved in this process, are largely unknown. By deep-sequencing of small RNA transcriptomes in combination with genomic analysis, we identified a large amount of piRNAs and miRNAs including over 1,000 novel miRNAs, which were differentially expressed during gonad reversal from ovary to testis via ovotesis. Biogenesis and expressions of miRNAs were dynamically changed during the reversal. Notably, phylogenetic analysis revealed dynamic expansions of miRNAs in vertebrates and an evolutionary trajectory of conserved miR-17-92 cluster in the *Eukarya*. We showed that the miR-17-92 cluster in vertebrates was generated through multiple duplications from ancestor miR-92 in invertebrates *Tetranychus urticae* and *Daphnia pulex* from the *Chelicerata* around 580 Mya. Moreover, we identified the sexual regulator *Dmrt1* as a direct target of the members miR-19a and -19b in the cluster. These data suggested dynamic biogenesis and expressions of small RNAs during sex reversal and revealed multiple expansions and evolutionary trajectory of miRNAs from invertebrates to vertebrates, which implicate small RNAs in sexual reversal and provide new insight into evolutionary and molecular mechanisms underlying sexual reversal.

Over 80% of the human genome participates in biological activities and only 2% of the genome is protein-coding sequences^1^. Thus most part of the genome is non-coded. Non-coding RNAs (ncRNAs) play substantial roles in various biological processes. The ncRNAs include long non-coding RNA (lncRNA) relatively large than >200 nucleotides (nt) and small non-coding RNA of 20-35 nt (sRNAs), such as tRNA, rRNA, siRNA, small nuclear RNA, miRNA and piRNA (PIWI-interacting RNA). Since discovery of miRNA^2^, sRNAs have been identified to accomplish a variety of biological functions especially in gene regulations. The regulatory sRNAs, miRNAs and piRNAs, differ in biogenesis, size and the interacting Argonaute-proteins^3^,^4^,^5^,^6^.

The biogenesis of miRNAs with approximately 22 nucleotides begins with the transcription of pri-miRNAs from the miRNA genes, which are located in either coding or noncoding genes^7^. Most miRNA genes are transcribed into pri-miRNAs by RNA polymerase III, while a fraction of miRNA genes are transcribed by RNA polymerase II^8^. The pri-miRNA is cleaved by Drosha and DGCR8 to generate a ~70 nt miRNA precursor (pre-miRNA) with an imperfect stem-loop^9^. Pre-miRNA transportation from the nucleus to the cytoplasm is mediated by Exportin5^10^. The pre-miRNA is further processed by the ribonuclease III (RNase III) enzyme Dicer to yield ~22 nt miRNA duplex^11^,^12^. After being unwound from the duplex, the mature miRNA (guide strand) and the star miRNA (passenger strand) are released to act as regulatory molecules^13^,^14^. In addition, miRNAs frequently exhibited multiple mature variants with different lengths and are termed isomiRs^15^. The variants are mainly generated through movement of RNase-III domains of Drosha and Dicer around the cleavage site or additions of nucleotides at the 3’-terminal^16^,^17^. Mature miRNAs bound to Argonaute (Ago) proteins are assembled within the RNA-induced silencing complex (RISC), and inhibit translation or promote degradation of mRNA targets^18^,^19^,^20^. In animals, the miRNAs bind to the 3’ untranslated region or coding sequence of the target mRNAs with imperfect complementary base pairing to trigger the suppression^21^,^22^,^23^.

The piRNAs are another abundant class of small ncRNAs, which are associated with PIWI proteins in germline^24^,^25^,^26^,^27^,^28^. Mature piRNAs are approximately 24-32 nt long and their sequences are diverse among species. In primary piRNA biogenesis, long single-stranded precursor is transcribed mainly from intergenic regions and processed via a Dicer-independent manner^25^,^29^. piRNAs can be processed through a secondary “ping-pong” model involved in Miwi2/Mili in mouse or Ago3/Aub in fly^30^,^31^. piRNAs have been shown to play important roles in genome defense and germ cell development. Mutations in Piwi and Mili genes in mice resulted in spermatogenesis defects^32^,^33^. In zebrafish, loss of germ cells has been detected in mutations of Ziwi or Zili^29^,^34^. Consistent with functions of piRNA-associated genes, a W-chromosome-derived, female-specific piRNA has been identified as a feminizing factor in silkworm (*Bombyx mori*), and plays an important role in primary sex determination in the WZ sex determination system^35^.

In addition to the germline piRNAs, a considerable amount of miRNAs has been identified in gonads in animals^36^,^37^,^38^,^39^,^40^. Mutations in the miRNA pathway genes frequently resulted in germline defects in *C. elegans* and mice^41^,^42^. Some miRNAs are associated with sexual development. For instance, miR-378 was spatiotemporally expressed in porcine granulosa cells and down-regulated aromatase expression, which converted androgens to estrogens and was essential for ovary development^43^. miR-124 can repress both translation and transcription of sexual regulator *Sox9* in ovarian cells^44^. Overexpression of miR-181 inhibited *Dax-1*, a negative regulator of androgen receptor and promoted the progression of prostate tumor growth in nude mice^45^. Loss of miR-184 resulted in complete loss of oogenesis in *Drosophila*^46^. Recent studies showed that a well-conserved miRNA let-7 functioned as a modulator of a somatic systemic signal in *Drosophila* gonads and four miRNAs participated in regulating post-mating responses in females^47^,^48^. In fish, miR-430 was a direct posttranscriptional regulator of sexual related genes *nanos*, *tdrd7*, *hub* and *sdf-1* which are necessary for the proper migration, maintenance and survival of primordial germ cells^49^,^50^,^51^,^52^. These studies highlight the importance of understanding the functions of miRNAs in germline development.

Origin and evolution of miRNAs remain open questions. Particularly, many novel miRNAs have been identified in vertebrates through deep sequencing^53^, which stimulated further exploration of miRNA evolution. New miRNAs emerged and further got stable in genome possibly through a mechanism of the birth-death^54^,^55^. Net gain of miRNAs was estimated as 0.3-1 new gene per Myr^56^,^57^. Several studies showed that the evolution of miRNAs and their regulatory networks were adaptive to various developmental processes, such as reproduction and metamorphosis in *Drosophila*, and the cardiac and muscle development in vertebrates^40^,^58^,^59^,^60^,^61^. In addition, the X-linked miRNAs evolved rapidly in mammals, implying possible roles in male reproduction^62^,^63^. Although extensive studies above, miRNA evolution and functions in sexual development remain largely unknown.

Sexual dimorphism in fish is often associated with differential growth between male and female, thus production of monosex populations is desirable in aquaculture. Some fish species undergo naturally sex changes via an intersex stage during their life cycles, such as a juvenile ovary to testis transition in zebrafish gonadal development^64^. Swamp eel (*Monopterus albus*), which belongs to the family Synbranchidae, undergoes naturally sex reversal from female to male via intersex, a process accompanied by extensive morphological and physiological change of the gonad^65^,^66^. Gonadal transformation is an ideal model system for sexual development in vertebrates.

Here we take advantage of sex reversal characteristic in the teleost fishes and use deep-sequencing techniques to analyze small RNA transcriptomes during gonad transformation from ovary to testis via ovotesis. We identified a large amount of piRNAs and miRNAs including over 1,000 novel miRNAs, which were differentially expressed during gonad reversal. We showed dynamic changes of miRNA biogenesis and expression in three types of gonads. Particularly, we revealed dynamic expansions of miRNAs in vertebrates and presented an evolutionary trajectory of conserved miR-17-92 cluster. Moreover, we identified the sexual regulator *Dmrt1* as a direct target of the members miR-19a and -19b in the cluster. These results provide new insight into the evolution and functions of miRNAs in sexual differentiation.

## Results

### Small RNA profiling during gonad reversal

**Figure 1:**
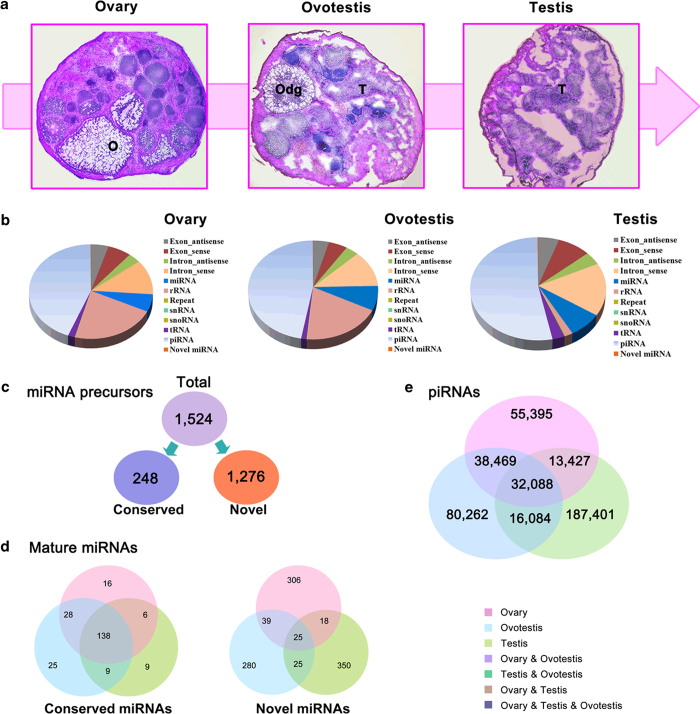
**Gonad reversal and small RNA profiling in swamp eel.** (**a**) Sex reversal from female into male via intersex revealed by H & E staining of ovary, ovotestis and testis (magnification x10). T, testis; O, ovary; Odg, degenerated ovum. (**b**) Read percentages for small RNA sequences in ovary, ovotestis and testis respectively. By aligning to reference genome, Rfam database, GenBank noncoding database and repeat annotation files, all mapped reads are classified into 12 categories as intron, exon, rRNA, snoRNA, tRNA, snRNA, miRNA, repeat sequences, piRNA and unannoted groups (including novel miRNAs). (**c**) Overview of the miRNA precursors including conserved and novel miRNAs. (**d**) Venn charts indicate expression patterns of conserved miRNAs and novel miRNAs among three gonads. Conserved miRNAs are mainly co-expressed in three types of gonads, while most novel miRNAs are specifically in one type of gonads. (**e**) Graphic representation of the overlapping and non-overlapping piRNAs among three stages of the gonads. In total, 139,379, 166,903 and 249,000 piRNAs were detected in ovary, ovotestis and testis, respectively.

To investigate roles of small RNAs during gonad reversal from ovary, ovotestis to testis, we first analyzed small RNA transcriptomes from these gonad tissues, obtained a total of 47,551,844 clean reads (11,882,708 in ovary, 13,321,543 in ovotestis and 22,347,593 in testis) using high-throughput sequencing by the Illumina HiSeq 2000 platform (Fig. 1a,b). After mapping to the reference genome, Rfam database, GenBank noncoding databases and repeat annotation files, the small RNAs were annotated and a full list of small RNAs involved in gonad reversal was obtained including tRNAs, snRNAs, miRNAs and piRNAs (Fig. 1b; Table S1). We further identified miRNAs and piRNAs in the three types of gonads. In total, 1,524 pre-miRNAs encoding 1,274 mature miRNAs including 231 conserved and 1043 novel miRNAs were obtained (Fig. 1c,d; Table S2, S3); and 139,379, 166,903 and 249,000 unique piRNA sequences were detected in ovary, ovotestis and testis (Fig. 1e), respectively, indicating that both miRNAs and piRNAs are enriched in germline during gonad reversal.

### piRNA pathways are associated with gonad reversal

**Figure 2:**
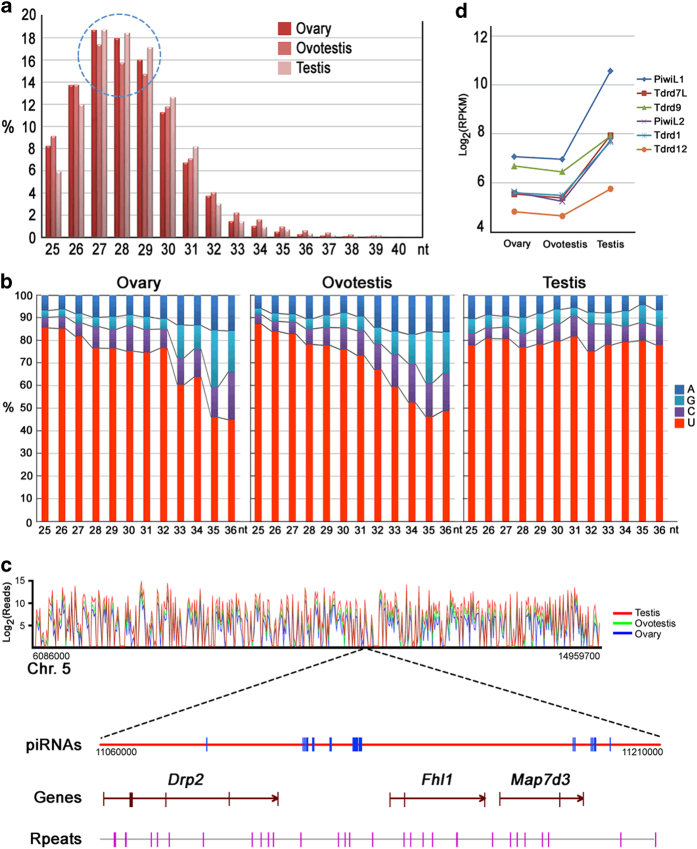
**Global view of piRNAs during gonad reversal.** (**a**) The size distributions (in nucleotides) of piRNAs cloned from the ovary, ovotestis and testis. The doted cycle indicates that the numbers of the piRNAs that range from 27 to 29 nucleotides are markedly reduced in ovotestis. (**b**) The preference distribution for uridine at the 5’ end position of different lengths (nt) of piRNAs in the ovary, ovotestis and testis. The piRNAs in the ovotestis and ovary show a remarkable loss in preference for uridine at the 5’ end position, particularly in the longer piRNAs. (**c**) An example of piRNAs mapped on the chromosome 5 of the swamp eel. The relative expressions of the piRNAs in three types of gonads are shown in different colors (ovary, blue; ovotestis, green; testis, red). A 150 kb window showed that piRNAs are mainly located in intergenic loci. piRNAs are indicated in blue, the exons of the genes are indicated in brown. The repeat loci in purple are shown at the bottom of the chart. (**d**) Differential expression of the piRNA-associated genes of the *Piwi* (*PiwiL1* and *PiwiL2*) and *Tdrd* (*Tdrd1*, *Tdrd7L, Tdrd9* and *Tdrd12*) families in the ovary, ovotestis and testis, which are upregulated during the transition from ovotestis to testis.

Germline piRNAs play an important role in genome defense and germ cell development. However, their mechanisms of action, biogenesis and functions in sex reversal remain unknown. Deep sequencing of the piRNA pools in the three stages of the gonads revealed a broad range of differential expression during gonad reversal (Fig. 1e). Despite the lack of gametogenesis in the ovotestis, a considerable abundance of piRNAs was observed. Meanwhile, the proportion of piRNAs with lengths ranging from 27-29 nucleotides was markedly reduced in the ovotestis (Fig. 2a). In addition, the piRNAs in the ovotestis and ovary showed a remarkable loss in preference for uridine at the most 5’ end position, particularly in the longer molecules (Fig. 2b). These data indicate that a certain amount of piRNAs in ovotestis may be important for following testis transition, and a strong 5’ U bias for piRNAs may be more pivotal for testicular than ovary development.

High-seq analysis indicated higher levels of piRNA expression in testis than in ovary and ovotestis. In addition, approximately 80% of the piRNAs were mapped to the intergenic regions. The 150 kb window showed the example of intergenic piRNAs between genes Drp2 and Fhl1 and piRNAs on the intron of Drp2 and Map7d3 (Fig. 2c). Consistent with the piRNA patterns, the genes encoding proteins of piRNA pathways, such as Piwis and Tdrds, were up-regulated during the progression from ovotestis to testis (Fig. 2d), suggesting that piRNA pathways are associated with gonad reversal.

### Dynamic changes of miRNA biogenesis and expression during gonad reversal

**Figure 3:**
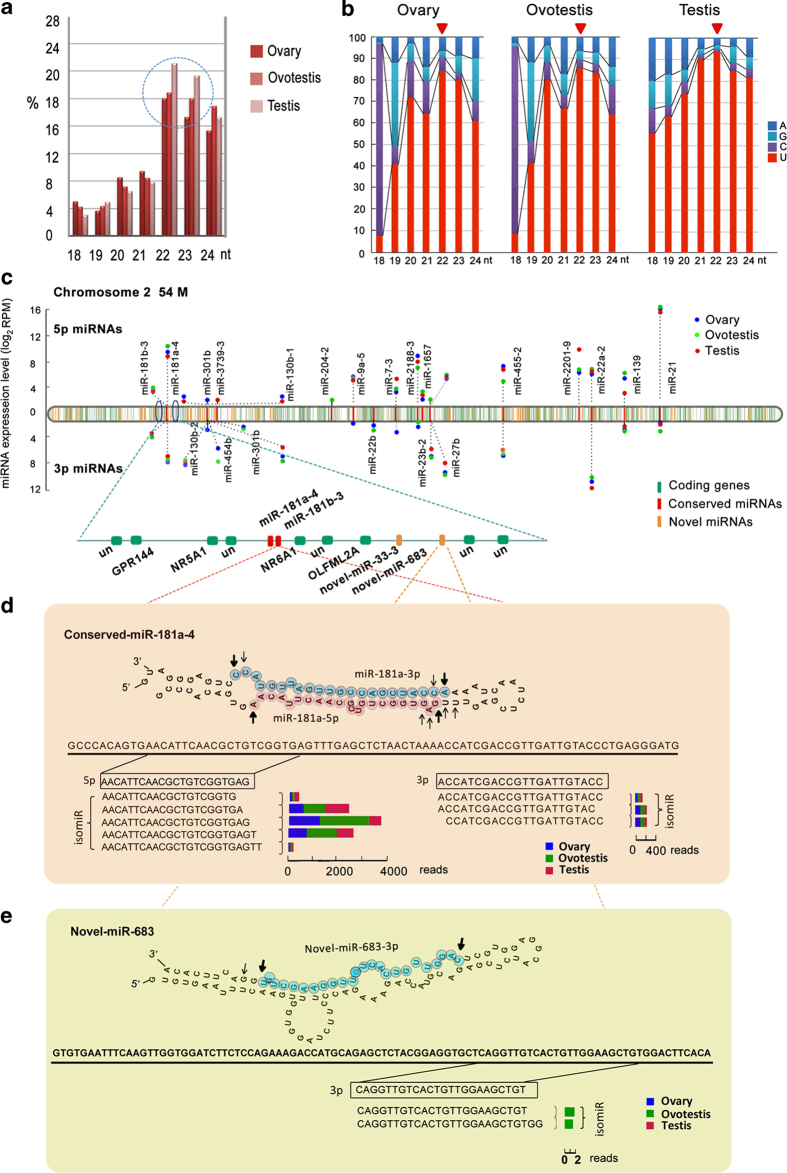
**Structure features of miRNAs during gonad reversal.** (**a**) Length distributions of the miRNAs cloned from ovary, ovotestis and testis respectively, which exhibit characteristic sizes in 22-23 nt. The doted cycle indicates that the numbers of the miRNAs that range from 22 to 23 nucleotides are markedly raised in testis. (**b**) The preference distribution for uridine at the 5’ end position of different lengths (nt) of miRNAs in the ovary, ovotestis and testis. The miRNAs in the ovary and ovotestis show a remarkable loss in preference for uridine at the 5’ end position. (**c**) The chromosome 2 harbors 25 conserved miRNAs (red), 126 novel miRNAs (orange), in addition to 1754 protein-coding genes (green). Most of the miRNA precursors are located in intergenic regions. Over half of the stem loops can generate both 5’ and 3’ miRNAs from both arms. The conserved miRNAs are enriched in ovotestis. RPM, Reads Per Million reads. (**d**) The conserved miRNAs are mainly derived from both arms of a precursor (both 5’ and 3’). miR-181a-4 as an example for conserved miRNA biogenesis shows that differential processes at 5’ or 3’ end generate various isomiRs, which are differentially present during gonad reversal. (**e**) Novel miRNAs are mainly derived from one (either 5’ or 3’) arm of a precursor. Novel-miR-683 is used as an example for novel miRNA biogenesis. Multiple isomiRs are processed, most of which are present in one type of gonads. The length of rectangles indicates the reads of the isomiR in different types of gonads (ovary, blue; ovotestis, green; testis, red). The dominant cleavage sites are shown by arrows. Bold arrows show the most abundant isomiR cleavage sites, whereas thin arrows indicate less abundant isomiR cleavage sites.

To examine potential roles of miRNAs in gonad differentiation, we first analyzed miRNA size and U-bias at first nucleotide. During gonad reversal, the proportion of miRNAs with length ranging from 22-23 nucleotides was markedly raised from ovary, ovotestis to testis (Fig. 3a). Preference for uridine at the most 5’ end of miRNAs showed an increasing tendency from 18 to 22 nt during gonad reversal; the U-bias was remarkable at first nucleotide of miRNAs with length of the 22 nt in testis (Fig. 3b). These data suggested that miRNAs with length of 22 nt are important for the gonad reversal.

Most of the miRNAs (65% of conserved miRNAs; 78% of the novel miRNAs) are located in intergenic regions. For instance, the chromosome 2 harbors 25 conserved and 126 novel miRNA precursors, all of which are intergenic miRNAs, in addition to 1,754 protein-coding genes (Fig. 3c). Over half of the stem loops (127, 51.21%) generated 72.73% (168) mature miRNAs from both arms, indicating various biogenesis during gonad reversal.

We observed diversity of miRNA biogenesis during gonad reversal. Most miRNAs from one arm of the pre-miRNA were generated at much lower frequencies (star strand) than the miRNAs from the opposite arm (guide strand). However, we also detected a few miRNAs generated in an equal frequency at 5’ or 3’ arm (Fig. 3c,d). In addition, the conserved miRNAs are mainly derived from both arms of a precursor (both 5’ and 3’), while novel miRNAs are mainly derived from one (either 5’ or 3’) arm of a precursor (Fig. 3d,e). Furthermore, miRNA diversity during gonad reversal was revealed by various isomiRs. More isomiRs were detected in conserved miRNA group than those in novel miRNAs. On average, 11.8, 13.8 and 12.6 isomiRs per conserved miRNA was detected in ovary, ovotestis and testis respectively, however the numbers (4.3, 3.2 and 7.3) were much lower for the novel miRNAs. In addition, for conserved miRNAs, multiple isomiRs were mainly detected in all three types of gonads, while for novel miRNAs, the isomiRs were present dominantly in one type of gonads (Fig. 3d,e). These results indicated that differential processes at 5’ or 3’ end generated various isomiRs, which were also differentially present during gonad reversal.

**Figure 4:**
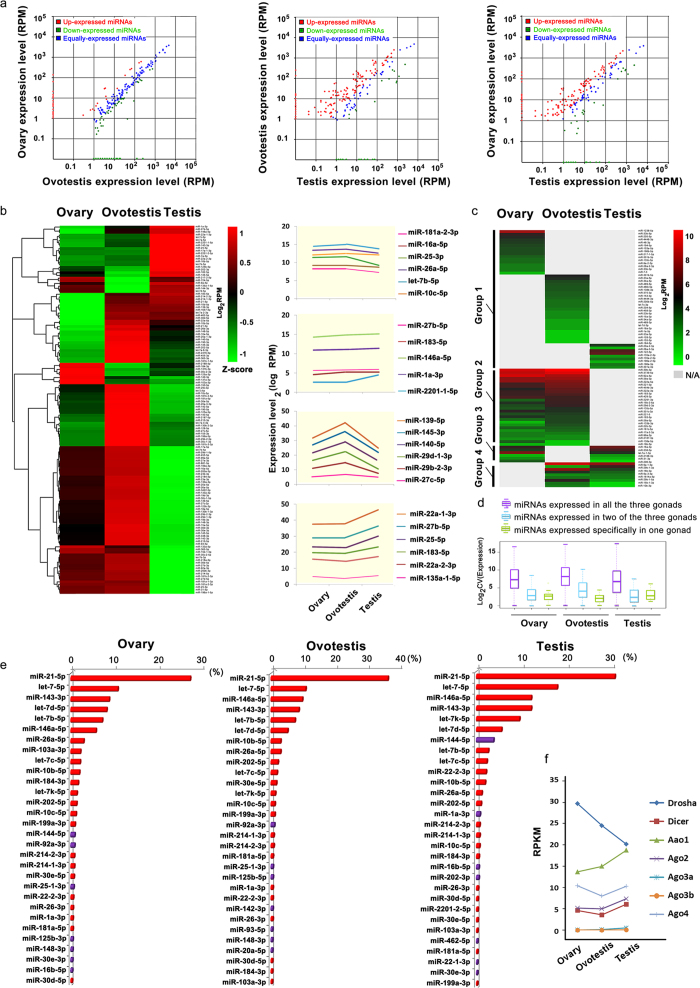
**Expression profiling of conserved miRNAs during gonad reversal.** (**a**) Scatter plot charts indicate the differential expressions of miRNAs in three types of gonads respectively. Red and green dots represent miRNAs up-regulated and down-regulated with a fold change >2 respectively, while the blue dots represent equally expressed miRNAs between two types of gonads. RPM, reads per million reads. (**b**) Hierarchical clustering of the conserved miRNAs and their expressions among ovary, ovotestis and testis. Colors indicate relative expressions compared to the mean value. For each miRNA, red and green colors indicate high and low frequency, respectively. A substantial fraction (66.42%) of the conserved miRNAs are highly expressed in ovotestis. 28 miRNAs (20.44%) are highly expressed in testis, and 14 miRNAs (10.22%) are enriched in ovary. The examples of the enriched miRNAs at different stages of gonads during sex reversal are indicated on the right column. (**c**) The heatmap shows that a fraction of the conserved miRNAs is specifically expressed in one or two types of gonads. (**d**) Comparisons of miRNA expression levels among different gonads show that the miRNAs expressed in three types of gonads exhibit higher levels than in one or two types of gonads. (**e**) Top thirty of the conserved miRNAs with the highest abundance in gonads. The most of these miRNAs (23, red) are enriched in all three gonads. The y axis, miRNA names; the x axis, % reads in each gonad. (**f**) Differential expression of the miRNA pathway genes (*Drosha, Dicer* and *Ago* members: *Ago1, 2, 3a, 3b* and *4*) in the ovary, ovotestis and testis.

**Figure 5:**
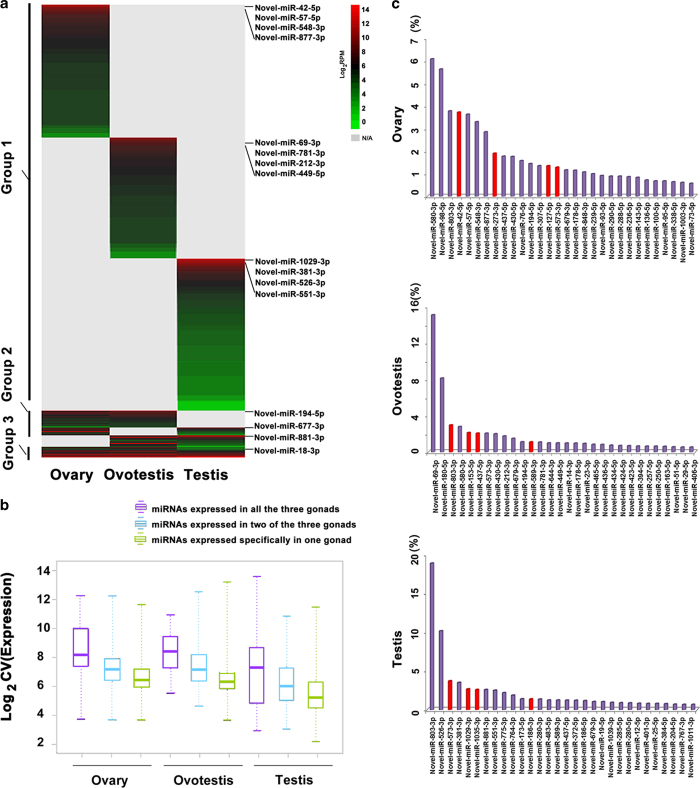
**Expression profiling of novel miRNAs during gonad reversal**. (**a**) The heatmap shows that the novel miRNAs (1043) are divided into three groups (group 1: specifically expressed in each gonad (89.7%); group 2: expressed in two types of gonads (7.9%); group 3: expressed in all three types of gonads (2.4%)). (**b**) Boxplot of novel miRNAs shows a substantial variation in expression levels in different gonads. (**c**) Top thirty of the novel miRNAs with the highest abundance in gonads. A few of these miRNAs (4, red) are enriched in all three gonads. The y axis, miRNA names; the x axis, % reads in each gonad.

We further investigated miRNA expression patterns in three types of gonads. In general, deep sequencing analysis showed that conserved miRNAs were mainly co-expressed in three types of gonads, while most novel miRNAs are specifically in one type of gonads (Figs. 4, 5). Particularly, from ovary to ovotestis transition, a small fraction of conserved miRNAs (16/135) were significantly up-regulated or down-regulated (36/135). However, during ovotestis to testis reversal, a large number of the conserved miRNAs (106/135) was significantly down-regulated, while a few of them (13) was up-regulated (Fig. 4a,b). For conserved miRNAs, there was a small fraction of specifically expressed-miRNAs in one type of gonads (16 in ovary, 25 in ovotestis and 9 in testis), and most of which were the star strands (miR*) instead of guide miRNAs (Fig. 4c). In contrast, most of the novel miRNAs were specifically expressed in one gonad (389 in ovary; 358 in ovotestis and 429 in testis); only 26 novel miRNAs were co-expressed in all three types of gonads (Fig. 5). Indeed, the miRNAs expressed in three types of gonads exhibit higher levels than in one or two types of gonads (Fig. 4d,e; Fig. 5b). In addition, consistent with the miRNA patterns, the miRNA pathway genes, such as *Dicer, Drosha, Ago1, Ago2* and *Ago4* were expressed at a high level in gonads (Fig. 4f). These results indicated that dynamic expressions of miRNAs, especially the upregulation in ovotestis, are important for gonad reversal from ovary to testis via ovotestis.

### Dynamic expansions and evolution of miRNAs in vertebrates

**Figure 6:**
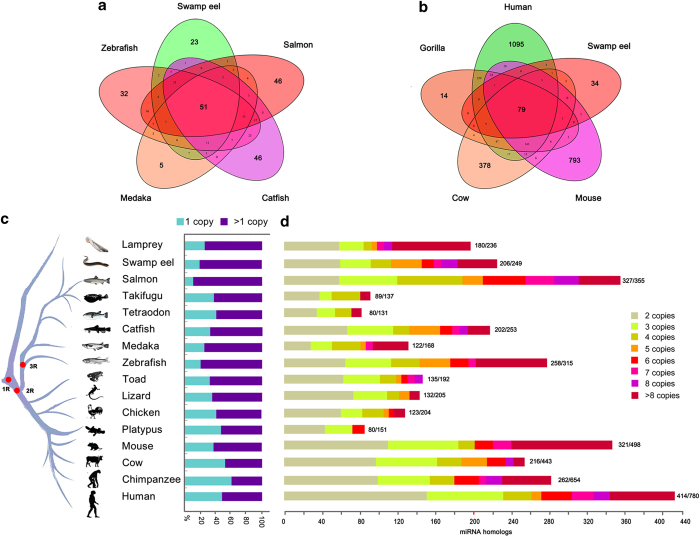
**Evolutionary conservation and duplications of miRNAs in vertebrates. (a**) Venn charts show that miRNAs are conserved among swamp eel, other teleosts (**a**) and mammals (**b**). Most of miRNAs among the teleosts and mammals are well-conserved (85.3%, swamp eel vs mammals), while the others are poorly-conserved. Data were from miRBase v21.0 and this study. (**c**) Relative percentages between single and multiple-copy miRNA precursors. Conserved miRNAs detected at least in two species were used in this analysis. The miR-430 has 78 copies, which result in a high proportion of multiple copies in lamprey. The bars show percentages of single or multiple-copy miRNA precursors. Red dots on the evolution tree indicate 1, 2, and 3 times of whole genome duplications during vertebrate speciation. (**d**) Numbers of multiple-copy miRNA precursors (n >1) in each species. miRNA homologs with a certain copy number include miR-n-1 , …-n-n; miR-na, -nb, …-nz; n = 1, 2, ..n. The numbers at the end of bars indicates multiple-copy numbers/total conserved miRNAs.

miRNA diversity would be associated with fine regulations of gene expressions. To explore the generation of various miRNAs, we analyzed expansion features of miRNA genes in vertebrates. Novel miRNAs were newly evolved, which are species specific. Considerable amounts of novel miRNAs were observed in vertebrates, for example, 1043 novel in swamp eel and more than 1,000 in human^53^ were detected. In addition, we further analyzed duplications of the conserved miRNAs. Evolutionary conservation analysis showed that a considerable fraction of the miRNAs was conserved among the teleosts and mammals (Fig. 6a,b). For example, 85.3% of the miRNAs in swamp eel is conserved in comparison with the mammalian miRNAs.

To analyze miRNA expansions, we categorized single- and multiple-copy miRNAs. Statistic analysis of the multiple-copy miRNAs showed that duplication events occurred frequently in most phylogenic branches, especially in three kinds of teleosts (92.1% in Salmon, 85.1% in swamp eel and 81.9% in zebrafish) (Fig. 6c), indicating a role of the third whole genome duplications in miRNA expansions in teleost lineages. In addition, multiple duplications of over 4 times were observed in some mammals (e.g. human, chimpanzee, mouse and cow), teleosts (e.g. zebrafish, medaka, salmon, catfish and swamp eel) and lamprey; in contrast, there were less multiple duplications in platypus, chicken, lizard, toad and pufferfish (Fig. 6d), indicating a significant contribution of local multiple duplications in a genomic region to the miRNA expansions.

**Figure 7:**
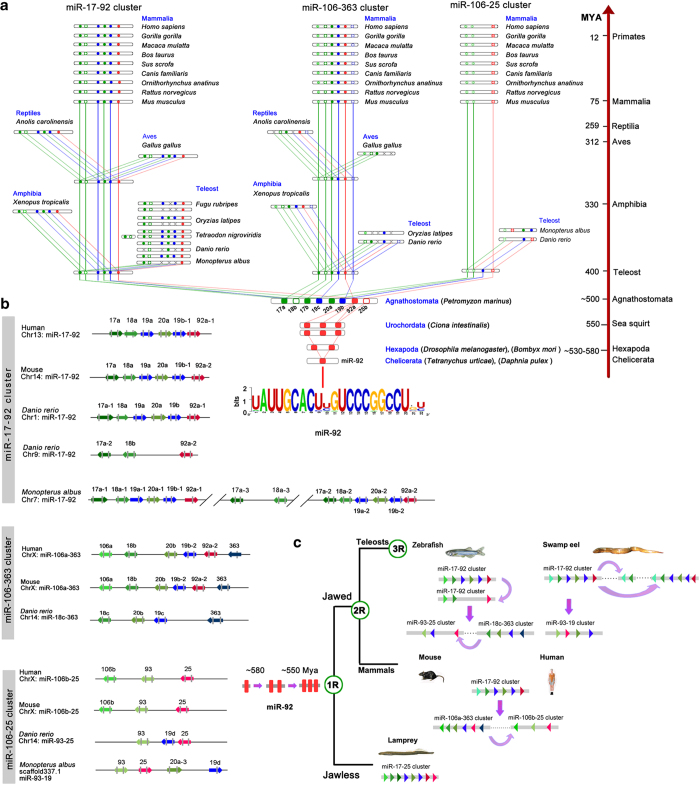
**Evolutionary trajectory of miR-17-92 cluster.** (**a**) Phylogenetic analysis of the miR-17-92 cluster. Original miRNA cluster in lamprey has eight members including miR-17a, −18b, 17b, −19c, −20a, −19b, −92a and −25b. Jawed vertebrates have miR-17-92, miR-106-363 and miR-93-25 clusters. Both *Tetranychus urticae* and *Daphnia pulex* in *Arthropoda* have single copy miR-92. Both fruitfly (*Drosophila melanogaster*) and silkworm (*Bombyx mori*) have two linked copies (miR-92a/b). In sea squirt, miR-92 was duplicated again to generate two clusters, each with three members (miR-92a/b/c/d/e-1/e-2) on different chromosomes. Consensus sequence uAUUGCACugUCCCgGcCU of the miR-92 is indicated at the bottom on the panel. Divergence times are indicated on the right (MYA, million years ago). Green: members of the miR-17 family; blue: members of the miR-19 family; Red: members of the miR-25 family. (**b**) The genomic arrangements of miR-17-92 cluster and its paralogues in vertebrates. (**c**) An evolution model for miR-17-92. Original miR-17-92 cluster, which originated from miR-92, formed two clusters after 2^nd^ whole genome duplication, one of which duplicated again within chromosome in mammals. In teleost fish, miR-17-92 was duplicated along with 3^rd^ whole genome duplication. Duplicated cluster members could be lost, however, intra-chromosome duplications frequently occurred to compensate the miRNA loss. During evolution, deletions and rearrangements of miRNAs within the cluster also occurred.

To further explore miRNA duplications in vertebrates, we focused on the evolution of miR-17-92 cluster, which appeared in the vertebrate lineage. Phylogenetic tree of the miR-17-92 precursors showed that 18 members in swamp eel were clustered into three families including miR-17, miR-19 and miR-92 (Suppl. Fig. 1). These miRNAs formed four clusters (3 miR-17-92 and 1 miR-93-19) in the genome of swamp eel (Fig. 7). To trace the original form of the cluster, we analyzed miR-17-92 clusters in both jawless and jawed vertebrates. In jawless lamprey there was only one original miR-17-92 cluster with eight members including miR-17a, −18b, 17b, −19c, −20a, −19b, −92a and −25b (Fig. 7a). In jawed vertebrates, the original miR-17-92 cluster formed two clusters (e.g. miR-17-92 and miRNA−106a-363 in human) along with the 2^nd^ whole genome duplication around 500 Mya. One of the two clusters duplicated again within the chromosome to form the third cluster miR-106b-25 in mammals. In the teleost fishes, another whole genome duplication event occurred about 300 Mya, thus, the miR-17-92 was further duplicated along with the 3^rd^ genome duplication to form four clusters, for example, two miR-17-92, one miR-18c-363 and one miR-93-25 in zebrafish. In swamp eel, there were three linked miR-17-92 clusters on the chromosome 7. The first miR-17-92 cluster spanned 602 bp, which comprised six miRNA genes and yielded 10 mature miRNAs in gonads. The second cluster spanned 741 bp with six miRNA genes and generated 12 mature miRNAs in gonads, while the third cluster contained miR-17a and miR-18a on the upstream 842 bp of the second cluster, generating four mature miRNAs in gonads. Another cluster miR-93-19 with 730 bp contained four miRNA genes, which generated 7 mature miRNAs in gonads (Fig. 7a,b). These results indicated that miR-17-92 cluster with eight members in the jawless lamprey were original form of the miR-17-92 in vertebrates; further inter-chromosomal and intra-chromosomal duplications generated multiple clusters in the jaw vertebrates.

To analyze origin of the miR-17-92 cluster before jawless vertebrates, we searched their homologs in the *Eukarya*. In early Bilateria animals, only miR-92 was detected and no other members were observed. miR-92 with single copy was first emerged in both *Tetranychus urticae* and *Daphnia pulex* in *Arthropoda* around 580 Mya. In *Hexapoda* (*Insecta*), miR-92 was duplicated once to form two linked copies (miR-92a/b) in fruitfly (*Drosophila melanogaster*) and silkworm (*Bombyx mori*). In *Urochordata*, miR-92 was duplicated again to generate two clusters, each with three members (miR-92a/c/d, miR-92b/e-1/e-2) on different chromosomes of sea squirt (Fig. 7a). These miR-92 genes were well-conserved and had a consensus sequence uAUUGCACugUCCCgGcCU. These results suggested that the original member of the miR-17-92 cluster was miR-92, which emerged first in the *Arthropoda* around 580 Mya. Based on these analyses, an evolution model for miR-17-92 was proposed (Fig. 7c).

### *Dmrt1* is a direct target of the miR-19a/b

**Figure 8:**
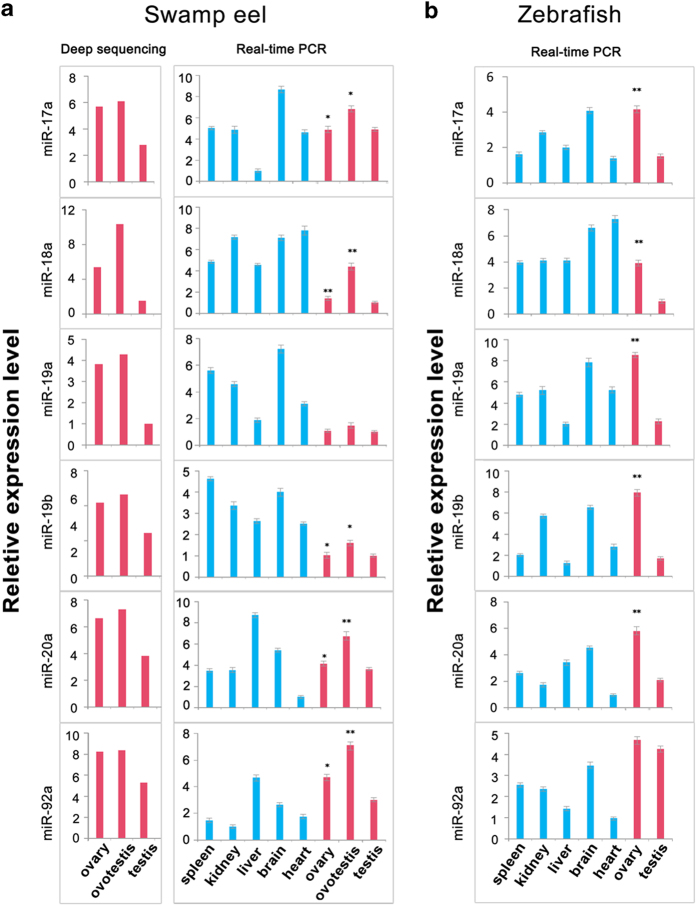
**Tissue distributions of mature miRNAs of miR-17-92 cluster in both zebrafish and swamp eel.** (**a**) Six mature miRNA guide strands are expressed in adult tissues of swamp eel. The expression levels in gonads were detected by q-PCR in comparison with deep sequencing (red bars). (**b**) Six mature miRNA guide strands are expressed in adult tissues of zebrafish. The expression was analyzed by q-PCR. The statistical significance was assessed by Student’s t-test with *p* value <0.05(*), *p* value <0.01(**). The *y* axis indicates the log2 fold change.

To illustrate functions of the miR-17-92 cluster in gonad differentiation, we investigated expression patterns of the miR-17-92 members in both zebrafish and swamp eel. Six guide strands of miR-17-92, also known as functional miRNA strands, were analyzed by qRT-PCR in adult tissues. These miRNAs could be detected in all the tissue types with various levels (Fig. 8a,b). During gonad reversal, these miRNAs were up-regulated in ovotestis compared with ovary and testis. All the expressions of the six members in ovary were higher than in testis in both zebrafish and swamp eel. This expression pattern was consistent with that in deep sequencing (Fig. 8a). In the meantime, the expression of six star miRNAs in miR-17-92 members can also be detected by qRT-PCR, which exhibited similar expression patterns with the guide miRNAs (Suppl. Fig. 2).

**Figure 9:**
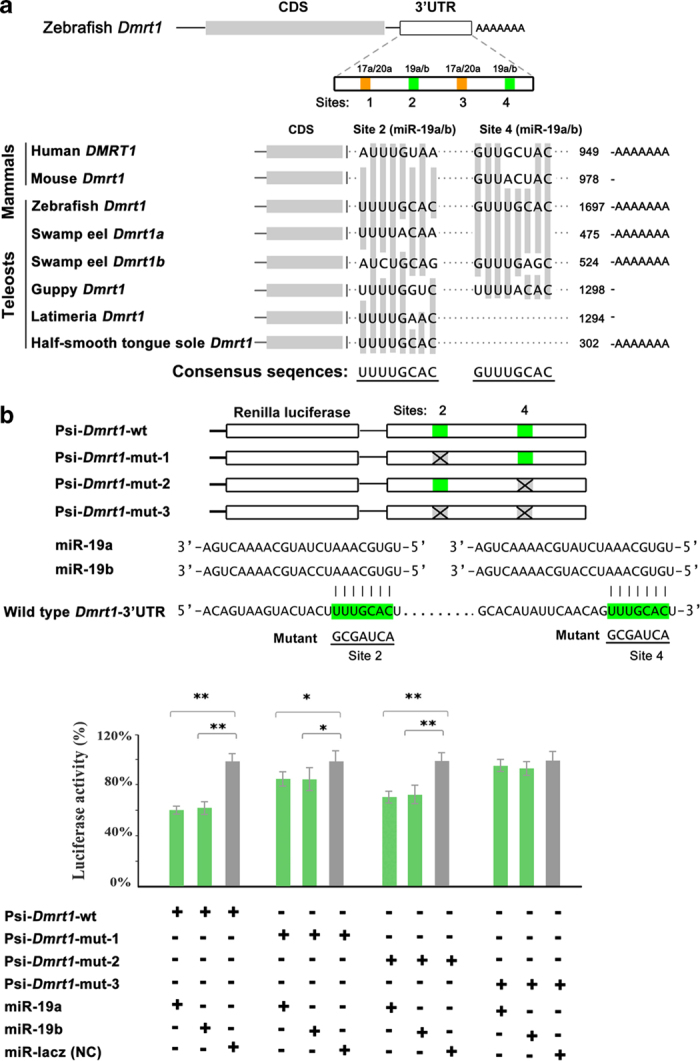
**Target identification of miR-19a/b.** (**a**) Schematic chart shows four candidate miRNA binding sites in *Dmrt1* 3’-UTR for miR-17a/20a and miR-19a/b in zebrafish. Sequence alignment of two miR-19a/b target sites in *Dmrt1* 3’UTR in both mammals and teleosts shows that the site 2 is conserved in the teleosts. (**b**) Luciferase reporter assay for validating the interaction of the miR-19a/b with the 3’-UTR of *Dmrt1*. Luciferase reporter was linked upstream of the *Dmrt1* 3’-UTR. Mutant sites of the miR-19a/b in the 3’-UTR are indicated in the symbol “X”. Luciferase activity due to the miR-19a/b binding to the 3’-UTR of wild type *Dmrt1* is significantly inhibited. The site 2 contributing to the inhibition effect is more efficient than the site 4. The activity is not affected when both two sites are mutated. The luciferase activity of Renilla luciferase was normalized by the firefly luciferase activity. The statistical significance was assessed by Student’s t-test with *p* value <0.05(*) and *p* value <0.01(**).

To analyze potential target genes of the miR-17-92 members in gonad differentiation, we searched miRNA targets in the zebrafish 3’UTR using the TargetScan (targetscan.org/fish_62/) and miRecords (miRecords.umn.edu/miRecords)^67^,^68^. In total, 1362 target genes were detected and categorized into 46 functional groups corresponding to three major categories (biological process, cellular component, and molecular function) by Gene ontology (GO) classification. Of which there were 14 known genes involved in sexual development with putative targets of miR-17-92 cluster (*Dmrt1, Wt1a, Amh, Cbx1a/b, Sox8, Sox9a/b, Bmp2, Lhx9, Wnt4a, Rspol, Foxl2* and *Sf-1*). Bioinformatic analysis displayed that *Dmrt1* was a target gene with four candidate miR-17-92 target sites in its 3’UTR. The site 1 and site 3 corresponded to the seeds of miR-17a/20a, while site 2 and site 4 to the seeds of miR-19a/b respectively. The target sites of miR-17a/20a, located at nt 251-283 and nt 1343-1375 of the 3’UTR, belong to the 7-mer-A1, which exactly matched to positions 2-7 seed region of the mature miRNA followed by an ‘A’. The target sites of miR-19a/b (nt 952-974 and 1452-1474) in the 3’UTR corresponded to 7-mer-m8 exactly matched to positions 2-8 of the mature miRNA (the seed + position 8) (Fig. 9a).

To confirm miR-17-92 regulations of *Dmrt1*, we generated a luciferase reporter fused with wild type *Dmrt1* 3’-UTR containing all four miR-17-92 sites. To express the mature miRNA guide strands miR-17a-5p, miR-20a-5p, miR-19a-3p and miR-19b-3p, we constructed miR-17a/20a/19a/19b-GFP plasmids by inserting a modified DNA fragments containing the precursors of the miRNAs into the pcDNA6.2-GW/EmGFP-miR vector, and the miRNA could co-express with GFP protein from a single mRNA. Luciferase assays showed that miR-19a/b significantly repressed activity of *Dmrt1* reporter, but the miR-17a/20a had no effective repression. To figure out the effective target between the two sites of miR-19a/b, we also generated *Dmrt1* mutant reporters with either one or both site mutations (mismatching to the seed region of miR-19a/b). The luciferase assays showed that either site 2 or site 4 mutant of *Dmrt1* 3’UTR significantly affected the luciferase activity, and the site 2 contributing to the inhibition effect was more efficient than the site 4, while double mutant of both sites abolished the inhibition effect (Fig. 9b). We further analyzed the evolutionary conservation of the sequence of the miR-19a/b target sites. Sequence alignment of two miR-19a/b target sites in *Dmrt1* 3’UTR in both mammals and teleosts showed that the site 2 is conserved in the teleosts, suggesting an importance of miR-19a/b in the fish *Dmrt1* regulation. Thus, miR-19a/b potentially regulates *Dmrt1* expression through targeting both sites in the 3’UTR of zebrafish *Dmrt1*. This result was consistent with the reverse relationship of expression levels between *Dmrt1* and miR-19a/b in gonads (Suppl. Fig. 3). Further analysis *in vivo* using transgenic animals will confirm the regulation.

## Discussion

Understanding evolution and functions of small RNAs in broadly biological processes is of intriguing interest. This study provides a large quantity of small RNAs to reveal dynamic flux of both piRNAs and miRNAs during gonad reversal from ovary to testis via ovotestis, suggesting roles of these small RNAs in sexual reversal. These dynamic changes during gonad reversal reflect in the following two aspects. First, a considerable amount of piRNAs and miRNAs are differentially or even specially expressed during gonad reversal. In the meanwhile, relevant pathway genes are also differentially expressed during the reversal. Second, differential biogenesis of both piRNAs and miRNAs is associated with gonad reversal, including changes in the 5’ U bias, size, strand bias (guide or star), isomiRs and abundance. These observations indicate that post-transcriptional processing of small RNAs is involved in sexual reversal. Our previous study in zebrafish gonadal piRNAs support differential biogenesis of small RNAs in germline^69^. In addition, differential isomiRs have been detected in human lymphoblastoid cell lines across populations by gende^70^.

The piRNA machinery has been shown to play an important role in germline defense by repression of transposable elements^71^,^72^. Recent studies uncover new functions of piRNA pathways in gene regulation. piRNAs can negatively regulate mRNAs at the posttranscriptional level in gametogenesis^72^,^73^ and embryogenesis^74^,^75^. Even a single piRNA from the sex-determining region of the W chromosome (fem piRNA) plays an important role in sex determination in silkworms^35^. The degradation of mRNAs by piRNAs requires PIWIL1 (also known as MIWI)^72^,^76^. In this study, we have identified a large amount of piRNAs specifically or broadly expressed in ovary, ovotestis and testis. Their functions in sex reversal are still unknown. Further identification of the piRNA targets and their biogenesis during gonad reversal will uncover piRNA functions in sexual development in vertebrates.

Notably here we reveal dynamic expansions of miRNAs especially in vertebrates through analysis of miRNA duplications across the *Eukarya*. The expansion of miRNAs at least includes *de novo* emergence of novel miRNAs, local duplications at original locus, (re)transposition, and whole-genome duplications. Particularly over 1,000 novel miRNA candidates during gonad reversal have been identified in the teleost fish and human^53^, which markedly contributes to the miRNA expansion. Several resources of new miRNA genes have been proposed, such as transposable elements, tRNA, mirror miRNAs, unstructured transcripts that gradually evolve into novel hairpin structures^77^,^78^. However, how new miRNA genes emerged remains an open question. The birth, death and adaptation of new miRNAs are a dynamic evolutionary process, so as to gain a net increase of new miRNA genes in a genome. The net gain rate is around 0.3-1 Myr per new gene in *Drosophila*^54^,^56^,^57^. In the teleost fishes, there is likely to be lower or closer to the net gain rate of new miRNA genes observed in *Drosophila*.

A great number of novel miRNAs have been identified in this study and others^53^, which benefited from the next generation sequencing (NGS) technology. It can overcome the insufficiency in sensitivity and specificity of several traditional technologies such as cloning and micro-array. Since its ability to detect low expressed genes from the background noise, it is very suitable for detection of small RNA expressions. Still, the regulation relationship between these miRNAs and their targets remains to be studied. In addition, species-specific or novel miRNAs, indicate different regulation modes of miRNAs and their targets in individual species. Nevertheless, the databases and information in this study provide a resource for investigation of miRNA functions in sexual development.

Our study also provides an evolutionary trajectory of miRNA gene clusters. We provide clear evidence supporting that the conserved miR-17-92 cluster in the vertebrate lineage originated from a singlet gene, miR-92, in *Tetranychus urticae* and *Daphnia pulex* from the *Chelicerata* in *Arthropoda* around 580 Mya. The miR-92 was then duplicated to get a doublet miR-92a/b in the genomes of *Drosophila* and silkworm in the *Hexapoda* in *Arthropoda*. A local duplication occurred again to form a triplet and then a non-local duplication took place, leading to generate two clusters (miR-92a/c/d, miR-92b/e-1/e-2) in sea squirt around ~550 Mya. In the following evolutionary processes, the miR-92 differentiated into an original miR-17-92 cluster with eight members in the jawless vertebrate, such as in lamprey in around ~500 Mya. The process is likely to accompany with duplication, loss and mutations in the miR-92 gene. The lamprey miR-17-92 cluster contains original members, from which three distinct families of the miR-17-92 formed in the jawed vertebrates. In the vertebrate linages, more miR-17-92 clusters were generated along with multiple whole genome duplication events. Particularly, in the teleost fish, fish-specific genome duplication (3R) occurred around ~400 Mya^79^,^80^, leading to emergence of four homologous clusters of the miR-17-92. Meanwhile, some newly emerged clusters probably have been lost. In addition, the cluster duplications tend to occur within a chromosome in the teleost fishes. For instance, there are three linked miR-17-92 clusters on the chromosome 7 in the swamp eel. These analyses provide an evolution model for the miR-17-92 (Fig. 7c), which interprets evolutionary trajectory of the miR-17-92 cluster in the *Eukarya*. The original miR-92 as an ancestor of the miR-17-92 cluster and its evolutionary trajectory are typical examples of the miRNA gene formation. Certainly, there could be other mechanisms of miRNA evolution to be explored. Nevertheless, our knowledge extends our understanding of evolution and functions of miRNAs in the germline development.

## Methods

### Ethics statement

All animal experiments and methods were performed in accordance with the relevant approved guidelines and regulations, as well as under the approval of the Ethics Committee of Wuhan University.

### Tissue preparation and total RNA extraction

The swamp eels (*Monopterus albus*) were obtained from markets in Wuhan, China. Their phenotypic sexes (ovary, ovotestis and testis) were confirmed by histological sectioning and microscope analysis. The total RNAs from gonads of swamp eel were extracted using TRIZOL (Invitrogen, CA, USA) according to the manufacturer’s protocol. Total RNA quantity was measured by Agilent Technologies 2100 with an RNA Integrity Number value greater than 8. We also ran a 1% agarose gel and judged the integrity of RNA.

### Small RNA library construction and Next generation Sequencing

Small RNA isolation and library construction were performed as described by Gyungsoon Park *et al*^81^. Small RNA samples were sequenced using Illumina HiSeq 2000 at BGI (Beijing Genomics Institution, Shenzhen). Briefly, 18–30 nt fraction of total RNAs was collected from 15% TBE urea polyacrylamide gel. The 5’RNA adapter was ligated to the small RNAs by T4 RNA ligase. After size fractionated and precipitated, the ligation products were subsequently ligated with 3’RNA adapter. Small RNAs with 5’ and 3’ adaptors were reversed into cDNAs using the adaptor primers by Superscript II reverse transcriptase (Invitrogen) and was PCR-amplified by the following conditions: 98 °C for 30 sec; 10 sec at 98 °C and 15 sec at 72 °C for 15 cycles; 10 min at 72 °C. The PCR products were purified by 6% PAGE gels. The cDNAs (small RNA and adaptors) were sequenced on the Illumina HiSeq 2000. Base calling was then performed using CASAVA v1.8.2.

### Data filtering and small RNA annotation

The raw data were a 50 nt long sequence-combination of around 30 nt small RNA sequence and remaining 3’ end adapter. There were also irregular sequences, caused by library preparation and sequencing, including lower quality reads, reads with 5’ adapter decontamination, reads with 3’adapter missing, inserted fragments, poly (A) stretches, and reads smaller than 18 nt. After removing the irregular sequences, all clean reads were aligned onto the swamp eel genome by SOAP de novo^82^. During the alignment, no mismatch was allowed. Each aligned locus was further annotated by comparing to gene annotation file either from UCSC database or De novo annotated from the genome project. The loci were compared to repeat annotation file (http://repeatmasker.org).

In the small RNAs library, there are still degraded long chain RNA molecules included, for example, rRNA, snoRNA, tRNA, snRNA etc. By aligning to the reference genome, Rfam (rfam.sanger.ac.uk) and GenBank (blast.ncbi.nlm.nih.gov) noncoding database and repeat annotation file (repeatmasker.org), the rRNA, snoRNA, tRNA, snRNA, exon_sence, exon_antisence, intron_sense, intron_antisense and repeat sequence were extracted. During the annotation, a maximum of 2 mismatches is allowed by the alignment. The remaining small RNA sequences include both miRNAs and piRNAs.

For annotation of conserved miRNAs, the small RNAs were aligned to the whole mature metazoan miRNA sequences in miRBase 18.0 (www.mirbase.org/) with a tolerance of two nt mismatchs outside the seed region. In order to account for imperfect Dicer processing that has been typically reported for mature miRNAs, at least 16 nt overlapping between sequence reads and reference miRNAs is required when alignment. Meanwhile, the sequences must be perfectly mapped onto the reference precursors. For each mature miRNA, we picked one sequence with the highest expression as its mature sequence from the several variants, also referred to isomiR. Then we mapped all the candidate miRNAs to the scaffolds of the swamp eel genome to predict their precursors. We obtained the genomic sequences including ~100 bp of flanking sequences on either side, and folded the RNA through miRNA prediction software Mireap (sourceforge.net/projects/mireap/).

There was a distinct subset of small RNAs from three types of gonads with a peak size distribution of 26–28 nt and a preference for a U at position 1. These sequences were aligned to the reference genome, Rfam (rfam.sanger.ac.uk) and GenBank (blast.ncbi.nlm.nih.gov) noncoding database and repeat annotation file (repeatmasker.org). Most of those reads were mapped to the intergenic regions containing transposons, pseudogenes, and other repetitive sequences. These RNAs are classed as the piRNAs.

### Prediction of novel miRNAs

We performed novel miRNA prediction using Mireap, which is a program that identifies both known and novel miRNAs from deeply sequenced small RNA libraries. For each miRNA, around 100 bp of the genomic sequence including miRNA and its flanking sequence on either side was extracted, and run the sequence through RNA folding software of Vienna package. Parameters for novel miRNA prediction were as follows: novel miRNA sequence length: 18-26; reference sequence length: 20–24; depth of Drosha/Dicer cutting site: 3; Maximal copy number of novel miRNAs on reference: 20; Maximal free energy allowed for a novel miRNA precursor: -18 kcal/mol; Maximal space between novel miRNA and miRNA*: 35; Minimal base pairs of novel miRNA and miRNA*: 14; Maximal bulge of novel miRNA and miRNA*: 4; Maximal asymmetry of novel miRNA/miRNA* duplex: 5; Flank sequence length of novel miRNA precursor : 10.

### miRNA expression analysis

Due to the different sequencing depth of each sample, before differential expression analysis, the expression was normalized to 1 million reads. We used scatter plot between two samples to reveal the expression difference in fold-change and *p* value. Heatmaps and hierarchical clustering were performed using R-program by transforming the normalized data to log 2 scales for visualization. Hierarchical clustering and boxplots were performed using R-program version 2.12.0.

For quantitative real-time PCR analysis, small RNAs were extracted from adult tissues from swamp eel and zebrafish by Fast MicroRNA Extraction Kit (BioTeke, Beijing, China) according to the manufacturer’s protocol and reversely transcribed using All-in-One™ microRNA qRT-PCR Detection Kit (Gene Copoeia, USA). Quantitative real-time fluorescent PCR (qRT-PCR) was performed using the ABI Step One Plus thermocycler (Applied Biosystems). The U6 small nuclear RNA was used as an endogenous control. qRT-PCR for miRNAs was performed using universal reverse primer: 5’-GCGAGCACAGAATTAATACGA-3’ and gene specific forward primers. In each assay, 10 μl of qRT-PCR detection mix (Genecopoeia, USA), 2 μl of 0.2 μM specific forward primer, 2 μl of 0.2 μM universal reverse primer, 1 μl of cDNA sample, 4.6 μl RNase-free H_2_O and 0.4 μl 50xROX were incubated at 95 °C for 5 min, and followed by 40 cycles of PCR (95 °C for 10 seconds, 60 °C for 20 seconds, 72 °C for 20 seconds). All reactions were run in triplicates and included negative controls for each gene. The relative amount of miRNA was calculated using the 2^-ΔΔCT^ method, data showed in the mean fold change ± SD of miRNAs in comparison with basic expression of U6. Statistical significance was assessed by Student’s t-test with *p* value <0.05(*), *p* value <0.01(**). All primer sequences were listed in supplementary table 4.

### Construction of expression vectors

The 3’ untranslated region (UTR) fragment of the zebrafish *Dmrt1* was amplified by PCR using gonad cDNA as a template. After digestion by XhoI and NotI, the PCR product was inserted into the 3’ UTR of the Renilla luciferase gene in pSiCHECK-2 vector (Promega) to get the Psi-*Dmrt1*-wild type (Psi- *Dmrt1*-wt). The mutant reporters were constructed by generating mismatches within the complementary sequence of the “seed region” of miRNA. For constructions of Psi-*Dmrt1*-mut1/2/3, mutations were introduced into target site of the Psi-*Dmrt1*-wt directly using a PCR-based protocol. To construct miR-17a/20a/19a/19b expression vectors (pcDNA6.2-EmGFP-17a/20a/19a/19b), pre-miRNA sequences were inserted into the flanking region of pcDNA6.2-GW/EmGFP-miR vector, which could be co-transcribed with EmGFP by CMV promotor and processed into mature miRNA. GFP expression can be used to monitor the transfection efficiency. All constructions were sequenced to verify correct sequences. All primer sequences were listed in supplementary Table S4.

### Cell Culture and Dual-luciferase assay

HEK293T cells were cultured in DMEM with 10% fetal bovine serum (HyClone, South America) under a humid environment with 5% CO_2_ at 37 °C. Before transfection, cells were plated in 48-well at a density of 5 × 10^4^. After 24 h, the transfection complex containing 50 ng of the reporter constructs (Psi-Dmrt1-wt or Psi-Dmrt1-mut), 300 ng of the miRNA vector (pcDNA6.2-17a/20a/19a/19b-GFP or the negative control plasmid pcDNA6.2-Laze), and 3 μL of Lipofectamine 2000 (Invitrogen-Life, USA) in serum-free medium was added, and each transfection reaction was repeated in triplicates. After 6 h, the serum-free medium was replaced with 300 μL of fresh DMEM (10% FBS). Luciferase assays were performed 48 h after transfection with the Dual-Luciferase Reporter Assay Kit (Promega, USA). The luciferase activity of Renilla luciferase was normalized by the firefly luciferase activity. The statistical significance was assessed by Student’s t-test with *p* value <0.05(*), *p* value <0.01(**).

### Phylogenetic analysis

Mature (-5p and -3p) and precursor sequences of miRNA members and the genomic location information of miR-17-92 and paralogous clusters in fourteen vertebrates (human, Gorilla, cow, mouse, platypus, chicken, lizard, toad, zebrafish, medaka, catfish, tetraodon, takifugu and salmon) were obtained from the miRBase 21.0. The mature and precursor miRNA sequences of miR-17-92 cluster in swamp eel were obtained from genome sequence database in our laboratory. Precursors of miR-17-92 cluster and the paralogous groups were aligned with Clustal X 2.0.12^83^ by multiple alignments, and a Neighbor Joining (NJ) phylogenetic tree based on miRNA genes was constructed in MEGA 6.0^84^ by using bootstrap with 1,000 replications.

### miRNA duplication analysis

The precursor, mature miRNA data and genomic location information of all the Eukarya were downloaded from miRBase v21.0. The miRNAs conservation analysis was performed using R-program version 2.12.0. The miRNA precursors of sixteen vertebrates (human, gorilla, cow, mouse, platypus, chicken, lizard, toad, zebrafish, medaka, catfish, tetraodon, takifugu, salmon, lamprey and swamp eel) were used for analysis of miRNA duplications. The duplicated miRNA genes consist of a group of miRNA homologs, which include miR-n-1, …-n-n; miR-na, -nb, …-nz; n = 1, 2, ..n. The homolog sequences and genomic locations of miR-17-92 members were searched in Eukarya. Consensus sequence of miR-92 in Eukarya was analyzed by Weblogo (http://weblogo.berkeley.edu/).

## Author Contributions

Conceived and designed the experiments: R.Z H.C. Performed the experiments: J.L M.L Y.S Q.H. Analyzed the data: R.Z J.L. Wrote the paper: R.Z J.L.

## Additional Information

**How to cite this article**: Liu, J. et al. Dynamic evolution and biogenesis of small RNAs during sex reversal. *Sci. Rep.* **5**, 09999; doi: 10.1038/srep09999 (2015).

## Supplementary Material

Supporting Information Supplementary Figure S1-S3 and Supplementary Tables S1 and S2
