# Supplementary material for: Dynamic evolution and biogenesis of small RNAs during sex reversal
Source: Sci Rep. 2015 May 6;5:9999. doi: 10.1038/srep09999 (PMC4421800; doi:10.1038/srep09999)
Supplement: Supporting Information — Supplementary Figure S1-S3 and Supplementary Tables S1 and S2 [file srep09999-s1.pdf]

Supplementary information for

Dynamic evolution and biogenesis of small RNAs during sex reversal

Running title:

Small RNA and sex reversal

Jie Liu, Majing Luo, Yue Sheng, Qiang Hong, Hanhua Cheng*, Rongjia Zhou*

Department of Genetics, College of Life Sciences, Wuhan University, Wuhan 430072, P. R. China

*Corresponding authors: Professors Rongjia Zhou and Hanhua Cheng, Department of Genetics and Center for Developmental Biology, College of Life Sciences, Wuhan University, Wuhan 430072, P. R. China, Fax: 0086-27-68756253, E-mail: rjzhou@whu.edu.cn, hhcheng@whu.edu.cn

Including:

Supplementary Table S1

Supplementary Table S2

Supplementary Table S2

Supplementary Table S2

Supplementary Figure S1

Supplementary Figure S2

Supplementary Figure S3

Supplementary Table S1 | Small RNA classifications

	Ovary		Ovotestis		Testis	
Type	Reads number	%	Reads number	%	Reads number	%
Exon_antisense	556,913	4.69	558,917	4.20	1,178,458	5.27
Exon_sense	739,801	6.23	656,688	4.93	1,857,518	8.31
Intron_antisense	401,011	3.37	457,397	3.43	926,393	4.15
Intron_sense	1,416,121	11.92	1,602,473	12.03	3,706,984	16.59
miRNA	709,265	5.97	1,065,014	7.99	1,707,05	7.64
rRNA	2,591,369	21.81	2,493,059	18.71	367,666	1.65
Repeat	8,512	0.07	7,531	0.06	43,213	0.19
snRNA	6,139	0.05	9,352	0.07	16,703	0.07
snoRNA	2,865	0.02	2,839	0.02	6,942	0.03
tRNA	213,729	1.80	170,644	1.28	629,270	2.82
piRNA	5,222,740	44.07	6,275,011	47.1	11,833,225	52.95
Novel miRNA	14,243	0.12	11,309	0.08	37,063	0.17
Total	11,882,708	100	13,321,543	100	22,347,593	100

Supplementary Table S2 | Conserved miRNA information

Conserved miRNA ID	Precursors	MFE	Mature ID		Mature sequences	
			Mature miRNA -5p	Mature miRNA -3p	Mature miRNA -5p	Mature miRNA -3p
let-7a-1	CCTAGCAAGGTGAGGTAGTAGGTTGTATAGTTTGGGGGATGGAGTAAATCCTACCCAGGTGATAACTATACAGTCTATTACCTTCCTTGAGAGG	-50.8	let-7a-5p	let-7a-1-3p	TGAGGTAGTAGGTTGTATAGTT	CTATACAGTCTATTACCTT
let-7a-2	CACTGTGGGCTGAGGTAGTAGGTTGTATAGTTTTAGGGTCACACCCGCACAGGGAGATAACTATTCAACCTACTGTCTTTCTCAAAGTGTC	-36.5	let-7a-5p	let-7a-2-3p	TGAGGTAGTAGGTTGTATAGTT	CTATTCAACCTACTGTCTTTC
let-7a-3	GCTCCCAGGTTGAGGTAGTAGGTTGTATAGTTGAGAATTACACCCCAGGAGATAACTGTACAACCTCCTAGCTTTCCCTGAGGTATG	-33.01	let-7a-5p	let-7a-3-3p	TGAGGTAGTAGGTTGTATAGTT	CTGTACAACCTCCTAGCTTTCC
let-7a-4	CTCCGCAGGTTGAGGTAGTAGGTTGTATAGTTCAGAGTGACACCAAAGGAGATAACTGTACAGCCTCCTAGCTTTCCCTGAGGACAC	-33.01	let-7a-5p	let-7a-4-3p	TGAGGTAGTAGGTTGTATAGTT	CTGTACAGCCTCCTAGCTTTCC
let-7a-5	TCCTTTGGGGTGAGGTAGTAGGTTGTATAGTTTTAGGGTCCTTCCCAAGTTGTCAGATGACTATACAACTTACTGTCTTTCCTGGAGCGACT	-38.3	let-7a-5p	let-7a-5-3p	TGAGGTAGTAGGTTGTATAGTT	CTATACAGTCTATTGCCTTCCT
let-7a-6	TCTGTCAGGGTGAGGTAGTAGGTTGTATAGTTTGGTGGGTGGGATTGCACCCTGCTCAGGTGATAACTATACAGTCTATTGCCTTCCTTGAGGAGCTC	-45.1	let-7a-5p	-	TGAGGTAGTAGGTTGTATAGTT	-
let-7a-7	TCCTTTGGGGTGAGGTAGTAGGTTGTATAGTTTTAGGGTCATACCCTTCGTGTCAGATAACTATACAACTTACTGTCTTTCCTAAAGTGGCT	-45.1	let-7a-5p	-	TGAGGTAGTAGGTTGTATAGTT	-
let-7b-1	TCGTACAGGGTGAGGTAGTAGGTTGTGTGGTTTCAGGGTTGTGATTTTACCCCATCAGGAGCTAACTATACAACCTACTGCCTTCCCTGGAGGGCA	-49.1	let-7b-5p	let-7b-3p	TGAGGTAGTAGGTTGTGTGG	CTATACAGTCTATTGCCTTCCT
let-7b-2	TCGTACGGGGTGAGGTAGTAGGTTGTGTGGTTTCAGGGTAGTGATTTTGCCCAATCAGGAGATAACTATACAACCTACTGCCTTCCCCGAAGGGCA	-50.8	let-7b-5p	let-7b-3p	TGAGGTAGTAGGTTGTGTGG	CTATACAGTCTATTGCCTTCCT
let-7c	GCATCCGGGTTGAGGTAGTAGGTTGTATGGTTTAGAATTACACCCTGGGAGTTAACTGTACAACCTTCTAGCTTTCCTTGGAGTA	-33.91	let-7c-5p	let-7c-3p	TGAGGTAGTAGGTTGTATGGTT	CTGTACAACCTTCTAGCTTTCCT
let-7d-1	CTCTGCAGTGTGAGGTAGTTGGTTGTATGGTTTCGCATAATAAACAGCTTGGAGATAACTGTACAACCTTCTAGCTTTCCCTGCGGTGT	-31.2	let-7d-5p	let-7d-3p	TGAGGTAGTTGGTTGTATGGTT	CTGTACAACCTTCTAGCTTTCC
let-7d-2	CTCTGCGGTGTGAGGTAGTTGGTTGTATGGTTTCGCATAATAAACAGCCTGGAGATAACTGTACAACCTTCTAGCTTTCCCTGCGGAGT	-33.91	let-7d-5p	let-7d-3p	TGAGGTAGTTGGTTGTATGGTT	CTGTACAACCTTCTAGCTTTCC
let-7j	CACTTTGGTCTGAGGTAGTTGTTTGTACAGTTTGAGGGTCTGTGATTCTGCCCCATACAGGAGCTAACTGTACAAGTGACTGCCTTGCCCACGGTT	-36.7	let-7j-5p	-	TGAGGTAGTTGTTTGTACAGTT	-
let-7k-1	TCCTTAAGGCTGAGGTAGTAGATTGAATAGTTGTGGGGTCTGAGTCCTCTCTCTGAGATAACTATACAATCTACTGTCTTTCCTAAGGAG	-43.5	let-7k-5p	-	TGAGGTAGTTGTTTGTACAGTT	-
let-7k-2	TCCTTGGGGCTGAGGTAGTAGATTGAATAGTTGTGGGGTTGTGTAACCTCTTTTTGAGATAACTATACAATCTACTGTCTTTCCCAAGGAA	-48.2	let-7k-5p	-	TGAGGTAGTTGTTTGTACAGTT	-
miR-103a-1	GCTCTACGCTTTTAGCCTCTTTACAGTGCTGCCTTGTCTGATCATGTTCAAGCAGCATTGTACAGGGCTATGACAGCATAGAG	-35.7	miR-103a-5p	miR-103a-3p	AGCAGCATTGTACAGGGCTATGA	AGCCTCTTTACGGTGCTGCCTTG
miR-103a-2	CTTTGCATTCAGCCTCTTTACGGTGCTGCCTTGTGGCATCTTGATCAAGCAGCATTGTACAGGGCTATGAAGGCATAGAG	-31.7	miR-103a-5p	miR-103a-3p	AGCAGCATTGTACAGGGCTATGA	AGCCTCTTTACGGTGCTGCCTTG
miR-107	GTGTGCAGTGAGCTTCTTTACAGTGTTGCCTTGTGGCATGGCGATCAAGCAGCATTGTACAGGGCTATCACTGCATA	-35.5	-	miR-107-3p	-	AGCAGCATTGTACAGGGCTA
miR-10a-1	ACCTATACACACCCTGTAGATCCGAATTTGTGTAAATACAACGGCAGCCACAAGTTCGCATCTTGGGGAGTTTGTAGGTG	-30.99	miR-10a-5p	-	CACCCTGTAGATCCGAATTTGT	-
miR-10a-2	ACCTATACACACCCTGTAGATCCGAATTTGTGTAAATACAACGGCAGCCACAAGTTCGCATCTTGGGGAGTTTGTAGGTG	-30.99	miR-10a-5p	-	CACCCTGTAGATCCGAATTTGT	-
miR-10b-1	CGTCTATATATACCCTGTAGAACCGAATTTGTGTGATAAAACCACAGTCACAGATTCGATTCTAGGGGAGTATATGGTCGA	-33.16	-	miR-10b-3p	-	AGATTCGATTCTAGGGGAGTA
miR-10b-2	TAGTTGTCTATATGTACCCTGTAGAACCGAATTTGTGTGAAGTCCAAACAGTCGCAAATACGTCTCTACAGGAATACATGGGCAA	-32.7	miR-10b-5p	miR-10b-3p	TACCCTGTAGAACCGAATTTGT	AGATTCGATTCTAGGGGAGTA
miR-10c	CTTCTATATCTACCCTGTAGATCCGGATTTGTGTAAAAATCATTAAAACAATCACAAATTCGCTTCTAGGGGAGTATATAGTGGA	-27.61	miR-10c-5p	-	TACCCTGTAGATCCGGATTTGT	-
miR-10d	TGCCTATATATACCCTGTAGAACCGAATGTGTGTGGAGCTGCCTCAGTCACAGATTGGGTTCTAGGGGAGTCTATGGGCGA	-39.1	miR-10d-5p	-	TACCCTGTAGAACCGAATGTGT	-
miR-122	TCCAGAGCTGTGGAGTGTGACAATGGTGTTTGTGTCCTGTATGTCAAACGCCATTATCACACTAAATAGCTACAGT	-33.5	miR-122-5p	-	AAACGCCATTATCACACTAAA	-
miR-1238	GTTTCAGATCATTCTCGTCTGTCTGCTCCAATCTCGCCTGCCTCTGAATGCAAACCCATCTGTGGAAGCTGTCAGCGAATATGAGTGTGGGCC	-20.47	miR-1238-5p	-	ATTCTCGTCTGTCTGCTCCA	-
miR-125a-1	TGCTTCATTGTCCCTGAGACCCTTAACCTGTGATGATATGAAAGGTCACAGGTGAGGTCCTTGGGAACAAAGGTGCAT	-33.4	miR-125a-5p	miR-125a-3p	TCCCTGAGACCCTTAACCTGTG	CAGGTGAGGTCCTTGGGAAC
miR-125a-2	TGCCCCTCTGTCCCTGAGACCCTTAACCTGTGAGGTCAAAGTAGGTCACAGGTGAGGTCCTCGGGAACAGGGCTGCAT	-38.7	miR-125a-5p	miR-125a-3p	TCCCTGAGACCCTTAACCTGTG	CAGGTGAGGTCCTTGGGAAC

miR-125b-1	TTTTTCTCTGTCCCTGAGACCCTAACTTGTGACGTTGTGCTTTCATGTCCACGGGTTAGGCTCTTGGGAGCTGGGAGGGA	-36.3	miR-125b-5p	miR-125b-3p	ACGGGTTAGGCTCTTGGGAGC	TCCCTGAGACCCTAACTTGTGA
miR-125b-2	CTTCACCTGCTCCCTGAGACCCTAACTTGTGAGCTCTCTTGATAAAAAATCACGGGTTAGGCTCTTGGGACGCGGGCGGAGGG	-40.93	miR-125b-5p	miR-125b-3p	ACGGGTTAGGCTCTTGGGAGC	TCCCTGAGACCCTAACTTGTGA
miR-125b-3	CCTCTCTCATTCCCTGAGACCCTAACTTGTGACGTTTGGCTGTGATGTGCACGGGTTGGGTTCTTGGGAGCTGCGAGGGGCA	-41	miR-125b-5p	miR-125b-3p	ACGGGTTAGGCTCTTGGGAGC	TCCCTGAGACCCTAACTTGTGA
miR-128-1	GAGAGGTGGGATACGGGACCGGGACGCTGTCTGAGAGGTCTCTATGAATCTCACAGTGAACCGGTCTCTTTTTCAGCCCCT	-35.7	miR-128-5p	miR-128-3p	TCACAGTGAACCGGTCTCTTT	GGGGGCCGTTACACTGTCAGA
miR-128-2	CAGCAGTAGAGGAGGGGGCCGTTACACTGTCAGAGATGTAGTCTGAGGGTCTCACAGTGAACCGGTCTCTTTTCCTGCTGCT	-42.1	miR-128-5p	miR-128-3p	TCACAGTGAACCGGTCTCTTT	GGGGGCCGTTACACTGTCAGA
miR-130a	TGTTCTTGTCCGTTGCCCTTTTTCTGTTGCACTACTGGACATTGAGATGAGCAGTGCAATATTAAAAGGGCATTGGCTGATGA	-30.8	-	miR-130a-3p	-	CAGTGCAATATTAAAAGGGCAT
miR-130b-1	GTTGTCCAAGGCTCTTTTTCTGTTGTACTACTGTGAAATCAGATGAGCAGTGCAATATTAAAAGGGCATTGGCTGACAA	-27.32	miR-130b-5p	miR-130b-1-3p	GCTCTTTTTCTGTTGTACTAC	CAGTGCAATATTAAAAGGGCAT
miR-130b-2	TCACTGCCTGACACTCTTTACATGTTGCACTACTGAGGGAGCTACAGCAAGCAGTGCAATAACGAAAGGGCATCAGTCAGCTG	-27.6	miR-130b-5p	miR-130b-2-3p	GCTCTTTTTCTGTTGTACTAC	CAGTGCAATAACGAAAGGGCAT
miR-133b	CACGCCTTGCTGTGGCTGGTCAAACGGAACCAAGTCAGGTGTTTGGTCCCCTTCAACCAGCTAAACCAGCTACTGCGTCGT	-30.9	-	miR-133b-3p	-	TTTGGTCCCCTTCAACCAGCTA
miR-133a-1	GCTTTGCTAAAGCTGGTAAAATGGAACCAAATCACCTCTTGAATGGATTTGGTCCCCTTCAACCAGCTGTAGCTATGCTT	-31.6	miR-133a-5p	miR-133a-3p	AGCTGGTAAAATGGAACCAAA	TTGGTCCCCTTCAACCAGCTGT
miR-133a-2	GCTTTGCTAAAGCTGGTAAAATGGAACCAAATCAACTGTTCAATGGATTTGGTCCCCTTCAACCAGCTGTAGCTGTGCAT	-32.1	miR-133a-5p	miR-133a-3p	AGCTGGTAAAATGGAACCAAA	TTGGTCCCCTTCAACCAGCTGT
miR-135a-1	CAGTGCGCTGTATGGCTTTTTATTCCTATCTGACTGTACTAATGGTTCATATAGGGATGGAAGCCATGCAATGCGCTGGG	-41	miR-135a-1-5p	miR-135a-1-3p	TATGGCTTTTTATTCCTATCTGA	ATATAGGGATGGAAGCCATGC
miR-135a-2	ACTTGTGTCTTATGGCTTTCTATTCCTATGTGAATTCTTTCTAACATGTCATGTAGGGTTTAAAGCCATTGGATACAACATG	-29.46	miR-135a-2-5p	miR-135a-2-3p	TATGGCTTTCTATTCCTATGTGA	ATGTAGGGTTTAAAGCCATTGG
miR-135c-1	ACTTGTGTCTTATGGCTTTCTATTCCTATGTGAATTCTTTCTAACATGTCATGTAGGGTTTAAAGCCATTGGATACAACATG	-29.46	miR-135c-5p	miR-135c-3p	TATGGCTTTCTATTCCTATGTG	ATGTAGGGTTTAAAGCCATTGG
miR-135c-2	TGCTGTGTTTTATGGCTTTCTATTCCTATGTGATTTTCTATAGCATGTCACATAGGGTCTAAAGCCATTGGGTACAGAG	-32.1	miR-135c-5p	miR-135c-3p	TATGGCTTTCTATTCCTATGTG	ATGTAGGGTTTAAAGCCATTGG
miR-137a	ACTCTCTTCGGTGACGGGTATTCTTGGGTGGATAATACGGATCACGTTGTTATTGCTTAAGAATACGCGTAGTCGAGGAGAG	-41.9	-	miR-137a-3p	-	TTATTGCTTGAGAATACGCGTA
miR-137b	CTCCGGTCTCGACCACGGGTATTCTTGGGTTGATAATACAGATGTGGATGTTATTGCTTGAGAATACGCGTAGTCGAGTGGAA	-36.2	-	miR-137b-3p	-	TTATTGCTTAAGAATACGCGTAG
miR-138-1	GGAGAGGGTCAGCTGGTGTTGTGAATCAGGCCGCTGAAGTGTCAGACACGGCTTTTTCATAACACCAGGGTTCCTCCCT	-40.9	miR-138-5p	-	AGCTGGTGTTGTGAATCAGGC	-
miR-138-2	GGGGCGGGACAGCTGGTGTTGTGAATCAGGCCGCCACAGTGCAGGGACCGGCTACTTCCCAACACCAGGGTCTCACCAT	-36.2	miR-138-5p	-	AGCTGGTGTTGTGAATCAGGC	-
miR-138-3	TGTGTGCTACAGCTGGTGTTGTGAATCAGGCCGATGACAAACACCTGCTATAACCCGGCTATTTCACAACACCAGGGTGGCACTGCAC	-41.35	miR-138-5p	-	AGCTGGTGTTGTGAATCAGGC	-
miR-138b-1	TGTGCCATGGTTGTGCAGTTTCCAAAACCAGACAAGGATCCCTCTGGTTTTGTGAATCAGCTGACATGGTATG	-27.6	-	miR-138b-3p	-	CTGGTTTTGTGAATCAGCTG
miR-138b-9	TGTGCCATGGTTCTGCAGTTTCCAAAACCAGACAAGGATCCCTCTGGTTTTGTGAATCAGCTGACATGGTATG	-27.6	-	miR-138b-3p	-	CTGGTTTTGTGAATCAGCTG
miR-138b-10	TGTGCCATGGTTGTGCAGTTTCCAAAACCAGAAAAGGATCCCTCTGGTTTTGTGAATCAGCTGACATGGTATG	-27.7	-	miR-138b-3p	-	CTGGTTTTGTGAATCAGCTG
miR-138b-11	TGTGTCATGGTTATGCAGTTTCCAAAACCAGACAAGGATCTATCTGGTTTTGTGAATCAGCTGACATGGTATG	-23	-	miR-138b-3p	-	CTGGTTTTGTGAATCAGCTG
miR-138b-2	TGTGCCATGGTTGTGCAGTTTCCAAAACCAGACAAGGATCCCTCTGGTTTTGTGAATCAGCTGACATGGTATG	-27.6	-	miR-138b-3p	-	CTGGTTTTGTGAATCAGCTG
miR-138b-3	TGTGCCATGGTTGTGCGGTTTCTAAAACCAGACAAGGATCCCTCTGGTTTTGTGAATCAGCTGACATGTTATG	-21	-	miR-138b-3p	-	CTGGTTTTGTGAATCAGCTG
miR-138b-4	TGTGCCATGCTTGTGCAATTTCCAAAACCAGACAAGGATCCCTCTGGTTTTGTGAATCAGCTGACATGGTATG	-26.6	-	miR-138b-3p	-	CTGGTTTTGTGAATCAGCTG
miR-138b-5	TGTGCCATGGTTGTGTAGTTTCCAAAACCAGACAAGGATCCCTCTGGTTTTGTGAATCAGCTGACATGGTATG	-27.6	-	miR-138b-3p	-	CTGGTTTTGTGAATCAGCTG
miR-138b-6	TGTGCCATGGTTGTGTGGTTTCTAAAACCAGACAGGGATCCCTCTGGTTTTGTGAATCAGCTGACATGGTATG	-31.6	-	miR-138b-3p	-	CTGGTTTTGTGAATCAGCTG
miR-138b-7	TGTGCCATGGTTGTGCAGTTTCCAAAACTAGACAAGGATCCATCTGGTTTTGTGAATCAGCTGACATGGTAGG	-24	-	miR-138b-3p	-	CTGGTTTTGTGAATCAGCTG
miR-138b-8	TGTGCCATGGTTGTGCAGTTTCCAAAACCAGACAGGGATCCCTCTGGTTTTGTGAATCAGCTGACATGGTATG	-31	-	miR-138b-3p	-	CTGGTTTTGTGAATCAGCTG
miR-139	TTGGTTGTATTCTACAGTGCATGTGTCTCCAGTATGTTAGTGATGCTACTGGAGACCCAGCTCTGTTGGAATAACAACCA	-39.5	miR-139-5p	miR-139-3p	TCTACAGTGCATGTGTCTCCAG	TGGAGACCCAGCTCTGTTGGA
miR-140	AGTGTCACGTCAGTGGTTTTACCCTATGGTAGGTGACATCATGCTGTTCTACCACAGGGTAGAACCACGGACGGGATGTTGG	-42.8	miR-140-5p	miR-140-3p	CAGTGGTTTTACCCTATGGTAG	ACCACAGGGTAGAACCACGGAC
miR-142-1	TGTTGTCACCCATAAAGTAGAAAGCACTACTAAACTTTACTGCACAGTGTAGTGTTTCCTACTTTATGGATGAGTATAC	-28.87	miR-142-5p	miR-142-3p	CATAAAGTAGAAAGCACTACT	TGTAGTGTTTCCTACTTTATGG
miR-142-2	TGCAGTCATCCATAAAGTAGAAAGCACTACTAAACTCCTCACCACAGTGTAGTGTTTCCTACTTTATGGATGAGTGTAC	-38.97	miR-142-5p	miR-142-3p	CATAAAGTAGAAAGCACTACT	TGTAGTGTTTCCTACTTTATGG

miR-143	TGTGGCCCATGGTGCAGTGCTGCATCTCTGGTCAGTTGGTAGTCTGAGATGAAGCACTGTAGCTCGGGACGGAG	-31.3	miR-143-5p	miR-143-3p	GGTGCAGTGCTGCATCTCTGG	TGAGATGAAGCACTGTAGCT
miR-144	GCCCTGGACAGGATATCATCTTATACTGTAAGTTTATTAAAGAGACACTACAGTATAGATGATGTACTATCCAGGGT	-37	miR-144-5p	miR-144-3p	GGATATCATCTTATACTGTAAGT	CTACAGTATAGATGATGTAC
miR-145	CTCTCCTGGGGTCCAGTTTTCCCAGGAATCCCTTGACCTATCAGAAAGGGGGATTCCTGGAAATACTGTTCTTGGGGGTGAG	-39.4	miR-145-5p	miR-145-3p	GTCCAGTTTTCCCAGGAATCCC	GGATTCCTGGAAATACTGTTCT
miR-146a	TGCTCTACTATGAGAACTGAATTCCATAGATGGTGGCTACTTCAGGTGTCATCTATGGGCTCAGTTCTTTTGGCATGGTAC	-33.1	miR-146a-5p	-	TGAGAACTGAATTCCATAGATGG	-
miR-148	TTTCCAAATAAAGTTCTGTGATACACTTAGACTCTAATTGCCTGCAGTCAGTGCATTACAGAACTTTGTTTTGGGAGT	-31.6	miR-148-5p	miR-148-3p	AAGTTCTGTGATACACTTAGACT	TCAGTGCATTACAGAACTTTG
miR-150	TGGTCTGGTCACTCCCAATCCTTGTACCAGTGTCTTGATACCAAAGTGACGCTGGGCAGGTTTGGGGGGGGCTTTGCTAA	-37.32	miR-150-5p	-	ACTCCCAATCCTTGTACCAGTG	-
miR-153-1	CGGTTGCCAGTGTCATTTTTGTGATGTTGCAGCTAGTAATATAAGCCCAGTTGCATAGTCACAAAAGTGATCATTGGAGACT	-36.7	-	miR-153-3p		TTGCATAGTCACAAAAGTGATC
miR-153-2	CGGTTGCCAGTGTCATTTTTGTGATGTTGCAGCTAGTAATATGAGCCCAGTTGCATAGTCACAAAAGTGATCATTGGAAACT	-36.2	-	miR-153-3p		TTGCATAGTCACAAAAGTGATC
miR-155	CAGATTGAGGATAATGCTAATCGTGATAGGGGTTGTTCATTACCAGACACCTAACATGTTAGCATTAGCTTCGCTTTGG	-28.3	miR-155-5p	miR-155-3p	ACCTAACATGTTAGCATTAG	ATAATGCTAATCGTGATAGG
miR-15a	GGTGATGCTGTAGCAGCACGGAATGGTTTGTGGGTTACACTGAGATACAGGCCATACTGTGCTGCTGCATTAAAACCT	-40.5	miR-15a-5p	miR-15a-3p	TAGCAGCACGGAATGGTTTGT	CAGGCCATACTGTGCTGCTGCA
miR-15b-1	TAGACTGCTATAGCAGCGCATCATGGTTTGAAACATTGCGGAAAAGGTGCGAACCATTATTTGCTGCTTTAGAATTTTAA	-24.3	miR-15b-1-5p	miR-15b-3p	TAGCAGCGCATCATGGTTTGA	TGCGAACCATTATTTGCTGCTTT
miR-15b-2	GGGTGCACTCTAGCAGCACATCATGTTTTGCAGATATGTACTGAATCACTCCAAAACATTATGCGCTGCTACTGTGAGCTCA	-31.9	miR-15b-2-5p	miR-15b-3p	TAGCAGCACATCATGTTTTGCA	TGCGAACCATTATTTGCTGCTTT
miR-16a	TGCCACGCTTTAGCAGCACGTAAATATTGGTGTGTGACAATAGACCCCAACCCCAATATTAGCAGTGCTGCTTCAGTGTGGCTGG	-39.89	miR-16a-5p	miR-16a-3p	TAGCAGCACGTAAATATTGGT	CAATATTAGCAGTGCTGCTTCA
miR-1657	CATTAAAAGTTACTAGTGTAGTTTGTTGGTGTGGCATTAAACAGATAATGAAATTATACCAAGGGATTAAGCGGTAGAAAATGAATGA	-22.3	miR-1657-5p	-	TACTAGTGTAGTTTGTTGGTGTGG	-
miR-16b	CACTTTACTGTAGCAGCACGTAAATATTGGAGTTAAGACTAAGTGAAGCCTCCAGTATTGATCGTGCTGCTGAAGCAAAGCTGA	-33.53	miR-16b-5p	miR-16b-3p	TAGCAGCACGTAAATATTGGAG	CCAGTATTGATCGTGCTGCTGAA
miR-17a-1	TGTGTATTGTCAAAGTGCTTACAGTGCAGGTAGTACTGTGTAATACCTACTGCAGTGAAGGCACTTACAGCAATACACT	-33.5	miR-17a-5p	miR-17a-1-3p	CAAAGTGCTTACAGTGCAGGTA	TACTGCAGTGAAGGCACTTACA
miR-17a-2	AGTGTAGTGCCAAAGTGCTTACAGTGCAGGTAGTTTTATAAGATCTACTGCAGTGAAGGCACTTTCAGCACTGTTC	-30.6	miR-17a-5p	miR-17a-2-3p	CAAAGTGCTTACAGTGCAGGTA	ACTGCAGTGAAGGCACTTTC
miR-17a-3	TGTGTATTGTCAAAGTGCTTACAGTGCAGGTAGTACTGTGTAATACCTACTGCAGTGAAGGCACTTACAGCAATACACT	-33.5	miR-17a-5p	-	CAAAGTGCTTACAGTGCAGGTA	-
miR-1814a	CTGCTCTGTTGTTTTTTTAGGTTTTGATTTTTTGTAGCATTGGATGAGAAAATCAAAGCCTAAAGAAAATACAGCGCTG	-29.8	miR-1814a-5p	-	GTTTTTTTAGGTTTTGATTTT	-
miR-181a-1	TGCCTCGGTGAACATTCAACGCTGTCGGTGAGTTTGGTATGGATCACATAAAAACCATCGACCGTTGACTGTGCCCCGCGGCTC	-36.7	miR-181a-5p	miR-181a-1-3p	AACATTCAACGCTGTCGGTGAG	ACCATCGACCGTTGACTGTGC
miR-181a-2	CGCCCCAGTGAACATTCAACGCTGTCGGTGAGTTTGAGCTAAATTGAAAAAACCATCGACCGTTGATTGTACCCTGTGGCCAG	-32.3	miR-181a-5p	miR-181a-2-3p	AACATTCAACGCTGTCGGTGAG	ACCATCGACCGTTGATTGTACC
miR-181a-3	AGTCTTGGGGAACATTCAACGCTGTCGGTGAGTTTGTGACTCAGTGAAACCATCGAGTGTTGAGTGTACCCTTACACTTGTGCTGACA	-31.8	miR-181a-5p	-	AACATTCAACGCTGTCGGTGAG	
miR-181a-4	GCCCACAGTGAACATTCAACGCTGTCGGTGAGTTTGAGCTCTAACTAAAACCATCGACCGTTGATTGTACCCTGAGGGATG	-32	miR-181a-5p	miR-181a-4-3p	AACATTCAACGCTGTCGGTGAG	ACCATCGACCGTTGATTGTACC
miR-181b-2	GGTCACAATCAACATTCATTGCTGTCGCTGGGTTGGACTGTGTAGAAAAGCTCAATGAACAATGAGTGCAACTGTGGCCC	-27.3	miR-181b-2-5p	-	AACATTCATTGCTGTCGCTGGGT	-
miR-181b-1	GGTCACAATCAACATTCATTGCTGTCGGTGGGTTTAACTGTGTGGAAGAGCTCACTGAACGATGAATGCAACTGTGTCCCA	-31	miR-181b-5p	miR-181b-1-3p	AACATTCATTGCTGTCGGTGG	CTCACTGAACGATGAATGCAA
miR-181b-3	GGCCGCAATAAACATTCATTGCTGTCGGTGGGTTTACATAAGAATAACTCACTGATCAATGAATGCAGACTGCGGTTC	-32.5	miR-181b-5p	miR-181b-3-3p	AACATTCATTGCTGTCGGTGG	CTCACTGATCAATGAATGCAG
miR-181c	GTTTCTGGTTCACATTCATCGCTGTCGGTGGGTTCATGATGCGATCAACCTGTCGGTCGATGAATGACAACCGTGACC	-26.9	miR-181c-5p	-	CACATTCATCGCTGTCGGTGGG	-
miR-182	TCTGGTGGTGTTTGGCAATGGTAGAACTCACACTGGTGAGGTAGATGGATCCGGTGGTTCTAGACTTGCCAACTACTGCTTGAG	-30.44	miR-182-5p	-	TTTGGCAATGGTAGAACTCACA	-
miR-183-1	CTGTTCTGTGTATGGCACTGGTAGAATTCACTGTGAGAGCTCACTATCAGTGAATTACCATAGGGCCATAAACAGAGCAGA	-38.2	miR-183-5p	-	TATGGCACTGGTAGAATTCACTG	-
miR-183-2	CTATTCTGTGTATGGCACTGGTAGAATTCACTGTCACAGCAGGACATCAGTGAATTACCATAGGGCCATAAACAGAGTAGA	-34.84	miR-183-5p	-	TATGGCACTGGTAGAATTCACTG	-
miR-184-1	TTGCTCACATCTCCTTATCACTTTTCCAGCCCAGCTATGTGTTCTGTATCTGTTGGACGGAGAACTGATAAGGGCATGTGCATGA	-29.49	miR-184-5p	miR-184-3p	TTATCACTTTTCCAGCCCAGC	TGGACGGAGAACTGATAAGGGC
miR-184-2	TGAGCACATCTCCTCATCACTTTTCCAGCCCAGCTATCTATCAAATGTTTGTTGGACGGAGAACTGATAAGGGTATGTGTCTG	-28.4	miR-184-5p	miR-184-3p	TTATCACTTTTCCAGCCCAGC	TGGACGGAGAACTGATAAGGGC
miR-18a-1	GTCTTTGTGCTAAGGTGCATCTAGTGCAGATAGTGAAATAGACTAGCACCTACTGCCCTAAGTGCTCCTTCTGGCATAAGGGG	-31.3	miR-18a-5p	miR-18a-3p	TAAGGTGCATCTAGTGCAGATA	ACTGCCCTAAGTGCTCCTTCT
miR-18a-2	GTTCTTATGCTAAGGTGCATCTAGTGCAGATAGTGAACTAGACGAGCACCTACTGCCCTAAGTGCTCCTTCTGGCATACGGTGCTGGAACT	-29.91	miR-18a-5p	miR-18a-3p	TAAGGTGCATCTAGTGCAGATA	ACTGCCCTAAGTGCTCCTTCT
miR-18a-3	GTCTTTGTGCTAAGGTGCATCTAGTGCAGATAGTGAAATAGACTAGCACCTACTGCCCTAAGTGCTCCTTCTGGCATAAGGGG	-31.3	miR-18a-5p	miR-18a-3p	TAAGGTGCATCTAGTGCAGATA	ACTGCCCTAAGTGCTCCTTCT

miR-190-1	GGGCTCTGTCTGATATGTTTGATATATTAGGTTGTTATTCAGTCCCAACTGTATATCAAACATATTCCTACAGTGTCCC	-30.8	miR-190-5p	miR-190-3p	TGATATGTTTGATATATTAGG	ACTGTATATCAAACATATTCCT
miR-190-2	GGGCTCTGTCTGATATGTTTGATATATTAGGTTGTTATTCAGTCCCAACTGTATATCAAACATATTCCTACAGTGTCCC	-30.8	miR-190-5p	miR-190-3p	TGATATGTTTGATATATTAGG	ACTGTATATCAAACATATTCCT
miR-193	TGTCTGAGGCTGGGTCTTTGCGGGCAAGGTGAGTCCTCAGTTAATTCAACTGGCCTACAAAGTCCCAGTTTCTGGCTCA	-36.8	miR-193-5p	miR-193-3p	TGGGTCTTTGCGGGCAAGGTGA	AACTGGCCTACAAAGTCCCAGT
miR-194	GCTTGCTGGCTGTAACAGCAACTCCATGTGGAATCTCTGCTTGTTCCAGTGGAAGTGCTGTTACCTGCAGAGAGCT	-34.9	miR-194-5p	-	TGTAACAGCAACTCCATGTGGA	-
miR-196a-1	TCGAGTGGTTTAGGTAGTTTCATGTTGTTGGGGTCCATTTCAATCTCTGCAACATGAAACTGTCTTAATTGCCCCA	-31.2	miR-196a-1-5p	-	TAGGTAGTTTCATGTTGTTGGG	-
miR-196a-2	AAGCGTGGTTTAGGTAGTTTCATGTTGTTGGGGTTGGCTTCCTGGCTCGGCAACAAGAAACTGCCTTGATTACGTCA	-38.6	miR-196a-2-5p	miR-196a-3p	TAGGTAGTTTCATGTTGTTGGG	ACTGTATATCAAACATATTCCTA
miR-196a-3	GGGCTCTGTCTGATATGTTTGATATATTAGGTTGTTATTCAGTCCCAACTGTATATCAAACATATTCCTACAGTGTCCCG	-30.8	miR-196a-3-5p	miR-196a-3p	TGATATGTTTGATATATTAGG	ACTGTATATCAAACATATTCCTA
miR-196b	TTGTGTGATGTAGGTAGTTTCAAGTTGTTGGGCTGGATTGTTAAGATCACAGGAACCTGAAACTGCCTGAATCACACCA	-35	miR-196b-5p	-	TAGGTAGTTTCAAGTTGTTGGG	-
miR-199a-1	CCCCCGCCTGCCCAGTGTTCAGACTACCTGTTCATCAGGCTACAGCTGAACAGTAGTCTGCACATTGGTTAGGCTGGGCTG	-44	miR-199a-5p	miR-199a-3p	CCCAGTGTTCAGACTACCTGTTC	ACAGTAGTCTGCACATTGGTTA
miR-199a-2	GCTCCGTCCACCCAGTGTTCAGACTACCTGTTCATTGTCATACTGGTGTACAGTAGTCTGCACATTGGTTAGACTGGGCAT	-36.5	miR-199a-5p	miR-199a-3p	CCCAGTGTTCAGACTACCTGTTC	ACAGTAGTCTGCACATTGGTTA
miR-199a-3	AGCCCGCCTGCCCAGTGTTCAGACTACCTGTTCAGGAAGTAGTGGTTGTACAGTAGTCTGCACATTGGTTAGGCTGGCTGG	-40.3	miR-199a-5p	miR-199a-3p	CCCAGTGTTCAGACTACCTGTTC	ACAGTAGTCTGCACATTGGTTA
miR-199a-4	GCTCCGTCATCCCAGTGTTCAGACTACCTGTTCAGGATCTTACTGGTGTACAGTAGTCTGCACATTGGTTAGACTGTGCAA	-33.2	miR-199a-5p	miR-199a-3p	CCCAGTGTTCAGACTACCTGTTC	ACAGTAGTCTGCACATTGGTTA
miR-19a-1	TTCTCTGCTAGTTTTGCATAGCTGCACTACAAGAATAGACGAGTTGTGCAAATCTATGCAAAACTGATGGTG	-33.53	-	miR-19a-3p	-	TGTGCAAACCCATGCAAAACTG
miR-19a-2	GTTCTCTGCTAGTTTTGCATAGCTGCACTACAAGAATAGACGAGTTGTGCAAATCTATGCAAAAAAAGCAGGAGT	-33.8	-	miR-19a-3p	-	TGTGCAAACCCATGCAAAACTG
miR-19b-1	TCACTTGGTCAGTTTTGCTGGTTTGCATTCCGCTTTAACTGATGTTTGCTGTGCAAATCCATGCAAAACTGACTAGGTTGA	-38.5	miR-19b-5p	miR-19b-3p	AGTTTTGCTGGTTTGCATTCCGC	TGTGCAAATCCATGCAAAACTG
miR-19b-2	AGTCTCTGGTTAGTTTTGCTGGTTTGCTTTCAGCTTTTCACCGTACTGCTGTGCAAATCCATGCAAAACTGATCATAGAAC	-34.02	miR-19b-5p	miR-19b-3p	AGTTTTGCTGGTTTGCATTCCGC	TGTGCAAATCCATGCAAAACTG
miR-19d	TGACCCTGGCCAGCTTTGCAGGGTGGGCAGTCAGCCTGTGTGCGTTAGGAGCAGCTGTGCAAACCCATGCAAAACTGACCATGGCTG	-35.2	-	miR-19d-3p		TGTGCAAACCCATGCAAAACTGA
miR-1a-1	ATTACCTCCTTAGTGTACGTACTTCTTTATGTACCCATATGAACATGATAGCTATGGAATGTAAAGAAGTATGTATTCTTGGTGAG	-28.72	miR-1a-5p	miR-1a-3p	ACGTACTTCTTTATGTACCCA	TGGAATGTAAAGAAGTATGTAT
miR-1a-2	GCCTGCTTGGGGGACATACTTCTTTATATGCCCATATGAACAAGAGCAACTATGGAATGTAAAGAAGTATGTATCCCAGGTTGG	-39.6	miR-1a-5p	miR-1a-3p	ACGTACTTCTTTATGTACCCA	TGGAATGTAAAGAAGTATGTAT
miR-200a	TCTCAGGATCCATCTTACCCGACAGTGCTGGATTGTACGACTGTTGTTCTAACACTGTCTGGTAACGATGTTTTCTGGGT	-35.2	-	miR-200a-3p	-	TAACACTGTCTGGTAACGATG
miR-200b	GGTGATTATCTCCATCTTACGAGGCAGCATTGGATTTCATCACTTTCTCTAATACTGCCTGGTAATGATGATGATAGTCAT	-34.52	miR-200b-5p	miR-200b-3p	TAATACTGCCTGGTAATGATGA	CATCTTACGAGGCAGCATTGGA
miR-202	CTGTTCCTTTTTCCTATGCATATACCTTTTTGAGATGTAGCTTTAAAGAGGCATAGGGCATGGGAAAATGGGGCT	-32.7	miR-202-5p	miR-202-3p	TTCCTATGCATATACCTTTTTG	AAAGAGGCATAGGGCATGGGA
miR-204-1	ACCTGTGGGATTCCCTTTGTCATCCTATGCCTGGAGCTCAAATGAGGCAAGGACAGCAAAGGGGTGCTCAATTGT	-29.6	miR-204-5p	-	TTCCCTTTGTCATCCTATGCCTG	-
miR-204-2	ACCTATGGACTTCCCTTTGTCATCCTATGCCTGGACTCATACAAAAGGGGCTGGGAAGGCAAAGGGACGCCCAGTTGT	-31.66	miR-204-5p	-	TTCCCTTTGTCATCCTATGCCTG	-
miR-205-1	ATGTATTCTATCCTTCATTCCACCGGAGTCTGTGTAAAGTGCCAATCAGATTTCAGTGGTTTGAAGAGTAAAACGCATG	-26.7	miR-205-5p	-	TCCTTCATTCCACCGGAGTCTGT	-
miR-205-2	ATGTATTCTATCCTTCATTCCACCGGAGTCTGTATCCGTACCTAACCAGATTTCAGTGGTGTGAAGTGTAAGACGCATG	-27.24	miR-205-5p	-	TCCTTCATTCCACCGGAGTCTGT	-
miR-206	GCCCCTTGTGAGGACATGCTTCCTTATATCCCCATATTAATACACCACTTATGGAATGTAAGGAAGTGTGTGGTTTCAAGGGG	-38.02	-	miR-206-3p	-	TGGAATGTAAGGAAGTGTGTGG
miR-20a-1	TCAGTAGTATTAAAGTGCTTATAGTGCAGGTAGTTTTTATGAAATTCTACTGCATTGTGAGCACTTAAAGTACTTCTAGAT	-32.1	miR-20a-5p	miR-20a-1-3p	TAAAGTGCTTATAGTGCAGGTAG	ACTGCATTGTGAGCACTTAA
miR-20a-2	CAGTGTGTGATAAAGTGCTTACAGTGCAGGTAGTGATGATAACCTGGCCTACTGCAGTGTGAGCACTTCTTTCCCACGCTGG	-38.8	miR-20a-5p	miR-20a-2-3p	TAAAGTGCTTATAGTGCAGGTAG	TACTGCAGTGTGAGCACTTCT
miR-20a-3	TCAGCAGTACTAAAGTGCTTATAGTGCAGGTAGTGTTTTTCTCCTATCTACTGCAATGTGAGCACTTGAAGTACTTCTAACT	-30.84	miR-20a-5p	miR-20a-3-3p	TAAAGTGCTTATAGTGCAGGTAG	CTGCAATGTGAGCACTTGAAGT
miR-210	AAAGCAGGTAAGCCACTGACTAACGCACATTGTGCCTGCTGCCAGTTCCACTGTGCGTGTGACAGCGGCTAACCTGGTTTTG	-34	miR-210-5p	miR-210-3p	AGCCACTGACTAACGCACATTG	CTGTGCGTGTGACAGCGGCTAA
miR-21-1	GCCTGTCACATAGCTTATCAGACTGGTGTTGGCTGTTAGATTGCAAGGCGACAACAGTCTGTAGGCTGTCTGACATTTC	-36.9	miR-21-5p	miR-21-3p	TAGCTTATCAGACTGGTGTTGG	CGACAACAGTCTGTAGGCTGT
miR-21-2	CCTTGTCAGCTAGCTTATCAGACTGGTGTTGGCTGTTTTATGAGCACGGCAGCAGCAGTCTGTAAGCTGGCTGACGTTGTGGG	-48.9	miR-21-5p	miR-21-3p	TAGCTTATCAGACTGGTGTTGG	CGACAACAGTCTGTAGGCTGT
miR-214-1	GTGGTGTGTCTGCCTGTCTACACTTGCTGTGCAGACCTTCTGCTCCTGTACAGCAGGCACAGACAGGCAGACAGATGGCA	-49.4	miR-214-5p	miR-214-1-3p	TGCCTGTCTACACTTGCTGTGC	TACAGCAGGCACAGACAGGCAG
miR-214-2	TGCAGTGTGTCCGCCTATCTCCACTTGCTGTGCAGAATATCCTCCAACCTGTACAGCAGGCACAGACAGGCAGATAGACAACA	-34.82	miR-214-5p	miR-214-2-3p	TGCCTGTCTACACTTGCTGTGC	ACAGCAGGCACAGACAGGCAG

miR-215	GCACAAGGTGATGACCTATGAATTGACAGCCAGTGATTGTAACATCTGCCTGTCAGTTCTGTAGGCCACTGCTGCG	-29.2	miR-215-5p	-	ATGACCTATGAATTGACAGC	-
miR-217-1	ATGTGGGTGATACTGCATCAGGAACTGATTGGCTGATGCTCAGTAGCCAACAGTACCTGATGCATTACCATCAGCATC	-37.5	miR-217-1-5p	-	TACTGCATCAGGAACTGATTGGC	-
miR-217-2	ATGCTGATGGTAATGCATCAGGTACTGTTGGCTACTGAGCATCAGCCAATCAGTTCCTGATGCAGTATCACCCACATC	-33.3	miR-217-2-5p	-	TAATGCATCAGGTACTGTTGGC	-
miR-2184-1	TTGGGCTCTGAACAGTAAGAGTTTATGTGCTGCTAGAAAGAAGCTCAGCATGTAGAGTCTTACAGTGCAGAGACAAAGACCAGG	-31.3	miR-2184-5p	-	AACAGTAAGAGTTTATGTGCTG	-
miR-2184-2	CTCAGCCCTGAACAGTAAGAGTTTATGTGCTGTTGTCTACCTGTCAGCACATGAGCTTTTACGGTGCAGGGAGGCAGAGG	-38.4	miR-2184-5p	-	AACAGTAAGAGTTTATGTGCTG	-
miR-2187	GATTCTGGCTTTAATTAGTATAGCCTGTTTTAGTGATATCAGCAATTCTTTACAGGCTATGCTAATCTGTGCCAGAATC	-36.4	miR-2187-5p	miR-2187-3p	TTAATTAGTATAGCCTGTTTTAG	TTTACAGGCTATGCTAATCTG
miR-2188	TGGTGTGGGAAAGGTCCAACCTCACATGTCCTGTGCGGCTGAAGGAAGGCTGTGTGAGGTCAGACCTATCCCACACGGC	-42.7	miR-2188-5p	miR-2188-3p	AAGGTCCAACCTCACATGTCCT	GCTGTGTGAGGTCAGACCTAT
miR-218a-1	GGTTGTTCCTTTGTGCTTGATCTAACCATGTGGCTGCCAGGTTCCTATAGTGGAACATGGTTCTGTCAAGCACCATGGAACGGCCA	-44.4	miR-218a-5p	-	TTGTGCTTGATCTAACCATGTG	-
miR-218a-2	CAGCTGTCTCTTGTGCTTGATCTAACCATGTGGCCCCGCCTATTGTGACATCACATGGTTCCGTCAAGCGCCAGGGACCGCCA	-40.8	miR-218a-5p	-	TTGTGCTTGATCTAACCATGTG	-
miR-2201-1	CTTTCACAACTGTCTGTAGAAGCTGGATGCATGACTGTGTGGCTGATTGCACCCGTAGCTATGGAAGAGTGTGGGGC	-22.6	miR-2201-5p	-	TGTCTGTAGAAGCTGGATGCAT	-
miR-2201-10	CTTTCACAACTGTCTGTAGAAGCTGGATGCATGCCTGGGTGGTTGACTGCATGCACCCGTAGCCATGGAAGAGAGTGGGGC	-23.3	miR-2201-5p	-	TGTCTGTAGAAGCTGGATGCATGC	-
miR-2201-11	TCCCATCATGGCACCATGCTGGAGTTTGTTGAGTTCCTGAAAGCGACTGATCCTTTCACAACTGTCTGTAGAAGCTGGATGCATGCCTGGGTGGTT	-25.5	miR-2201-5p	-	TGTCTGTAGAAGCTGGATGCAT	-
miR-2201-12	GGACCACGCCGGGGGGCCATGCTGGAGTTTGTTGAGATCCTGAGAGCGACTGATCCTTTCACAACTGTCTGTAGAAGCTGGATGCATGCCTGGGTGGTT	-32.9	miR-2201-5p	-	TGTCTGTAGAAGCTGGATGCAT	-
miR-2201-13	TCCCATCATGGCACCATGCTGGAGTTTATTGAGATCCTGAGAGTGACTGATCCTGTCACAACTGTCTGTAGAAGCTGGATGCATGCCTGGGTGGTT	-27.1	miR-2201-5p	-	TGTCTGTAGAAGCTGGATGCAT	-
miR-2201-2	CTTTCATAACTGTCTGTAGAAGCTGGATGCATGCATAGGTGGTTGATTGCATGCACCCGTAGCTATGGAAGAGAGTGGGGC	-27.1	miR-2201-5p	-	TGTCTGTAGAAGCTGGATGCAT	-
miR-2201-3	CTTTCACAACTGTCTGTAGAAGCTGGATGCATGCATAGGTGGTTGACTGCATGCACCCGTAGCTATGGAAGAGAGTGGGGC	-27.8	miR-2201-5p	-	TGTCTGTAGAAGCTGGATGCAT	-
miR-2201-4	CTTTCACAACTGTCTGTAGAAGCTGGATGCATGCCTGGGTGGTTGATTGCACCTGTAGCTATGGAAGAGAGTGGGGC	-24.4	miR-2201-5p	-	TGTCTGTAGAAGCTGGATGCAT	-
miR-2201-5	CTTTCACAGCTGTCTGTAGAAGCTGGATGCATGCCTGGGTGGTTGATTGCACCCGTAGCTACGGAAGAGAGTGGGGC	-27	miR-2201-5p	-	TGTCTGTAGAAGCTGGATGCAT	-
miR-2201-6	CTTTTACAACTGTCTGTAGAAGCTGGATGCATGCCTGGGTGGTTGATTGCACCCGTAGCTATGGAAGAGAGTGGGGC	-21.3	miR-2201-5p	-	TGTCTGTAGAAGCTGGATGCAT	-
miR-2201-7	CCAACACAACTGTCTGTAGAAGCTGGATGCATGCCTGGGTGGTTGATTGCACCCGTAGCTATGGAAGAGAGTGGGGC	-23.1	miR-2201-5p	-	TGTCTGTAGAAGCTGGATGCAT	-
miR-2201-8	CTTTCACAGCTGTCTGTAGAAGCTGGATGCATGCCTGGGTGGTTGATTGCACCCATAGCTATGGAAGAGAGTGGGGC	-23.4	miR-2201-5p	-	TGTCTGTAGAAGCTGGATGCAT	-
miR-2201-9	CTTTCACAACTGTCTGTAGAAGCTGGATGCATGCCTGGGTGGTTGATTGCACCCGTAGCTATGGAAGAGAGTGGGGC	-24.4	miR-2201-5p	-	TGTCTGTAGAAGCTGGATGCAT	-
miR-221	TTTGTCCTGAACCTGGCATACAATGTAGATTTCTGTGTGGTTCAATTCTACAGCTACATTGTCTGCTGGGTTTCAGGCTAGCA	-34.6	miR-221-5p	miR-221-3p	ACCTGGCATACAATGTAGATT	AGCTACATTGTCTGCTGGGTTT
miR-22a-1	GACACACAGCAGTTCTTCACTGGCAAGCTTTATGTTCCTGTACACATGCTAAAGCTGCCAGCTGAAGAACTGCTCTGGTCAG	-43	miR-22a-5p	miR-22a-1-3p	AGTTCTTCACTGGCAAGCTTTA	AAGCTGCCAGCTGAAGAACTG
miR-22a-2	GACCTACAGCAGTTCTTCACTGGCAAGCTTTATGTCCTTATATACCAACTAAAGCTGCCAGCTGAAGAACTGTTGTGGTTGGC	-42.2	miR-22a-5p	miR-22a-2-3p	AGTTCTTCACTGGCAAGCTTTA	AAGCTGCCAGCTGAAGAACTGT
miR-222a-1	CCCCAGCGGCTGCTCAGTAGTCAGTGTAGATCCTGTGGGGCTGGCAGCAGCTACATCTGGCTACTGGGTCTCTGCTGGCAG	-43.9	miR-222a-5p	miR-222a-3p	TGCTCAGTAGTCAGTGTAGATC	AGCTACATCTGGCTACTGGGTCT
miR-222a-2	TGCTGTCAGTTGCTCAGTAGGCAGTGTAGATCCTGTGTAGCAATCAGCAGCTACATCTGGCTACTGGGTCTCTGACGGCTC	-42.8	miR-222a-5p	miR-222a-3p	TGCTCAGTAGTCAGTGTAGATC	AGCTACATCTGGCTACTGGGTCT
miR-2291	GCATTGACCTGCAGGGTGGTGATGATGCAGAGCGGAGGAGGTCCGTGGTCTGAAGGAGCGACTGAGGCGAGGAGGAAATGA	-21.8	-	miR-2291-3p	-	TCTGAAGGAGCGACTGAGGCGAG
miR-22b	CGTTACCTCACAGTCGTTCTTCACTGGCTAGCTTTATGTCCCACGCCTCACGCTAAAGCTGCCAGTTGAAGAGCTGTTGTGTGTAAC	-38.1	-	miR-22b-3p	-	AAGCTGCCAGTTGAAGAGCTGT
miR-23a-1	TGTGGTGGGGAGGGTTCCTGGCACCGTGATTTGATGAATAAAGAGATATAAAAATCACATTGCCAGGGATTTCCACTCCTTCAC	-33.41	-	miR-23a-3p	-	ATCACATTGCCAGGGATTTCC
miR-23a-2	TTGGCTGGAGGGATTCCTGGCAGAGTGATTTGGTCATGATGTAATGTAAATCACATTGCCAGGGATTTCCAACCAGCTTC	-39.8	-	miR-23a-3p	-	ATCACATTGCCAGGGATTTCC
miR-23a-3	TGTTGACCAGGGGAATTCCTGGCAGAGTGATTTTTGAGACTACACGACTGAATCACATTGCCAGGGATTTCCAATGGCTGAC	-37.3	-	miR-23a-3p	-	ATCACATTGCCAGGGATTTCC
miR-23b-1	GTGGCTGTGTGGGTCTCTGGCATGATGATTTGGGACAGACAATAAAAATCACATTGCCAGGGATTACTACACTGCTA	-32.24	miR-23b-5p	miR-23b-3p	AGGGTTCCTGGCGTGCTGATTT	ATCACATTGCCAGGGATTACCA
miR-23b-2	TGGGCTGTGAGGGTTCCTGGCGTGCTGATTTGTGACTTATGATAAAATCACATTGCCAGGGATTACCACACAGGCCT	-34.62	miR-23b-5p	miR-23b-3p	AGGGTTCCTGGCGTGCTGATTT	ATCACATTGCCAGGGATTACCA
miR-2476	ATCTAGTGGTTCCCATATGGTCTAGCGGTTAGGATTCCTGGTTTTCGCCTGGGTTTGACTCCTGGTATGGGAACTACTTTAG	-32.4	miR-2476-5p	-	TCCCATATGGTCTAGCGGTTAG	

miR-25	TGGTGTTGAGAGGCGGAGACTTGGGCAATTGCCAGCCATCCCAGAGGGCATTGCACTTGTCTCGGTCTGACAGTGCCGGC	-34.6	miR-25-5p	miR-25-3p	AGGCGGAGACTTGGGCAATTG	CATTGCACTTGTCTCGGTCTGA
miR-26b	CTGGGCCTGATTCAAGTAATCCAGGATAGGTTTGTTCAGACAAGTACAGCCTATTCCGGATGACTTGGTTCAGGAACGC	-34.6	-	miR-26b-3p	-	TTCAAGTAATCCAGGATAGG
miR-26a-1	CTGGGTCTGTTTCAAGTAATCCAGGATAGGCTTGTTAAAGTGGGGGAAGCCTATTCAGGATGACTTGGTTCAGAAACAA	-35.6	miR-26a-5p	-	TTCAAGTAATCCAGGATAGGCT	
miR-26a-2	TGTAACCTGGTTCAAGTAATCCAGGATAGGCTTTCTGTACCTGCTTTGGCCTATGCTTGATTACTTGCGCTTGGGGGCAGC	-35.2	miR-26a-5p	miR-26a-2-3p	TTCAAGTAATCCAGGATAGGCT	CCTATGCTTGATTACTTGCGCT
miR-26a-3	TGTGATTCAGTTCAAGTAATCCAGGATAGGCTGTGTGTATTCTGTTGGCCTATTCATGATTACTTGCACTGGGTGGCAG	-35.5	miR-26a-5p	miR-26a-3-3p	TTCAAGTAATCCAGGATAGGCT	CCTATTCATGATTACTTGCACT
miR-27a	TGGTTGAAGTGCAGGACTTAGCTTGCTCCGTGAACAGTTCAGTGAAAACCTGTGTTCACAGTGGCTAAGTTCCGCTCCTCACAAG	-28.5	-	miR-27a-3p	-	TTCACAGTGGCTAAGTTCCGC
miR-27b	AGCTAGGCACAGAGCTTAGCTGATTGGTGAACAGTGATTGATTTCCTCTTTGTTCACAGTGGCTAAGTTCTGCACCTGAGGAGA	-33.3	miR-27b-5p	miR-27b-3p	AGAGCTTAGCTGATTGGTGAAC	TTCACAGTGGCTAAGTTCTGCA
miR-27c	TGCGCGGCAGCAGGACTTAACCCACGTGTGAACAGTATGTGTTCACCATGTTCACAGTGGTTAAGTTCTGCCGCCACACGG	-40.6	miR-27c-5p	miR-27c-3p	CAGGACTTAACCCACGTGTGAAC	TTCACAGTGGTTAAGTTCTGC
miR-27d-1	CTATGTGTTGTTCACAGTGGCTAAGTTCAGTGCCTGAGGTGAAATAGTTGATTCGATGGAGTGGATTTGTTCCGGTGAGTGGAAAGAAC	-18.5	miR-27d-5p	-	TTCACAGTGGCTAAGTTCAGT	-
miR-27d-2	TTCTAAAGATGCAGAGCTTAGCTCATTAGTGAGCATTGAACCAAACAGGAATTGTTCACAGTGGCTAAGTTCAGTGTCTGACATG	-32.5	miR-27d-5p	-	TTCACAGTGGCTAAGTTCAGT	-
miR-29a	TCCAAGGTTGACCGATTTCTTTTGGTGTTCAGAGTCTGCTTTTGTTTCTAGCACCATTTGAAATCGGTTACAGTGAAGGA	-29.1	miR-29a-5p	miR-29a-3p	ACCGATTTCTTTTGGTGTTCAGA	TAGCACCATTTGAAATCGGTTA
miR-29b-1	TCCCCAGGAAACTGGTTTCAGATGGTGTCTTAGAGTATTAAATCTCATCTAGCACCATTTGAAATCGGTGTTCTTGAGCAG	-33.5	miR-29b-1-5p	miR-29b-1-3p	ACTGGTTTCAGATGGTGTCTTAGA	TAGCACCATTTGAAATCGGTGT
miR-29b-2	CCCTTGGAAGCTGGTTTCAGATGGTGGCATAGAGTATCGTCACCTGTCTAGCACCATTTGAAATCAGTGTTCTTGGGGCG	-35.6	miR-29b-2-5p	miR-29b-2-3p	CTGGTTTCAGATGGTGGCATAGA	TAGCACCATTTGAAATCAGTGT
miR-29c-1	TCTCACCAAGGCTGACCGTTTTCCTCTGGTGTTCAGAGTTTCCATCATGTCTAGCACCATTTGAAATCGGTTACAATGTTGAG	-24.1	-	miR-29c-3p	-	TAGCACCATTTGAAATCGGTTA
miR-29c-2	CCACCAAAAGATAACTGATTTCTTCTGGTGCTTAGAGCTCACTACAGCCTTCTAGCACCATTTGAAATCGGTTATAAAACTGTG	-31.3	-	miR-29c-3p	-	TAGCACCATTTGAAATCGGTTA
miR-29c-3	CTCTCCAAGGTTGACCGATTTCTTTTGGTGTTCAGAGTCTGCTTTTGTTTCTAGCACCATTTGAAATCGGTTACAGTGAAGGA	-30.6	-	miR-29c-3p	-	TAGCACCATTTGAAATCGGTTA
miR-29d-1	CCCCAAGAAGCTGGTTTCACGTGGTGGTTTAGATGTGTGTTCCTGTTGTCTAGCACCATTTGAAATCAGTGTTCTTGGGGAG	-40.2	miR-29d-1-5p	miR-29d-1-3p	CTGGTTTCACGTGGTGGTTTAGA	TAGCACCATTTGAAATCAGTGT
miR-29d-2	TGCACTCAGAAACTGATTTCATTTGGTGGCATAGATGTTTGTAACTAGTCTCTAGCACCATATGAAATCAGTGTTCTGGGACGT	-33.5	-	miR-29d-2-3p	CTGGTTTCACGTGGTGGTTTAGA	TAGCACCATATGAAATCAGTGT
miR-301a-1	AGAGGTCAGCTGCTTTGACGATGTTGCACTACTGTACCATCCGCTAAAGCAGTGCAATAGTATTGTCATAGCATTCGGCCTT	-29.94	-	miR-301a-3p	-	CAGTGCAATAGTATTGTCATAGC
miR-301a-2	GAGGTCAGCTGCTTTGACAATGTTGCACTACTGTACCATCCATTCTAGCAGTGCAATAGTATTGTCATAGCATTTGGCCCC	-31.8	-	miR-301a-3p	-	CAGTGCAATAGTATTGTCATAGC
miR-301b	GGTCAGCTGCTTTGACGATGTTGCACTACTGTACCATCCGCTAAAGCAGTGCAATAGTATTGTCATAGCATTCGGCCT	-27.94	miR-301b-5p	miR-301b-3p	TTTGACGATGTTGCACTACTG	CAGTGCAATAGTATTGTCATAG
miR-301c-1	AGGTCAGCTGCTTTGACAATGTTGCACTACTGTACCATCCATTCTAGCAGTGCAATAGTATTGTCATAGCATTTGGCCC	-31.8	-	miR-301c-3p	-	CAGTGCAATAGTATTGTCATAG
miR-301c-2	GAGGTCAGCTGCTTTGACGATGTTGCACTACTGTACCATCCGCTAAAGCAGTGCAATAGTATTGTCATAGCATTCGGCCT	-28.74	-	miR-301c-3p	-	CAGTGCAATAGTATTGTCATAG
miR-30b	ACTCAGTTCATGTAAACATCCTACACTCAGCTGTAATCATTTGCAGCAGAGGCTGAGAGAAGGTTGTTTACTTGAACTGGCT	-34	miR-30b-5p	miR-30b-3p	TGTAAACATCCTACACTCAGCT	CTGAGAGAAGGTTGTTTACTT
miR-30c-1	TCTCCAGGCATGTAAACATCCTACACTCTCAGCTGTGTTGTCATGGAGCTGGGAGAGGGGTGTTTACACTTCTGGTG	-37.9	miR-30c-1-5p	miR-30c-1-3p	TGTAAACATCCTACACTCTCAGC	CTGGGAGAGGGGTGTTTAC
miR-30c-2	TTCCAGGTAGTGTAAACATCCTACACTCTCGGCATCTGCACCCTGGTGGCCGGGAGTGGGACTGTTTGCACTGGCTGGCT	-45.3	miR-30c-2-5p	miR-30c-2-3p	TGTAAACATCCTACACTCTCGG	CGGGAGTGGGACTGTTTGCA
miR-30d	ATTTTGGGGCTGTAAACATCCCCGACTGGAAGCTGTCTATCTGCAGAGCTTTCAGTCTGATGTTTGCTTCTGCCGACTGC	-32.4	miR-30d-5p	miR-30d-3p	TGTAAACATCCCCGACTGGAAG	CTTTCAGTCTGATGTTTGCTTC
miR-30e-1	TCAGGGCTACTGTAAACATCCTTGACTGGAAGCTGGTGTTCGGTTCTGGGGCTTTCAGTCGGATGTTTGCAGCAGCCAACTGC	-41.1	miR-30e-5p	miR-30e-3p	TGTAAACATCCTTGACTGGAAG	CTTTCAGTCGGATGTTTGCAGC
miR-30e-2	CTGGAGGGGCTGTAAACATCCTTGACTGGAAGCTGGGGTTTGGACGGTGAAGGCTTTCAGTCGGATGTTTGCAGCACCTTATTGC	-42.2	miR-30e-5p	miR-30e-3p	TGTAAACATCCTTGACTGGAAG	CTTTCAGTCGGATGTTTGCAGC
miR-31	CCAGGAAAGAAGGCAAGATGTTGGCATAGCTGTTGAGTTGAAAGCCCTGCTATGCCATCAAATTGCCATTTTTCTCTCCC	-32	miR-31-5p	miR-31-3p	AGGCAAGATGTTGGCATAGCTG	GCTATGCCATCAAATTGCCATT
miR-33b-1	TGGAGCTGTGGTGCATTGTAGTTGCATTGCATGTTATGCTAACACACTGCAATGGATCTGCTTTGCAACACAGAACTTGGCCAT	-31.1	miR-33b-5p	miR-33b-3p	GTGCATTGTAGTTGCATTGC	CAATGTACCTGCAGTGCAACA
miR-33b-2	CAGAGCTGCGGTGCATTGTAGTTGCATTGCATGTGTGTCTCAGCGGAGTGCAATGTACCTGCAGTGCAACACAGAGCTGGG	-40.1	miR-33b-5p	miR-33b-3p	GTGCATTGTAGTTGCATTGC	CAATGTACCTGCAGTGCAACA
miR-33-3	GGTGGCTGTAGTGCATTGTAGTTGCATTGCATGATCCTGAAGTCGAGTGCAATGTGTCTGCAGTGCAGTACAGAGGCCT	-41.8	miR-33-5p	miR-33-3p	GTGCATTGTAGTTGCATTGC	CAATGTACCTGCAGTGCAACA
miR-338-1	TGCTTCCTGGAACAATATCCTGGTGCTGCTCGAGTGCTCCATACAGACTCCAGCATCAGTGATTTTGTTGCAGGCGGCCA	-37	miR-338-5p	miR-338-3p	AACAATATCCTGGTGCTGCTCGA	TCCAGCATCAGTGATTTTGTTG
miR-338-2	TGCCTCCTGGGAACAATATCAGGATGCTGTCTGGGTGTGTAAAGCAGAACTCCAGCATCAGTGATTTTGTTGCCAGGGGGAG	-40.8	miR-338-5p	miR-338-3p	AACAATATCCTGGTGCTGCTCGA	TCCAGCATCAGTGATTTTGTTG

miR-33a-1	TGGAGCTGTGGTGCATTGTAGTTGCATTGCATGTTATGCTAACACACTGCAATGGATCTGCTTTGCAACACAGAACTTGGCCAT	-31.1	-	miR-33a-3p	-	CAATGTACCTGCAGTGCAACA
miR-33a-2	CAGAGCTGCGGTGCATTGTAGTTGCATTGCATGTGTGTCTCAGCGGAGTGCAATGTACCTGCAGTGCAACACAGAGCTGGG	-40.1	-	miR-33a-3p	-	CAATGTACCTGCAGTGCAACA
miR-33a-3	GGTGGCTGTAGTGCATTGTAGTTGCATTGCATGATCCTGAAGTCGAGTGCAATGTGTCTGCAGTGCAGTACAGAGGCCT	-41.8	-	miR-33a-3p	-	CAATGTACCTGCAGTGCAACA
miR-3422	GACTCTAGTCTGGCACTGTGAAGAGACATGAGAGGTGTAGGCTCGAATGTAGGCTCGGACAGGTCTCGCAACTAATGAGTAGACTAGTTGA	-24.4	miR-3422-5p	-	TGGCACTGTGAAGAGACATGAG	-
miR-34a	GGTGTTTCTCTGGCAGTGTCTTAGCTGGTTGTTGTGAGGAGTGAGAACGAAGCAATCAGCAAGTATACTGCCGCAGAAATTCTTC	-34.6	miR-34a-5p	miR-34a-3p	TGGCAGTGTCTTAGCTGGTTGT	AATCAGCAAGTATACTGCCGCA
miR-365	CAAGAAAAGTGAGGGACTTTTAGGGGCAGCTGTGTTTTATTAACCCAGTCATAATGCCCCTAAAAATCCTTATTGCTCTTGC	-34.8	miR-365-5p	miR-365-3p	GAGGGACTTTTAGGGGCAGCT	TAATGCCCCTAAAAATCCTTA
miR-3739-1	AGTACAGTCATTGGTGTAGAGGGAGAAGAGCAGCAAGGAGAGCACACATCTCTGGGGGATGCCTTTGCTGATTGTCCGGATGCTGGATG	-23.4	miR-3739-5p	-	TTGGTGTAGAGGGAGAAGAGCAGC	-
miR-3739-2	GGTCCAGCCATTGGTGTAGAGGGAGAAGAGCAGCAGGGAGAGCACACATCCCTGGGGTGGCGTCTGTGCTGGTTGTGCGGGTGCTGGATG	-33.8	miR-3739-5p	-	TTGGTGTAGAGGGAGAAGAGCAGC	-
miR-3739-3	CTGTACGCTGACTAGTCACCGTCTTGGATGAGAATGATGGCCAGAGGGGTCCCCTGAGGTGCGGTCGTTGGTGTAGAGGGAGAAGAGCAGCATGGAGAGCA	-32.7	miR-3739-5p	-	TTGGTGTAGAGGGAGAAGAGCAGC	-
miR-375	CCGTATTTGCTTCACGTTGAGCCTCACGTACAATACCTGAAGATGAAGTTTTGTTCGTTCGGCTCGCGTTATGCAGGTACG	-30.3	-	miR-375-3p	-	TTTGTTCGTTCGGCTCGCGTTA
miR-429	CCGTTGATAGGCGTCTTACCAGACATGGTTAGATGTAATTATTAGTGTCTAATACTGTCTGGTAATGCCGTCCATTAAATG	-29.3	-	miR-429-3p	-	TAATACTGTCTGGTAATGCCGT
miR-4444-1	GAAGGACGGATCATGCCTGCACGTCGAGCTGTTTCAATGTACTCCTCCAGAGCCTGTCGTTCGGGTTTAGAGATGTGGAAGAGGCGGCTGTTGGGT	-28.32	-	miR-4444-3p	-	TTAGAGATGTGGAAGAGGCG
miR-4444-2	TGCCTGCACATCGAGCTGTTTCAATGTACTCCTCCAGAGCCTGTCATTCGGGTTTAGAGATGTGGAAGAGGCGGCTGTTGGGT	-26.7	-	miR-4444-3p	-	TTAGAGATGTGGAAGAGGCG
miR-4448	ACTGCAGTAACATATTTCAGATAATTCAATGAGTTAACTTTGCATATGGCTCGTTGGTCTAGGGGTATGATTCTCGCTTT	-21.3	-	miR-4448-3p	-	GGCTCGTTGGTCTAGGGGTATGA
miR-454b	GCAGACAAGACCCTATCAATATTGCCTCTGCTTTCACTAGATTTAACAGAGTAGTGCAATATTGCTTATAGGGTCTTGACTCT	-33.32	-	miR-454b-3p	-	TAGTGCAATATTGCTTATAGGG
miR-455-1	GGCGTGAGGGTATGTGCCCTTGGACTACATCGTGGAAGCCAGCACCATGCAGTCCATGGGCATATACACTTGCCTCATG	-40.1	miR-455-5p	miR-455-3p	TATGTGCCCTTGGACTACATCG	TGCAGTCCATGGGCATATACAC
miR-455-2	GATGTGAGGGTATGTGCCCTTGGACTACATCGTGGAAGCCAGCACCATGCAGTCCATGGGCATATACACTTGCCTCAAG	-39.8	miR-455-5p	miR-455-3p	TATGTGCCCTTGGACTACATCG	TGCAGTCCATGGGCATATACAC
miR-458	TTGCAGATCGCAGCGCCATTTTCAGAGCTATAAGTGTGAAAGTTGTCATAGCTCTTTTAAATGGTACTGCTGTATGCAC	-28.99	-	miR-458-3p	-	ATAGCTCTTTTAAATGGTACTG
miR-462	GGTGGGATTGTAACGGAACCCATAATGCAGCTGTGATTCAGAGCTCCAGCTGGTTATGGGGTCCGTTTCCATCCCACA	-43.5	miR-462-5p	-	TAACGGAACCCATAATGCAGCTG	-
miR-466	AGGTTGTGTGTTTATGCTGTGTGTGTGTGTGTGTGTGTGTGTGTGTGTGTGTGTGTGTGTGTGTGTGTGCATTTGCAAGCACCATAATATCACTGTCACCA	-25.2	miR-466-5p	-	TTTATGCTGTGTGTGTGTGTGT	-
miR-48-1	GGTACTTGGTAGTTACTGCTTTCCTTGCGGTCTCTCTATGGGAGGGAGAACTGAGGTAGGACTCAAGATGGCTGACCCAGGTGC	-30.9	-	miR-48-3p	-	TGAGGTAGGACTCAAGATGGCTG
miR-48-2	TTACTTGGACTCGCATCACTACTGTTGTTATGGTTTAGGTCCCCTCACCAGGAGGGTGAGGGAGAACTGAGGTAGGACTCAAGATGGCTGACCCAGGTGC	-34.6	-	miR-48-3p	-	TGAGGTAGGACTCAAGATGGCTG
miR-489	GGTGGCCTGGTGGTCGTATGTATGACGTCATTTACTTCAATGTTTGGAGTGACATCATATGTACGGCTGCTAAACTGCTA	-35.3	miR-489-5p	miR-489-3p	TGGTCGTATGTATGACGTCATT	GTGACATCATATGTACGGCTGC
miR-7-1	CACAGCAGCTTGGAAGACTAGTGATTTTGTTGTTTTGGGGTTAAACTAACAACAAGTCATTGTCTCCCTCACTGCTTTGA	-28	miR-7-5p	miR-7-3p	TGGAAGACTAGTGATTTTGTTG	AACAAATCACAGTCTGCCAAAT
miR-7-2	TAGTGCTAAGTGGAAGACTAGTGATTTTGTTGTGACGCTGATCAAAGAACAACAAGTCCCAGTCTGCCTCTTAGCGCAA	-32.6	miR-7-5p	miR-7-3p	TGGAAGACTAGTGATTTTGTTG	AACAAATCACAGTCTGCCAAAT
miR-7-3	TGCTGCTGTGTGGAAGACTAGTGATTTTGTTGTTTTTAGTTAGATCAACCGACAACAAATCACAGTCTGCCAAATAGCATGGGCT	-31	miR-7-5p	miR-7-3p	TGGAAGACTAGTGATTTTGTTG	AACAAATCACAGTCTGCCAAAT
miR-7-4	TGACACACGGTGGAAGACTAGTGATTTTGTTGTTGTAAATTCACATTCTTGACAACAAGTCACAGTCTACCTCAGTGAGTCAC	-31.4	miR-7-5p	miR-7-3p	TGGAAGACTAGTGATTTTGTTG	AACAAATCACAGTCTGCCAAAT
miR-722-1	GAACAGAATGGAATTTGAAACGTTTTAGCCAAAAATGTTTTCATGGTGAAGGAGTTTTTTGCAGAAACGTTTCAGATTTCGTTTTGTT	-38.7	-	miR-722-3p	-	TTTTGCAGAAACGTTTCAGATT
miR-722-2	GAATAGAATGGAATTTGAAACGTTTTAGCCAAAAATGTTTCCATAGTCAAGGTGTTTTTTGCAGAAACGTTTCAGATTTCGTTTTGTT	-33.8	-	miR-722-3p	-	TTTTGCAGAAACGTTTCAGATT
miR-724-1	CAGACTGGATTTAAAGGGAATTTGCGACTGTTAGTCAAAACATTAGAACAGCCACACCTTCCTTTTAAGATCTTGCCTGC	-20.14	miR-724-5p	-	TTAAAGGGAATTTGCGACTGTT	-
miR-724-2	CAGACTGGGTTTAAAGGGAATTTGCGACTGTTAATCAAACCATTCGAACAGCCACACCTTCCTTTTAAGATCTTGTCTGC	-24.54	miR-724-5p	-	TTAAAGGGAATTTGCGACTGTT	-
miR-881-1	GTTACTTGGATAACTGTGGCAATTCTAGAGCTAATACATGCCAACGAGCGCTGACCTCTGGGGATGCCTGCATTTATCACACCCAAA	-22.6	miR-881-5p	-	TAACTGTGGCAATTCTAGAG	-
miR-881-2	GTTACTTGGATAACTGTGGCAATTCTAGAGCCAACACATGCCAATGAGTGCTGACCTCCAGAGATGCGTGCATTTATCAGACCTAA	-18.7	miR-881-5p	-	TAACTGTGGCAATTCTAGAG	-
miR-881-3	GTTACTTGGATAACTGTGGCAATTCTAGAGCCAACACATGCCAATGAGTGCTGACCTCCAGAGATGCGTGCATTTATCAGACCTAA	-18.7	miR-881-5p	-	TAACTGTGGCAATTCTAGAG	-
miR-92a-1	CCCTTCCAGTGCAGGTGGGGATTAGTAGCAATGCTGTGTACCTGGAGGTATTGCACTTGTCCCGGCCTGTGTAGGAATGA	-41.2	miR-92a-5p	miR-92a-3p	AGGTTGGGAGAGGTTGCAATGC	TATTGCACTTGTCCCGGCCTGT

miR-92a-2	CCCTTTCTGTGCAGGTTGGGAGAGGTTGCAATGCTCTATACATGTATGGTATTGCACTTGTCCCGGCCTGTGGAGGACATG	-38.9	miR-92a-5p	miR-92a-3p	AGGTTGGGAGAGGTTGCAATGC	TATTGCACTTGTCCCGGCCTGT
miR-93	TCTGGGTGCCAAAAGTGCTGTTTGTGCAGGTAGCAGTCATCCAACTACTGCAAAACCAGCACTTCAGGCAGACAGGT	-32.6	miR-93-5p	-	AAAAGTGCTGTTTGTGCAGGTAG	-
miR-99a	ATTTGGCACAAACCCGTAGATCCGATCTTGTGGCCAATCTGACAGCACAAGCTCGCCTCTGTGGGTCTTTGTCACTGT	-25.52	miR-99a-5p	miR-99a-3p	AACCCGTAGATCCGATCTTGTG	CAAGCTCGCCTCTGTGGGTCT
miR-9a-1	TTTTTTTTTCTCTTTGGTTATCTAGCTGTATGAGTTTATGAAATATCATAAAGCTAGAGAACCGAATGTATAAACTAA	-21.5	miR-9a-5p	miR-9a-2-3p	TCTTTGGTTATCTAGCTGTATGA	TAAAGCTAGAGAACCGAATGT
miR-9a-2	TCTGTTTTTGTCTTTGGTTATCTAGCTGTATGAGTGTAAATACGTGTCATAAAGCTAGATAACCGAAAGTAGAAATGA	-31.5	miR-9a-5p	miR-9a-3p	TCTTTGGTTATCTAGCTGTATGA	TAAAGCTAGATAACCGAAA
miR-9a-3	TCAGTTTGTGTCTTTGGTTATCTAGCTGTATGAGTTTTAATTTCATAAAGCTAGAGAACCGAAAGTATGAACTGAC	-35.7	miR-9a-5p	miR-9a-3-3p	TCTTTGGTTATCTAGCTGTATGA	TAAAGCTAGAGAACCGAAAGTA
miR-9a-4	TTGGTTGTTATCTTTGGTTATCTAGCTGTATGAGTGATGTACATTCTTCATAAAGCTAGATAACCGAAAGTAACAAGAA	-35.6	miR-9a-5p	miR-9a-3p	TCTTTGGTTATCTAGCTGTATGA	TAAAGCTAGATAACCGAAA
miR-9a-5	TGGGTTGTTATCTTTGGTTATCTAGCTGTATGAGTGTTCTGCTCGTCATAAAGCTAGATAACCGAAAGTAAAAACTA	-32.8	miR-9a-5p	miR-9a-3p	TCTTTGGTTATCTAGCTGTATGA	TAAAGCTAGATAACCGAAA
miR-9a-6	TTGTCTGTTATCTTTGGTTATCTAGCTGTATGAGTGACGTACATTCTTCATAAAGCTAGATAACCGAAAGTAACAAGAA	-34.1	miR-9a-5p	miR-9a-3p	TCTTTGGTTATCTAGCTGTATGA	TAAAGCTAGATAACCGAAA
miR-9a-7	CCTGTTCCTTTCTTTGGTTATCTAGCTGTATGAGTGTTAATCATCCATCATAAAGCTAGATAACCGAAAGTAGGAATGA	-34	miR-9a-5p	miR-9a-3p	TCTTTGGTTATCTAGCTGTATGA	TAAAGCTAGATAACCGAAA

Supplementary Table S3 | Novel miRNA information

Novel ID	Precursors	MFE	Mature ID		Mature sequences	
			Mature miRNA -5p	Mature miRNA -3p	Mature miRNA -5p	Mature miRNA -3p
Novel-mir-1	CCCCGGGATCAAACCACGGGCGCTGTCCAGTGCTTTTGGTCTCCCCCCTCGTGGTTTGAAGAGTCGT	-22.1	Novel-mir-1-5p	-	AAACCACGGGCGCTGTCCAG	-
Novel-mir-2	GAGCTGACTTAAATGAGTAAGCAGCTGATGTGCCAAAAAGTTGCAAAACATTGCCAGGCAGCTTGTGCTCATAGCAAGCCTCA	-22.6	Novel-mir-2-5p	-	AAATGAGTAAGCAGCTGATGTGCC	-
Novel-mir-3	AACACCCAGAAATCAACAAAATAGTCAAGGACATGGCGAGCCCCCTGGCGTATTGTCATAAGCTCCCTAGAAGCTTGGCTGTGATGTGAGGCTGGTGAA	-32.5	Novel-mir-3-5p	Novel-mir-3-3p	AACAAAATAGTCAAGGACATGGC	TAGAAGCTTGGCTGTGATGTGA
Novel-mir-4	CCTGCAGTAGAACACATTCAGGTTGTCTGCTAGATGACGGTCCTCTACAGCAGGGTGGATGGTTTTCTTTAGC	-20.1	Novel-mir-4-5p	-	AACACATTCAGGTTGTCTGCTAGA	-
Novel-mir-5	GGACACAGCAAACATGTGGAAGAAGGTGTTCTGGTCAGGCAAAATTGAACTTTTTTGGTCTACATGCAAAACGCTATGTGTGG	-19.26	Novel-mir-5-5p	-	AACATGTGGAAGAAGGTGTTCTG	-
Novel-mir-6-1	GCACGAAAGAAATCTCTTCAGGACTGTGCATGCGCAAGGGATAAACCCAATAGCGACAGCTCTTAGAGGGAAAGCCTATACAA	-22.3	Novel-mir-6-5p	-	AATCTCTTCAGGACTGTGCATGCG	-
Novel-mir-6-2	GGCGCAAAAGAATCTCTTCAGGACTGTGCATGCGCGAGGAATACACCCAATAGCAACAGTTCTTAGAGGGCAAAGCCT	-21.94	Novel-mir-6-5p	-	AATCTCTTCAGGACTGTGCATGCG	-
Novel-mir-6-3	GGCGCAAAAGAATCTCTTCAGGACTGTGCATGCGCGAGGGATAAACCCAGTAGCGACAGCTCTTAGAGGGTGAAGCCT	-22.84	Novel-mir-6-5p	-	AATCTCTTCAGGACTGTGCATGCG	-
Novel-mir-6-4	GGCGCGAAAGAATCTCTTCAGGACTGTGCATGCGCGAGGGATAAACCCAATAGCGACAGCTCTTAGAGGGCGAAGCCT	-23.5	Novel-mir-6-5p	-	AATCTCTTCAGGACTGTGCATGCG	-
Novel-mir-6-5	GGCGCGAAAGAATCTCTTCAGGACTGTGCATGCGTGAGGGATAAACCCAATAGCGGCAGCTCTTAGAGGGCTACGCCT	-24.8	Novel-mir-6-5p	-	AATCTCTTCAGGACTGTGCATGCG	-
Novel-mir-6-6	GGCGTGAAAGAATCTCTTCAGGACTGTGCATGCGCGAGGGATAAACCCAATAGCGACAGCTCTTAGAGGGCTACGCCT	-25.3	Novel-mir-6-5p	-	AATCTCTTCAGGACTGTGCATGCG	-
Novel-mir-6-7	GTGCAAAAAAAATCTCTTCAGGACTGTGCATGCGTGAGGGATAAACCCAATAGTGACAGTTTTTAGAGGGCTATGCCA	-21	Novel-mir-6-5p	-	AATCTCTTCAGGACTGTGCATGCG	-
Novel-mir-7	ATGTGAGGACACAAGGATGTGAAGCTGAACGGCGTGCAATAAAATGTCATCTGTGCAATAACTGTGTGTCTTGTCTG	-18.9	Novel-mir-7-5p	-	ACAAGGATGTGAAGCTGAACGGC	-
Novel-mir-8	CCAGGCAAGAACATTTACTTTGGAGCAAGCTGCCTGGAACTTCGGCTCTCTCTGAAGGTAGTGCAGTCATGGTTGA	-21.7	Novel-mir-8-5p	-	ACATTTACTTTGGAGCAAGCTGCC	-
Novel-mir-9	GGGGATGGACACCAGATGGAGCAGAGCAGAGCACCTCCCCTTTTGCCACCTCACTGCGCTCATGGTCTTTATTTGTCTCTTCATCCTTC	-22.71	Novel-mir-9-5p	-	ACCAGATGGAGCAGAGCAGAGC	-
Novel-mir-10	CCCGTGTAGTACCAGATGGGGCAGAGCAGAGCACCTCCCCTTTTGCCACCTCACTGCACGCATGGTCTGTCTTTAGCTCTTCTTCCTGCACAAACTT	-24.2	Novel-mir-10-5p	Novel-mir-10-3p	ACCAGATGGGGCAGAGCAGAGCAC	ATGGTCTGTCTTTAGCTCTTCTTCCT
Novel-mir-11	ACCAAGGCTGACCGTTTTCCTCTGGTGTTCAGAGTTTCCATCATGTCTAGCACCATTTGAAATCGGTTACAATGTTGAGGG	-22	Novel-mir-11-5p	-	ACCGTTTTCCTCTGGTGTTCAGA	-
Novel-mir-12-1	CTTGCTAAAAACCTGTAAAATTCTGTGAGGCAGTAAGTTCCATGTTGAACTTGTTTACAAAGTTTTTCTTCAGGGAATTTTTTTCCAGGTGAAAATTTAT	-22.1	Novel-mir-12-5p	-	ACCTGTAAAATTCTGTGAGGCAG	-
Novel-mir-12-2	CTTGCTAAAAACCTGTAAAATTCTGTGAGGCAGTAAGTTCCATGTTGAACTTGTTTACAAAGTTTTTCTTCAGGGAATTTTTTTCCAGGTGATAATTTAT	-22.1	Novel-mir-12-5p	-	ACCTGTAAAATTCTGTGAGGCAG	-
Novel-mir-13	GCGGTCAGAAACGATAGTGATGGGGGTGCCGTGAATATGAAAAACATGCTGAACCAGGAGCTGTACGGTTTCTAGAGCAG	-19.3	Novel-mir-13-5p	-	ACGATAGTGATGGGGGTGCCGT	-
Novel-mir-14	TACTATGAGAACTGAATTCCATAGATGGTGGCTACTTCAGGTGTCATCTATGGGCTCAGTTCTTTTGGCATGGTACT	-32.8	Novel-mir-14-5p	Novel-mir-14-3p	ACTGAATTCCATAGATGG	ATCTATGGGCTCAGTTCTTTTG
Novel-mir-15	CCAAAAGATAACTGATTTCTTCTGGTGCTTAGAGCTCACTACAGCCTTCTAGCACCATTTGAAATCGGTTATAAAACTGTGGA	-32.2	Novel-mir-15-5p	-	ACTGATTTCTTCTGGTGCTTAGA	-

Novel-mir-16	TATTTTTAAAACTTACTTGAAGACTCATTTGTATGCCTTTGCATTTGATAGGCTATGAGAATGTGTTTGGGCTGTGTTTTGGTTGATAG	-19	Novel-mir-16-5p	-	ACTTACTTGAAGACTCATTTGTAT	-
Novel-mir-17	GAGCCCAGGAACTTGTAGGACTGATACAGTGCTCTTGAGCATATTCAGTTCCAGGTTGTTGCGGACAC	-25	Novel-mir-17-5p	-	ACTTGTAGGACTGATACAGTGC	-
Novel-mir-18	GAAAAAAATTAGAAGAAGGCATATGGACGTTGTTTTACCTAAAACTACGGCCAGATGCCTTCTGCTAAGATTTTTC	-31.7	Novel-mir-18-5p	Novel-mir-18-3p	AGAAGAAGGCATATGGACGTTGT	TACGGCCAGATGCCTTCTGCT
Novel-mir-19	TTTAGATAACAGAGAAAGAAATGTGGAATTGCTGGTTCTTGTCATTTATTAATTTTTCCACGTTAATTTCTTTATAATTTATTTAGGG	-18.4	Novel-mir-19-5p	-	AGAGAAAGAAATGTGGAATTGCTG	-
Novel-mir-20	CAGAACTTCCAGAGGACGGAAAGTAGAGCAGATTTTCTTCCTCTCTTTCTACTTTCTTTCTCTCAGGAACTTGAA	-23	Novel-mir-20-5p	-	AGAGGACGGAAAGTAGAGCAGA	-
Novel-mir-21	GGAGGGGCAAAGAGGAGTGGAGGGAGAAGAGGAGGGGATACTGCCTGAGTTTGTTTTGATTCTCACTTTGCTACTAACGG	-23	Novel-mir-21-5p	Novel-mir-21-3p	AGAGGAGTGGAGGGAGAAGAGGA	TTGTTTTGATTCTCACTTTG
Novel-mir-22	CACAGGCAGCAGATGGATGTTTACTGTGAGGAGGTGCTGGATGTGCTGACCTCCTGCAGGATGGCGCTGCAAGAGCTGCAGACC	-29.4	Novel-mir-22-5p	-	AGATGGATGTTTACTGTGAGGAGG	-
Novel-mir-23	GATCAGTTCATCAAGCTCGGCATCTGTACATGAACTGTCAAGTCCGCGGACAGACAAGTTGTTCTCTGCCATCCTCCTGAACAGTGT	-18.1	Novel-mir-23-5p	Novel-mir-23-3p	AGCTCGGCATCTGTACATGAACTGTCA	ACAAGTTGTTCTCTGCCATCCTCC
Novel-mir-24	GACCCTGGCCAGCTTTGCAGGGTGGGCAGTCAGCCTGTGTGCGTTAGGAGCAGCTGTGCAAACCCATGCAAAACTGACCATGGCTGGAGTTGTCA	-35.3	Novel-mir-24-5p	-	AGCTTTGCAGGGTGGGCAGTCAGC	-
Novel-mir-25	TGGCCAACACAGGAAGAGGATGAAGACAGTGATGTGTTCCTAGAGGATACATGTAGGATCACAGGATTGCGTCACATATTTATTGAGAACGGTAC	-19.5	Novel-mir-25-5p	-	AGGAAGAGGATGAAGACAGTGATG	-
Novel-mir-26-1	TGCTAGGCAGAGGAAGGACAAGTTGGCAGCCATCTGTACTGTGTGGGACAAGTGGGTGGAACGGCTTCCACTTCTCTATAACCCTGGGCCCAATG	-27.2	Novel-mir-26-5p	-	AGGAAGGACAAGTTGGCAGC	-
Novel-mir-26-2	TGCTAGGCAGAGGAAGGACAAGTTGGCAGCCATCTGTACTGTGTGGGACAAGTGGGTGGAACAGCTTCCACTTTTCTATAACCCTGGGC	-27.9	Novel-mir-26-5p	-	AGGAAGGACAAGTTGGCAGC	-
Novel-mir-27	GTTGAAGTGCAGGACTTAGCTTGCTCCGTGAACAGTTCAGTGAAAACCTGTGTTCACAGTGGCTAAGTTCCGCTCCTCACA	-28.5	Novel-mir-27-5p	-	AGGACTTAGCTTGCTCCGTGAAC	-
Novel-mir-28	CCCCGGCACCAGGCTTGAGAGCATAGCGGCCGTTCGGGAGGACGACCTCTATGCCAGGCTTGAGAGCATAGTGGCCGGTCAGGA	-37.6	Novel-mir-28-5p	-	AGGCTTGAGAGCATAGCGGC	-
Novel-mir-29	TCAGTAGCAGAGGGGTTGTACATTAATGAGCACCATCGACAAATGCTCATATGCTCCCACCCCAACTGCTGTGAA	-25.6	Novel-mir-29-5p	-	AGGGGTTGTACATTAATGAGC	-
Novel-mir-30-1	AAGAATACACAGGTAAATGCTCTGCAGAGAGGGCAGCAGAACCCTGGTGATCTTCTCTACAGTCTTTACCACTCTCTGCAGGCGCTTGCGGTACATAGCA	-31.5	Novel-mir-30-5p	-	AGGTAAATGCTCTGCAGAGAGGGC	-
Novel-mir-30-2	CACCCTGCGGAGGTAAATGCTCTGCAGAGAGGGCAGTGGAGTCCTTCTGATCTTCTCCACAGTCTTTACCACTCTCTGCAGGCGCTTGTGGTCCATAGCAGTAGT	-37.73	Novel-mir-30-5p	Novel-mir-30-3p	AGGTAAATGCTCTGCAGAGAGGGC	CACTCTCTGCAGGCGCTTGTGGTCCA
Novel-mir-31	CTTCCAGTGCAGGTGGGGATTAGTAGCAATGCTGTGTACCTGGAGGTATTGCACTTGTCCCGGCCTGTGTAGGAAT	-38.8	Novel-mir-31-5p	-	AGGTGGGGATTAGTAGCAATGC	-
Novel-mir-32	CACGGAGTCGAGTATAAAGAGGAGCGAGCTAAGCACTCACTGGAGGCTAACTGTTATATGCATTGTTCTTCTGTGACCAGCAATAAAGG	-19.72	Novel-mir-32-5p	Novel-mir-32-3p	AGTATAAAGAGGAGCGAGCTAAGC	TATGCATTGTTCTTCTGTGACCA
Novel-mir-33-1	TTTAATCCGGATAAAATTGATCCGGATTCAGTAATCCTTTTTTTGCAATCCGTGATCACGTAATCCATCTTACT	-18.7	Novel-mir-33-5p	-	ATAAAATTGATCCGGATTCAGTAA	-
Novel-mir-33-2	TTTAATCCGGATAAAATTGATCCGGATTCAGTAATCCTTTTTTTGCGATCCGTGATCACGTAATCCAGCTTACT	-19.1	Novel-mir-33-5p	-	ATAAAATTGATCCGGATTCAGTAA	-
Novel-mir-33-3	TTTAATCCGGATAAAATTGATCCGGATTCAGTAATCCTTTTTTTGCGATCCGTGATCACGTAATCCATCTTACT	-19.1	Novel-mir-33-5p	-	ATAAAATTGATCCGGATTCAGTAA	-
Novel-mir-33-4	TTTAATCCGGATAAAATTGATCCGGATTCAGTAATCTTTTTTTTGCGATCCGTGATCACGTAATCCAGCTTACT	-19.1	Novel-mir-33-5p	-	ATAAAATTGATCCGGATTCAGTAA	-
Novel-mir-34	TCTCCTTCGCATAGAGTTGAATAGGTTGTCGATTGTGGCCTGTGGAATGTTGGTCCACTCCTCTTCAATGGCTGTGCGAACTTGCTGG	-28.5	Novel-mir-34-5p	Novel-mir-34-3p	ATAGAGTTGAATAGGTTGTCGATT	TCCTCTTCAATGGCTGTGCG
Novel-mir-35	TCTTCCACACATCATGGACAGGTGCTTTCCTTTGCGTCAGTCACAGGAGCCTGACAGAAGGGAAGTGACGACAGTCCAATGTATGAATTT	-29.8	Novel-mir-35-5p	-	ATCATGGACAGGTGCTTTCCTTT	-
Novel-mir-36	TCAGGAGCTGATCTGAACAAGTGGATTTCGGAGGGAAACCGATGTTGGAAGTGTGCTTGCTCTCATTCTCCCCA	-22.3	Novel-mir-36-5p	-	ATCTGAACAAGTGGATTTCGGAG	-
Novel-mir-37-1	AGTTTTGTCCATGAAGTGATGAGGTTGGGCTGCGAGAGAAAGCGACACTCCTGAGCTGGTCAAGGTGGTGATCCG	-18.3	Novel-mir-37-5p	-	ATGAAGTGATGAGGTTGGGCTGCG	-
Novel-mir-37-2	GTTATTGTCCATGAAGTGATGAGGTTGGGCTGCGAGGAAAAGTGACACTCCTGAGCTGGTCAAGGTGGTGATCCGGTAGACTGAAGAAGTAACA	-20.4	Novel-mir-37-5p	-	ATGAAGTGATGAGGTTGGGCTGCG	-

Novel-mir-38	CGAGTGAGGAATGATCAAACGAGGGAAATGGGTTTTAACCACTGGGTACATCTCAAGTCTCTTGTATGATCATTCCTTGCTCTTT	-43.7	Novel-mir-38-5p	Novel-mir-38-3p	ATGATCAAACGAGGGAAATGGG	CAAGTCTCTTGTATGATCATTC
Novel-mir-39	ACATCCATAAATGCTGCTTAAAGCTGTACTGTGACTATCGCACAGTGGAAATGTGTACTGTGGTCAGATGAATCAGTGTTTGAGGTCGTTATGGAAGA	-27.2	Novel-mir-39-5p	-	ATGCTGCTTAAAGCTGTACTGT	-
Novel-mir-40	ATTAATCCTGATGTTGCTTTGGCCACTGAGGGGTCGGTTCTGATTTGCAATGAATTTCAGAGATACTTCATTGATAAAGTTTGGGCTATTAGACAAAAGATTC	-20.7	Novel-mir-40-5p	Novel-mir-40-3p	ATGTTGCTTTGGCCACTGAGGGGTC	TTGATAAAGTTTGGGCTATTAGA
Novel-mir-41	CCGCTTTGCCTAGTATTTCTCCAATAAGTTCAGTTCTCTTCACAAGGTTACCCTCTTTCAGGGAGATTAATGGTCTTATCCTGGGAATGGCTGGTCTGCAC	-24.7	Novel-mir-41-5p	Novel-mir-41-3p	ATTTCTCCAATAAGTTCAGTTCT	TAATGGTCTTATCCTGGGAATGGC
Novel-mir-42-1	TTGCAGACCAATTTGATGCAGCGTGCGGCGTATGGTCTGAGCACTGACAGGCTGACCCCCCACCCCTTCAACCTCTGCAGC	-21.6	Novel-mir-42-5p	-	ATTTGATGCAGCGTGCGGCGTATG	-
Novel-mir-42-2	TTGCAGACCAATTTGATGCAGCGTGCGGCGTATGGTCTGAGCACTGACAGGCTGACCCCCCACCTCTTCAACCTCTGCAGC	-23.9	Novel-mir-42-5p	-	ATTTGATGCAGCGTGCGGCGTATG	-
Novel-mir-43	GGCCATGGGCCAAACACGTCGAGACCCACATAGGTGACAGGAGGGTCTGCCTGTGTCCTGTCTTCTGGTAGGTCAGACATGATTTGGTCTTGCA	-41.8	Novel-mir-43-5p	-	CAAACACGTCGAGACCCACATAGG	-
Novel-mir-44	TGTTTGTTGGCAAATGGACGGTGTGCCTTGCCTTCGTGTGTCAAAGTGTCATGCACAGAAGTTCAGGTAATCAGCCATTTAGTGAGGGAATAT	-27.4	Novel-mir-44-5p	-	CAAATGGACGGTGTGCCTTG	-
Novel-mir-45	TAGGTCTTAACAAGATCTGGAGAGTTGGGAGTACTTATTGACTTGTTTGCCCCAGACTTGGAGAAAACTGTAAGACAGC	-19.7	Novel-mir-45-5p	-	CAAGATCTGGAGAGTTGGGAG	-
Novel-mir-46	CCTTGTGCGGCAATTTACATACTGGGTGAAGTTTGGGGAGAGAAGCATGGTTAAAGTCCCCCCGAGGTAAGAATTAAAGCCTGTGGATGTTGTGACTACAT	-18.96	Novel-mir-46-5p	Novel-mir-46-3p	CAATTTACATACTGGGTGAAGT	TAAGAATTAAAGCCTGTGGATGTT
Novel-mir-47	TAATGCTCCAGCCACCTTTGAGAGATTGATGGAGCGGGTCCTCAAGGACATTCCCAGAACCCGCTGCGTAGTTTACCTGGACGACTTGCTGGTGCACGC	-33	Novel-mir-47-5p	Novel-mir-47-3p	CACCTTTGAGAGATTGATGGAG	GCGTAGTTTACCTGGACGACTTGC
Novel-mir-48	GCAGCGTGTCCACTGCCTGGAATCTTTGCTGCACTTTCGTAGCCCGGTTAGCGCAGTTGGTAGAGCATG	-20.7	Novel-mir-48-5p	-	CACTGCCTGGAATCTTTGCTGCA	-
Novel-mir-49	TCCATAAACACAGATATTAAGGTGGAAGTGTTGTACTGAATTGCATACAATTGTACAGGTGTACCTACTAAAGTGTCCGGTTAGTGTA	-18.4	Novel-mir-49-5p	-	CAGATATTAAGGTGGAAGTGTTGT	-
Novel-mir-50	CCATCTACAGCAGGACTGGTTTTGGCCTCTGCACCCGTCTGTGTCTGCTGGCTGGGAGCAGCTGCTTTCTTTAT	-23.8	Novel-mir-50-5p	-	CAGGACTGGTTTTGGCCTCTGCA	-
Novel-mir-51	GGGCTCAAAGCAGGTGTAGAAGGTGTTGAGGTCATCCGGCAGGCTGTCTGTGGGGCTGATGATGCTGGTGGTGGTCCT	-22.4	Novel-mir-51-5p	Novel-mir-51-3p	CAGGTGTAGAAGGTGTTGAGGTCATC	TGTCTGTGGGGCTGATGATGCTGG
Novel-mir-52	CGTGTGTAAACAGTTGGTACATCATAGATGGAGAAGCTCGTGGCCGTTGCTCCAACACGACCTACTGCTCTGTGAATTAACTCATGGCGATGG	-24.22	Novel-mir-52-5p	-	CAGTTGGTACATCATAGATGGAGA	-
Novel-mir-53	TACATTGCCGCATTTCTCAAGGGCAAGCAGGAGGCGAGTGGGTATCCAGCAGGTGTCACAGATGAGGCAGGCAGACTG	-21.1	Novel-mir-53-5p	-	CATTTCTCAAGGGCAAGCAGGAGG	-
Novel-mir-54	CTGAGCCAAACCAGACAGTGATGGACGAGCAGAGAACAGACTCCCTGATTCCTCTGTAGAACTGTATCGGCAGCTCCTGGA	-21.4	Novel-mir-54-5p	Novel-mir-54-3p	CCAGACAGTGATGGACGAGCAGAGAA	TTCCTCTGTAGAACTGTATCGGC
Novel-mir-55	ATAAACTGGGGGCCCCTGTCTGACACCACATCCCTGGGGAGACCACGCAACCGGAACACATGTAGGATGAGGAGTTGGGCTGTGTCCTTAGCTGAG	-33.2	Novel-mir-55-5p	Novel-mir-55-3p	CCCCTGTCTGACACCACATCCCTGG	TAGGATGAGGAGTTGGGCTGTGTC
Novel-mir-56	GCTGCCGTAACCGGTCTGAAGTGAATCTCCCTCCATTGAGCTGTGTCCGGCTTCTCCTTTAAGGTATGTTCAACAGGCTGATTGTAGA	-22.42	Novel-mir-56-5p	-	CCGGTCTGAAGTGAATCTCCCT	-
Novel-mir-57	CCAGGTGTGGGATGCCTAGTTTAACTGTTGGCCTGGAGTGGCCAAGCCACCGTTGGGATCGACAGGGCGCTGGCTAGAAATCTGA	-32.2	Novel-mir-57-5p	Novel-mir-57-3p	CCTAGTTTAACTGTTGGCCTGGA	TGGGATCGACAGGGCGCTGGCT
Novel-mir-58-1	ACCCTTCAAACCTGAGAGACCTGGAGCAGCTGGCAAAAGAAGAGTGGTCCAGAATTCCAGTAGAGAGGTGGA	-20.6	Novel-mir-58-5p	-	CCTGAGAGACCTGGAGCAGCTGGC	-
Novel-mir-58-2	AGCCTTCAAACCTGAGAGACCTGGAGCAGCTGGCAAAAGAAGACTGGTCCAAGATTCCAGTAAAGAGGTG	-20.3	Novel-mir-58-5p	-	CCTGAGAGACCTGGAGCAGCTGGC	-
Novel-mir-59	ACTCCTCCTCCCTGGAGAACTGACGAGAGAGGGCATCCACCTTGCCGTTAAGGCGACCTGGGCGAAAAGAGAGAGAGA	-22.5	Novel-mir-59-5p	-	CCTGGAGAACTGACGAGAGAGGGC	-
Novel-mir-60	TGGTTCCTAATAAGACGAAGGTTTGGCCTGGTGAATGTCCGCTCGCTGAAGAACAAAACCTTTATTTTGAGGGACT	-23.5	Novel-mir-60-5p	Novel-mir-60-3p	CGAAGGTTTGGCCTGGTGAAT	TCGCTGAAGAACAAAACCTTTATT
Novel-mir-61-1	AGTGAGAAGGGGGGTCGCAGAGACGTCAAGGGGGACAGAGTCCAGCTGTTGAGAAGTAGCTAGTGGCTGAGCTGATA	-18.8	Novel-mir-61-5p	Novel-mir-61-3p	CGCAGAGACGTCAAGGGGGA	GCTGTTGAGAAGTAGCTAGTGGC
Novel-mir-61-2	GAGAAGGGGGGTCGCAGAGACGTCAAGGGGGACAGAGTCCAGCTGTTGAGAAGTAGCTAGTGGCTGAGCTGAAA	-18.5	Novel-mir-61-5p	Novel-mir-61-3p	CGCAGAGACGTCAAGGGGGA	GCTGTTGAGAAGTAGCTAGTGGC
Novel-mir-62	AGGTGGGATACGGGACCGGGACGCTGTCTGAGAGGTCTCTATGAATCTCACAGTGAACCGGTCTCTTTTTCAGCCCCTCACTCCTCACCTG	-37.5	Novel-mir-62-5p	-	CGGGACCGGGACGCTGTCTGAG	-

Novel-mir-63	CACCCTGCGGCGGTAAATACTCTGCAGAGATGGCAGTAGAGTCCTGGTGATCTTCTCCGCAGTCTTTAACACTCTGCAGGCTTTTGCGGTCCATAGCAGTA	-33.8	Novel-mir-63-5p	-	CGGTAAATACTCTGCAGAGATGGC	-
Novel-mir-64-1	CACCCTGCAGCGGTAAATGCTCTGCAGAGAGGGCAGTGGAGTCCTGGTGATCTTCTCAACAGTTTTACCACTCTCTGCAGGCACTTGCGGTCCATAGCAGT	-38.9	Novel-mir-64-5p	-	CGGTAAATGCTCTGCAGAGAGGGC	-
Novel-mir-64-2	CACCCTGCAGCGGTAAATGCTCTGCAGAGAGGGCAGTGGGGTCCTGGCGATCCTCTCCGCAGTCTTTACCACTCTCTGCAGGTGC	-37.1	Novel-mir-64-5p	-	CGGTAAATGCTCTGCAGAGAGGGC	-
Novel-mir-65	ACCAACTGCGCTAACCGGGCTACGAAAGTGCAGCAAAGATTCCAGGCAGTGGACACGCTGCATGGTCGTACCAACTTTAGCATTGAACAT	-29.2	Novel-mir-65-5p	-	CTAACCGGGCTACGAAAGTGCAGC	-
Novel-mir-66	ACAAAGCAGCCCTAGTCTCATCCCAGACGGCTTGACAGCGAGTGATGTGGGCTTAGACAGAGGGGACAGCAATAT	-23.6	Novel-mir-66-5p	Novel-mir-66-3p	CTAGTCTCATCCCAGACGGCT	TGATGTGGGCTTAGACAGAGGGG
Novel-mir-67	TAGGTGCAGCCTCAGGAAAATAAAAAGGGGAAAACATTTGGTCTTTTAACTGGTGGTAAATGTCCTGGGCTTCTGTGGGGCTGAGGTAGG	-20.8	Novel-mir-67-5p	Novel-mir-67-3p	CTCAGGAAAATAAAAAGGGGAAAAC	TAAATGTCCTGGGCTTCTGTGGGG
Novel-mir-68	TGTTTCATTCCTCCAATTCTCTTTTTCCTACTGTCTTTGTATGCCCTGCCACCCCAGAGAGCACAGGATCCAAGAGGGAAAGGTGGTGTTCCAACT	-20	Novel-mir-68-5p	-	CTCCAATTCTCTTTTTCCTACTG	-
Novel-mir-69	ACAGGCAGGGCTCCACTGGGTGATGGTGCCAGTAGTTCAATCAATGGTGGGGTTGTGTTGGACCAGCCAGGAGTGGCCCAGAACCAG	-27.8	Novel-mir-69-5p	Novel-mir-69-3p	CTCCACTGGGTGATGGTGCCAGTAGT	TGTGTTGGACCAGCCAGGAGTGGC
Novel-mir-70	TGATTGTTCCCTCCTTCTTTGCTTGCCTTTTTGCTTCGTGTGTTGTCTGCTGTCATAGATAGGCCAGTACAGCAAGGAGGGTGTAGTGG	-29.2	Novel-mir-70-5p	-	CTCCTTCTTTGCTTGCCTTTTT	-
Novel-mir-71	CTCGTCAGTGCTCTTAGGGCTGTAGCCTGTGCTGCTTGTGTGGGCGTGCGTGCGTATGTGTGTTGTGTA	-19.3	Novel-mir-71-5p	-	CTCTTAGGGCTGTAGCCTGTGC	-
Novel-mir-72	CAGTGACCTGCTGAAGATAAGTGTGAAGATGGGGGCCAGCTGATCAGCATACACTTTCAGACAGGAGGTGCTGC	-24.3	Novel-mir-72-5p	-	CTGAAGATAAGTGTGAAGATGGGG	-
Novel-mir-73	TGATTCCATACTGGACATAATCCTGGACTGCACCAACATAGAAGGTCGGCGGGTTTATGGAGAGTGGTGGAAGAT	-21.7	Novel-mir-73-5p	-	CTGGACATAATCCTGGACTGCACC	-
Novel-mir-74	AAAAAATCAGCTGGACTAGATTTTTAGGTTGAAGCCTCTTGGATGAGAGGTGAAACGTCTTCGACCTAGAAATCTAGTCCAGTTGATGTTTTC	-52.7	Novel-mir-74-5p	-	CTGGACTAGATTTTTAGGTTGAAG	-
Novel-mir-75	ACAGTGTCTACTGTAATGCACTGTATGCTGGAGTTGGTCAATTGGGGCTTGCCCGCCTCCAGAGGGTCCAGAATGCTGCCGCTCGGTTATTGG	-26.5	Novel-mir-75-5p	-	CTGTAATGCACTGTATGCTGGAGT	-
Novel-mir-76-1	ACTGTCTGCTGAAGTCTGTACTTGTCCTGTTTGGTTGCTGAGCCAAACCAGACAGTGATAGAGGTGCAGAGAACAGACTCG	-32.1	Novel-mir-76-5p	Novel-mir-76-3p	CTGTACTTGTCCTGTTTGGT	CAGACAGTGATAGAGGTGCAGAG
Novel-mir-76-2	GTCCACTGAAATCTGTACTTGTCCTGTTTGGTTGCTGAGCCAAACCAGACAGTGATAGAGGTGCAGAGAACAGACTCA	-30.9	Novel-mir-76-5p	Novel-mir-76-3p	CTGTACTTGTCCTGTTTGGT	CAGACAGTGATAGAGGTGCAGAG
Novel-mir-76-3	GTCCACTGAAGTCTGTACTTGTCCTGTTTGGTTGCTGAGCCAAACCAGACAGTGATAGAGGTGCAGAGAACAGACTCG	-29.8	Novel-mir-76-5p	Novel-mir-76-3p	CTGTACTTGTCCTGTTTGGT	CAGACAGTGATAGAGGTGCAGAG
Novel-mir-77	GCGGGACCACCTGTCAAATTAATTCTGAAGAGAAGTGGTATCTTTATTGTGTGTGATAATTATAAGTTGAGTCAAGGTTGGGCTTTTTGAGCAGTGGTCTAA	-22.3	Novel-mir-77-5p	Novel-mir-77-3p	CTGTCAAATTAATTCTGAAGAG	TGAGTCAAGGTTGGGCTTTTTGAGC
Novel-mir-78	GACTGAGCTGCTGTCGGCATCACAATGGGGGAACCTCTCGTATCCTGTGCTGTGAAAGGCTGACTTGCCTTTGTAGTG	-25.3	Novel-mir-78-5p	-	CTGTCGGCATCACAATGGGGGAAC	-
Novel-mir-79	CTGCTTCAGGCTGTGTCATGTTTGTGAATGCGTGTGCTTGTGTACGTCTGTATTTTGTGCACGCTCTGTTTATACAAGTGCATATCTGTTAGTTGA	-27.9	Novel-mir-79-5p	-	CTGTGTCATGTTTGTGAATGCGTG	-
Novel-mir-80-1	CGAGTAGCAGCTGTTAATCATGGCCTGCACGCACATTACCTTATTACCTTATGCAGGAGTGTGGCTTGTGCTGTAGTTAGCAGCAATGACATT	-28.6	Novel-mir-80-5p	-	CTGTTAATCATGGCCTGCACGCA	-
Novel-mir-80-2	CGAGTAGCAGCTGTTAATCATGGCCTGCACGCACATTACCTTTTTACCTTATGCAGGAGTGTGGCTTGTGCTGTAGTTAGCAGCAATGACCTTGT	-27.72	Novel-mir-80-5p	-	CTGTTAATCATGGCCTGCACGCA	-
Novel-mir-80-3	GGAGTAGCAGCTGTTAATCATGGCCTGCACGCACATTACCTCGTTACTTTATGCAGGAGTGTGGCTTGTGCTGTAGTTAGCAGCAATGATGTTATGGG	-32.1	Novel-mir-80-5p	-	CTGTTAATCATGGCCTGCACGCA	-
Novel-mir-81	GCCGTATAGCCTTGGATTGTAGCGCCCCCAGGCGGTCTTATGTCTCTACTGCGGGTCGTTACTGGTTCTTTCTACAACGAC	-22.3	Novel-mir-81-5p	-	CTTGGATTGTAGCGCCCCCAGG	-
Novel-mir-82	AAGCGAGTGCCTTTGAGTCATGCTGGAGAGGAGCTATGTGCTGCCCAAAAGGAATTTAAGGGCTGTAGGCCA	-18.6	Novel-mir-82-5p	-	CTTTGAGTCATGCTGGAGAGGAGC	-
Novel-mir-83	ACATCTGAGACTTTTATTGGGCTTTCGGACAGCGGAGGGAGAGGACTTGTCTTACCCCCTACATAGGACAAAGGAAACGAGGGGAAAAGATCAGAGTGG	-24.3	Novel-mir-83-5p	Novel-mir-83-3p	CTTTTATTGGGCTTTCGGACAGCG	TAGGACAAAGGAAACGAGGGGAAAAGAT
Novel-mir-84-1	ACAAGACAAAGAAGATGATAGTGGACATGAGGAAGGAGAGGAGATCTCATCAGCCACTGTTCATCTGGGAGCTCGAAA	-22.9	Novel-mir-84-5p	-	GAAGATGATAGTGGACATGAGG	-
Novel-mir-84-2	ACAAGACAAGGAAGATGATAGTGGACATGAGGAAGGACAGGAGATCTCATCAGCCGCTGTTCATCCGGGGGCTCGA	-18.2	Novel-mir-84-5p	-	GAAGATGATAGTGGACATGAGG	-

Novel-mir-84-3	ACAAGACGACGAAGATGATAGTGGACATGAGGAAGGACAGGAGATCTCATCAGCCACTGTTCATCCGGGGGCTCGA	-19.4	Novel-mir-84-5p	-	GAAGATGATAGTGGACATGAGG	-
Novel-mir-85	TGTGTTCATTGAATAAATCAGCACTGTTATGAAGTTACTTGGACTGCCTCGTCTCTCAGAGTTAGTGTAGCAGAGTTGTAATTCCATTTGCCAT	-19	Novel-mir-85-5p	-	GAATAAATCAGCACTGTTATGAAG	-
Novel-mir-86	GTGCCCACCTGACGACCCTGTAGACTTGCCTTCACTCGTATACCCACACAGAACATGTGTTGGTGGTAATGTAGTTACTGACTCACTGGGTGTGTTA	-21.2	Novel-mir-86-5p	-	GACGACCCTGTAGACTTGCCTT	-
Novel-mir-87	TCGGGAGGAAGGAGATTGCTTAAAGCAATTTTCAGACGATTGTATTCTCTGGGATTTGGATAACTCATATCAGGAATTGTGGGTGTTGGCCCTCTGTAG	-26.1	Novel-mir-87-5p	Novel-mir-87-3p	GACGATTGTATTCTCTGGGATT	TATCAGGAATTGTGGGTGTTGG
Novel-mir-88-1	AAATATCAGAGACTGATTTAATGGGTACTGTGGACAGCTGAATAATTGCCCTGCCCATTGTGTGCAGGCTGAGCTCT	-20.8	Novel-mir-88-5p	-	GACTGATTTAATGGGTACTGTGGA	-
Novel-mir-88-2	AAATATCAGAGACTGATTTAATGGGTACTGTGGACAGCTGAATGATTGCCCTGCCCATTGTGTGCAGGTTGGTTTTTT	-20.9	Novel-mir-88-5p	-	GACTGATTTAATGGGTACTGTGGA	-
Novel-mir-89	TGTGCTCCTGGACTTGGTCAAAGCTCCTCAGCAGATTAAAGTGACTCTGAGGCGTTTAGAACAAGTTCAGGGTCCTGGA	-31.7	Novel-mir-89-5p	Novel-mir-89-3p	GACTTGGTCAAAGCTCCTCAG	TGAGGCGTTTAGAACAAGTTCA
Novel-mir-90	AGACTGAAAAGAGACTGAGAACTTTGCCCAGGGCCGGGCACGGAGACTACGGCAAAGTTTCGCCGGCGGAGC	-20.3	Novel-mir-90-5p	-	GAGACTGAGAACTTTGCCCAGGGC	-
Novel-mir-91	CTTCAGAAAGGAGTACTTTATCGTCAGCATGGAGCAATTAAGCAGCTAGTTGTTCCTCAGTCTGTTCGGGATGTTGTGCTTACTTTGGGTCACTC	-27.1	Novel-mir-91-5p	-	GAGTACTTTATCGTCAGCATGGAG	-
Novel-mir-92	AAATGGCTCAGATGTTGAGTATCAAACTGTATGTTATATATGTCATGTATATAGTTTGATACACAGCACAACAAGCCGTTTT	-30.14	Novel-mir-92-5p	Novel-mir-92-3p	GATGTTGAGTATCAAACTGTAT	TAGTTTGATACACAGCACAAC
Novel-mir-93-1	TGATAGGGCCGCAGAAATGACTGGACCACTGGAGGAGCCGCCCGGGGTCGGAGGAGAGGTTTTGGCACTACGTAT	-19.7	Novel-mir-93-5p	Novel-mir-93-3p	GCAGAAATGACTGGACCACTGGAG	CCCGGGGTCGGAGGAGAGGTTTTGG
Novel-mir-93-2	TGATAGGGCCGCAGAAATGACTGGACCACTGGAGGAGCCGCCCGGGGTCGGAGGAGAGGTTTTGGCACTGCGTAT	-19.5	Novel-mir-93-5p	Novel-mir-93-3p	GCAGAAATGACTGGACCACTGGAG	CCCGGGGTCGGAGGAGAGGTTTTGG
Novel-mir-93-3	TGATCGGGCCGCAGAAATGACTGGACCACTGGAGGAGCCGCCCGGGGTCGGAGGAGAGGTTTTGGCACTGCGTAT	-19.9	Novel-mir-93-5p	Novel-mir-93-3p	GCAGAAATGACTGGACCACTGGAG	CCCGGGGTCGGAGGAGAGGTTTTGG
Novel-mir-93-4	TGATCGGGCCGCAGAAATGACTGGACCACTGGAGGAGTCGCCCAGGGTCGGAGGAAAGGTTTTGGCACTGCGTATAGAGCAG	-21.9	Novel-mir-93-5p	-	GCAGAAATGACTGGACCACTGGAG	-
Novel-mir-93-5	TGATTGGGCCGCAGAAATGACTGGACCACTGGAGGAGCCGCCCGGGGTCGGAGGAGAGGTTTTGGCACTGCGTATAGAGCAG	-22	Novel-mir-93-5p	Novel-mir-93-3p	GCAGAAATGACTGGACCACTGGAG	CCCGGGGTCGGAGGAGAGGTTTTGG
Novel-mir-93-6	TGATCGGGCCGCAGAAATGACTGGACCACTGGAGGAGCCGCCCGGGGTCAGAGGAGAGGTTTTGGCACTGCGTATAGAGCAG	-23.1	Novel-mir-93-5p	-	GCAGAAATGACTGGACCACTGGAG	-
Novel-mir-94	CCTATTTATCGCATACCAGGGATCATGGACCGGTTTGCGTATGTCAAAATACTTGAAGAGGTCATGTTGCCTTATGCTGAAGAGGA	-24.7	Novel-mir-94-5p	-	GCATACCAGGGATCATGGAC	-
Novel-mir-95	CTCAGTCAGAGCATTGAAGATGGGTCGTGGCTGTGTCTTCCAACATGACAACAACCCGAAGCACACAGCCAGAATAATCAAGGAGTGGCTCTGTAAGAA	-31.5	Novel-mir-95-5p	-	GCATTGAAGATGGGTCGTGGCTGT	-
Novel-mir-96	CATAAAACTCGCATTTAGTAGGCGAGTTCCTGATGATTTTAGGTCATCTGCCTCTAAATTGCCCTGTAGTTTTTTT	-20.9	Novel-mir-96-5p	-	GCATTTAGTAGGCGAGTTCCTGA	-
Novel-mir-97	GAGCCAGAAAGCCTAGTGGATCAAACACTGTAGCTACTGAGGATTCCCTGCCGTGTTGCTGGTTTCTCTTCCAGGGTGA	-18.8	Novel-mir-97-5p	-	GCCTAGTGGATCAAACACTGTAGC	-
Novel-mir-98	ACTGGGTCATGCCTTTACTGTGGTGCTCCTGACCATCTCATAGCCGCCTGCCTGGTGCGGCCAAAAGAGCGGGCCCGCCAGTGAAGGTGGGGACACTGAC	-39.2	Novel-mir-98-5p	-	GCCTTTACTGTGGTGCTCCTGACC	-
Novel-mir-99	AAATTAGCCAGCTATCGGAATTGCAGAAAGGCGTGATGAAGAAAGGGGTACCTAAGAAGACAAGATGGCGCCACTGTATTTGGCCTGCTGCCTGCTCG	-23.9	Novel-mir-99-5p	-	GCTATCGGAATTGCAGAAAGGCGT	-
Novel-mir-100	GTGGTGCAAGGCTGAGTGTCTATCCTTTCTGGGCTCACCATCACACCACCTGGTGGCCAACTTCAGAGCAGAGAGCATCACTAATGTCTAGCGATGCA	-30.6	Novel-mir-100-5p	-	GCTGAGTGTCTATCCTTTCTG	-
Novel-mir-101	CCCCTTGGAAGCTGGTTTCAGATGGTGGCATAGAGTATCGTCACCTGTCTAGCACCATTTGAAATCAGTGTTCTTGGGGC	-37.8	Novel-mir-101-5p	-	GCTGGTTTCAGATGGTGGCATAGA	-
Novel-mir-102	ACAAGACGAAGGAGATGATAGTGGACATGAGGACAAAGAGGAGATCTCATCGGCCACTGTTCATCCGGGGGCTAGA	-20.9	Novel-mir-102-5p	-	GGAGATGATAGTGGACATGAGGAC	-
Novel-mir-103-1	GCGCCTGCTCGGCACCGTCGTGTACTGAACCCATCCACTGCCGGTCTGTTTAGCACGGCGTTTGAACAGGCAAGTGTG	-27.9	Novel-mir-103-5p	Novel-mir-103-3p	GGCACCGTCGTGTACTGAAC	TCTGTTTAGCACGGCGTTTGAACA
Novel-mir-103-2	GCGCCTGCTCGGCACCGTCGTGTACTGAACCCATCCACTGCCGGTCTGTTTAGCACGGCGTTTGAACAGGCAATTGCG	-27.9	Novel-mir-103-5p	Novel-mir-103-3p	GGCACCGTCGTGTACTGAAC	TCTGTTTAGCACGGCGTTTGAACA
Novel-mir-104	CCTCCCTGTTGGGCATCTTATTCTGCGCCAGAGGCTCTCGAATGTCATCGGGTGCTGGGTAGTGTTTGGGTCCCGGGGTGGG	-33.3	Novel-mir-104-5p	-	GGGCATCTTATTCTGCGCCAGAGG	-

Novel-mir-105	TGTTAGGGTCGACAGGTCATGTTGCTGCTGCTGCAGTGCCTGAGCCAGTTGAGCGTTTGACAGTGAACT	-22	Novel-mir-105-5p	Novel-mir-105-3p	GGGTCGACAGGTCATGTTGCT	TGCCTGAGCCAGTTGAGCGTTTG
Novel-mir-106	GTGAGAAGGGGGGTCGCAGAGACGTCAAGGGGGACAGAGTCCAGCTGTTGAGAAGTAGCTAGTGGCTGAGCTGATA	-18.5	Novel-mir-106-5p	Novel-mir-61-3p	GGGTCGCAGAGACGTCAAGGGGGAC	GCTGTTGAGAAGTAGCTAGTGGC
Novel-mir-107	TTATTCATTAGGTCTTATTCTGATTGGCTGGGATGAAGGGGAGCCAGCCAATCAGAGCAGGTCCTCATGAATAAT	-37.9	Novel-mir-107-5p	-	GGTCTTATTCTGATTGGCTGG	-
Novel-mir-108	ACTGGTGCCAGAGATCTCGGGCTGGTGTGCGGGTCCCCCGATGAGACAGCGCTGGCAGCCGGGCGGTGCGAG	-27.3	Novel-mir-108-5p	Novel-mir-108-3p	GGTGCCAGAGATCTCGGGCTG	CGATGAGACAGCGCTGGCAGCCGG
Novel-mir-108	CTGGTGCCAGAGATCTCGGGCTGGTGTGCGGGTCCCCCGATGAGACAGCGCTGGCAGCCGGGCGGTGCGAA	-26.8	Novel-mir-108-5p	Novel-mir-108-3p	GGTGCCAGAGATCTCGGGCTG	CGATGAGACAGCGCTGGCAGCCGG
Novel-mir-109	GAGGTCTCAAAGCGGGTGTAGAAGGTGTTGAGGTCATCCGGTAGGCTGTCTGTGGGGCTGATGATGCTGGTGGTGGTCCT	-22	Novel-mir-109-5p	Novel-mir-51-3p	GGTGTAGAAGGTGTTGAGGTCATC	TGTCTGTGGGGCTGATGATGCTGG
Novel-mir-110	CGTTCATCAAGTACTGACTACTTTTTCGGCTGGCGTCAGCTACTGACTAAGGCATTGAGCGCCCCGCTGGCAAGACTGAGCACACCGGCATGAACA	-24.2	Novel-mir-110-5p	-	GTACTGACTACTTTTTCGGCTGG	-
Novel-mir-111-1	CAGTGATGCAGTACTTAAGGTGAGCGTTGGTGTGTCCTCATCGCCAGATGATCTCTTGACGTCCTGCTATCATCA	-24.2	Novel-mir-111-5p	-	GTACTTAAGGTGAGCGTTGGT	-
Novel-mir-111-2	TTTTGATGCAGTACTTAAGGTGAGCGTTGGTGTGTCCTCATCGCCAGATGATCTCTTGACGTCCTGCTATCATCA	-23.2	Novel-mir-111-5p	-	GTACTTAAGGTGAGCGTTGGT	-
Novel-mir-112	TTGACAGGCAGTAGTAAGTTCCTGTGGAGCACGCACAGTGTTTTAAGGCCCTTCTCTGGTTGCAC	-21.4	Novel-mir-112-5p	-	GTAGTAAGTTCCTGTGGAGCACG	-
Novel-mir-113	GGCGACAGGTCTGTAGTCGTTCAGTCCACTGATGGAGGTATTTTTGGGGACTGGAATGATGGTGGAGCATTTCAA	-22.4	Novel-mir-113-5p	Novel-mir-113-3p	GTAGTCGTTCAGTCCACTGATGG	TTTGGGGACTGGAATGATGGTGG
Novel-mir-114	AGCAGACCACTGCAGTGATACCCACAGCTATGACAGTCTCTGTGTTATAGAAACTGGCAATCATGCCCACCATGTA	-18.2	Novel-mir-114-5p	Novel-mir-114-3p	GTGATACCCACAGCTATGACAGT	TGTTATAGAAACTGGCAATCATGC
Novel-mir-115	CGACAATGGTGAGCATGACAGTGTTGCCCTGGGAGGGAGGAAGGCCGGTGACAAAGTCTAGGGCAATATGAGACCAGGGTC	-26.12	Novel-mir-115-5p	Novel-mir-115-3p	GTGTTGCCCTGGGAGGGAGGA	TGACAAAGTCTAGGGCAATATGA
Novel-mir-116	CTGCTCTGTTGTTTTTTTAGGTTTTGATTTTTTGTAGCATTGGATGAGAAAATCAAAGCCTAAAGAAAATACAGCGCTG	-29.8	Novel-mir-116-5p	-	GTTTTTTTAGGTTTTGATTTTT	-
Novel-mir-117	CCCAGTTTGCTAAAACATCCTGCAGAGCAGTGGCTATGTTTTCACTGGTGTGGGATTCAGGGAAATATGCAACTTCCAGGCAGCGTGTTGTTAGTTCAAA	-28.6	Novel-mir-117-5p	Novel-mir-117-3p	TAAAACATCCTGCAGAGCAGTGGCTA	TGCAACTTCCAGGCAGCGTGTTG
Novel-mir-118	ATAAACTAGCTAAAGGTTAATACATACTGACATATAAATGCATACACGTAGGTGGTGTGAACTCATATGTTGTTGAATGATTTTTGCTAACTGAT	-18.3	Novel-mir-118-5p	-	TAAAGGTTAATACATACTGACATA	-
Novel-mir-119	TGTTGAGCGATAACTCAGTAAAGCTAGGTCGGGGTCATCTTGTTTCAGGATCCACTTTGCTGTCTTTATGGCTCGTTC	-22.5	Novel-mir-119-5p	-	TAACTCAGTAAAGCTAGGTCGGGG	-
Novel-mir-120	TGCTTTTACATAACTGTTGAACTTTTCTGGCCAGTTGACGTCACTGGCTGTGCTAAAGTGCTCTATATTATGTTTTTTCATGAGAGCC	-20.7	Novel-mir-120-5p	-	TAACTGTTGAACTTTTCTGGCC	-
Novel-mir-121	CGCACATACATAAGACGGAGCAGTGTGGGCACGAGGAAGGTTAATGTGTGTGTGTGTGTGTGTATTGTAGTGCCTATGTAGTGTGT	-21.7	Novel-mir-121-5p	-	TAAGACGGAGCAGTGTGGGCACGA	-
Novel-mir-122-1	CCCCGGCCAATAAGAGAGGGTAAACCTGAACCGATCAAATAACAAGGCCCACTTCGCTTGTCTCGGGTTGAGTCTTTTGGTGGACTGGAGGT	-26.2	Novel-mir-122-5p	-	TAAGAGAGGGTAAACCTGAACCGA	-
Novel-mir-122-2	CCCTGGCCGATAAGAGAGGGTAAACCTGAACCGATCAAGGAACAACGCCCACCTCGCTTGTCTCGGGTTGAGTCTTTTGGTGGACTGGAGGT	-27.2	Novel-mir-122-5p	-	TAAGAGAGGGTAAACCTGAACCGA	-
Novel-mir-123	CTGGCCCAGATAAGATAGTTGAGCTTTGGCGACACCACTACTGTGATCTATTTAATTGTGTCCCTAGTGAAACTTTCTCTATAGGTATAGGGTTGA	-22.4	Novel-mir-123-5p	-	TAAGATAGTTGAGCTTTGGCGACA	-
Novel-mir-123	CTGGCCTGGATAAGATAGTTGAGCTTTGGCGACACCGCTACTGTGATCTATTTAATTGTGTCCCTAGTGAAACTTTCTCTATAGGTAGGG	-24.6	Novel-mir-123-5p	-	TAAGATAGTTGAGCTTTGGCGACA	-
Novel-mir-124	GGGATCTGGGTAAGCAAGAACAGGGGCCCGCACCAGTGCTTCCTTAAGCTGGTGGAGGGCAGCGCTGCAGGCCTCGTCCCA	-35.8	Novel-mir-124-5p	-	TAAGCAAGAACAGGGGCCCGCACC	-
Novel-mir-125	CACTTCAGTTTAAGTCTCACACCAGTGCAAAACAAAAATCGTGACGCTGCTCAGGTGTGGGACCCTAACTGTGGGGGA	-26.7	Novel-mir-125-5p	Novel-mir-125-3p	TAAGTCTCACACCAGTGCAA	CTGCTCAGGTGTGGGACCCTAA
Novel-mir-126	CACTTCAGTTTAAGTCTCACACCAGTGCAAAACAAAAATCGTGACGCTGCTCAGGTGTGGGACCCTAACTGTGGGG	-26.7	Novel-mir-126-5p	-	TAAGTCTCACACCAGTGCAAAA	-
Novel-mir-127-1	TGACACCATGTAATATACTGCACACAGAGACGGGCGTGTTTCTCTGTCTGATGTCTCTTTATTTCACGCCGTTTCTTGGTGGTTCTTACAACAAGACC	-24.49	Novel-mir-127-5p	-	TAATATACTGCACACAGAGACGGG	-
Novel-mir-127-2	TGACACCATGTAATATACTGCACACAGAGACGGGCGTGTTTCTCTGTCTGATGTCTCTTTATTTCGCGCCGTTTCTTGGTGGTTCTTACAACAAGACA	-24.37	Novel-mir-127-5p	-	TAATATACTGCACACAGAGACGGG	-

Novel-mir-127-3	TGACACCATGTAATATACTGCACACAGAGACGGGCGTGTTTCTCTGTCTGATGTCTCTTTATTTCGCGCCGTTTCTTGGTGGTTCTTACAACAAGACC	-24.37	Novel-mir-127-5p	-	TAATATACTGCACACAGAGACGGG	-
Novel-mir-127-4	TTGGTGGCTGTAATATACTGCACACAGAGACGGGCGTGTTTCTCTGTCTGATGTCTCTTTATTTCGCGCCGTTTCTTGGTGGTTCTTACAACAAGACAAG	-26.57	Novel-mir-127-5p	-	TAATATACTGCACACAGAGACGGG	-
Novel-mir-127-5	TTTGATTCTGTAATATACTGCACACAGAGACGGGCGTGTTTCTCTGTCTGATGTCTCTTTATTTCGCGCCGTTTCTTGGTGGTTCTTACAACAAGACA	-24.17	Novel-mir-127-5p	-	TAATATACTGCACACAGAGACGGG	-
Novel-mir-136	GAGACTACGGTAATCAATACATGGACGGAGGCCAAGCAAACCTGACAATAATCTACAGGCACTTGGGCGTTCATGTGAGCTTGCCTGACATCTA	-25.1	Novel-mir-136-3p	-	TAATCAATACATGGACGGAGGC	-
Novel-mir-128-1	AGGATTGGGGTAATGTGAGCATGTCGACGGGTGTCAGTGAGCAGCCGGGCAGCGGCATTTTGCACCAGCTGCAAC	-23.8	Novel-mir-128-5p	Novel-mir-128-3p	TAATGTGAGCATGTCGACGGG	GCAGCCGGGCAGCGGCATTTTGCAC
Novel-mir-128-2	AGGATTGGGGTAATGTGAGCATGTCGACGGGTGTCAGTGAGCAGCCGGGCAGCGGCATTTTGCACCAGCTGCAGC	-23.8	Novel-mir-128-5p	Novel-mir-128-3p	TAATGTGAGCATGTCGACGGG	GCAGCCGGGCAGCGGCATTTTGCAC
Novel-mir-129	GTCATGAATTTACACAGTCTAGATCAAGGAATGGATTTTTTTTAACTCTCTCTGCTGAGTTTCTGATTCATAACTTAAAGCTGTGGGCTGCTGGGAGGACA	-22.3	Novel-mir-129-5p	-	TACACAGTCTAGATCAAGGAATGG	-
Novel-mir-130	AAAGCTCAAATACAGATGAGTGGAAGTGAGAGGTGTGCTGCTTCTTATCCCTCCACTATTCCTAGTTTAAGGGCACA	-23.4	Novel-mir-130-5p	-	TACAGATGAGTGGAAGTGAGAGGT	-
Novel-mir-131	CCTCTGTCCATACCTTTACTACCCTGACCTGAGGTGACACTTTTTTCCACCACTGGAGTATACACAGGTAGTAGGTGAACAAGGTTGTGGTCAGTGA	-28.71	Novel-mir-131-5p	-	TACCTTTACTACCCTGACCTGAGG	-
Novel-mir-132-1	CCTCTGTCCATACCTTTACTACCCTGACCTGTGGTGACACCTTTTTCACCACTGGAGTATACACAGGAAGCAGGTGGACAAGGTTGTGGCCAGAGC	-37.9	Novel-mir-132-5p	-	TACCTTTACTACCCTGACCTGTGG	-
Novel-mir-132-2	CCTCTGTCCATACCTTTACTACCCTGACCTGTGGTGACACCTTTTTCACCACTGGAGTATACACAGGAAGCAGGTGGACAAGGTTGTGGTCAGAGC	-39	Novel-mir-132-5p	-	TACCTTTACTACCCTGACCTGTGG	-
Novel-mir-132-3	CCTCTGTCCATACCTTTACTACCCTGACCTGTGGTGACACCTTTTTCACCACTGGAGTATACACAGGAATCAGGTGGACAAGGTTGTGGTGAGC	-33.5	Novel-mir-132-5p	-	TACCTTTACTACCCTGACCTGTGG	-
Novel-mir-132-4	CCTCTGTCCATACCTTTACTACCCTGACCTGTGGTGGCACCTTTTTTACCACTGGAGTATACACAGGAAGTAGGTGGACAAGGTTGTGGTCAGAGC	-38	Novel-mir-132-5p	-	TACCTTTACTACCCTGACCTGTGG	-
Novel-mir-132-5	CCTCTGTCCTTACCTTTACTACCCTGACCTGTGGTGACACCTTTTTCACCACTGGAGTATACACAGGAATCAGGTGGACAAGGTTGTGGTCAGAGT	-37.6	Novel-mir-132-5p	-	TACCTTTACTACCCTGACCTGTGG	-
Novel-mir-133	GTGCCGTGAATACGAAACACATGCTGAACCAGGAGCTGGGTGGTTTCTAGAGCAGTGGGGAGTTGTGGAAGGGCAAGA	-23.4	Novel-mir-133-5p	Novel-mir-133-3p	TACGAAACACATGCTGAACCAGGAGC	TTCTAGAGCAGTGGGGAGTTGTGG
Novel-mir-134	CTGGACTCTCTACTACTCTGGAGTTGCCCACGGTGACGACGGTTTGCTGTCTATATATGGCAACTTTAATAGTGCAAACAATTA	-21.3	Novel-mir-134-5p	-	TACTACTCTGGAGTTGCCCACGGT	-
Novel-mir-135	TGAACGAACCTACTCGTGAACTGGAGTCTCTCCTTGATGGGGAGAGGGTGCACCCATGAAAGGGACTATTTC	-23.1	Novel-mir-135-5p	-	TACTCGTGAACTGGAGTCTCT	-
Novel-mir-136	ATGTGGGTGATACTGCATCAGGAACTGATTGGCTGATGCTCAGTAGCCAACAGTACCTGATGCATTACCATCAGCATC	-37.5	Novel-mir-136-5p	-	TACTGCATCAGGAACTGATTGGC	-
Novel-mir-137	AAGGCGCGGCTACTGGGCATGCAGGATAGTTGTCATCAGGAAGAGCCACATAGTCCTGTACCATTAACTGACTGTCCATATCTGCTCTCATTTCT	-25.7	Novel-mir-137-5p	Novel-mir-137-3p	TACTGGGCATGCAGGATAGTTG	TAACTGACTGTCCATATCTGC
Novel-mir-138-1	ACATTCAATATACTGTATAACTCAAGCTGCACTAACTTAAATGTAACATCTTCTGGTTAAGTCGTAGCAGTGATACATTAAGTTGGGTTT	-19.4	Novel-mir-138-5p	-	TACTGTATAACTCAAGCTGCACT	-
Novel-mir-138-2	ACATTCAATATACTGTATAACTCAAGCTGCACTAACTTGAATAACATCTTCAGGTTAAGTTGTAGCAGTAATACATTAAGTTGGTTTA	-24.4	Novel-mir-138-5p	-	TACTGTATAACTCAAGCTGCACT	-
Novel-mir-152	TGGGCTCCTATACTGTGGAATGGTCTGCCATGGGAGATTAGATCTGCTCCGAACCAGACTGTTTTTAGATCACTGCTCAA	-21.92	Novel-mir-152-3p	-	TACTGTGGAATGGTCTGCCATGGG	-
Novel-mir-139	TACGGGGAGTTACTGTGTGAGAGGAAGGCGGTGATTTCGTGTTTAAGCTCGTAAAAGCGTGACATTACCTTTCCCCTTGAAAGCCAGCAAACTTCTGAAT	-25.6	Novel-mir-139-5p	-	TACTGTGTGAGAGGAAGGCGGTGA	-
Novel-mir-140	TCTGCTCCTGTACTTGGTCAAAGCTCCTCAGTGTTGGATAGATGCCTGAGAGGTTCTGAACAAGTATAGGATCTCTG	-30.5	Novel-mir-140-5p	Novel-mir-140-3p	TACTTGGTCAAAGCTCCTCAG	TGAGAGGTTCTGAACAAGTAT
Novel-mir-141	CCTTAGCCTCTACTTTAGGTAGGCAAACCTCTTCAAGGTAAGGCCACATAGTGAGGTCCTCCTGCTGTAGAATATTCTC	-21.1	Novel-mir-141-5p	-	TACTTTAGGTAGGCAAACCTCTTC	-
Novel-mir-142	TCTCCCAATCTACTTTCTTAGGATATCTGTCCCTTAACCATTTTTTTTTCAGCTGCCTGGGTCTTGGTTTGCAGAGATGTTGAGGAGGGAAGTGGTGACA	-26	Novel-mir-142-5p	-	TACTTTCTTAGGATATCTGTCCCT	-
Novel-mir-143-1	TCTGTGGGCCTAGACTATGGAATGCCCTGCCGGTTGTACTACGGGTGGAAAGCGATCTAGTGGCCTTTAAATCGGCCTTAAAGAC	-24.1	Novel-mir-143-5p	-	TAGACTATGGAATGCCCTGCCGGT	-
Novel-mir-143-2	TTTGTGGCCCTAGACTATGGAATGCCCTGCCGGTTGAACTACGGGTGGAAAGCGATCTAGTGGCCTTTAAATCGGCCCTAAAG	-21.8	Novel-mir-143-5p	-	TAGACTATGGAATGCCCTGCCGGT	-

Novel-mir-144	TGCAATGAAATAGATAAAGCCTAAATTGGTCGGAGCACCAGTCAGTCAGGTCAGGTCCTTTGTTACCAGTCCTGGGTCAGTCAGCC	-19.3	Novel-mir-144-5p	-	TAGATAAAGCCTAAATTGGTCGGA	-
Novel-mir-145	GCAGCCCCTGTAGATGAGCCTGAGGTATTTGCATGAGCTGGTGCAGAACTGGAGCTCAATCTAGATGTTGAAGGTGCTGA	-26.2	Novel-mir-145-5p	-	TAGATGAGCCTGAGGTATTTGCAT	-
Novel-mir-146	GTCTGATAGATAGCAAGATCTGGACAATTGGAGGAAGTTGAGAAATATACAGCTATTTATGATCCAGTGCTGGTTTATTTTATCAGGT	-21.6	Novel-mir-146-5p	-	TAGCAAGATCTGGACAATTGGAGG	-
Novel-mir-147	TGTCAGGCTGTAGCAGCACATCATTACTGGTATTCCTTCTCTCTGCATTGCTGGTATGATATGTGCTGCTCCTATCAGACAC	-28.2	Novel-mir-147-5p	-	TAGCAGCACATCATTACTGGTA	-
Novel-mir-148	GCACTTCCAGTAGCTGTGGATCTGACCTGCACAACATTCAGGAGGAATTTTTCTTTCTCTTTAGTTCAGCTCATACTTAGGATGTCTGGTGTG	-19.6	Novel-mir-148-5p	-	TAGCTGTGGATCTGACCTGC	-
Novel-mir-149	AGAGATTCAATAGGACTTTACTCTCTATGCTGGGAATGCTGAATCAAGAAAAGAAACGTCAGTGGAGCCAACATGTAGGATATTTAGTCCATGCATACAAC	-21.9	Novel-mir-149-5p	-	TAGGACTTTACTCTCTATGCTGGG	-
Novel-mir-150-1	AGTGATCTGTTAGGATGATAAGGCTCTACGGGGTCTTTAAGATACGATGGAGCCTTGTCATTAAGGGCTTTGTATG	-29.3	Novel-mir-150-5p	-	TAGGATGATAAGGCTCTACGGGG	-
Novel-mir-150-2	AGTGCTCTGTTAGGATGATAAGGCTCTACGGGGTCTTTAAGATACGATGGAGCCTTGTCATTAAGGGCTTT	-33.2	Novel-mir-150-5p	-	TAGGATGATAAGGCTCTACGGGG	-
Novel-mir-151	AGTGCTCTGTTAGGATGATGAGGCAGTATGAAGTCTTTAAGATACAATGGAGCCTTGTCATTTAGGGCTTT	-23.3	Novel-mir-151-5p	-	TAGGATGATGAGGCAGTATGAAGT	-
Novel-mir-152	CATGGTTCAATAGGATGTTTGGTAAATGGAAGGGGGTTATTGTCTCTGTACTCATTACAGTGGCAATTATGATAACTGTCTTGATTACATGTGGTTGTTGTTGTG	-21.8	Novel-mir-152-5p	Novel-mir-152-3p	TAGGATGTTTGGTAAATGGAAGGG	TGATAACTGTCTTGATTACATGTGGT
Novel-mir-153-1	AATGATACACTAGGCAATTGTTTGGGCAACTCTGATTAGCAACAGTGCCTGAGAAATTGCCTTGGTATTGCT	-26.1	Novel-mir-153-5p	-	TAGGCAATTGTTTGGGCAACTCT	-
Novel-mir-153-2	AATGATACACTAGGCAATTGTTTGGGCAACTCTGATTAGCAACAGTGCCTGAGAAATTGCCTTGGTATTGCTGCTTT	-26.1	Novel-mir-153-5p	Novel-mir-153-3p	TAGGCAATTGTTTGGGCAACTCT	TGCCTGAGAAATTGCCTTGGTA
Novel-mir-154	GCAGGGGGGCTAGGCGTGTCACTGCGTGTCACAGTCACTGCTTGCGCACGGGGCCACGCCCTGCCTACCTGTC	-36.5	Novel-mir-154-5p	-	TAGGCGTGTCACTGCGTGTCA	-
Novel-mir-155	GCAGGGGGGCTAGGCGTGTCACTGCGTGTCACAGTCACTGCTTGCGCACGGGGCCACGCCCTGCCTACCTGTCT	-36.5	Novel-mir-155-5p	Novel-mir-155-3p	TAGGCGTGTCACTGCGTGTCACA	TGCGCACGGGGCCACGCCCTGC
Novel-mir-156	AGCTGTTTTTTAGGTACTATAGTTGTTAAAACCTGAGGTTTATGAAGTCTCTAGTCTTTGAAGTTGGTGTAGTGTCTAGGAGTT	-19.8	Novel-mir-156-5p	Novel-mir-156-3p	TAGGTACTATAGTTGTTAAAACCTGA	TAGTCTTTGAAGTTGGTGTAGTG
Novel-mir-157	AAGGGCCTCTTAGGTTGGCTTGTTCCTGGGCCCTGATCTACAGTAGTAACAGACAAATATTAGGATGGCTTAG	-18.2	Novel-mir-157-5p	Novel-mir-157-3p	TAGGTTGGCTTGTTCCTGGGC	GTAGTAACAGACAAATATTAGG
Novel-mir-158	GACCCACCAATAGGTTGTAAACATGGCACATTAAGTGGCCATCTGAACAAGTGCGAAGTGAATATACCTGCTTGTGTGAGGGA	-20	Novel-mir-158-5p	-	TAGGTTGTAAACATGGCACATT	-
Novel-mir-159	TGGAAAAAGGTAGTACGTTCGCTTCAGCACCGGTGTCAAGCTTGAATTTTACTGCCACACCCCTGATGCTTGCTGTTGTGTACCATGCACCTTCCT	-23.32	Novel-mir-159-5p	-	TAGTACGTTCGCTTCAGCACCGGT	-
Novel-mir-160-1	TACTCTGCGGTAGTAGATGCTCTGCAGAGAGGGCAGTGGAGTCCTGGTGATCTTCTCAGTCTTTACCACTCTCTGCAGGTGCGTGTGGTCCACAGCACT	-39.6	Novel-mir-160-5p	-	TAGTAGATGCTCTGCAGAGAGGGC	-
Novel-mir-160-2	TACCCTGCGGTAGTAGATGCTCTGCAGAGAGGGCAGTGGCGTCCTGGTGATCTTCTCCGCAGTCTTTACCACTCTCTGCAGGTGCTTGCGATCCATAGCAG	-37.4	Novel-mir-160-5p	-	TAGTAGATGCTCTGCAGAGAGGGC	-
Novel-mir-161	CGGGTTGAGGTAGTAGGTTGTATGGTTTAGAATTACACCCTGGGAGTTAACTGTACAACCTTCTAGCTTTCCTTGGA	-27.2	Novel-mir-161-5p	Novel-mir-161-3p	TAGTAGGTTGTATGGTTTAGA	GTTAACTGTACAACCTTCTAGC
Novel-mir-162	GTTGCCGGGGTAGTTAAGGAACTCCTCGGTGGCAAGGCTCAGGGTGTGAATGAGGCCTGCCCCGAGTTTCTCAAGTCTCTGGATGT	-33.3	Novel-mir-162-5p	-	TAGTTAAGGAACTCCTCGGTGGCA	-
Novel-mir-163	AGATGCCCTGTAGTTACTCTGTACTTGTTGGGGGAGCATGGAGATGCTAATGGCATGGCCATAGTGATTGTGCCAGGTGCAAAC	-25.5	Novel-mir-163-5p	-	TAGTTACTCTGTACTTGTTGGGG	-
Novel-mir-164-1	TCTTTGACAGTAGTTTACATTTCTATGCCTCTGCACAGGAAACAGCTGTGGCTGGAGAAATAATTCTACATGGTGTCAACCT	-21.9	Novel-mir-164-5p	-	TAGTTTACATTTCTATGCCTCTGC	-
Novel-mir-164-2	TCTTTGACAGTAGTTTACATTTCTATGCCTCTGCACAGGTGACAGCCTGGCTGGAAAAATAATTCTACATGGTGTCAACCT	-21.2	Novel-mir-164-5p	-	TAGTTTACATTTCTATGCCTCTGC	-
Novel-mir-165	TGCAAGATGTTAGTTTTATGATCAGTGGTGATATTCACGCCGTCAGCGGAGACAACACGACTGAGAAAAGTGACTTTTTGTC	-25.6	Novel-mir-165-5p	-	TAGTTTTATGATCAGTGGTG	-
Novel-mir-166	ACATAGCCTATATAGAAAGACAAAGGGCACTGGCTATAGGCTACAATGCCAAGCAATGGTGCACTGATTCACACTGAGTTGTGTA	-21.1	Novel-mir-166-5p	-	TATAGAAAGACAAAGGGCACTGGC	-
Novel-mir-167-1	CAATTGTTGGTATAGAGTGAAAACAGCAGAGGGGAGAGCAAACATCCCTGGGTGGCACCTGTGCTGATTGTCTGTGTGCTGGATGTGT	-26.4	Novel-mir-167-5p	-	TATAGAGTGAAAACAGCAGAGGGG	-

Novel-mir-167-2	CAGTTGTTGGTATAGAGTGAAAACAGCAGAGGGGAGAGCAGACATCTCTCGGGGGGCGCCTGTGCCTACTGT	-22.2	Novel-mir-167-5p	-	TATAGAGTGAAAACAGCAGAGGGG	-
Novel-mir-168-1	ACGTTATACTTATAGGTTAAGGATTTTATTGTGCTCATTGCCCGAGTTATTGTCAATTGAGTTAGTAAAGGTCTTAAGCTCCTGAGAGAGACTA	-20.2	Novel-mir-168-5p	-	TATAGGTTAAGGATTTTATTGTGC	-
Novel-mir-168-2	GCGTTATACTTATAGGTTAAGGATTTTATTGTGCTCATTGCTCGAGTTATTGTCAATTGAGTTAGTAAAAGTCTTAAGCTCCTGAGAGAGACAA	-21.9	Novel-mir-168-5p	-	TATAGGTTAAGGATTTTATTGTGC	-
Novel-mir-169	GCGTCTCCAGTATCAATGAAGTGCTGCACCAGCCCTGTCCTGGTGCAGTCTTCTGTTTTTGCTGCGAAAATGTC	-28.7	Novel-mir-169-5p	-	TATCAATGAAGTGCTGCACCAGC	-
Novel-mir-170-1	TCATGCAGGTTATCCAGAGCCTGCGTGAAGCCCCTCGTGTGCACCAGCAGGTCATCCAGGTAAACTACGCAGCGGGTTCTGGGAATGTCTTTGAG	-33.8	Novel-mir-170-5p	Novel-mir-170-3p	TATCCAGAGCCTGCGTGAAGC	TAAACTACGCAGCGGGTTCTGGGAA
Novel-mir-170-2	TCGTGCAGGTTATCCAGAGCCTGCGTGAAGCCCCTCGCGTGCACCAGCAAGTCGTCCAGGTAAACTACGCAGCGGGTTCTGGGAATGTCCTTGAG	-31.3	Novel-mir-170-5p	Novel-mir-170-3p	TATCCAGAGCCTGCGTGAAGC	TAAACTACGCAGCGGGTTCTGGGAA
Novel-mir-171	GCATGGACTCTATGATTGTCTCTGTTGAGGTCTAATGTTCAACATCTCGAAACCCAAACGTCAGGCTCTGGAAGAGTACATCGAAACCCCACGACATGCC	-19.2	Novel-mir-171-5p	-	TATGATTGTCTCTGTTGAGGTCTA	-
Novel-mir-172	GGTTCAGCCCTATGCAGAACAGCAGCAGGGGACAAATACTGCACTTGATGTTGACTTGTGCAATTGGCTTGCAGTTGGCCTCTAGGGTTGTTTTTCA	-30.3	Novel-mir-172-5p	-	TATGCAGAACAGCAGCAGGGGAC	-
Novel-mir-173	GGTTCAGGCCTATGCAGAACAGCAGCGGGGACAGAGTATCTCCATGGTAGATACCGCACTTGATGTTGACTTGTGTAATTGGCTTGAAGTTGG	-31.4	Novel-mir-173-5p	Novel-mir-173-3p	TATGCAGAACAGCAGCGGGGACAGA	TTGATGTTGACTTGTGTAATTGGC
Novel-mir-174	GGCATCACTGTATGGAGGAGGTGGAGCAGTGGCAGTATTTGTTTTTAAATTCTGGACAAAATCAGCTTTGGGCATAAGTGGTCTTGTCA	-20.1	Novel-mir-174-5p	-	TATGGAGGAGGTGGAGCAGTGGC	-
Novel-mir-175	GTGTAGCGCATATGTAGTGTGAGAAGAGTCAAGCCAAAACGCACAGTGTCTGTCAGCGTAGCATTCCACCCACACGTACATAATGCGGCACT	-28.7	Novel-mir-175-5p	-	TATGTAGTGTGAGAAGAGTCA	-
Novel-mir-176	AAGGAAGCTCTATGTCAACACTGTCCAGCAGCGCAGTCAACTGTTCTGGGCTCGGACGCATCTGGGATGGACCATCACA	-22.6	Novel-mir-176-5p	-	TATGTCAACACTGTCCAGCAGCGC	-
Novel-mir-177-1	AAGGAAGCCCTATGTCAACAGTGTCCAGAAGCGCAGTCGATTGTTCTGGGCTCAGAGGCATCTGGGATGGACCAT	-21.9	Novel-mir-177-5p	-	TATGTCAACAGTGTCCAGAAGCGC	-
Novel-mir-177-2	AGGGAAGCCCTATGTCAACAGTGTCCAGAAGCGCAGTCAACTGCTCTGTGCTCTGAGGCATCTGGGATGAACCTT	-26.7	Novel-mir-177-5p	-	TATGTCAACAGTGTCCAGAAGCGC	-
Novel-mir-177-3	TTCAGAGCCCTATGTCAACAGTGTCCAGAAGCGCAGTTGACTGCTCTGGGCTTGAAGGCATGTGGGATGGAC	-31.4	Novel-mir-177-5p	-	TATGTCAACAGTGTCCAGAAGCGC	-
Novel-mir-178	GGCGGGGATCTATGTGTGCTTGGACGTGACGGATGAGGAACTGTTCCCCCGGTTTTATTGGGGATCCCGGCTT	-25	Novel-mir-178-5p	-	TATGTGTGCTTGGACGTGACGGA	-
Novel-mir-179	TGGCCCTGGATATGTTGTTAGCTGAGAGAGGAGGAGTATGTAAAATGTTTGGCACATTTTGTTGTACTTTCATACCTAACAATACCTTCCCAGCTGGCAG	-25.4	Novel-mir-179-5p	-	TATGTTGTTAGCTGAGAGAGGAGG	-
Novel-mir-189-1	ATAATCTCCTTATTGATGTCATTGTCCTTATACCATAAAAAGCGAAGGAAGTTTCGATGATCTTTGTGCACTAAGAAACAGTGGAACATCTGCTGGATGTC	-19.6	Novel-mir-189-3p	-	TATTGATGTCATTGTCCTTATACC	-
Novel-mir-189-2	ATAATCTCCTTATTGATGTCATTGTCCTTATACCATAAAAAGCGAAGGAAGTTTCGATGGTCTTTGTGCACTAAGAAACAGTGGAACATCTGCTGGATGTC	-19.6	Novel-mir-189-3p	-	TATTGATGTCATTGTCCTTATACC	-
Novel-mir-180	AGGGGGGTTGTATTGTCGGGAGAGGGCGTCTGGTTTCTGGTTTCGGGACCCCGGCCGATAAGAGAGGGTAAACCAGAACCG	-25	Novel-mir-180-5p	-	TATTGTCGGGAGAGGGCGTCTGG	-
Novel-mir-181	AGGGTCTGACTATTGTGGGAACTTTGCGACAGAACAAACCAGACATTCCTCCAGTCATGAAGGCTTCCAGGTCCAGAGAGGTCCA	-18.4	Novel-mir-181-5p	-	TATTGTGGGAACTTTGCGACAGA	-
Novel-mir-182	CTGAGTCAGCTATTTAGGAGTCTGATGGCAGAGGGAAAGAAAGAGTTTTTCAGTCTAGAAGTCCTGCATTTCACACTTCTGAGCCTCTGACCGGAGGGCA	-29.3	Novel-mir-182-5p	Novel-mir-182-3p	TATTTAGGAGTCTGATGGCAGAGG	CATTTCACACTTCTGAGCCTCTGA
Novel-mir-183	TAGGATGAGATATTTTGGGAACTGGGACAACACAGGATGTCTTCCATAGGGCTGGGACTCTCTCCAG	-18.8	Novel-mir-183-5p	-	TATTTTGGGAACTGGGACAACA	-
Novel-mir-184-1	TTGAATGGGTTCAAAATGCAGCAGAGGAGGTGTCAGCTAACACTTCCTGTGTTGTATGTGCTGGACCTCGTCCT	-34.3	Novel-mir-184-5p	Novel-mir-184-3p	TCAAAATGCAGCAGAGGAGGTGTC	TTCCTGTGTTGTATGTGCTGG
Novel-mir-184-2	TTGAATGGGTTCAAAATGCAGCAGAGGAGGTGTCAGTTAACACTTCCTGTGTTGTATGTGCTGGACCTCGTCCT	-34.3	Novel-mir-184-5p	Novel-mir-184-3p	TCAAAATGCAGCAGAGGAGGTGTC	TTCCTGTGTTGTATGTGCTGG
Novel-mir-185	TCTGCCTCCCTCAAAGCCTCAAGGGATGACGGTGTGGCGAGCCGTACGTGCTGTCGTAGTCTCTCTGGTGCAAGGCCCCGCAGAT	-29	Novel-mir-185-5p	Novel-mir-185-3p	TCAAAGCCTCAAGGGATGACGG	GTCGTAGTCTCTCTGGTGCAAGG
Novel-mir-186	TCTGCCTCCCTCAAAGCCTCAAGGGATGACGGTGTGGCGAGCCGTACGTGCTGTCGTAGTCTCTCTGGTGCAAGGCCCCGCAGATG	-29	Novel-mir-186-5p	Novel-mir-186-3p	TCAAAGCCTCAAGGGATGACGGTG	TCGTAGTCTCTCTGGTGCAAGGC
Novel-mir-187-1	GGTATGGTCTTCAACTTGTCCGCTGCAGACTGGCCTAATACCTCTCTGACCATGTCCAGGGCCGCAGGTAAGATGAGCTCCTCTGCTA	-22.47	Novel-mir-187-5p	-	TCAACTTGTCCGCTGCAGACTGGC	-

Novel-mir-187-2	GGTATGGTCTTCAACTTGTCCGCTGCAGACTGGCCTAATACTTCTCTGACTATGTCCAGGGCCGCAGGTAAGATGAGCTCCTCTGCTG	-22.47	Novel-mir-187-5p	-	TCAACTTGTCCGCTGCAGACTGGC	-
Novel-mir-188	ATTTATACAGTCAAGTTGGTACTGTGGCAGTTCAAGACTGTTATCAGTCTCTGTCTACCAGACAGAGCCGGGTCAGTCACAAACTTTTCTGCATACTC	-29.8	Novel-mir-188-5p	-	TCAAGTTGGTACTGTGGCAGT	-
Novel-mir-189	GGAGGGGAGTTCAATGTCTCCTCCCCGGCTCTGACACCATGCGGGCTTAGCTGGATGGAGTGCCCGGGGGTTGTGTCGTTGGCCTTTCCCTCCC	-44	Novel-mir-189-5p	-	TCAATGTCTCCTCCCCGGCTCTG	-
Novel-mir-190	GGGAGGGGGTTCAATGTCTCCTCCCCGGCTCTGAACTGACATGGGCTTAGCCGGATGTAGCGCCCGGCCGGGGGATATGTTGTTGGCTTTTCCCTCCCT	-43.4	Novel-mir-190-5p	-	TCAATGTCTCCTCCCCGGCTCTGA	-
Novel-mir-191-1	GACAGCCACCTCACAGTGCAGTGCAGCGGCAGGTCACCTGGAAGCCCTTGGTCCAAATAGTTACTTGCCCCACTGTATTTGTGAATACACAGCCGTCC	-31.7	Novel-mir-191-5p	-	TCACAGTGCAGTGCAGCGGCAG	-
Novel-mir-191-2	GACAGCCACCTCACAGTGCAGTGCAGCGGCAGGTCACCTGGAAGCCCTTGGTCCAGTTCAGTGATGAGCTTCTTCAATTGCCTGTGGTTAAGCGCAT	-30.6	Novel-mir-191-5p	-	TCACAGTGCAGTGCAGCGGCAG	-
Novel-mir-192-1	AGATGTTCCATCACTATGCACCTCAGGACGCCCCAATCATTCGCCGTGATTGGAGTCCTCAGCGTCTCCGGAGTGAAAGTTTCACC	-24	Novel-mir-192-5p	-	TCACTATGCACCTCAGGACGCC	-
Novel-mir-192-2	AGATGTTCCATCACTATGCACCTCAGGACGCCCCAGTCATTCGCCGTGATTGGAGTCCTCAGCGTCTCCGGAGTGAAAGTTTCACC	-24	Novel-mir-192-5p	-	TCACTATGCACCTCAGGACGCC	-
Novel-mir-192-3	AGATGTTCCATCACTATGCACCTCAGGACGCCCCCATCATTCGCCGTGATTTGAGTCCTCAGCGTCTCCGGAGTGAAAGTGACTCTTCCTCC	-20.6	Novel-mir-192-5p	-	TCACTATGCACCTCAGGACGCC	-
Novel-mir-192-4	AGATGTTCCATCACTATGCACCTCAGGACGCCCTAATCATTCGCCGTGATTGAAGTCCTCAGCGTCTCCAGAGTGAAAGTTTCACC	-19.3	Novel-mir-192-5p	-	TCACTATGCACCTCAGGACGCC	-
Novel-mir-193	GGCTCTGTTTTCAGAATCATCTTTTCTCTTGGCCATTGTTAGGGGCTAGCTTTGACTTGAAATTAGGGTTG	-18.4	Novel-mir-193-5p	-	TCAGAATCATCTTTTCTCTTGG	-
Novel-mir-194-1	TCCAGCTCTGTCAGACTAGAGGACTGGGAGCTGAGAACCGAGGACAGAGCTTCACAGCTCCTCTCTGACAGGTTACAACCGCTGAGTCTGGAG	-38.46	Novel-mir-194-5p	-	TCAGACTAGAGGACTGGGAGCTGA	-
Novel-mir-194-2	TCCAGCTCTGTCAGACTAGAGGACTGGGAGCTGAAAACTGAGGACAGAGCTTCACAGCTTCTCTTTGACAGGTTACAATCACTCAGGCTGGAG	-35.13	Novel-mir-194-5p	-	TCAGACTAGAGGACTGGGAGCTGA	-
Novel-mir-195	ACAACAGCACTCAGAGAGGTCTGTTAGAGAGGCTCGAAGCACAAAGGGAGCTCAGTCATATATAAGCTTTTACACAGGGTTGGGAGGCATCCGA	-18.1	Novel-mir-195-5p	-	TCAGAGAGGTCTGTTAGAGAGGC	-
Novel-mir-196	CTCCTGGAAGTCAGATGGTTAACTGGACAGAGCCTCCAGCCACAGTTGTGAAAAGATTCTCTGTTCCTCTTTTTGCCTCGGATTTTATAGGAT	-27.5	Novel-mir-196-5p	-	TCAGATGGTTAACTGGACAGAGC	-
Novel-mir-197-1	TGGCTATGGATCAGATGTTAGCAGCGCAAGGAGGTGTTTGTAAAATCATTGGCCCAGAGTGTTGCACTTATATCCCAGATAATTCAGTCT	-20.2	Novel-mir-197-5p	-	TCAGATGTTAGCAGCGCAAGGAG	-
Novel-mir-197-2	TGGCTATGGATCAGATGTTAGCAGCGCAAGGAGGTGTTTGTAAAATTATTGGCTCAAAGTGTTGCACTTATATCCCAGATAATTCAGTCT	-18.92	Novel-mir-197-5p	-	TCAGATGTTAGCAGCGCAAGGAG	-
Novel-mir-198	ACGGGGACCGTCAGCGTTGAAATCTCGGTACCTCCTGCGAAAAGCACAAACGTGATAGTTCAAGCCAGAATGCACAGCGACTGTGGGCGTACCCTT	-24.3	Novel-mir-198-5p	-	TCAGCGTTGAAATCTCGGTACC	-
Novel-mir-199	CATGTAAACCTCAGGAGTTGTTGTTCTTTGCCTTGTAATGCTTAAATGACAGGAATAGGACTGCTGAGATTTACTGTAA	-20.1	Novel-mir-199-5p	Novel-mir-100-3p	TCAGGAGTTGTTGTTCTTTGC	GACAGGAATAGGACTGCTGAGA
Novel-mir-200-1	CATTCCGTGCTCAGGATGTCAGATGCGCGCGCTCAAAAAAACATTCCGTGCTCAGGATGTCCCATTGCTCGCTCAGGAAAT	-20.76	Novel-mir-200-5p	-	TCAGGATGTCAGATGCGCGCGC	-
Novel-mir-200-2	TATTCCGTGCTCAGGATGTCAGATGCGCGCGCTCAAAAAAACATTCCTCAGATGCGCACGCTCAAAATGTCCCATTGCTCGCTCAGGAAAT	-22.29	Novel-mir-200-5p	-	TCAGGATGTCAGATGCGCGCGC	-
Novel-mir-201	TTCACACCAGTCAGTGTGTGAACGAGAAAGAGGCGGACCCAGGTAGCCGACTTCAGTGCATGCAGTGATTTATTATATACAGTGATGAAG	-23.5	Novel-mir-201-5p	-	TCAGTGTGTGAACGAGAAAGAGGC	-
Novel-mir-202-1	GTTAAAGCCATCAGTTGAGTTGGTGCCGCAGATGGCTGCCTCGGTTCTGTACCGGAAACAAATTTCTTCTGTGTGTAACATACTTGGCCAGTAAAGGTGA	-23.2	Novel-mir-202-5p	-	TCAGTTGAGTTGGTGCCGCAGAT	-
Novel-mir-202-2	GTTAGACCCATCAGTTGAGTTGGTGCCGCAGATGGCTGCCTCGGTTCTGTACCGGCAACAAATTCCCTGTGTGTAACA	-23	Novel-mir-202-5p	-	TCAGTTGAGTTGGTGCCGCAGAT	-
Novel-mir-203	CTGGCTCTCCTCAGTTTGCGGAAGAAGTAGAGGTGTGTTTGAGCTTTCTTCTACTGCTTCTATTCTACTGCTGACTCCAA	-23	Novel-mir-203-5p	-	TCAGTTTGCGGAAGAAGTAGAGG	-
Novel-mir-204-1	CTGGCTCTACTCAGTTTGCGGAGAAAGTAGAGGCATGTTTGAGCTTTCTTGGCCAGTGATGTGGTGTTGTTGGT	-21.7	Novel-mir-204-5p	Novel-mir-204-3p	TCAGTTTGCGGAGAAAGTAGAGGC	TGAGCTTTCTTGGCCAGTGATGTGG
Novel-mir-204-2	CTGGGTCTCCTCAGTTTGCGGAGAAAGTAGAGGCGTGATTGAGCCTTCTTGGCCAGTGATGTGGTGTTGTTCATCCAGGAGAGGTCCTCAGA	-29.5	Novel-mir-204-5p	-	TCAGTTTGCGGAGAAAGTAGAGGC	-
Novel-mir-205-1	GTGAGCTGGGTCAGTTTGGAATGGAGGATGTCCGGGATTATGGCGTTGAACACCAAGCTGAAGTCCACA	-23	Novel-mir-205-5p	-	TCAGTTTGGAATGGAGGATGTC	-

Novel-mir-205-2	GTGAGCTGGGTCAGTTTGGAATGGAGGATGTCTGGTATGATGGTGTTGAATGCCAAGCTGAAGTCCACA	-20.7	Novel-mir-205-5p	Novel-mir-205-3p	TCAGTTTGGAATGGAGGATGTC	TGGTGTTGAATGCCAAGCTGAAG
Novel-mir-206-1	GTGAGCTGGGTCAGTTTGGAGTAGAGGATGTCCAGGATGATGGTGTTGAACGCTGAGCTGAAGTCCACA	-19.2	Novel-mir-206-5p	-	TCAGTTTGGAGTAGAGGATGTCCA	-
Novel-mir-206-2	GTGAGCTGGGTCAGTTTGGAGTAGAGGATGTCCAAGATGCTGGTGTTGAATGCTGAGCTGAAGTCCACA	-18	Novel-mir-206-5p	-	TCAGTTTGGAGTAGAGGATGTCCA	-
Novel-mir-207	GTGAGCAGGGTCAGTTTGGAGTGGAGGATGAGTGGGATGATGGGCCCATGCATAGGTCCCTGGGGAGTTGAGGTGTTGCAGGATG	-20.2	Novel-mir-207-5p	Novel-mir-207-3p	TCAGTTTGGAGTGGAGGATGA	TAGGTCCCTGGGGAGTTGAGGTG
Novel-mir-208	GTGAGCTGGGTCAGTTTGGAGTGGAGGATGTCAGGGATGATGGTGTTGAATGCCAAGCTGAGGTCCACAAAGAGG	-22.8	Novel-mir-208-5p	Novel-mir-208-3p	TCAGTTTGGAGTGGAGGATGTCA	ATGGTGTTGAATGCCAAGCTGAGGTC
Novel-mir-209	GTGAGCCGGGTCAGTTTGGAGTGGAGGGTGTCTGGGATGATGGTGTTGAATGCCAAGATGAAATCCACAAAGAAGGCCCCCTGCATAG	-24.5	Novel-mir-209-5p	-	TCAGTTTGGAGTGGAGGGTGTCTG	-
Novel-mir-210	GCCTATAGCATCATAACTAAGGGAAGGCTCAGGGTCACCTGTAGTAGCCCTAACTATAAGCTTTAGCA	-19.4	Novel-mir-210-5p	-	TCATAACTAAGGGAAGGCTCAGG	-
Novel-mir-211-1	AGAATCCGACTCATAAGGTGGTGCGGTCGGCTGTGGAGCAAAGGGTTGCTGCTGTGCATATAGCTGTACAGCATTCTGCACCGGCATTTGGTTAGTTGTAG	-35.7	Novel-mir-211-5p	-	TCATAAGGTGGTGCGGTCGGCTGT	-
Novel-mir-211-2	AGAATCCCATTCATAAGGTGGTGCGGTCGGCTGTGGAGCAAAGGGTTGCTGCTGTGCATATAGCTGTGCAGCATTCTGCACAGACATTTGATTAGCTGTAG	-28.5	Novel-mir-211-5p	-	TCATAAGGTGGTGCGGTCGGCTGT	-
Novel-mir-211-3	AGAATCTGACTCATAAGGTGGTGCGGTCGGCTGTGGAGCAAAGGGTTGCTGCTCTGCATATAGCTGTGCAGCATTCTG	-23.2	Novel-mir-211-5p	-	TCATAAGGTGGTGCGGTCGGCTGT	-
Novel-mir-211	AGAATCTGACTCATAAGGTGGTGCGGTCGGCTGTGGAGCAAAGGGTTGCTGCTGTGCATATAGCTGTGCAGCATTCTGCACAGACATTTGATTAGCTTTA	-31.5	Novel-mir-211-5p	-	TCATAAGGTGGTGCGGTCGGCTGT	-
Novel-mir-212-1	TTCAAAAACGTCATAGCTTGCAGCACTCTTTGAGAGCTTCAAAGTAAGGAAGCAGGTTGAGGCATGTGGTAT	-18.7	Novel-mir-212-5p	Novel-mir-212-3p	TCATAGCTTGCAGCACTCTTTGA	AAAGTAAGGAAGCAGGTTGAGGCA
Novel-mir-212-2	TTCAAAAACGTCATAGCTTGCAGCACTCTTTGAGAGCTTCAAAGTAAGGAAGCAGGTTGAGGCATGTGGTATT	-18.7	Novel-mir-212-5p	Novel-mir-212-3p	TCATAGCTTGCAGCACTCTTTGA	CAAAGTAAGGAAGCAGGTTGAGGC
Novel-mir-213-1	ACAAACAGAGTCATATCAGGATCTGGGATAGGCTCAGCTGCCAGGTCTATTCTGATTTTTATATCTCTCTCCGCTTTTTTC	-20	Novel-mir-213-5p	Novel-mir-213-3p	TCATATCAGGATCTGGGATAGGC	TCTATTCTGATTTTTATATCTCTCTC
Novel-mir-213-2	ACAAACAGAGTCATATCAGGATCTGGGATAGGCTCAGCTGCCAGGTCTATTCTGATTTTTATATCTCTCTCTGCTTTT	-21.2	Novel-mir-213-5p	Novel-mir-213-3p	TCATATCAGGATCTGGGATAGGC	CTATTCTGATTTTTATATCTCT
Novel-mir-214	ATCAGGACCATCATCAAGAAGGCACAGGAGAGGATTTACTTCCAGTGCCAGCTCAGGAAGTTTGACCTGCC	-18.7	Novel-mir-214-5p	-	TCATCAAGAAGGCACAGGAGAGG	-
Novel-mir-215-1	TCTATGTAAATCATGTTATCAAATGAACCTCCTGGGGTGATGCTTCGTTTTGCTCTATGCCAAGGGTGGTCTTTTATTAGTTGATCTAAGTTAGAG	-19.8	Novel-mir-215-5p	-	TCATGTTATCAAATGAACCTCCTG	-
Novel-mir-215-2	TCTATGTAAATCATGTTATCAAATGAACCTCCTGGTGTGATGCTTCGTTTTGCTCTATGCCAAGGGTGGTCTTTTATTAGTTGATCTAGGTTGGAG	-20.6	Novel-mir-215-5p	-	TCATGTTATCAAATGAACCTCCTG	-
Novel-mir-216	ACATGAGCTTTCATTTGAGAATATGAGCAGAGTTCTTTTGCAACTTGTGTTTTAGCAATGAAAAATGTGTTAAGATTTATGAGAATGGAGGATTGTGTTTT	-22.2	Novel-mir-216-5p	Novel-mir-216-3p	TCATTTGAGAATATGAGCAGAGT	TGTGTTAAGATTTATGAGAATGGAGG
Novel-mir-217	CTGAGCTGATTCCAAAAGGCAGTCTGTTGTACACAAAAAGGCCACGGTGTGTGTTTATAGTTGTGTACTTTTTTGAGTCCTCGTCCA	-19.1	Novel-mir-217-5p	Novel-mir-217-3p	TCCAAAAGGCAGTCTGTTGTACACA	TTATAGTTGTGTACTTTTTTGAG
Novel-mir-218-1	GGCCGGGGATTCCAATATCTGGTAGACTGGGAGGGTTATGGTCCAGAAGAGCAGTCCTGGATTCCCCGCTCCT	-27.9	Novel-mir-218-5p	-	TCCAATATCTGGTAGACTGGGAGG	-
Novel-mir-218-2	GGCTGGGAATTCCAATATCTGGTAGACTGGGAGGGTTATAGTCCAGAGGAGCGGGCCTGGATTCCCTGCTC	-24.2	Novel-mir-218-5p	-	TCCAATATCTGGTAGACTGGGAGG	-
Novel-mir-219	GCTGCCCACCTCCACTGTAAACAGTTTGGCAGGGCACTGCTTCTCCCTCCTGAGGCGCCGTGTCCATAAAGTTCTCTCAGTGTGCAAATCTCAGCT	-28.24	Novel-mir-219-5p	-	TCCACTGTAAACAGTTTGGCAGGG	-
Novel-mir-220	GCCAGGCACTTCCAGTCAGAGGAGTGCAGTTGGCTCAGCCAGTATGCAAAATTTGCTTAAGGAGTGTAATCC	-20.8	Novel-mir-220-5p	-	TCCAGTCAGAGGAGTGCAGTTGGC	-
Novel-mir-221	NNNGCCTAGGTCCATCGGTTCCTCTGGAGTGTGGCGGTTGCTGGTGTTGGTGGCCCCCAAACGGAGAGGAACCCGGGCCTCGACTGGTGGT	-41.3	Novel-mir-221-5p	-	TCCATCGGTTCCTCTGGAGTGTGG	-
Novel-mir-222	CTTGGTGTCATCCATGTAGAGGAGGTGGCTAATGGTTGCTCCATTTCGTAGTCAGTATTCGTAACTGCTCTTGGTGATGATCTGGCTGAGG	-24	Novel-mir-222-5p	-	TCCATGTAGAGGAGGTGGCTAATG	-
Novel-mir-223	CTTGATGTCATCCATGTAGAGGAGGTGGCTGATAGTTGTTCCATTCCATAATTGGTATCCGTAACCACTCTTGGTGATGATCAGT	-21	Novel-mir-223-5p	-	TCCATGTAGAGGAGGTGGCTGATA	-
Novel-mir-224	GCCCTCTAAATCCATTGTAGGCCAGGGCACATACTGTAGCTGTGCTCCTTTGGCCAGCAGTGGAAATGATGTGGCC	-34	Novel-mir-224-5p	-	TCCATTGTAGGCCAGGGCACATAC	-

Novel-mir-225	AACCACAGTATCCATTGTCAGCAGAAGCAATAGCAGGTATTAAGCCTGTGATTTCATCATTGTTGGAACAGGGTGTTTTGGTTC	-24.4	Novel-mir-225-5p	-	TCCATTGTCAGCAGAAGCAATAGC	-
Novel-mir-226	ACGACCCTGTTCCGAGAGGTCTGGTTCTGAAGGGTGGTCAGAGCCCCCTTCACCACCTCCTCCTGCAGGCCAGC	-31	Novel-mir-226-5p	Novel-mir-226-3p	TCCGAGAGGTCTGGTTCTGAAGGG	CTTCACCACCTCCTCCTGCAG
Novel-mir-227-1	CACCAAGGCATCCGCGACTGTCTCTGCCTCCTGGTCTGGTATGGCGTATGCCTCCGGCCACTTTGTAAAATAGTCCATGGCGGCCAGGACATAGCG	-29.9	Novel-mir-227-5p	Novel-mir-227-3p	TCCGCGACTGTCTCTGCCTCCTG	TTGTAAAATAGTCCATGGCGGCCA
Novel-mir-227-2	CACCAAGGCATCCGCGACTGTCTCTGCCTCCTGGTCTGGTATGGCGTATGCCTCCGGCCACTTTGTAAAATAGTCCATGGCGGCCAGGACATAGC	-28.3	Novel-mir-227-5p	Novel-mir-227-3p	TCCGCGACTGTCTCTGCCTCCTG	TTGTAAAATAGTCCATGGCGGCC
Novel-mir-227-3	CACCAAGGCATCCGCGACTGTCTCTGCCTCCTGGTCTGGTATGGCGTATGCCTCCGGCCATTTTGTAAAATAGTCCATGGCGGCCAGGACATAGC	-28.3	Novel-mir-227-5p	Novel-mir-227-3p	TCCGCGACTGTCTCTGCCTCCTG	TTGTAAAATAGTCCATGGCGGCC
Novel-mir-228	CACGACCCGGTCCGGATAAGTGGAAGAAGATGGATGGATGTGAAGTCCCAGACTTCTTTTCGAGTCCAGATAGTGGAAAGA	-20.1	Novel-mir-228-5p	-	TCCGGATAAGTGGAAGAAGATGGA	-
Novel-mir-229	CTGAACCTCTTCCTGAAGTACTGGTCCTTCTGAGGGTCAGTGAACCCTCTGACCTCTGTCACCATGTCCACACACTCAGGAGGGATCTG	-27.5	Novel-mir-229-5p	Novel-mir-229-3p	TCCTGAAGTACTGGTCCTTCTGAG	TCTGTCACCATGTCCACACACTCAG
Novel-mir-230	CTCATGGGACTCCTGAGACAGCTGACGGGTACTAGCAGGCCAAGCGGGCTGCAGCCCAGGCAGTTGCGGAGG	-28.2	Novel-mir-230-5p	-	TCCTGAGACAGCTGACGGGTACT	-
Novel-mir-231-1	ATTTTCCAGCTCCTGGTTTAGCCGTTCAGCTTGGCCGTTGGTTTGGGGGTGATAGCTGGAAGAGAGACAGGGACGTGCTCCGAGG	-23.1	Novel-mir-231-5p	Novel-mir-231-3p	TCCTGGTTTAGCCGTTCAGCTTGG	TGATAGCTGGAAGAGAGACAGGGACG
Novel-mir-231-2	ATTTTCCTGTTCCTGGTTTAGCCGTTCAGCTTGGCCGTTGGTTTGCGGGTGATAAGCGGAAGAGAGACAGGGACGTGCTCCGAGG	-24.2	Novel-mir-231-5p	Novel-mir-231-3p	TCCTGGTTTAGCCGTTCAGCTTGG	TGATAAGCGGAAGAGAGACAGGGACG
Novel-mir-232	TGCACCTCCCTCCTGTAGGCTGCTTTGTCCCCACTCTTTGTGGGAGGCATGCGTAAACAGGGTGTAGATCATGGGACTGAGGACACATCCCTGTGGGGTGC	-41.2	Novel-mir-232-5p	-	TCCTGTAGGCTGCTTTGTCCCCAC	-
Novel-mir-233	TGTCTGAGCTTCCTGTTACCTGCCTCCCTAGGGTGCAGTGGTTTTTGTTTGGCATGTCTTTTTGAGGATTGGGTGTAGGCTGTTTGAGCT	-22.3	Novel-mir-233-5p	Novel-mir-233-3p	TCCTGTTACCTGCCTCCCTAGGGT	TTTTTGAGGATTGGGTGTAGGC
Novel-mir-234	AGATAAAACATGTCCTTCAGTTCACACAAGCAGTGTCTCACAGGTCCCTGTGGTCTGTAGTCTGTTCTGAAAGGTTAAATTCCATTG	-20.4	Novel-mir-234-5p	Novel-mir-234-3p	TCCTTCAGTTCACACAAGCAGTGT	TCTGTAGTCTGTTCTGAAAGGTTA
Novel-mir-235-1	TTGACTGGGTTCGAAATGCAACAGAGTAGGCGTCAGTTCGCGCCTCGTGTGTTGTATGTGCTGGACCCCGCCCT	-28.8	Novel-mir-235-5p	-	TCGAAATGCAACAGAGTAGGCGTC	-
Novel-mir-235-2	TTGATTGGGTTCGAAATGCAACAGAGTAGGCGTCAGTTCACGCCTTGTGTGTTGTGTGTGCTGGACCCCGTCCT	-30.4	Novel-mir-235-5p	-	TCGAAATGCAACAGAGTAGGCGTC	-
Novel-mir-236	CCCTGGACTGTCGAAGCAACAGACGGTCCTCTCGAACAGTTGTCTTATGAGGTCTGCCTGACCTGGGCCTGTCATGAACTTCACCAGTTTCTTC	-26.9	Novel-mir-236-5p	-	TCGAAGCAACAGACGGTCCTCTC	-
Novel-mir-237	CAAGGACACCTCGACATGTTGACTGGACAAGCGGCGAATCGATCCGCTAATCCTCCGGTTAGGAAACGACCCGCTCTAC	-18.2	Novel-mir-237-5p	-	TCGACATGTTGACTGGACAAGCGG	-
Novel-mir-238	CCTGGGCTGGTCGAGGTAGGGATGATGGTGGCTGAGGAGACAGGAGCATGTACAAACAGTTAAAGGAGACTGTGCATTGACCTGTGTAGT	-26	Novel-mir-238-5p	Novel-mir-238-3p	TCGAGGTAGGGATGATGGTGGCTG	TTAAAGGAGACTGTGCATTGA
Novel-mir-239	CCTGGACTGGTCGAGTTGGATGTTACTGAAGATGAACAGAAATAGTACATACGGTAAGTAGTAGACTGTGCATTGACCTGCAGGGTGGTG	-20.2	Novel-mir-239-5p	Novel-mir-239-3p	TCGAGTTGGATGTTACTGAAGATG	TAAGTAGTAGACTGTGCATTGACCTG
Novel-mir-240	CTGGCTCCTCTCGATGTGGAGGAGCAGCGGCTCTACTCTGAGCTCCTCCCGGGTGACTGAGCTCC	-24.6	Novel-mir-240-5p	-	TCGATGTGGAGGAGCAGCGGC	-
Novel-mir-241	GTGGGGAAAATCGATTTGAATTGTGAACAGGGGTGATTCACCATGGGCATTTGTTTCAAGCTATGATTCTTGCA	-19.6	Novel-mir-241-5p	-	TCGATTTGAATTGTGAACAGGGGT	-
Novel-mir-242	CTGCAGGAGATCGCAAATGACGGTGACCTTAAACGTACGTTTGAGACAACAACTCTGCCGATGTTCTGGAT	-18.7	Novel-mir-242-5p	Novel-mir-242-3p	TCGCAAATGACGGTGACCTTAAAC	TGTATTCCTTTGATGCTATTATT
Novel-mir-243	CTTTACAATGTCGGAAAACAGTCTGAGCATAGCTCCGTCCAGCTCGTCGTCAGTTTGCCCTTCGGGCTCCTCCTCACAGATCGTGTCGAGA	-19.5	Novel-mir-243-5p	-	TCGGAAAACAGTCTGAGCATA	-
Novel-mir-244	TGGTTCTCAGTCGGAAAAGGATGGCTTGTTCCCTTCAGGTTGGTGGAGAGTTCCTGCCTCGAGTGGAGGAGTT	-19.3	Novel-mir-244-5p	-	TCGGAAAAGGATGGCTTGTTCCC	-
Novel-mir-245	ACAGCATGAGTCGGAGGAAAGGGAGGCTGTGGCTGTCTTCTGTCTCCGCTTCTTCTTCCTGTACATGTAGA	-24.5	Novel-mir-245-5p	-	TCGGAGGAAAGGGAGGCTGTGG	-
Novel-mir-246	GTGTCGAGCTTCGGATGGACACAGTCAGTGGTGGAGATGAGAAGAGGACCGGGCTGTGGACACTGGGGCAGGCGA	-29.16	Novel-mir-246-5p	-	TCGGATGGACACAGTCAGTGGTGG	-
Novel-mir-247	ATCCCAAGGATCGTTGTCCAGGCTGCAGTTGGTGCAAAATGGCGCCGCCTGGCTGCTCACTGACACCCGTCGACATGCT	-23.7	Novel-mir-247-5p	-	TCGTTGTCCAGGCTGCAGTTGGTG	-
Novel-mir-248	TTGTCCAAAGTCGTTTCCCAGGAGTCGCTCAGGCGGTGCCAGGTCGGTGCGAGGAAGGCGATTCCTAAGCTGCTGAGTAAGA	-24.6	Novel-mir-248-5p	-	TCGTTTCCCAGGAGTCGCTCAGG	-

Novel-mir-249	ACTGTAAACATCTACACTGATTCTGCCTATGCAGTACAAGCAATGCATGTGGACCTGTGTCACTGGAGAAGAAAAGGTTTTGTGACCTCTGCAGG	-23.1	Novel-mir-249-5p	-	TCTACACTGATTCTGCCTATGCAG	-
Novel-mir-250	GTAACCTAAGTCTACAGGAAGAGGGTCGCCAGACGTATTCTGGCCAAACACTATAACCTCAGATTTAGCATCA	-19.3	Novel-mir-250-5p	-	TCTACAGGAAGAGGGTCGCCAGAC	-
Novel-mir-251	ATGCAAATCTTCTAGAATGTTCGAATAAGCAGCATGTGTGCGTATGTGTTGGTATTTTCAGGCGTTTCTGAAAGTAGACATGCAG	-22.1	Novel-mir-251-5p	-	TCTAGAATGTTCGAATAAGCAGC	-
Novel-mir-252	CCTCATTTCTTCTAGGAGTAGACACCTTACTGGCCACAAGCGGAGGGTTGAGGGAGTATTCTCTTGAGTCAAAGGGGT	-21.3	Novel-mir-252-5p	-	TCTAGGAGTAGACACCTTACTGGC	-
Novel-mir-253	CAGGTTGGACTCTAGTTGGAAACCGCAGTCTCAATGCTGTCCACAAGGCGTTGCGATGCCGGTTGAAAATGTCACCATCATGGGCT	-23	Novel-mir-253-5p	Novel-mir-253-3p	TCTAGTTGGAAACCGCAGTCTCAA	TGCGATGCCGGTTGAAAATGTCACC
Novel-mir-254	GGGCGTATACTCTATCTGGTGGCCCATCCCCTGGATCAGGTTCCTGTTGCTGCGCCTCCCATATGGTGTCCTCAA	-18.4	Novel-mir-254-5p	Novel-mir-254-3p	TCTATCTGGTGGCCCATCCCCTGG	TCCTGTTGCTGCGCCTCCCATATG
Novel-mir-255	CAGCGCCTCGTCTATGTTGCAAGGCGCCAGGAGCCGGACATGCTGTTGGAGCCGCACTGGGGTGAGGCCCCT	-30.1	Novel-mir-255-5p	-	TCTATGTTGCAAGGCGCCAGGAGC	-
Novel-mir-256	GTGTCCCCACTCTCACTGGTGGGCCCGCTCTTTTGGCCGCACAGGACAGGCGGCGATGAGATGGTCTGGGGCCCCACAGTAGAGGCAAG	-37.2	Novel-mir-256-5p	Novel-mir-256-3p	TCTCACTGGTGGGCCCGCTCTTTTG	TGAGATGGTCTGGGGCCCCACAG
Novel-mir-257	GTGTAGCCGGTCTCATCTCTACGCGGCCATCAAGTAACATCTCTGCGTTGTGTCTTTAGTGATGAAGCGTAGACAGGGGTTTGCCATCCACA	-23.8	Novel-mir-257-5p	-	TCTCATCTCTACGCGGCCATCAA	-
Novel-mir-258	TCTGAGAATATCTCCGCAGGAGGACTGAAGCTTTGTGCAAACATCTGCCTCTGTGCCTTCTACTGGGATTTTCCCTGG	-22.3	Novel-mir-258-5p	-	TCTCCGCAGGAGGACTGAAGCT	-
Novel-mir-259-1	CTTGGCCTAATCTGAACTCCACCTCCACCCACCCCCTGAAAGGTATCGGTGTGTGGTTTGCAGCCAGTCCAAGC	-21.2	Novel-mir-259-5p	Novel-mir-259-3p	TCTGAACTCCACCTCCACCCACCC	GTATCGGTGTGTGGTTTGCAGCCA
Novel-mir-259-2	CTTGGCCTAATCTGAACTCCACCTCCACCCACCCCCTGAAAGGTATCGGTGTGTGGTTTGCAGCCAGTCCAAT	-18.9	Novel-mir-259-5p	Novel-mir-259-3p	TCTGAACTCCACCTCCACCCACCC	GTATCGGTGTGTGGTTTGCAGCCA
Novel-mir-260-1	CATTGCCTGATCTGAATGCATGAGAGCAAGCACGCAGCATGTGGCGTACCTGACTGTTTATCTGGGGCTTCTGGTTGGGAAACC	-20.3	Novel-mir-260-5p	-	TCTGAATGCATGAGAGCAAGCACG	-
Novel-mir-260-2	CATTGCCTGATCTGAATGCATGAGAGCAAGCACGTAGCATGTGGCATACCTGACTGTTTATCCAGGGCTTCTGGTTGGGGAGCC	-21.6	Novel-mir-260-5p	-	TCTGAATGCATGAGAGCAAGCACG	-
Novel-mir-261	CATCGCCTGATCTGAATGCATGAGATGACGCAGCATGTGGCATACCTGACTTGTTTATCCAGAGCTTCTGGTTGGGGACA	-20	Novel-mir-261-5p	-	TCTGAATGCATGAGATGACGCAGC	-
Novel-mir-262	AGCTCGGCCATCTGAGCGGAGCTTGGCTGTGGGATCACTTCAGCTTGCAGCACTCTGAAGCCTGCA	-22.2	Novel-mir-262-5p	Novel-mir-262-3p	TCTGAGCGGAGCTTGGCTGTGG	TTCAGCTTGCAGCACTCTGAAG
Novel-mir-263	TTTCTTTCCATCTGATAGTTTTCTGAATTGTGCACTGGTTTGCTGTTCTCGCTCACACAAAGCCCACAGTTCATTGAGCTGTGAGAGGTCAGAGGC	-32.2	Novel-mir-263-5p	-	TCTGATAGTTTTCTGAATTGTG	-
Novel-mir-264	GGAGGAATTGTCTGGAGAAAGAGAAGTCTGGGCATCTTTGCTTAGACTGCTGCCCCCACGGCCCGGTCCCG	-25.5	Novel-mir-264-5p	-	TCTGGAGAAAGAGAAGTCTGGGCA	-
Novel-mir-265-1	GGAGGAAGTGTCTGGAGAGAGGGAAGTCTTGGCATCCTTGCTTAGACTTCTGGCCCCGCAACCCGATTCCC	-23.6	Novel-mir-265-5p	-	TCTGGAGAGAGGGAAGTCTTGGC	-
Novel-mir-265-2	GGAGGAAGTGTCTGGAGAGAGGGAAGTCTTGGCAGCTTTGCTTAGACTGCTCCCCCAGCGACCTGGTTCCG	-25.7	Novel-mir-265-5p	-	TCTGGAGAGAGGGAAGTCTTGGC	-
Novel-mir-255-3	GATGAAGCTGTCTGTAAGTCTGGTGGAGTGGGCCTGGAGACTCCGATACCTTCTCCCTGAAGGCAGGAGTCT	-20.6	Novel-mir-255-3p	-	TCTGTAAGTCTGGTGGAGTG	-
Novel-mir-266-1	CAACCTCCCATCTGTAGGCTGACTCATCATCGTCTCGGATGAGACCAATGACCGCTGTGTCATCTGCAAACTTTAGGAGTTTGA	-24.5	Novel-mir-266-5p	-	TCTGTAGGCTGACTCATCATC	-
Novel-mir-266-2	CACCCTCCCATCTGTAGGCTGACTCATCATCATCTCGGATGAGACCAGTGACCGTTGTGTCATCTGCAAACTTTAGGAGCTTGA	-20.5	Novel-mir-266-5p	-	TCTGTAGGCTGACTCATCATC	-
Novel-mir-267	TTGAGGTCTGTCTGTGGAACTGATGATGCTGGTGGTGGTCCTGTAGTCTGTGATGTGCTGCAGGCCTAGTCACATCCGTCCAGAGTCAGCAGTAGA	-32.1	Novel-mir-267-5p	-	TCTGTGGAACTGATGATGCTGGTG	-
Novel-mir-268	CGGCAGGCTGTCTGTGGAGCTTATGATGCTGGTGGTGGTCCTGTAGTCTGTGATGTGCTGCAGGCCTTGCCA	-26.3	Novel-mir-268-5p	-	TCTGTGGAGCTTATGATGCTGGTG	-
Novel-mir-269	GTTTCAATATGTCTGACTTTGTGTTCATCATTGACCTCTCCAGTACCCCCATCTATGATGTAGAGCTCTGTGTAGATCTGATTCAGAAATGTTGGGTT	-18.99	Novel-mir-269-5p	-	TCTGTGTAGATCTGATTCAGAA	-
Novel-mir-270	GTCCTCATCTTCTGTGTCTGAATTGAACAGCTGGGAAATTTCGCCATCAAACATGCTGGGTTCCCTCTCATCATTGTCGGAGTCAGTCTCGTTGC	-18.3	Novel-mir-270-5p	Novel-mir-270-3p	TCTGTGTCTGAATTGAACAGCTGGG	CCCTCTCATCATTGTCGGAGTCA
Novel-mir-271	GTGATTAAATTCTGTTAGGAGCGCAATGAGACGGCAGGCCACAAACCTTTGTCTCTCGTCTTTTGTTGTATTAAACAGGATCATTAATCTG	-25.4	Novel-mir-271-5p	-	TCTGTTAGGAGCGCAATGAGACGG	-

Novel-mir-272-1	TGGTGAGCCTTCTTAACCAGGCTGTGGGTGTTGGTAGTCCAGGACAGATCCTCTGAGATGTGGGTCCCCAG	-20.2	Novel-mir-272-5p	Novel-mir-272-3p	TCTTAACCAGGCTGTGGGTGTTGG	AGGACAGATCCTCTGAGATG
Novel-mir-272-2	TGGTGAGCCTTCTTAACCAGGCTGTGGGTGTTGGTGGTCCAGGACACATGCTCTGAGATGTGGGTCCCCAG	-22.7	Novel-mir-272-5p	-	TCTTAACCAGGCTGTGGGTGTTGG	-
Novel-mir-272-3	TGGTGAGCCTTCTTAACCAGGCTGTGGGTGTTGGTGGTCCAGGACAGATCCTCTGAGATGTGGGTCCCAAGGAA	-20.2	Novel-mir-272-5p	Novel-mir-272-3p	TCTTAACCAGGCTGTGGGTGTTGG	CAGGACAGATCCTCTGAGATGTGGG
Novel-mir-272-4	TGGTGAGCCTTCTTAACCAGGCTGTGGGTGTTGGTGGTCCAGGACAGATCCTCTGAGATGTGGGTCCCCAG	-20.2	Novel-mir-272-5p	Novel-mir-272-3p	TCTTAACCAGGCTGTGGGTGTTGG	AGGACAGATCCTCTGAGATG
Novel-mir-273	TGCCAAACTTTCTTCAAACAGGGTCTCATTTGTGACATCTCCATCTCGGTCATAGAAGAGGGTGACGTGGGGAGGATCAGGAG	-19.3	Novel-mir-273-5p	Novel-mir-273-3p	TCTTCAAACAGGGTCTCATTTGTGA	TCATAGAAGAGGGTGACGTGGGGA
Novel-mir-274-1	GCAAGCCTGTTCTTCAGGGGTCAGACGGGCGTGCCCTAATTCCATGGGTTCATCCGTAGGTCTCTGGAAGGATGTGTCGGT	-29.9	Novel-mir-274-5p	Novel-mir-274-3p	TCTTCAGGGGTCAGACGGGCGTGC	TCATCCGTAGGTCTCTGGAAGG
Novel-mir-274-2	GCGGGCCCGTTCTTCAGGGGTCAGACGGGCGTGCCCTAATTCCATGGGTTCATCCGTAGGTCTCTGGAAGGATGTGTCGGT	-35.7	Novel-mir-274-5p	Novel-mir-274-3p	TCTTCAGGGGTCAGACGGGCGTGC	TCATCCGTAGGTCTCTGGAAGG
Novel-mir-273	GACAAACTGTTCTTGTAGATACAGGGGTTTCTGGTGACCAGTTATTATGTAGCTCTGCTTGAAAAAGGACTGGCCCTGGACCGTCAAAGCAACAGATGGTC	-25.7	Novel-mir-273-3p	-	TCTTGTAGATACAGGGGTTTCTGG	-
Novel-mir-275	CAATCCTGCATCTTGTCCTTGTGTAGCTGCCTGCTCAGTAAAACCGAGCTGTGAGCTGCCAGGGCTGA	-22.1	Novel-mir-275-5p	-	TCTTGTCCTTGTGTAGCTGCCTGC	-
Novel-mir-276	TGGGGCGTTGTCTTGTTGAAATGTAAACCTTTACCCCAGCCTGAGATCCTAAGCACTCTGGAGAAGGTTTTCATTCAGAATTGATCAATGTTTTT	-24.1	Novel-mir-276-5p	-	TCTTGTTGAAATGTAAACCTTT	-
Novel-mir-277	GGTGAACCGGTCTTTGAGGGTTTGGAAGGCCTGCTCTGCTGTCCTGGACCATGCAAACCTAACCTTGGTAGACGTGAGCTG	-22.2	Novel-mir-277-5p	-	TCTTTGAGGGTTTGGAAGGCCTG	-
Novel-mir-278	GGGGGTTTAGTCTTTTGGCGGACTGGAGGTAGGCCAAGTTCTTATGATTGGTCCAGACTAGAAAAGGTTGGGCAGCAC	-19.1	Novel-mir-278-5p	Novel-mir-278-3p	TCTTTTGGCGGACTGGAGGTAGGC	TATGATTGGTCCAGACTAGAAAAGGT
Novel-mir-279	TTCCTGCTGCTGAAAAACATCCCCACGGCATGATGCTGCCACCACCATGCTTCACTGTAGGGATGGTATTGGCCAGGTGGTGA	-25.6	Novel-mir-279-5p	Novel-mir-279-3p	TGAAAAACATCCCCACGGCATGA	TGCTTCACTGTAGGGATGGTATTGGC
Novel-mir-280	CGCCAAGAAATGAAAACATCTCTTGCAGATATGGCCACGGGTGGTTATCTGAGTCTGCTGGAGACGTAGGTGTGAGTGGC	-28.2	Novel-mir-280-5p	Novel-mir-280-3p	TGAAAACATCTCTTGCAGATATGGC	TCTGAGTCTGCTGGAGACGTAGG
Novel-mir-281	TTTCAAGAGCTGAACTATTCTGCAACATTTGTTGATGTGGTTCTGCAAGTAAATGCTGCAGAATTGTGCTCCTCTTGGAAT	-27.6	Novel-mir-281-5p	Novel-mir-281-3p	TGAACTATTCTGCAACATTTGT	TAAATGCTGCAGAATTGTGCTC
Novel-mir-282-1	GCCATCAAGCTGAAGAAGGAGCTCTATCGGGCCCGGTTGGCTCATGGGACTCCTGAAGCAGCTGGCAGGTACCGAC	-32.6	Novel-mir-282-5p	Novel-mir-282-3p	TGAAGAAGGAGCTCTATCGGGC	CTCATGGGACTCCTGAAGCAGCTGGC
Novel-mir-282-2	GCCATCAAGCTGAAGAAGGAGCTCTATCGGGCCTGGTTGGCTCATGGGACTCCTGAAGCAGCCGTCAGGTA	-30.9	Novel-mir-282-5p	-	TGAAGAAGGAGCTCTATCGGGC	-
Novel-mir-282-3	GCCGTCAAGCTGAAGAAGGAGCTCTATCGGGCCTGGTTGGCTCATGGGACTCCTGAAGCAGCTTGCAGGTA	-37.3	Novel-mir-282-5p	-	TGAAGAAGGAGCTCTATCGGGC	-
Novel-mir-282-4	GCCGTCAAGCTGAAGAAGGAGCTCTATCGGGCCTGGTTGGCTTATGGGACTCCTGAAGCAGCTGGCAGGTA	-34.3	Novel-mir-282-5p	Novel-mir-282-3p	TGAAGAAGGAGCTCTATCGGGC	TTATGGGACTCCTGAAGCAGC
Novel-mir-282-5	GCTGTCAAGCTGAAGAAGGAGCTCTATCGGGCCTGGTTGGCTCATAGAACTACTGAAGCAGCTGGCAGG	-30.8	Novel-mir-282-5p	-	TGAAGAAGGAGCTCTATCGGGC	-
Novel-mir-282-6	GCTGTCAAGCTGAAGAAGGAGCTCTATCGGGCCTGGTTGGCTCATGGGACTGCTGAAGCAGCTGGCAGG	-32.8	Novel-mir-282-5p	-	TGAAGAAGGAGCTCTATCGGGC	-
Novel-mir-282-7	GCTGTCAAGCTGAAGAAGGAGCTCTATCGGGCCTGGTTGGCTCATGGGACTTCTGAAGCAACTGGCATGTA	-26.3	Novel-mir-282-5p	-	TGAAGAAGGAGCTCTATCGGGC	-
Novel-mir-282-8	GCTGTCAAGCTGAAGAAGGAGCTCTATCGGGCTTGGTTGGCTCATGGGACTCTTGAAGCAGCTAGCAGG	-29	Novel-mir-282-5p	-	TGAAGAAGGAGCTCTATCGGGC	-
Novel-mir-282-9	GTAGTCAAGCTGAAGAAGGAGCTCTATCGGGCCTGGTTGGCTCATGAGACTCCTGAAGCAGCTGGCAGG	-31.5	Novel-mir-282-5p	-	TGAAGAAGGAGCTCTATCGGGC	-
Novel-mir-282-10	GTTGTCAAGCTGAAGAAGGAGCTCTATCGGGCCTGGTTGGCTCATGGGACTCCTGAAGCAGCTGGCAGG	-35	Novel-mir-282-5p	Novel-mir-282-3p	TGAAGAAGGAGCTCTATCGGGC	TCATGGGACTCCTGAAGCAGC
Novel-mir-283	TCAGTCGTGGTGAAGAAGGAGCTGAGCTGTAAGACGAGTCTCTCGATTTACTGGTAGCTCTGTATTTACGGGTCGATCTA	-24.6	Novel-mir-283-5p	-	TGAAGAAGGAGCTGAGCTGTAAGA	-
Novel-mir-284	TCTGTCGTGGTGAAGAAGGAGCTGGACTGTAAGGTGAAGCTGTCAATTTACTGGTCGATCTACGTTCCTACTCTCACCTATGGTCATGAG	-29	Novel-mir-284-5p	-	TGAAGAAGGAGCTGGACTGTAAGG	-
Novel-mir-285	CACAGGCAGATGAAGAATGTAAGACCCCGAACCCAACAGGCCTGTACATTATATCTGGTTTGGGCTAATCCCATTCTACACAGGCACGTC	-19.9	Novel-mir-285-5p	-	TGAAGAATGTAAGACCCCGAACC	-

Novel-mir-286-1	TCCAGCTTCATGAAGAGGCTGTGAACCATACTTCTGTTGTGTGGATAGGGGCCTCAGATAGAGACGGAA	-23.9	Novel-mir-286-5p	-	TGAAGAGGCTGTGAACCATACT	-
Novel-mir-286-2	TCCGGCTTCCTGAAGAGGCTGTGAACCATACTTCTGTAGTGTGGATAGGCCTTCAGATAGACGGAA	-28.4	Novel-mir-286-5p	-	TGAAGAGGCTGTGAACCATACT	-
Novel-mir-286-3	TCCGGCTTCCTGAAGAGGCTGTGAACCATACTTCTGTAGTGTGGATAGGGGCTTCAGATAGATGGAA	-24.2	Novel-mir-286-5p	-	TGAAGAGGCTGTGAACCATACT	-
Novel-mir-286-4	TCCGGCTTCCTGAAGAGGCTGTGAACCATACTTCTGTGTGTGGATAGGGTTTCAGATAGAGACGGAA	-22.6	Novel-mir-286-5p	-	TGAAGAGGCTGTGAACCATACT	-
Novel-mir-286-5	TCCGGCTTCCTGAAGAGGCTGTGAACCATACTTTTGTAGTGTGGATAGGGGCTTCAGATAGACGGAA	-25.9	Novel-mir-286-5p	-	TGAAGAGGCTGTGAACCATACT	-
Novel-mir-287	GAAGTGCCGATGAAGAGTCCTTGTGAGTCTGCAGCCTCTGGCAAGTGACTGAGGAGTGAAACTGGGTAATATAGCACTAA	-20.1	Novel-mir-287-5p	-	TGAAGAGTCCTTGTGAGTCTGCAG	-
Novel-mir-288	GAAGGCCACATGAAGTTTGAAGGTCTGCAGCTATTGACTCTGCAGAAAGTTGGCAGCCTCTGTGCACTATGTGTCCCAGCAGCCACT	-21.3	Novel-mir-288-5p	-	TGAAGTTTGAAGGTCTGCAGCT	-
Novel-mir-289	AAAAACAGGGTGAATACGGAGGGAATCAGGGAGCTTGAGTCTGATGGCAGTGGGGTGAATGATTTCCTGGTTGATGAAGGGCCCTAGGAACCGGGGCAAG	-25.2	Novel-mir-289-5p	-	TGAATACGGAGGGAATCAGGGAGC	-
Novel-mir-290	GCTGATAGAGTGACTCACTTATTGTGGAATACATCGTATAACGTTGCACTGCTTTGTTCCACTGAATAAGTGTACGCTTTAAATGGTA	-24.8	Novel-mir-290-5p	-	TGACTCACTTATTGTGGAAT	-
Novel-mir-297	AAAAGACACGTGACTGATGACGTCCGACGCAGAAACTGAGTGACTCCTGTGACAGCTCCGTTGATTGATGTCACAACATGAGGTCTTGC	-23.9	Novel-mir-297-3p	-	TGACTGATGACGTCCGACGCAGAA	-
Novel-mir-291	TAAAAGAAATTGACTTGAAGATCTGAGGGACCTTTTAGGTTTGTGCCAATGCACTACTTCAGTGAGGTAAAATGGGGTCAAACAGTAAATATCATTTG	-21.1	Novel-mir-291-5p	-	TGACTTGAAGATCTGAGGGACCTT	-
Novel-mir-292	GAGAGGGGAGTGAGAAAGTCGGTGCAGCCGGGCAAACCTGACATGACCGTGTTTTTACTGATGCAGCCCCATAA	-23.91	Novel-mir-292-5p	-	TGAGAAAGTCGGTGCAGCCGGG	-
Novel-mir-293	CGTCTAGGGATGAGAAGATTTCGGCCCCTGCTAGCTTTGCCAGGACTTCATCTGTCATTGGTAGAATGTATCGTTCTCTTTTTATTTGATCATT	-21.3	Novel-mir-293-5p	-	TGAGAAGATTTCGGCCCCTGCTAG	-
Novel-mir-294-1	TTTGCTCCGATGAGAAGGCTGGCTTGCTGAAGGCGGAAGTCGGAGGCAGAGACTTCTCTGAGCGGAGCACC	-33.4	Novel-mir-294-5p	-	TGAGAAGGCTGGCTTGCTGAAGGC	-
Novel-mir-294-2	TTTGCTCCGGTGAGAAGGCTGGCTTGCTGAAGGCGGAGGTCAGAGGCAGAGCCCTTCTCCGAGTAGAG	-25.5	Novel-mir-294-5p	-	TGAGAAGGCTGGCTTGCTGAAGGC	-
Novel-mir-295	TCAACAATGGTGAGAATGACTGCGTTGCCTTGGGAGGGGGGAAGACCGGTGTACTCCTTCAGAGCCTGTCATTCGGATTTGGAGAT	-21.7	Novel-mir-295-5p	-	TGAGAATGACTGCGTTGCCTTGGG	-
Novel-mir-296	GGACTCCAGATGAGATAGGGCTTGATGCAGCATGTAGGTAATGGCGTCATCAACCCCAGTGCCAGGAGGGT	-18.3	Novel-mir-296-5p	-	TGAGATAGGGCTTGATGCAGCATG	-
Novel-mir-297	TGAGCAGGGGTGAGATGCAGAATGACAAGGAGTTTGTTTGACCCTCGCCTCTTTGTCCTTCCTGCTCTCATCCCTCTCATGT	-38.99	Novel-mir-297-5p	Novel-mir-297-3p	TGAGATGCAGAATGACAAGGAGT	TCTTTGTCCTTCCTGCTCTCATC
Novel-mir-298	TTGTACAGTTTGAGGGTCTGTGATTCTGCCCCATACAGGAGCTAACTGTACAAGTGACTGCCTTGCCCACGGTTGAGTCCACTCATCAGACCATGCTT	-26.8	Novel-mir-298-5p	-	TGAGGGTCTGTGATTCTGCCCCAT	-
Novel-mir-299	TGGCAGCTCCTGAGGTGTTGGTCCAGCCTGGTGCTCTTATAGACGCACCCCATGACTGGACATCGCTCATTCCTGATGTTTACGCGTCCGCTCGCCAG	-35.8	Novel-mir-299-5p	-	TGAGGTGTTGGTCCAGCCTGGTGC	-
Novel-mir-300-1	TCGCACAAGCTGGGACAGGAGCTCCTGAAGAGGAAGCTGACCATAGTGGGAACTATTCGCAGAAACAATTCTGAGCTCCCACCCCAACTTTTGATT	-31.8	Novel-mir-300-5p	-	TGAGTTGATAGTCCGGTTCTGTG	-
Novel-mir-300-2	TCGATGAACCTGAGTTGATAGTCCGGTTCTGTGAATATTATCAATTTTGAGTCCTGCATAATCAGATTGGGCTTTATCCC	-18	Novel-mir-300-5p	-	TGAGTTGATAGTCCGGTTCTGTG	-
Novel-mir-301	ATTACTTCATTGATAAAGTTTGGGCCATTAGACAGAGTATTCCTAAGACTACTCCTACTGTACCAACTGTCCCCTCTGTCCAGTTTACCCTGGAAATCT	-19.1	Novel-mir-301-5p	-	TGATAAAGTTTGGGCCATTAGACA	-
Novel-mir-302	GAGAGAAGCTTGATAGCTAAAAGCTCTGCCTCCCATGTGGACTGTGGAAATAGAACTGGCTTTTAGGTTTTCCAT	-19.8	Novel-mir-302-5p	-	TGATAGCTAAAAGCTCTGCCTCCC	-
Novel-mir-303	ACAACTGCATTGATGGTTTTGAACAATACTCTAGGCTGATTTCTATTTTTAGAGATAATGTCAGTTAGATATTGGGACTTAGCTGCGCTCAAAGCTCTT	-20.8	Novel-mir-303-5p	Novel-mir-303-3p	TGATGGTTTTGAACAATACTCTAGGC	TAGATATTGGGACTTAGCTGCGCT
Novel-mir-304	ATGCAGCAGTTGATGTTGACTTTAAGCTGGACTGTGAAGATCTACTGGATTTTTTTGATGTCTGTGATCTAGCATCACTTGCTCCAA	-23.5	Novel-mir-304-5p	-	TGATGTTGACTTTAAGCTGGACTG	-
Novel-mir-305	TGGAGACTTTTGATTTGATCCTCTTGTCTGTCCTTATTCAAATTTATGTCAGCCAGGAGGTTGATCTTCTTTTGCTGCTGCTTTACCAGGTCTTTG	-23.4	Novel-mir-305-5p	-	TGATTTGATCCTCTTGTCTGTC	-
Novel-mir-306	GTGCCTGCTCTGATTTGGAACACGAGGACAGCAGTCTCTTCTGGGTGGTCCTCAGGTCCCGAAGTAGTCTT	-20.9	Novel-mir-306-5p	-	TGATTTGGAACACGAGGACAGCAG	-

Novel-mir-307	CTGTAGAAGTTGCAAAGGATTCTAGGCGACATGCCAAACCTCCTCAGGAAGTACAGTCGCCATTGGGCCTTCTTGACCAGA	-19.4	Novel-mir-307-5p	-	TGCAAAGGATTCTAGGCGACATGC	-
Novel-mir-308	GACAGTGCACTGCAAGGCTGTGCCAGGAATTTTGGTTGATGATTCCAAGTACAACCCTGCTGTCTACTGCTCA	-26.6	Novel-mir-308-5p	Novel-mir-308-3p	TGCAAGGCTGTGCCAGGAATTT	TTCCAAGTACAACCCTGCTGT
Novel-mir-309	GTCAAACAACTGCAGCTGGACCAGGACAAGACCATTGTCTCATATGATGTCACATCCTTGTTCACTTGCATCCCCACCAC	-18.7	Novel-mir-309-5p	-	TGCAGCTGGACCAGGACAAGACC	-
Novel-mir-310	AAGTCACAACTGCAGCTGGACCAGGAGAAGATCATGGTCTCATATGATGTCACATCCTTGTTCACTTGCATCCCCACCACAGAGACTG	-22.6	Novel-mir-310-5p	-	TGCAGCTGGACCAGGAGAAGATCA	-
Novel-mir-311	GGTGTCCAGTTGCATTGAGTTTGGCTGTAGTGAGAATGTAAGTGAGCGGGTTGTTATCAGTCCTTACTGTAAACTGTACCCCA	-21.6	Novel-mir-311-5p	-	TGCATTGAGTTTGGCTGTAGTGA	-
Novel-mir-312	TCATGTACCGTGCGATAGTCAGGTTTGCCGGGCTTTGGCACTGGATTGATGGGGGTGTTCCACGGAGACCAG	-23.1	Novel-mir-312-5p	-	TGCGATAGTCAGGTTTGCCGGGC	-
Novel-mir-313	AGTAGGTGTGTGCGTAGTTGCTGTTGGTATGATCATTGCTGCTGATGTGTAATCACCATGGAGCCAGCTAGGAATACAAATGA	-19.7	Novel-mir-313-5p	Novel-mir-313-3p	TGCGTAGTTGCTGTTGGTATGATC	TCACCATGGAGCCAGCTAGGA
Novel-mir-314	ACAGGTAACCTGCGTATAGGGGACTGCTGGCTCCGGGCTGGAGAGCGTGCTTGGAACCCAGCGCCGAGGTATGCAGACAGGAACA	-31.1	Novel-mir-314-5p	-	TGCGTATAGGGGACTGCTGGCTCC	-
Novel-mir-315	TTGTTACCATTGCTAACTCTGAGAGACGAGACGTGTGTGTGTGTCTGTTGATTAAGTGGTGTGTAGCTA	-19.3	Novel-mir-315-5p	-	TGCTAACTCTGAGAGACGAGACG	-
Novel-mir-316	GTATTGCTGTTGCTAGAGGTTCAATGAAGATGGCGAATAGAGATGGTAAGAGTGGGCATCCTTGTCTGGTTCTCTGGTGAGGAGTGAAACT	-22.15	Novel-mir-316-5p	-	TGCTAGAGGTTCAATGAAGATGGC	-
Novel-mir-317	CCAATAGAAATGCTGTTTAATACGGGCTGTGGTTTTATGCTTTCCTAAGTGGCCAGCCCAAGGAATAGAATGGCCCAGTAAGTAAACACAACCTCCCATA	-22.5	Novel-mir-317-5p	-	TGCTGTTTAATACGGGCTGTGGTT	-
Novel-mir-318	ATGATGAAGCTGCTTGAGTCTGGCTGTGAAACAGGGCTTGTTGTTGTTGTATGTGCAGAAGGACTTGGTGGGCACATACAT	-22.1	Novel-mir-318-5p	Novel-mir-318-3p	TGCTTGAGTCTGGCTGTGAAACAGG	TGTATGTGCAGAAGGACTTGGTGG
Novel-mir-319	CCCTGGCTGGTGCTTTCAGATACTCGACTAGGGTGCTATGAAACAGTACAGTCAGTTTAGAAAGAGGAGGAGGGGA	-21.3	Novel-mir-319-5p	-	TGCTTTCAGATACTCGACTAGG	-
Novel-mir-320	CTGCGCAGAGTGCTTTTCCTGGGGTGTGGTCTTCATTTTTGAGTGCAGATGGGCACTGAGATGGATTCTGGATTGGCCTGAGACAGACTG	-24.7	Novel-mir-320-5p	Novel-mir-320-3p	TGCTTTTCCTGGGGTGTGGTCTTCA	TGAGATGGATTCTGGATTGGCCTG
Novel-mir-321	GCAGGCAGCTTGGAAAGATGAAAGACCCTGGACCAAGAAACCTAGATAATTTTAATCATTCTGGGCTAACCCCATACTACGCAGGCA	-18.91	Novel-mir-321-5p	-	TGGAAAGATGAAAGACCCTGGACC	-
Novel-mir-322	GAACCCATGATGGAAAGGTAGACAGCATAGTCATCATTACTGGCTGATGGAGTCTGGTTCCCTGTGGTAAAACCATCCA	-18.3	Novel-mir-322-5p	Novel-mir-322-3p	TGGAAAGGTAGACAGCATAGTCA	TGATGGAGTCTGGTTCCCTGTGGTA
Novel-mir-323	TTATATGAGCTGGAAATGGATTTGTGAACGGAATAAGTCGAGACTGATACCGGAGTGAGTTAAGTCGACTACTAAATTAAATTTTCTGTTGTTCGAGTTG	-18.5	Novel-mir-323-5p	-	TGGAAATGGATTTGTGAACGGAA	-
Novel-mir-324	GAGGGATGGATGGAACGAGAGATTGACAGGCAGATTGGTGCAGCTTCTGCAGTAATGCGGTTGATGTACTGGTCCGTCGTGGTGAA	-26.5	Novel-mir-324-5p	-	TGGAACGAGAGATTGACAGGCAG	-
Novel-mir-325	GAGGGAAGCATGGAACGGGAAATTGACAGGCGGATCAATGCAGCTTCTGCAGTAATGCGGTTGGTGTACCGGTCCGTTGTGGTGAAG	-25.7	Novel-mir-325-5p	-	TGGAACGGGAAATTGACAGGCGGA	-
Novel-mir-326	GAGGGAAGGATGGAACGGGAGACTGACAGGCGGATCGGCGCAGCTTGTGCAGTAATGCGGTTGATGTACCGGTCCATCGTGG	-26.1	Novel-mir-326-5p	Novel-mir-326-3p	TGGAACGGGAGACTGACAGGCGGA	TGCAGTAATGCGGTTGATGTACCGG
Novel-mir-327	GAGGGAAGGATGGAACGGGAGATTGACAGGCGTATCGGTGCAGCTTCTGCAGTAATGCGGTCGATGTACCGGTCCATCGTGGTGAA	-31.8	Novel-mir-327-5p	-	TGGAACGGGAGATTGACAGGCGT	-
Novel-mir-328-1	ACAGAACAAGTGGAACTGAGTCAGGTTTGTAGGCCTCCTTTCTCGCACACACTTTTTCAGTTCTGCCCACAAATTTTCTATTGGACTGA	-21.63	Novel-mir-328-5p	-	TGGAACTGAGTCAGGTTTGTAGGC	-
Novel-mir-328-2	ACAGATCTGGTGGAACTGAGTCAGGTTTGTAGGCCTCCTTGCTCGCACACACTTTTTCAGTTCAGCCCACAAATGTTTTATTGGATTGAGGTCAGG	-27.2	Novel-mir-328-5p	-	TGGAACTGAGTCAGGTTTGTAGGC	-
Novel-mir-329	TTATGTGTGTTGGAAGTTAGGGACAGAGGAGGGAGAGTGTATCTTCTTGTTGTTTTTGTGTCAATTTCATGTGT	-18.5	Novel-mir-329-5p	-	TGGAAGTTAGGGACAGAGGAGGGA	-
Novel-mir-330	GCCAACAAGCTGGAATAGGTTCGGATACTGGAGCAATGTGTGGTGTTCAAATGAATCTCACAACCCATATGTTTGAACACTTTCTTACTGTATTTGAA	-22.3	Novel-mir-330-5p	-	TGGAATAGGTTCGGATACTGGAGC	-
Novel-mir-331	TCTTGGGGACTGGAATGATGGTGGAGTGTTTGAAGTAGGAGGGGACTTCACACAGCTCCAGTGACCTGTT	-22.7	Novel-mir-331-5p	-	TGGAATGATGGTGGAGTGTTTGA	-
Novel-mir-332	GAGGGAAGGATGGAATGGGAGATTGACAGGCAGATCGGTGCAGCTTCTGCAGTAATGCGGTCAATGTACCAGTCCGTTGTGGTGAA	-28.2	Novel-mir-332-5p	-	TGGAATGGGAGATTGACAGGCAGA	-
Novel-mir-333	GGCTGCTTGCTGGAATGTGATAGAAACCAGGTGTGCGTGTGTGTGTGTGTGTGTGTGTGTGAGATTTTCAGGCGGCAGCTT	-25	Novel-mir-333-5p	-	TGGAATGTGATAGAAACCAGGTGT	-

Novel-mir-334-1	CCCAGTCTGGTGGAGGTGGAAGGACGGCCCGTCAGTGTCCCCACTTTCACTGGCGGGCCTGCTCTTTTGGCCACACCGGGCAAGC	-49.9	Novel-mir-334-5p	-	TGGAGGTGGAAGGACGGCCCGTCA	-
Novel-mir-334-2	CCCGATCTGGTGGAGGTGGAAGGACGGCCCGTCAGTGTCCCCACTTTCACTGGCGGGCCCACTCTTTTGGCCGCACCGGGCAAG	-45.8	Novel-mir-334-5p	-	TGGAGGTGGAAGGACGGCCCGTCA	-
Novel-mir-335-1	CGACACACAGTGGATAACATGAATTTACCAGGGTAGTTTGATACCAGTGGACATCAGGATCCAGGACGTGTAATATGCGAAGTCGC	-20.1	Novel-mir-335-5p	-	TGGATAACATGAATTTACCAGGGT	-
Novel-mir-335-2	GTCGACACAGTGGATAACATGAATTTACCAGGGTAGTTCAATACCAGTGGACATCAGGATCCAGGATGTGTAATA	-19.7	Novel-mir-335-5p	Novel-mir-335-3p	TGGATAACATGAATTTACCAGGGT	CAGTGGACATCAGGATCCAGG
Novel-mir-336-1	AGCCAACCTCTGGATATGACGCTGAGCACGGGCACACAACTTCCTTGATCGACTACGGCGAGGCCTGTTCTGAGTGGAACCTGTCCTGTTAAACCGCTG	-26.03	Novel-mir-336-5p	Novel-mir-336-3p	TGGATATGACGCTGAGCACGGGC	CTGTTCTGAGTGGAACCTGTCCTGT
Novel-mir-336-2	AGCCATCTTCTGGATATGACGCTGAGCACGGGCACACAACTTTCTTGATCGACCACAGCGAGGCCTGTTCTGAGTGGAACCTGTCCTGTTAAACCGCTG	-24.83	Novel-mir-336-5p	Novel-mir-336-3p	TGGATATGACGCTGAGCACGGGC	CTGTTCTGAGTGGAACCTGTCCTGT
Novel-mir-337	GTGGTCGGGCTGGATCTTTTTGTCGGTAAGCTTGTTACTGCAGTAGGTGCGGAAGATGGCCAGCTTTTTTTTTG	-21.4	Novel-mir-337-5p	Novel-mir-337-3p	TGGATCTTTTTGTCGGTAAGCT	TGCAGTAGGTGCGGAAGATGGCCAGC
Novel-mir-338	CTTAAGTCTCTGGATGTTGTGGGGCTGGCTTGGCTGACACGCCTCTGCAATGTCACATGGCAGTTGGGGACAGTGA	-29.4	Novel-mir-338-5p	-	TGGATGTTGTGGGGCTGGCTTGGC	-
Novel-mir-339	CTCAAGTCTCTGGATGTTGTGGGGCTGTTTTGGCTGACACGCCTCTGCAACGTCGCATGGCGGTTGGGGACAGTGC	-28.8	Novel-mir-339-5p	-	TGGATGTTGTGGGGCTGTTTTGGC	-
Novel-mir-340	CTTACAGCGCTGGATTCAGAGAGACAGAGCTCACGCAGGAGAACCGTCTTCTGAATCCTAAATTAAGTTTTTACAACACATTTATCTTCTTGGGCTGCTC	-28	Novel-mir-340-5p	-	TGGATTCAGAGAGACAGAGCTCA	-
Novel-mir-341	TTCTTCACGATGGATTTAACTGAACTCTGGGGGATGCTCAGTGCCTTGGAAATTTCTTTGTATCCATCCCGACTTA	-19.7	Novel-mir-341-5p	-	TGGATTTAACTGAACTCTGGGGGA	-
Novel-mir-342	GCATTTTCTATGGATTTGGCATAGGCTGGCATGCATTTCAGCATGGGGGTGCCAGGTCAACTCTCTGTGTTTCATGAGACAGTATGGGGATGCG	-33.9	Novel-mir-342-5p	-	TGGATTTGGCATAGGCTGGCATGC	-
Novel-mir-343	CTTCAGACGTTGGATTTTATGATGGGGACGCAGGTAAGGTTTTCTTCTGCTGACTCTTCCATGAAGGTCGTGTCTGTGC	-20.8	Novel-mir-343-5p	-	TGGATTTTATGATGGGGACGCAGG	-
Novel-mir-344	CACCCTGTGGTGGCAAATGCTCTGCAGAGAGGGCAGTGGAGTCCTGGTGATCTTCTCCACGGTCCACCACTCTCTGCAGGTGCTTGCGGTCCATAGCAGT	-47	Novel-mir-344-5p	-	TGGCAAATGCTCTGCAGAGAGGGC	-
Novel-mir-345	CAGATACACATGGCTAGGAAAGTTCACCAGAGGTAATCATGGCATCGCCGTCAGATACACATGGCTAGGAAAGTTCCTCTAGAGGTAATCAC	-22.5	Novel-mir-345-5p	-	TGGCTAGGAAAGTTCACCAGAGG	-
Novel-mir-346	TATGGAAGATTGGCTGGTTCGTTGGATGGGATTGAGTATAGTTAACGTTTCTACACTGACCACCGTGGGACAGGATTGTGACCTCGTCGGACCAAG	-19.9	Novel-mir-346-5p	-	TGGCTGGTTCGTTGGATGGG	-
Novel-mir-347	GCCCCTTTGATGGGAATGTGGGTGGCCGAGAGCCAAACCTGTTAGGTAGGAACGGCCCTACGGCGGCGATCGGCCAGGCGTTTGTTTTGGTCGGCT	-36.3	Novel-mir-347-5p	-	TGGGAATGTGGGTGGCCGAGAGC	-
Novel-mir-348-1	TCACACAAGCTGGGACAGGAGCTCCTGAAGAGGAAGCTGACCATGGTGGGAACTATTCACAAAAACAATTCTGAGCTCCCACCTCAACTATTCATT	-22.3	Novel-mir-348-5p	-	TGGGACAGGAGCTCCTGAAGAGG	-
Novel-mir-348-2	TCGGAAGTAAGGGATGCCGTCAAGCTGAAGAAGGAGTCCTTTCGAGCCTGGTTGGCTCATGGGACTCCTGAGACAGCTGACGGGTACTAGCAGGCCAA	-46.5	Novel-mir-348-5p	Novel-mir-348-3p	TGGGACAGGAGCTCCTGAAGAGG	TCCTGAGACAGCTGACGGGTACT
Novel-mir-349	CTGAGGACACTGGGACTCACTCCTGGACTGGTTGGGTTTGATGCTACTGAAGACAAACAGAAACAGTACATACGGTGAGTTGTAGACTGTGCATCGA	-27.3	Novel-mir-349-5p	-	TGGGACTCACTCCTGGACTGGTTG	-
Novel-mir-350	CACTATGAGCTGGGACTGAAGACGTGCTCCAGAGTTTCATTTTGTGGTAAGTTTTATATCTTTGCTCCACTTT	-19.5	Novel-mir-350-5p	-	TGGGACTGAAGACGTGCTCCAGAG	-
Novel-mir-351	CTGCGGACACTGGGATTCACTCCTGGACTGGTTGTGGTGATGGTTGCTGAGGAGAGAGTCACAGAAACAGTTAATGGGAGACTGTGCATCGACCTGCGT	-27.7	Novel-mir-351-5p	-	TGGGATTCACTCCTGGACTGGTTG	-
Novel-mir-352	GACTCATCCATGGGATTGCCAGACAGAGAAGCGCGATTCGTTACCCAGAGAACACGTCTCCACTGCTGTCGAGTCCAGTGGCAGCGTG	-22	Novel-mir-352-5p	-	TGGGATTGCCAGACAGAGAAGCG	-
Novel-mir-353-1	AGTACACAGCTGGGATTTACTGTGTAGGGGACTTATTCCTACTCCTCTACACCAAGCCAGTTGCTCAGACA	-22	Novel-mir-353-5p	-	TGGGATTTACTGTGTAGGGGAC	-
Novel-mir-353-2	AGTACACAGCTGGGATTTACTGTGTAGGGGACTTATTCCTACTCCTCTACACCAAGCCAGTTGCTCAGGGTTGCTCTGCAACAACCCTGTGTTCC	-33.9	Novel-mir-353-5p	-	TGGGATTTACTGTGTAGGGGAC	-
Novel-mir-353-3	CGTACACAGCTGGGATTTACTGTGTAGGGGACTTATTCCTACTCCTCTACACCAAGCCAGTTGTTCAGACA	-23.9	Novel-mir-353-5p	-	TGGGATTTACTGTGTAGGGGAC	-
Novel-mir-353-4	GGTACACAGCTGGGATTTACTGTGTAGGGGACTTATTCCTACTCCTCTACACCAAGCCAGTTGCTCAGACA	-21.8	Novel-mir-353-5p	-	TGGGATTTACTGTGTAGGGGAC	-
Novel-mir-354	GTTACGTCTTTGGGGACAACTGTGTTGAAGGCGGAGCTTTAGTCAATGAAGAGTATCCTGATGTAGGAGTCTCCATTTTCCAGGTGAG	-22.8	Novel-mir-354-5p	-	TGGGGACAACTGTGTTGAAGGCGG	-

Novel-mir-355	AACAGTGTAGTGGGGTTGACTGTCAGTGACTCACTCGTGTGCCATCATATAAATCACATGACTGGGTAGCTGAATTGCTCAAGGCCACTCATTCCT	-32.72	Novel-mir-355-5p	-	TGGGGTTGACTGTCAGTGACT	-
Novel-mir-356-1	CAGGTAGAACTGGGTATAGAGATGTTGATGACACTGGGGGCGCTGTAGGGATGGCCATGTCTTTAATCTTCTCTTGTCCAGTCTGTTCCTT	-28.5	Novel-mir-356-5p	Novel-mir-356-3p	TGGGTATAGAGATGTTGATG	TTAATCTTCTCTTGTCCAGT
Novel-mir-356-2	TAGGTAAAACTGGGTATAGAGATGTTGATGCCACTGGGGGCACTGTAGGGGTGACCATGTCTTTAATCTTCTCTTGTCCAGCCTGTTCCTT	-24.3	Novel-mir-356-5p	-	TGGGTATAGAGATGTTGATG	-
Novel-mir-357	AAGTCTGACATGGGTTAGAACAATCTGGCAGCTTCCCACAGGGTGAGCAGCTGCTATATACTGTTGTTAGCAGGTGTGGGTAAT	-25.2	Novel-mir-357-5p	-	TGGGTTAGAACAATCTGGCAG	-
Novel-mir-358	CACCCTGCGGTGGTAAATACTCTGCTGAGAGGGCAGCGGAGTCCTGGTGATCTTCTCCGCAGTCTTTACCACTCTCTGCAGGCGC	-39	Novel-mir-358-5p	-	TGGTAAATACTCTGCTGAGAGGGC	-
Novel-mir-359	CACCCTGTGGTGGTAAATGCACTGCAGAGAGGGCAGTGGAGTCCTGGTGATCTTCTCCGCAGTCTTTACCAATCTCTGCAGGTGC	-35.7	Novel-mir-359-5p	-	TGGTAAATGCACTGCAGAGAGGGC	-
Novel-mir-360	CACCCTGTGGTGGTAAATGCTCTACAGAGAGGGCAGGGGCGTCCTAGTGATCTTCTCTGCAGTCTTGACCACTCTCTGCAGGCGCTTGCGGTCCATAACAG	-35	Novel-mir-360-5p	-	TGGTAAATGCTCTACAGAGAGGGC	-
Novel-mir-361-1	CACCCTGCGGTGGTAAATGCTCTGCAAAGAGGGCAACGGAGTCCTGGTGATCTTCTCAGCAATCTTTACCACTCTCTGCAGGCGCTTGCGGTCCATAGCCG	-32.5	Novel-mir-361-5p	-	TGGTAAATGCTCTGCAAAGAGGGC	-
Novel-mir-361-2	CACCCTGCGGTGGTAAATGCTCTGCAAAGAGGGCAGTGGAGTCCTAGTGATCTTCTTCGCAGTCTTTACCACCAGACAGTGATGCAGCTGGTC	-35.4	Novel-mir-361-5p	-	TGGTAAATGCTCTGCAAAGAGGGC	-
Novel-mir-361-3	CACCCTGCGGTGGTAAATGCTCTGCAAAGAGGGCAGTGGAGTCCTGGTGATCTTCTTCGCAGTCTTTACCACCAGACAGTGATGCAGCTGGTC	-34.1	Novel-mir-361-5p	-	TGGTAAATGCTCTGCAAAGAGGGC	-
Novel-mir-361-4	CACCCTGTGGTGGTAAATGCTCTGCAAAGAGGGCAGTGGAGTCCTAGTGATCTTTGCCGCAGTCTTTACCACTCTCTGCAGGCGC	-34.3	Novel-mir-361-5p	-	TGGTAAATGCTCTGCAAAGAGGGC	-
Novel-mir-362-1	CACCCTTCGGTGGTAAATGCTCTGCAGAAAGGGCATTGGAGTCCTGGTGATCTTCTCTGCAGTCTTTACCACTCTCTGCAGGCGC	-31.2	Novel-mir-362-5p	-	TGGTAAATGCTCTGCAGAAAGGGC	-
Novel-mir-362-2	CACCATGCAGTGGTAAATGCTCTGCAGAGAAGGCAGTGGAGCCCTAGTGATCTTCTCCGCAGTCTTTACCACTCTCTACAGGCACTTGCGGTC	-32.8	Novel-mir-362-5p	-	TGGTAAATGCTCTGCAGAGAAGGC	-
Novel-mir-362-3	CACCCTGCAGTGGTAAATGCTCTGCAGAGAAGGCAGTGGAGTCCTGGTGATTTTCTCCGCAGTCTTTACCACCCCCTGCAGGTGC	-34.7	Novel-mir-362-5p	-	TGGTAAATGCTCTGCAGAGAAGGC	-
Novel-mir-362-4	CACCCTGCGGTGGTAAATGCTCTGCAGAGAAGGCAGTGGAGTCCTGGTGATCTTCTCCGCAGTCTTTACCACTCTCTGCAGGCGC	-37	Novel-mir-362-5p	-	TGGTAAATGCTCTGCAGAGAAGGC	-
Novel-mir-363-1	CACCCTGCGGTGGTAAATGCTCTGCAGAGAGAGCAGTGGAGTCCTGGTGATCTCCACAGTCTTTACCACTCTCTGCAGGCGCTTGCAGTCCATAGCAGTAG	-40.5	Novel-mir-363-5p	-	TGGTAAATGCTCTGCAGAGAGAGC	-
Novel-mir-363-2	CACCCTGCTGTGGTAAATGCTCTGCAGAGAGAGCAGTGGAGTCCTGGTGATCTTATTGTGCTTATTTATCTTATTTCAGTGTGA	-18.8	Novel-mir-363-5p	-	TGGTAAATGCTCTGCAGAGAGAGC	-
Novel-mir-363-3	CACTCTGCGGTGGTAAATGCTCTGCAGAGAGAGCAGTAGAGTCCTGGTGATCTCCACAGTCTTTACCACTGTCTGCAGGTGC	-30.4	Novel-mir-363-5p	-	TGGTAAATGCTCTGCAGAGAGAGC	-
Novel-mir-364-1	ACCCTGCGGGTGGTAAATGCTCTGCAGAGAGGCCAGTGGAGTCCTGGTGATCTTTTACACAGTCTTTACCACTTTCAGCAGGCA	-32.9	Novel-mir-364-5p	-	TGGTAAATGCTCTGCAGAGAGGC	-
Novel-mir-364-2	CACCCTGCAGTGGTAAATGCTCTGCAGAGAGGCCCAGTGGAGTCCTGGTGATCTTTTCCGCAGTCTTTACCACTCTCTGCAGGTGC	-33.2	Novel-mir-364-5p	-	TGGTAAATGCTCTGCAGAGAGGC	-
Novel-mir-365-1	CACCCTGCGGTGGTAAATGCTCTGCAGAGAGGGTAGTGGAGTCCTGGTGATCTTCTCAGCAGCAGCACTTACCACTCTCTGCAGGTGC	-40.9	Novel-mir-365-5p	-	TGGTAAATGCTCTGCAGAGAGGGT	-
Novel-mir-365-2	CACCCTGCGGTGGTAAATGCTCTGCAGAGAGGGTAGTGGAGTCCTGGTGATCTTCTCCACAGTCTTTACCAGTCTCTGCAGGTACTTGCCATCCATAGCAG	-44.8	Novel-mir-365-5p	-	TGGTAAATGCTCTGCAGAGAGGGT	-
Novel-mir-365-3	CACCCTGTGGTGGTAAATGCTCTGCAGAGAGGGTAGTGGAGTCCTGGTGATCTTCTCCACAGTCTTTACCACTCTCTGCAGGCGCTTGCGGTCCATAGCAG	-47	Novel-mir-365-5p	-	TGGTAAATGCTCTGCAGAGAGGGT	-
Novel-mir-365-4	CAGCTTGTGGTGGTAAATGCTCTGCAGAGAGGGTAGTGGAGTCCTAGTGATCTTCTCTGCAGTCTTTACCACTCTCTGCAGGGGCTTTGCGTGCTGC	-39.9	Novel-mir-365-5p	-	TGGTAAATGCTCTGCAGAGAGGGT	-
Novel-mir-366	CACCCTGCGGTGGTAAATGCTCTGCAGAGATGGCAGTGGAGTCCTGGTGATCTTCTCCACAGTCTTTACCACTCTCTCTCTTTAAGGTCT	-23.72	Novel-mir-366-5p	-	TGGTAAATGCTCTGCAGAGATGGC	-
Novel-mir-367	CACCCTGCGGTGGTAAATGCTCTGCAGAGGGGGCAGTGGAATCCTGGTGATCTTCTCTGCAGTCTTTACCACTCTCTGCAGGCGC	-40.3	Novel-mir-367-5p	-	TGGTAAATGCTCTGCAGAGGGGGC	-
Novel-mir-368-1	CACCCTGCAGTGGTAAATGCTCTGCAGAGTGGGCAGTGGAGTCCTGGTGATCTTCTCCGCAGTCTTTACCACTCTCTGCAGGTGCTTGTGG	-33.5	Novel-mir-368-5p	-	TGGTAAATGCTCTGCAGAGTGGGC	-
Novel-mir-368-2	CAGCCTGCGGTGGTAAATGCTCTGCAGAGTGGGCAGTGGAGTCCTAGTGATCTTCTCTGCAGTCTTTACCACTCTCTGCAGGCGC	-43.1	Novel-mir-368-5p	-	TGGTAAATGCTCTGCAGAGTGGGC	-

Novel-mir-369	CACCATGCGGTGGTAAATGCTCTGCAGGGAGGGCAGTGGACTCCTGGTGATCTTCTCGACAGTCTTTACCACTCTCTGCAGGCGCTTGCTGCCATACCAGA	-40.6	Novel-mir-369-5p	-	TGGTAAATGCTCTGCAGGGAGGGC	-
Novel-mir-370-1	CACCCTGCAGTGGTAAATGCTCTGCGGAGAGGGCAGTGGTGTCCTGGTGATCTTGTCTGCAGTCTTTACCACTCTCTGCAGGCACTTGCAGTCCATAGCAG	-39.3	Novel-mir-370-5p	-	TGGTAAATGCTCTGCGGAGAGGGC	-
Novel-mir-370-2	CACCCTGCGGTGGTAAATGCTCTGCGGAGAGGGCAGTGGAGTCCTGTTTACTGTCTGTTTATACTGCTTTTTCTGCATTGTTTGCACTGTTTGTTGA	-30.72	Novel-mir-370-5p	-	TGGTAAATGCTCTGCGGAGAGGGC	-
Novel-mir-370-3	CACCCTGTGGTGGTAAATGCTCTGCGGAGAGGGCAGTGGAGTCCTGGTGATCTTCTCTGCAGTCTTTATCACTCTCTGCAGGTGCTTGTGG	-37.5	Novel-mir-370-5p	-	TGGTAAATGCTCTGCGGAGAGGGC	-
Novel-mir-370-4	AACCCTACGGTGGTAAATGCTCTGTAGAGAGGGCAGCGGAGCCCTGGTGATCTTCTCTGCAGCCTTTACCATTCTCTGCAGGTGC	-34.3	Novel-mir-370-5p	-	TGGTAAATGCTCTGTAGAGAGGGC	-
Novel-mir-370-5	CACCCTGCGGTGGTAAATGCTCTGTAGAGAGGGCACTGGAGTCCTGGTGATCTTCTCTACAGTCTTTACCACTCTCTGCAGGTGC	-43.7	Novel-mir-370-5p	-	TGGTAAATGCTCTGTAGAGAGGGC	-
Novel-mir-370-6	CACCCTGCGGTGGTAAATGCTCTGTAGAGAGGGCAGTGGAGTCCTGGTGATCTTCTCTGCAGTCTTTACCACTCTCTGCAGGCGC	-39.3	Novel-mir-370-5p	-	TGGTAAATGCTCTGTAGAGAGGGC	-
Novel-mir-370-7	CACCCTGCGGTGGTAAATGCTCTGTAGAGAGGGCAGTGGAGTCCTGGTGATCTTCTCTGCAGTCTTTACCACTCTCTGCAGGTGC	-39.5	Novel-mir-370-5p	-	TGGTAAATGCTCTGTAGAGAGGGC	-
Novel-mir-370-8	TTCCCTGTGGTGGTAAATGCTCTGTAGAGAGGGCAGTGGAGTCCTGGTGATTTTCTCGGTAGTCTTTACCACTCTTTGCAGGTGC	-30.9	Novel-mir-370-5p	-	TGGTAAATGCTCTGTAGAGAGGGC	-
Novel-mir-370-9	CACCCTGCAGTGGTAAATGTTCTGCAGAGAGGGCAGTGGAGTCTTGGTGATCTTCTCCGCAGTCTTTACCACTCTCTGCAGGCGC	-34.2	Novel-mir-370-5p	-	TGGTAAATGTTCTGCAGAGAGGGC	-
Novel-mir-370-10	CACCCTGCGGTGGTAAATGTTCTGCAGAGAGGGCAGTGGAGTCCTGGTGATCTTCTCTGCAGCCTTTACCACTCTCTGCAGGCGCTTGTGG	-41.5	Novel-mir-370-5p	-	TGGTAAATGTTCTGCAGAGAGGGC	-
Novel-mir-370-11	CACCCTGCGGTGGTAAATGTTCTGCAGAGAGGGCAGTGGAGTCCTGGTGATCTTCTCTGCAGTCTTTACCACTCTTTGTGGACGCTTGCAGTCCA	-39.8	Novel-mir-370-5p	-	TGGTAAATGTTCTGCAGAGAGGGC	-
Novel-mir-371	CGCTCTGCGGTGGTAAATTCTCTGCAGAGAGGGCAGTGGAGTCCTGGTGATCTTCTCCACAGTCTTGACCACTCTCTGCAGGCGCTTGCGGTCCATAGCA	-38.7	Novel-mir-371-5p	-	TGGTAAATTCTCTGCAGAGAGGGC	-
Novel-mir-372	CACCCTGCGGTGGTAAGTGCTCTGCAGAGAGGGCAGTGGAGTCCTGGTGATCTTCTCTGAAGTCTTTGCCACTCTCTGCAGGCGCTTGCGGTCCGTAGCAG	-53.1	Novel-mir-372-5p	-	TGGTAAGTGCTCTGCAGAGAGGGC	-
Novel-mir-373	GTGTCGGATGTGGTAGTCTGAAGCACAGGACGGTGCTTTCCCCCTTTCTGTTCACCCTCTACACGTCGGACTT	-22.8	Novel-mir-373-5p	-	TGGTAGTCTGAAGCACAGGACGGT	-
Novel-mir-374	GTGGGGTTTATGGTATTATCTTGGGTGCTGCACGCCTTCGCCAGTGTGTTAATTATTTCTTTGTTATTTAGGATGGCAGCCACTAAATTT	-18.74	Novel-mir-374-5p	Novel-mir-374-3p	TGGTATTATCTTGGGTGCTGCACG	TTGTTATTTAGGATGGCAGC
Novel-mir-375	CGACGGGTGCTGGTATTGGAGACCCCCAGCCGAGGAAAAGCCCGGGCCTCGACTGGTGGTTCTGCTGTGGCACGGGGCCGG	-31.8	Novel-mir-375-5p	Novel-mir-375-3p	TGGTATTGGAGACCCCCAGCCGAGG	TCGACTGGTGGTTCTGCTGTGGC
Novel-mir-376	TACCTTGCGGTGGTGAATGCTCTGCAGAGAGGGCAGTGGAGTCCTGGTGATTTTCCCTGCACTCTTTACCACTCTCTGCAGGCGCTTGTGGTCCATAGCAGTTGT	-40.6	Novel-mir-376-5p	Novel-mir-376-3p	TGGTGAATGCTCTGCAGAGAGGGC	CACTCTCTGCAGGCGCTTGTGGTCCA
Novel-mir-377	GATATGAGTATGGTTAAATCTCAGCTGCCTGACATTGAGAAAGGAGGTGCACCGTAGATTAAGGCATTTTTAGGTGCGTGCGCAGGATTTGTGCCTGCACT	-24.7	Novel-mir-377-5p	-	TGGTTAAATCTCAGCTGCCTGACA	-
Novel-mir-378	CAGGTTGGTTTGGTTGTAGACTGTTTTTAGGGGATTACTGCATGATCTTCGGGAGTGATAATCGATGCAAGCGTAATCCAGCAGA	-20	Novel-mir-378-5p	-	TGGTTGTAGACTGTTTTTAGG	-
Novel-mir-379	ACTGAGTTCCTGGTTTGAAAGAACTGGACGTGGCTTTATAATAGAATAGAGCCTTTACTATTAACTGGCTATGTTCCACAGGCTTTTAAGGTTGCTGGAAT	-27.6	Novel-mir-379-5p	-	TGGTTTGAAAGAACTGGACGTGGC	-
Novel-mir-380-1	TCGGAGTTCCTGGTTTGAAAGAACTGGATGAGGCTTTATAATGGTTTACAGCCTTTATACAGTAAGTTTATAGTTTCAATTTCGTGGAACATTTA	-22.2	Novel-mir-380-5p	-	TGGTTTGAAAGAACTGGATGAGGC	-
Novel-mir-380-2	TCTGAGTTCCTGGTTTGAAAGAACTGGATGAGGCTTTATAATAGAATAGAGCCTTTACCGGTTTAGCATACTCACTAAGTCCTG	-25.3	Novel-mir-380-5p	-	TGGTTTGAAAGAACTGGATGAGGC	-
Novel-mir-380-3	GTTTAGCATTAGCTCGCAGCACCACACATGCCTCCCATTCAGCAATGTGGTTTTGTTTGAGGTGCTGGAATAAATTCGTCA	-21.6	Novel-mir-380-3p	-	TGGTTTTGTTTGAGGTGCTGGAAT	-
Novel-mir-380-4	GTTTAGCATTAGCTCGCAGCGCCACACACGCCTCCCATTCAGCAATGTGGTTTTGTTTGAGGTGCTGGAATAAATTCGTCG	-20.5	Novel-mir-380-3p	-	TGGTTTTGTTTGAGGTGCTGGAAT	-
Novel-mir-380-5	GTTTAGCGTTAGCTCGCAGCGCCACACACGCCTCCCATTCAGCAATGTGGTTTTGTTTGAGGTGCTGGAATAAATTTGTCG	-21.2	Novel-mir-380-3p	-	TGGTTTTGTTTGAGGTGCTGGAAT	-
Novel-mir-381	TTGGCACATGTGTAAATGTATGGTCTGGCAGGAGTACCACCTCTTTTGTAAAAGACTGAGCCGTAGAATGTGAAAC	-18.1	Novel-mir-381-5p	Novel-mir-381-3p	TGTAAATGTATGGTCTGGCAGGAG	TTTGTAAAAGACTGAGCCGTAGAAT
Novel-mir-382-1	CACCAGCCACTGTAACAGGAAGAGTGGCAGAGATTCTCAAAGCAACCCACATGTTGGGGTAAATTTCTGCCAGCTTCTTCTCATGCAGGAAGGTCAAAA	-32.2	Novel-mir-382-5p	-	TGTAACAGGAAGAGTGGCAGAG	-

Novel-mir-382-2	CAGCAGCCACTGTAACAGGAAGAGTGGCAGAGATTCTCAAAGCAACCCACATGTTGGGGTAAATTTCTGCCAGCTTCTTCTCATACAGGAAGGTCAAAA	-32.2	Novel-mir-382-5p	-	TGTAACAGGAAGAGTGGCAGAG	-
Novel-mir-382-3	CAGCAGCCACTGTAACAGGAAGAGTGGCAGAGATTCTCAAAGCAACCCACATGTTGGGGTAAATTTCTGCCAGCTTCTTCTCATGCAGGAAGGTCAAAA	-32.2	Novel-mir-382-5p	-	TGTAACAGGAAGAGTGGCAGAG	-
Novel-mir-382-4	CAGCAGCCACTGTAACAGGAAGAGTGGCAGAGATTCTCAATGCAACCCACATGTTGGGGTAAATTTCTGCCAGCTTCTTCTCATGCAGGAAGGTCAAAA	-33.1	Novel-mir-382-5p	-	TGTAACAGGAAGAGTGGCAGAG	-
Novel-mir-382-5	CAGCAGCCGCTGTAACAGGAAGAGTGGCAGAGATTCTCAAAGCAACCCACATGTTGGGGTAAATTTCTGCCAGCTTCTTCTCATGCAGGAAGGTCAAAA	-32.2	Novel-mir-382-5p	-	TGTAACAGGAAGAGTGGCAGAG	-
Novel-mir-382-6	CCCCAGCCACTGTAACAGGAAGAGTGGCAGAGATTCTCAAAACAACCCACATGTTGGGGTAAATTTCTGCCAGCTTCTTCTCATGCAGGAAGGTCAAAA	-33.2	Novel-mir-382-5p	-	TGTAACAGGAAGAGTGGCAGAG	-
Novel-mir-382-7	CCCCAGCCACTGTAACAGGAAGAGTGGCAGAGATTCTCAAAGCAACCCACATGTTGGGGTAAATTTCTGCCAGCTTCTTCTCATGCAGGAAGGTCAAAA	-32.2	Novel-mir-382-5p	-	TGTAACAGGAAGAGTGGCAGAG	-
Novel-mir-383	CAGCAGCCACTGTAACAGGAAGAGTGGCAGAGATTCTCAAAGCAACCCACATGTTGGGGTAAATTTCTGCCAGCTCCTTCTCATGCAGGAAGGTCAAAA	-32.5	Novel-mir-383-5p	-	TGTAACAGGAAGAGTGGCAGAGA	-
Novel-mir-384	TCTCCACAGATGTAATTCAAGGTGGACGACGGTGTTTGGAAGGAGCCGTTCCAGTGTTGTTTGCCTGGAATGACTACTCTCTGCCTGCA	-27.5	Novel-mir-384-5p	-	TGTAATTCAAGGTGGACGACGGTG	-
Novel-mir-385-1	TTCCTCAGTCTGTACTTTGCAGCTTTGTACTCTGTCTCATTGCCTGATTTGAATGCAAGACAGCAAGCATGCAGCATGTGGCGTACCTGCTGTGTGC	-25.6	Novel-mir-385-5p	-	TGTACTTTGCAGCTTTGTACTCT	-
Novel-mir-385-2	TTCCTCAGTCTGTACTTTGCAGCTTTGTACTCTGTCTCGTTGCCTGATCTGAATGCATGAGAGTGATAATGCAGCATGTGGCATACCTGACTGTTTTA	-22.3	Novel-mir-385-5p	-	TGTACTTTGCAGCTTTGTACTCT	-
Novel-mir-386	CAGTCATTGGTGTAGAGTGAAAACAGCAGAGGGTCCAATTGTCCGGATGCTGGATGTGGTTTTCCCCAGCTGCACCTGTTCACAA	-26.2	Novel-mir-386-5p	-	TGTAGAGTGAAAACAGCAGAGGGT	-
Novel-mir-387	CAGACAAGTCTGTAGGCTGTACAGAATGTCTGGGTTCCTTTTAGAGGTATTCTGTGGTTTTCTGAAGATGTACACTGG	-18.7	Novel-mir-387-5p	-	TGTAGGCTGTACAGAATGTCTGGG	-
Novel-mir-388-1	AGAGATCTGTTGTATTAGAAGATGTTGGTGTAGCCAGGATGTCTCCTCAGGCTGTTAAATGCAAGGTTAGTTAAAAGCAGAGAAAGGCGG	-19.2	Novel-mir-388-5p	Novel-mir-388-3p	TGTATTAGAAGATGTTGGTGTAGC	TAAATGCAAGGTTAGTTAAAAGCAG
Novel-mir-388-2	AGAGGTCTGTTGTATTAGAAGATGTTGGTGTAGCCAGGATGTCTCCTCAGGCTGTTAAATGCAAGGTTAGTTAAAGCAGAGAAAGGCTGCA	-21.9	Novel-mir-388-5p	-	TGTATTAGAAGATGTTGGTGTAGC	-
Novel-mir-389-1	ACCTTATAAATGTATTAGGTTCTGTGTGGCGGGGGGAACTCCACTTTCTAGCGGCCTGTGTGTGTGTCTGGA	-20.6	Novel-mir-389-5p	-	TGTATTAGGTTCTGTGTGGCGG	-
Novel-mir-389-2	ACCTTATAAATGTATTAGGTTCTGTGTGGCGGGGGGGACTCCACTTTCCAGCGGCCTGTGTGTGTGTGTGTCTGGA	-24.3	Novel-mir-389-5p	-	TGTATTAGGTTCTGTGTGGCGG	-
Novel-mir-389-3	ACCTTATAGATGTATTAGGTTCTGTGTGGCGGGGGGAAAAAAATCCACTTTCCAGCGGCATGTGTGTGTATCTGGA	-18.1	Novel-mir-389-5p	-	TGTATTAGGTTCTGTGTGGCGG	-
Novel-mir-390	GTTAGTATCTTGTATTCCTTTGATGCTATTATTCTGTCATATTGCTGCTGCTAGAGGCTCAATGAAAATGGCAAATAGAGATGGTGAGAGTGGGCA	-18.6	Novel-mir-390-5p	-	TGTATTCCTTTGATGCTATTATT	-
Novel-mir-391-1	ACATGCTGTCTGTATTTGGGCAGTTCGTGGGAGAGATTTGCTCTGTTAAAGTCCCCTAGAACAATGAGTAGAGAGTCAGGATGTT	-23	Novel-mir-391-5p	-	TGTATTTGGGCAGTTCGTGGGAGA	-
Novel-mir-391-2	ACATGCTGTCTGTATTTGGGCAGTTCGTGGGAGAGATTTGCTCTGTTAGAGTCTCCTAGAACAATGAGTGGAGAGTCGGGATGTT	-25.4	Novel-mir-391-5p	Novel-mir-391-1-3p	TGTATTTGGGCAGTTCGTGGGAGA	TCCTAGAACAATGAGTGGAGA
Novel-mir-391-3	ATGTGCTGTCTGTATTTGGGCAGTTCGTGGGAGAGATTTGCCCTGTTAATGTCCCCTAGAACAATGAGTGGAGAGTCAGGATGTT	-20.5	Novel-mir-391-5p	Novel-mir-391-2-3p	TGTATTTGGGCAGTTCGTGGGAGA	CCCTAGAACAATGAGTGGAGA
Novel-mir-391-4	ATGTGCTGTCTGTATTTGGGCAGTTCGTGGGAGAGATTTGCTTTGTTAAAGTCTCCTAGAACAATGAGTGGAGAGTCGGGATGTTCC	-22	Novel-mir-391-5p	Novel-mir-391-3-3p	TGTATTTGGGCAGTTCGTGGGAGA	TCCTAGAACAATGAGTGGAGAGTC
Novel-mir-392	AGAGCAGTGTCCTGTTCCTTGGTGTCACCTGTTGAGAGATGCTCATCACAGTGTTATTCAGTGTTCGTGCATATTCACACAAGGCCTCATGTTGTAACTAA	-20.7	Novel-mir-392-5p	Novel-mir-392-3p	TGTCACCTGTTGAGAGATGCTCA	TGCATATTCACACAAGGCCTCATG
Novel-mir-393	GTTTGTCGGATGTCATGTGAATCACTTTGGATCGAGTGTGTTCAGAGACAGGATATCCCTATTTAGTGTCGTTCATTTGAATCTGTCTTTGCA	-22	Novel-mir-393-5p	-	TGTCATGTGAATCACTTTGGATCG	-
Novel-mir-394-1	CTAGTCTCCGTGTCCAGAGATCGGGCCGTCGGGGCCCCTGCCTTCGACTGCTGCCCAATCCACTATGCACCGGCCAGTCCAGA	-25.6	Novel-mir-394-5p	-	TGTCCAGAGATCGGGCCGTCGG	-
Novel-mir-394-2	CTAGTCTCCGTGTCCAGAGATCGGGCCGTCGGGGCCCCTGCCTTCGACTGCTGCCCAATCCACTATGCACCGGCCCCTTATGGATCCT	-26.4	Novel-mir-394-5p	-	TGTCCAGAGATCGGGCCGTCGG	-
Novel-mir-394-3	CTAGTCTCCGTGTCCAGAGATCGGGCCGTCGGGGCCCCTGCCTTCGACTGCTGCCCAATCCACTGTGCACCGGCCCCTTGCGGATCCT	-29.8	Novel-mir-394-5p	Novel-mir-394-3p	TGTCCAGAGATCGGGCCGTCGG	GACTGCTGCCCAATCCACTGTGCACC
Novel-mir-395	GCTTGGCCGGTGTCTATGTGATGCTGGACCAGGCTCGTCCGGGTGCAGTCCTCATCACAGGCAGCAAAAATGTCCAAGA	-28	Novel-mir-395-5p	-	TGTCTATGTGATGCTGGACCAGGC	-

Novel-mir-396-1	CTGGAGGAAGTGTCTGGAGAGAGGAAAGTCTGGGCATCTTTGCTTAGACTGCTGCCCCCACGACCCGATCCCGGATAAGCGGAGGA	-27.4	Novel-mir-396-5p	-	TGTCTGGAGAGAGGAAAGTCTGGG	-
Novel-mir-396-2	CTGGAGGAAGTGTCTGGAGAGAGGAAAGTCTGGGCATCTTTGCTTAGACTGCTGCCCCCGCGACCCGGTCCCGGA	-27.9	Novel-mir-396-5p	-	TGTCTGGAGAGAGGAAAGTCTGGG	-
Novel-mir-396-3	CTGGAGGAAGTGTCTGGAGAGAGGAAAGTCTGGGCATCTTTGCTTAGACTGCTGCCCCCGCGACCCGGTCCTGGAT	-26	Novel-mir-396-5p	-	TGTCTGGAGAGAGGAAAGTCTGGG	-
Novel-mir-396-4	CTGGAGGAAGTGTCTGGAGAGAGGAAAGTCTGGGCATCTTTGCTTAGACTGCTGCCCCCGCGACCCGGTTCTGGA	-25.2	Novel-mir-396-5p	-	TGTCTGGAGAGAGGAAAGTCTGGG	-
Novel-mir-396-5	CTGGAGGAAGTGTCTGGAGAGAGGAAAGTCTGGGCATCTTTGCTTAGACTGCTGCCCTCGCGACCCGGTCCCGGA	-30.5	Novel-mir-396-5p	Novel-mir-396-3p	TGTCTGGAGAGAGGAAAGTCTGGG	TTGCTTAGACTGCTGCCCTCGCGACC
Novel-mir-396-6	CTGGGGGAAGTGTCTGGAGAGAGGAAAGTCTGGGCATCTTTGCTTAGACTGCTGCCCCCGCGACCTGGTCCCGGA	-32.6	Novel-mir-396-5p	-	TGTCTGGAGAGAGGAAAGTCTGGG	-
Novel-mir-397-1	CTGGAGGAAGTGTCTGGAGAGTGGGAAGTCTGGGCATCTCTGCTTAGACTGCTGCCTCCGCGACCCGGTTTCGGA	-32.6	Novel-mir-397-5p	-	TGTCTGGAGAGTGGGAAGTCTGGG	-
Novel-mir-397-2	CTGGAGGAAGTGTCTGGAGAGTGGGAAGTCTGGGCATCTTTGCTTAGAATGCTGCCCTCATGACCCGATCTCAGATAAGTGGAAAAA	-25.4	Novel-mir-397-5p	-	TGTCTGGAGAGTGGGAAGTCTGGG	-
Novel-mir-397-3	CTGGAGTAAGTGTCTGGAGAGTGGGAAGTCTGGGAATCCTTGCTTAGACTGCTGCCCTCGTGACCCGGTTCCGGA	-27	Novel-mir-397-5p	-	TGTCTGGAGAGTGGGAAGTCTGGG	-
Novel-mir-398	TTCGGCAGGCTGTCTGTGGAGCTGATGATGGTGGTCCTGTAGTCTGTGATGTGCTGCAGGCCTTGCCACATCCGTCCAGAGTCAGCAGTAGAAT	-41.2	Novel-mir-398-5p	Novel-mir-398-3p	TGTCTGTGGAGCTGATGATGGT	CACATCCGTCCAGAGTCAGC
Novel-mir-399	TCCAGGAGGCTGTCTGTGGAGCTGATGATGGTGTGGTCCTGTAGTCTGTGATGTGCTGCGGGCCTTGCCACATCTGCCCGGAGTCAGCAGTAGAAT	-42.3	Novel-mir-399-5p	-	TGTCTGTGGAGCTGATGATGGTG	-
Novel-mir-400	TCCGGTAGGCTGTCTGTGGGGCTGATGATGCTGGTGGTGGTCCTGTAGTTCGTGATGTGCTGCAGGCCTTGCCACA	-28.8	Novel-mir-400-5p	-	TGTCTGTGGGGCTGATGATGCTGG	-
Novel-mir-401-1	AGTGAGTCAATGTCTTGTTCACTCCTGGCATACAGCTTAATGTCATCCATGCAGAGGAGGTGGCTGATGGTTGCTCC	-19.9	Novel-mir-401-5p	Novel-mir-401-3p	TGTCTTGTTCACTCCTGGCATACA	TCCATGCAGAGGAGGTGGCTGA
Novel-mir-401-2	AGTGAGTTGATGTCTTGTTCACTCCTGGCATACAGCTTGATGTCATCCATGTAGAGGAGGTGGCTGATGGTTGCTCCAT	-19.6	Novel-mir-401-5p	Novel-mir-401-3p	TGTCTTGTTCACTCCTGGCATACA	TCCATGTAGAGGAGGTGGCTGATG
Novel-mir-402	AGGCGGACTCTGTGACATCAGTCAGGACCTCAGTGCTGTGGAAGTCCAGAGGAAGCCGACACTCGTCCAGACCGTCAA	-28.1	Novel-mir-402-5p	Novel-mir-402-3p	TGTGACATCAGTCAGGACCTCAGTGC	TCCAGAGGAAGCCGACACTCGTCC
Novel-mir-403	TCTAGGGCGATGTGAGACCAGGGCCGACTAGGTGTTGTCAGCAGTCGAAGAAGACCCGCTGGGGACTGATGGAGTGGCTTGGCTTGCGCACAGGGAGCGCA	-35.3	Novel-mir-403-5p	-	TGTGAGACCAGGGCCGACTAGGTG	-
Novel-mir-404	AAGTCTGGCTTGTGAGAGACTTTGAGACTCTGAAACTCTTAGGGTGCCCAAACTTTTGCACCATGCCATAATA	-19.7	Novel-mir-404-5p	-	TGTGAGAGACTTTGAGACTCTGA	-
Novel-mir-405	GTCATTTTACTGTGATAGTGTAAAGGCTGGCAAATAGCAACAAACCTGTCATAGGCCATTTTATATATCACTTTTGTTGATTGGCA	-21.8	Novel-mir-405-5p	-	TGTGATAGTGTAAAGGCTGG	-
Novel-mir-406	ACAGCAAAGATGTGGAAGCTGATGTCCTCATGATGGTTTCCAGCGATGGTGAGATGAAGTGGTGGCGTCCGGTGGGTGATGT	-20.1	Novel-mir-406-5p	Novel-mir-406-3p	TGTGGAAGCTGATGTCCTCATGATG	TGAGATGAAGTGGTGGCGTCCGG
Novel-mir-407	GCTCAGCCTGTGTGGACCAGAAGAGTAAAATGAATGATCGTATCAAAAGGGCCTCGCTTTGCAGCACTGGAAATGTGGGCTTATG	-18	Novel-mir-407-5p	-	TGTGGACCAGAAGAGTAAAATGAA	-
Novel-mir-408-1	GGTGGAGTGATGTGGAGTAGTGGAGGTCCGTGGTCCGAAGGAGCAATAGCGGCTGGGAAGACAATTCACGGGGCACAAGGCAGGAACGGAGCTGGGAAGAAA	-23.21	Novel-mir-408-5p	Novel-mir-408-3p	TGTGGAGTAGTGGAGGTCCGTGGT	CACGGGGCACAAGGCAGGAACGGAGC
Novel-mir-408-2	GGTGGAGTGATGTGGAGTAGTGGAGGTCCGTGGTCCGGAGAAGCAATAGCGGCTGACACGGGGCACAAGGCAGGAACGAAGCTG	-21	Novel-mir-408-5p	-	TGTGGAGTAGTGGAGGTCCGTGGT	-
Novel-mir-409	GGGAGCAGAGTGTGGATCTGGGGTGGCAGCGTGATCGTGGTGAGACAATAGGAGCGAGGTGAGTTGTGCTACCTGCTGGGTGAGAGCGGAGATGTCCTG	-24.1	Novel-mir-409-5p	Novel-mir-409-3p	TGTGGATCTGGGGTGGCAGCGTGA	GAGTTGTGCTACCTGCTGGGTGAGAGCG
Novel-mir-410-1	CGTTTAGCGATGTGTATGAATCTTTGTTGATGTGTATTGTATGTCGATGTAGTTAATGATACATGCGTTCATTGTAGGTAAATGC	-18.1	Novel-mir-410-5p	-	TGTGTATGAATCTTTGTTGATGT	-
Novel-mir-410-2	CGTTTAGCGATGTGTATGAATCTTTGTTGATGTGTATTGTATGTCGATGTAGTTAATGATACATGCGTTCATTGTATGTAAATGC	-18.1	Novel-mir-410-5p	-	TGTGTATGAATCTTTGTTGATGT	-
Novel-mir-411	GGGTATGCACTGTGTATGTGGAGCCTGGCAAAGCGAGCTGCTGTATGTTTCATTTCCTTTTTGCACAGAATAAAGTCCTG	-18.44	Novel-mir-411-5p	-	TGTGTATGTGGAGCCTGGCAAAG	-
Novel-mir-412	TCCACGGAGGGGTTGTGTCGGACCAGCCAGGAGTGACCCAATACCAGAGGTGTGTCGGGGACAGCAAAGATATGG	-19.04	Novel-mir-412-5p	Novel-mir-412-3p	TGTGTCGGACCAGCCAGGAGTG	TACCAGAGGTGTGTCGGGGACAGC
Novel-mir-413	CCTCATCTTCTGTGTCTGAATTGAACAGCTGGGCAATTTCGCCATCAAACATGCCGGGTTCCCTCTCATCATTGTCGGAGTCAGTCTCGATGC	-20.1	Novel-mir-413-5p	Novel-mir-270-3p	TGTGTCTGAATTGAACAGCTGGGC	CCCTCTCATCATTGTCGGAGTCA

Novel-mir-414	TGGGGTCTGATGTGTGATGTGGGCAAGCAGCCAGCCGTCAATGGCCTGGGTCTCTATAGCACAGGGTAGCGGCTCCAG	-26.5	Novel-mir-414-5p	-	TGTGTGATGTGGGCAAGCAGC	-
Novel-mir-415	CTGCATGCGTTGTGTGTACGACCGTTTGAAAGCCCATTATAATGTGTGCAGTTTTCACTATTGCTCGGCCAATTGCAGCAACATGCCT	-22.1	Novel-mir-415-5p	Novel-mir-415-3p	TGTGTGTACGACCGTTTGAAAGCCCA	TCACTATTGCTCGGCCAATTGCAG
Novel-mir-416	AAGGTCAGGATGTGTTCTTAGAATTTTACAGATGAAAACTGCCTGGGGACAAAATCATCAAATGAGGGGTCAGAACGAGGTCAACCGACCAA	-19.4	Novel-mir-416-5p	-	TGTGTTCTTAGAATTTTACAGATG	-
Novel-mir-417	TTAGCCGCCATGTGTTGGAGTTTACCCCAGTGAACGAGAGGGTCGCTTCCCTGCGCCTTCGAGTCAAGGATAGGTCTCTCAATGCCATTTGTGCCTAC	-29.4	Novel-mir-417-5p	-	TGTGTTGGAGTTTACCCCAGTGAA	-
Novel-mir-418	TTAGCCGCCATGTGTTGGAGTTTACCCTGGTGAACGAGAGGGGGTCGTTTCCCTGCGCCTTCGGGCCGGGGTTAGGTCTCTCACTGTCGTTTGT	-33.1	Novel-mir-418-5p	-	TGTGTTGGAGTTTACCCTGGTGA	-
Novel-mir-419-1	CTGCCACCGCTGTGTTTGACTGTAAGGATGGTATTAATAAGGTGATGCTCAATGCCTGGTTTTCTCCACACATGCAGTTGGGAACTGTGGCCA	-28.5	Novel-mir-419-5p	-	TGTGTTTGACTGTAAGGATGGT	-
Novel-mir-419-2	TTCCCACCTCTGTGTTTGACTGTAAGGATGGTGTTCTTAGGGTCATACTCAGCATTTTTCTTCCTCCAAACACAGCGAGTCAAGT	-21	Novel-mir-419-5p	-	TGTGTTTGACTGTAAGGATGGT	-
Novel-mir-420	AGGTCCAACATGTTATAGAAGACGACGACTGCCCATCTGTGGGTGGTGGCTCTTGTGGAGTACTGTCTGATCA	-23.8	Novel-mir-420-5p	-	TGTTATAGAAGACGACGACTGCCC	-
Novel-mir-421-1	CTAGAGAGGGTGTTATGAGCATATTCCACCCAGGGCAACAACTGGCTCCAGTCCCCTGGTGAGGAACTGGTAACACAGCGGAGG	-29.8	Novel-mir-421-5p	Novel-mir-421-3p	TGTTATGAGCATATTCCACCCAGG	TGGTGAGGAACTGGTAACACA
Novel-mir-421-2	CTAGAGAGTGTGTTATGAGCATATTCCACCCAGGGCAACAACTGGCTCCAGTCCTCTGGTGAGGAACTGGTGACACAGCGGAGGGCGTTCTCCAGC	-32.7	Novel-mir-421-5p	Novel-mir-421-3p	TGTTATGAGCATATTCCACCCAGG	TGGTGAGGAACTGGTGACACA
Novel-mir-422	TAAAAGTGGGTGTTCAACTATGGCTGTGGATATTGGCCTGTTCCTCTACTCCAGTCGCAGTGGCATCCATAGGCC	-23.2	Novel-mir-422-5p	-	TGTTCAACTATGGCTGTGGAT	-
Novel-mir-423	ACATTAACACTGTTCATCGATGGAGTCCAGGTGGAGAGGGTGGCCGCTTTTGGGTTCCTGGGCGTCATCTTTGAGCAGCACCTGACC	-34.2	Novel-mir-423-5p	-	TGTTCATCGATGGAGTCCAGGTG	-
Novel-mir-424-1	ATTAAGCCACTGTTCATCGATGGAGTCCAGGTGGAGAGGGTGGCCGCTTTTGGGTTCCTGGGCGTCATCTTTGAGCAGCACCTGACTTGGG	-36	Novel-mir-424-5p	-	TGTTCATCGATGGAGTCCAGGTGG	-
Novel-mir-424-2	ATTAAGCCACTGTTCATCGATGGAGTCCAGGTGGAGAGGGTGGCCGCTTTTGGGTTCCTGGGCGTCATCTTTGAGCAGCACCTGACTTGGGGCGCTAATA	-40	Novel-mir-424-5p	-	TGTTCATCGATGGAGTCCAGGTGG	-
Novel-mir-425	AGGTGGGTCTTGTTCTTTGATCGGTTCTGGTTCACCCTCTCCTATTGACCAGGCTCCCGCAATCAAAAACTGGATGCCCTC	-22.54	Novel-mir-425-5p	-	TGTTCTTTGATCGGTTCTGGTTCA	-
Novel-mir-426	TTATATCGGGTGTTGAAGATGTCTGTAAGGGCAGAGGGGCGCCGATGATCTTGCTGGCTGTTTTCACTATGCGTTGTAGG	-26.4	Novel-mir-426-5p	-	TGTTGAAGATGTCTGTAAGGG	-
Novel-mir-427	CTGAGTCAGCTGTTTAGAAGTCTGATGGCAGAGGGAAAGAAAGATTTTTCAGTCTAAAGGTCCTGCATTTCACATTTCTGAGCTTCCGACCGGA	-28.2	Novel-mir-427-5p	-	TGTTTAGAAGTCTGATGGCAGAGG	-
Novel-mir-428	TCCTGTCAGCTGTTTAGGAGTCCGATGGCAGAGGGAAAGAAAGAGTTTTTCAGTCTAGAGGTCCTGCATTTCACACTTCTGAACCTCTGGCCAGAGGGTAG	-29.1	Novel-mir-428-5p	Novel-mir-428-3p	TGTTTAGGAGTCCGATGGCAGAGG	GCATTTCACACTTCTGAACCTCTGGC
Novel-mir-429	CTCAGTCAGCTGTTTAGGAGTCGGATGGCAGAGGGAAAGAAAGAGTTTTTCAGCCTAAAAGTCCTGCATTTCACACTTCTGAGCCTCCGACCGGAG	-25.61	Novel-mir-429-5p	Novel-mir-429-3p	TGTTTAGGAGTCGGATGGCAGAGG	CTGCATTTCACACTTCTGAGCCT
Novel-mir-430	CTGAGTTAGCTGTTTAGGAGTCTGATAGCAGAGGGGAAGGAAGAGTCTTTCAATCTAGAGCTCCTGCATTTCACACTTCTGATCCTCCGACCGAAGG	-23.4	Novel-mir-430-5p	-	TGTTTAGGAGTCTGATAGCAGAGG	-
Novel-mir-431	ACTCCTCAGCTGTTTAGGAGTCTGATCGCAGAGGAAAAGAAACAGTTTTTCAGTCTAGAGGTCCTGAATTTTACACTACTGAACCTCCGGCCAGAGGGCA	-22.2	Novel-mir-431-5p	-	TGTTTAGGAGTCTGATCGCAGAGG	-
Novel-mir-432	AGGTAGATCCTGTTTAGGAGTCTGATGGCAAAGGGAAAGAAAGAGGTTTTCAGTCTAGAGGTCCTGCATTTCACACTTCTGAGCCTCCAACCG	-22.9	Novel-mir-432-5p	-	TGTTTAGGAGTCTGATGGCAAAGG	-
Novel-mir-433-1	ACTCCTCAATTGTTTAGGAGTCTGATGGCAGAGAGAAAGAAAGAGTTTTTCAATCTAGAGGTCCTGCATTTCATACTTCTGAACCTCCGGCCAGAGGGTA	-26.22	Novel-mir-433-5p	-	TGTTTAGGAGTCTGATGGCAGAGA	-
Novel-mir-433-2	ACTCCTCAGCTGTTTAGGAGTCTGATGGCAGAGAGAGTTTTTCAGTCTAGTGGTCCTGCATTTCACACTTATGAACTTCTGGCCAGAGAGTAGGGAGG	-23.51	Novel-mir-433-5p	-	TGTTTAGGAGTCTGATGGCAGAGA	-
Novel-mir-433-3	CTGAGTCAGCTGTTTAGGAGTCTGATGGCAGAGAGAAAGAAAGAGCTTTTCAGTCTAGAGGTCCTGCACACTTCTGAACCTCCGACCGGA	-26.4	Novel-mir-433-5p	-	TGTTTAGGAGTCTGATGGCAGAGA	-
Novel-mir-433-4	GTGGGTCAGCTGTTTAGGAGTCTGATGGCAGAGAGTTTTTCAGTCTAGTGGTCCTGCATTTCACACTTCTGAGCCTCCGACCGGAGGGTA	-28.12	Novel-mir-433-5p	Novel-mir-433-3p	TGTTTAGGAGTCTGATGGCAGAGA	TGCATTTCACACTTCTGAGCCTCCGA
Novel-mir-433-5	CTGAGTCAGCTGTTTAGGAGTCTGATGGCAGAGAGGAAAAAAGAGTTTTTCAGTCCAGTGGTTCTGCATTTCACACTTCTGAACCTCCGGCCAT	-27.5	Novel-mir-433-5p	-	TGTTTAGGAGTCTGATGGCAGAGA	-
Novel-mir-433-6	GTGGGTCAGCTGTTTAGGAGTCTGATGGCAGAGAGTTTTTCAGTCTAGTGGTCCTGCATTTCACACTTCTGAGCCTCCGACCGGA	-28.12	Novel-mir-433-5p	-	TGTTTAGGAGTCTGATGGCAGAGA	-

Novel-mir-434	CTGAGTCAGCTGTTTAGGAGTCTGATGGCAGGGGGAAAGAAAGAGTTTTTCAGTCTAGAGGTCCTGGATTTCACACTGCTGAGCCTCCAACCGGA	-22.9	Novel-mir-434-5p	-	TGTTTAGGAGTCTGATGGCAGGGG	-
Novel-mir-435	CTGAGTCAGCTGTTTAGGAGTCTGATGGCGGAGGGAAAGAAAGAGTTTTTCAGTCTAGTGGTCCTGCATTTCACACTTCTGAACCTCTGGCCAGAAGGTAG	-33.7	Novel-mir-435-5p	-	TGTTTAGGAGTCTGATGGCAT	-
Novel-mir-436	CTGAGTCAGCTGTTTAGGAGTCTGATGGCATAGGGAATGAAAGAGTTTTTCAGTCTAGTGGTCCTGCATTTCACATTTCTGAACCTCCGACTGGA	-23.9	Novel-mir-436-5p	-	TGTTTAGGAGTCTGATGGCATA	-
Novel-mir-437-1	ACTCCTCAGCTGTTTAGGAGTCTGATGGCGGAGGGAAAGAAAGAGTTTTTCAGTCTAGTGGTCCTGCATTTCACACTTCTGAATCTCCGGCCAGAGGGCA	-29.8	Novel-mir-437-5p	Novel-mir-437-3p	TGTTTAGGAGTCTGATGGCGGAGG	TGCATTTCACACTTCTGAATCTCCGG
Novel-mir-437-2	ATGCCTCAGCTGTTTAGGAGTCTGATGGCGGAGGGAAAGAAAGAGTTTTTCAGTCTAGAGGTCCTGCATTTCACACTTCTGAATCTCCGGCCAGAGGGCA	-29.7	Novel-mir-437-5p	Novel-mir-437-3p	TGTTTAGGAGTCTGATGGCGGAGG	TGCATTTCACACTTCTGAATCTCCGG
Novel-mir-437-3	ACTCTTCAGCTGTTTAGGAGTCTGATGGCGGAGGGAAAGAAAGAGTTTTTCAGTCTTTACCACTCTCTGCAGGCTCTTGCGGTCCATAGCGGTA	-29.5	Novel-mir-437-5p	Novel-mir-437-3p	TGTTTAGGAGTCTGATGGCGGAGG	CTCTCTGCAGGCTCTTGCGGT
Novel-mir-438-1	ACTCCTCAGCTGTTTAGGAGTCTGATGGTAGAGGCAAAGAAAGAGTTTTTCAGTCTAGAGGTCCTGCATTTCACACTCCGGCCAGAGTGTAGGAAG	-28.5	Novel-mir-438-5p	-	TGTTTAGGAGTCTGATGGTAGAGG	-
Novel-mir-438-2	ACTCCTCAGCTGTTTAGGAGTCTGATGGTAGAGGGAAAGAAAGAGTTTTTCAGTCTAGTGGTCCTGCATTTCAAACTTCTAAGGGTAGGGAGG	-26.5	Novel-mir-438-5p	-	TGTTTAGGAGTCTGATGGTAGAGG	-
Novel-mir-438-3	ACTCCTCAGCTGTTTAGGAGTCTGATGGTAGAGGGAAAGAAAGAGTTTTTCAGTCTGGAGGTCCTACACTTAACACTTCTGAACCTCCGGTCAGAGGGTA	-25.4	Novel-mir-438-5p	-	TGTTTAGGAGTCTGATGGTAGAGG	-
Novel-mir-438-4	ACTCCTCAGCTGTTTAGGAGTCTGATGGTAGAGGGAAAGAAAGAGTTTTTCGGTCTAGTAGTTGTGCATTTCACACTTCTGAACCTCTGGCCAGAGGGTAG	-28	Novel-mir-438-5p	Novel-mir-428-3p	TGTTTAGGAGTCTGATGGTAGAGG	GCATTTCACACTTCTGAACCTCTGGC
Novel-mir-439-1	ACTCCTCAGCTGTTTAGGAGTCTGATTGCAGAGGGAAAGAAAGAGTTTTTCAGTCTGGAGGTCCTACACTTCTGAACTTGCGGCCAGAGGTTAGGGAGA	-31.3	Novel-mir-439-5p	-	TGTTTAGGAGTCTGATTGCAGAGG	-
Novel-mir-439-2	ACTCCTCAGGTGTTTAGGAGTCTGATTGCAGAGGAAAATAAAGAGTTTTTCAGTCTGGAGGTCCTACACTTCACACTTCTGAACCTTCGGCCAGAAGGTAG	-25.7	Novel-mir-439-5p	-	TGTTTAGGAGTCTGATTGCAGAGG	-
Novel-mir-440-1	CTGAGTCAGCTGTTTAGGAGTGTGATGGCAGTCGGTTTTTCAGCCTAGAGGTCCTGCATTTCACACGTCTGAGCCTCCGACCGGA	-30	Novel-mir-440-5p	-	TGTTTAGGAGTCTGGTGGCAGAGG	-
Novel-mir-440-2	CTGAGTCAGCTGTTTAGGAGTCTGGTGGCAGAGGGAAAGAAAGAGTTTTTCAGTCTAGAGGTCCTGTCTTTCACATTTCTGAGCCTCTGACTGGA	-28.9	Novel-mir-440-5p	-	TGTTTAGGAGTCTGGTGGCAGAGG	-
Novel-mir-441	TCCTGTCAGCTGTTTAGGAGTCTGTTGGCAGAGGGAAAGAAATGGTTTTTCAGCCTAGAGGTCATGCATTTCACACTTCTGAACCTCCGGCCGGAG	-27.6	Novel-mir-441-5p	-	TGTTTAGGAGTCTGTTGGCAGAGG	-
Novel-mir-442-1	ACTCCTCAGCTGTTTAGGAGTTTGATGGCAGAGGGAAAGAAAGAGTTATTCAGTCTAGAGGTCCTACACTTCACACTTCTGAACCTCCGGCCAGAGGGTA	-27.5	Novel-mir-442-5p	-	TGTTTAGGAGTTTGATGGCAGAGG	-
Novel-mir-442-2	TCCTGTCAGTTGTTTAGGAGTTTGATGGCAGAGGGAAAGAAAGAGTTCTTTAGTCTAGACGCCCTGTATTTCACACTTCTGTACCTCTGGCCTGAGGGT	-30.7	Novel-mir-442-5p	-	TGTTTAGGAGTTTGATGGCAGAGG	-
Novel-mir-443	CTGAGTCAGCTGTTTAGGAGTTTGATGGCAGTGGGAAAGAAAGAGTTTTTCAGTCTGGAGGTCCTGCATTTCACACTTCTGAGCCTCTGACCAGAGCACA	-34.4	Novel-mir-443-5p	Novel-mir-182-3p	TGTTTAGGAGTTTGATGGCAGTGG	CATTTCACACTTCTGAGCCTCTGA
Novel-mir-444	AGTAGCCGGGTGTTTGACCAACCGCAGGAGGTGAGGAGATGGAGAGCTGGGCCGAGCTGTGTAGGTGGGGTCACTCCTGGGCTGGTCGAC	-30.2	Novel-mir-444-5p	Novel-mir-444-3p	TGTTTGACCAACCGCAGGAGGTGA	TGTGTAGGTGGGGTCACTCCTGG
Novel-mir-445-1	CTGGAGGAAGTGTTTGGAGAGAGGGAAGTCTGGGCATCTTTGCTTAGACTGCTGCCCCCGCGACCCAGTTCCAGATA	-28.8	Novel-mir-445-5p	-	TGTTTGGAGAGAGGGAAGTCTGG	-
Novel-mir-445-2	CTGGAGGAAGTGTTTGGAGAGAGGGAAGTCTGGGCATCTTTGCTTAGACTGCTGCGCCCGCGACTCGGTCCCGGA	-27.2	Novel-mir-445-5p	-	TGTTTGGAGAGAGGGAAGTCTGG	-
Novel-mir-446-1	ATTAAGTGATTGTTTTAGTGTTGGGACCCTGTAGTTCTACAAGAGGGTCTGGCAAGGATGTAATAGTTGTTGTCT	-18.9	Novel-mir-446-5p	-	TGTTTTAGTGTTGGGACCCTGTAG	-
Novel-mir-446-2	ATTACGAGATTGTTTTAGTGTTGGGACCCTGTAGTTCTACAATAGGGTCTGGCAAGGATGTAATAGTTGTTGT	-20.3	Novel-mir-446-5p	-	TGTTTTAGTGTTGGGACCCTGTAG	-
Novel-mir-446-3	ATTGGTTGATTGTTTTAGTGTTGGGACCCTGTAGTTCCACAATAGGGTCTGGCAAAGATGTAATAGGAGTTGTTTAGATTTTCATTTCCATA	-20.9	Novel-mir-446-5p	-	TGTTTTAGTGTTGGGACCCTGTAG	-
Novel-mir-447	TGAAGAGTTTTTAAGATTGATAGATAAGCATGCACCGTTCCGCTCTTTTAGAGTGAAGGGGCGTGATAATCTTTGGTTTTTGTTGGAAATTGGTAA	-26.3	Novel-mir-447-5p	-	TTAAGATTGATAGATAAGCATGCA	-
Novel-mir-448	TGAAGAGTTTTTAAGATTGATAGATAAGCATGCGCCGTTCCGCTCTTTTAGAGTGAAGGGGCGTGATAATCCTTGGTTTTCGTTGGAAATTGGTAA	-23.9	Novel-mir-448-5p	-	TTAAGATTGATAGATAAGCATGCG	-
Novel-mir-449-1	GGCCTGTTCTTTACAATTAGAAGCTGAGGTGGGAGCTCAGTCCTGTTTTCTCTAATTGTGCCAACCATTGTCAGCTTTCTTTTGAGAAGTTCCTGTCC	-26.2	Novel-mir-449-5p	-	TTACAATTAGAAGCTGAGGTGGGA	-
Novel-mir-449-2	GGCCTGTTCTTTACAATTAGAAGCTGAGGTGGGAGCTCAGTCCTGTTTTTCCTAATTGTGCCAACCATTGTCAGCTTTCTTTTGAGAAGTTCCTGTCC	-25.82	Novel-mir-449-5p	-	TTACAATTAGAAGCTGAGGTGGGA	-

Novel-mir-449-3	GGCCTGTTCTTTACAATTAGAAGCTGAGGTGGGAGCTCAGTCCTGTTTTTTCTAACTGTGCCAACCATTGTCAGCTTTCTTTTGAGAAGTTCCTGTCC	-28.5	Novel-mir-449-5p	-	TTACAATTAGAAGCTGAGGTGGGA	-
Novel-mir-449-4	GGCCTGTTCTTTACAATTAGAAGCTGAGGTGGGAGCTCAGTCCTGTTTTTTCTAATTATGCCAACCATTGTCAGCTTTCTTTTGAGAAGTTCCTGTCC	-22.6	Novel-mir-449-5p	-	TTACAATTAGAAGCTGAGGTGGGA	-
Novel-mir-449-5	GGCCTGTTCTTTACAATTAGAAGCTGAGGTGGGAGCTCAGTCCTGTTTTTTTCTAATTGTGCCAACCATTGTCAGCTTTCTTTTGAGAAGTTCCTGTCC	-25.74	Novel-mir-449-5p	-	TTACAATTAGAAGCTGAGGTGGGA	-
Novel-mir-449-6	GGTCTGTTCTTTACAATTAGAAGCTGAGGTGGGAGCTCAGTCCTGTTTTTTCTAATTGTGCCAACCATTGTCAGCTTTC	-18.8	Novel-mir-449-5p	Novel-mir-449-3p	TTACAATTAGAAGCTGAGGTGGGA	TGTTTTTTCTAATTGTGCCAACCATT
Novel-mir-450	TGAGTTAAGTTTACATTCCAGGCTGACATCCTCTAACATTAGTAAGCTTACTTCTTAATCACACCGTTTCGGTTGTAACAAGCCAGAATGTCACTCAT	-18.3	Novel-mir-450-5p	-	TTACATTCCAGGCTGACATCCTCT	-
Novel-mir-451	AGCAGGATGTTTACTGGAAGTCTGAGCACAGGTATAACAATACAGCGCTGGGATCAAAGAGACAGAGCTCCCGCA	-20	Novel-mir-451-5p	-	TTACTGGAAGTCTGAGCACAGGTA	-
Novel-mir-452	TTCTGGTGGCTTAGATGTTGTGGGAGCAGTGGCTGCAGCATGCCGAAGTTTCATGTACTTCTCGTACTGGAGT	-20.2	Novel-mir-452-5p	Novel-mir-452-3p	TTAGATGTTGTGGGAGCAGTGGC	CGAAGTTTCATGTACTTCTCGTA
Novel-mir-453	GACACGTACATTAGTGAGTGTACAGGGCGATGGGAAAGTGAATCAAGCAAACAAACACATGATACTGTACCTCTGTGTTCTCACTGTAATGTGATATGTG	-26.73	Novel-mir-453-5p	-	TTAGTGAGTGTACAGGGCGATGG	-
Novel-mir-454	AAAAAGCCGGTTATCAGGTTGGGAGCTGTCGAGCACATAACTGCAGCAGCAGACTGGTGCTTTGCCCCTTTAGCATGGCTAGGC	-28.9	Novel-mir-454-5p	-	TTATCAGGTTGGGAGCTGTCGAGC	-
Novel-mir-455	GTGACTGAACTTATCGCACACAGCCCACTTAAGCGCCAAAAACTCCAGTCTGTGAGCTAGGTATTGTGACTGGGCATGGTTGAGTGACT	-20.5	Novel-mir-455-5p	-	TTATCGCACACAGCCCACTTAAGC	-
Novel-mir-456	AATCTGGCTTTTATGGAAGAGTGACAAGAAGAACGCCATTGTTGAAAGAAAGGAATAAGAAGTCCTGTTTGCAGTTTGCCACAAGCCACGTA	-18.5	Novel-mir-456-5p	-	TTATGGAAGAGTGACAAGAAGAA	-
Novel-mir-457	AATCTGGCTCTTATGGAAGAGTGGCAAGAAAGAAAGCAATAAGAAGTCCTGTTTGCAGTTTGCCACAAGCCATGTAGGGGACACAGCAA	-24.5	Novel-mir-457-5p	-	TTATGGAAGAGTGGCAAGAAA	-
Novel-mir-458	GGTGTCTTGTTTATGTCAAAACTAAACATGTAGCCATTTCAACCTGCTACATGTGCGTAGACGTCACACACACGCACCG	-23.7	Novel-mir-458-5p	-	TTATGTCAAAACTAAACATGTAGC	-
Novel-mir-459	GAACAGTAGCTTCAACGTGATCGACAGGCATGAGTGAGACCGATCAGCGTTACCATGTCGGCTGTGTGGAGTTATTTTAA	-22	Novel-mir-459-5p	-	TTCAACGTGATCGACAGGCATGAG	-
Novel-mir-460	GTTTTGCTAGTTCACACGAATTCCAAACCGGGTGCATGCCGAAATTCATATTTTCTTTTTGGGAGACTGGCTGGTTCGTTGGACGGGATTAAATGTA	-22.1	Novel-mir-460-5p	Novel-mir-460-3p	TTCACACGAATTCCAAACCGGGTG	TGGCTGGTTCGTTGGACGGG
Novel-mir-461-1	TGTCACAGACTTCCAACTCACGAACAGGTACACCATAAAATAAGCCAAAGTCCAAGGTGTGCCCATGCTCATGGGTAGGACCAGCCACAGATTATGTGAGA	-25.8	Novel-mir-461-5p	-	TTCCAACTCACGAACAGGTACACC	-
Novel-mir-461-2	CGTCACAGATTTCCAACTCACGAACAGGTACACCATAAGATAAGACAAGGTCCGAGGTGTGCCCATGCTCATGGGTAGGACCAGCCATAGATTGTATGAAA	-25	Novel-mir-461-5p	-	TTCCAACTCACGAACAGGTACACC	-
Novel-mir-462	CCTCTGGGTCTTCGTCTGTCCCCAAGGTGAACGTGTCGTATTTGTCTTGGGCGAGGTTGAGGAACGTGTAAGCTCGCACCTTCGTGGA	-24.9	Novel-mir-462-5p	-	TTCGTCTGTCCCCAAGGTGAAC	-
Novel-mir-463-1	GTGTGTGTCTTTCTAAACTCTGTCCAGTCGGCCCAGTTTGCCACAGGTGGATCCAGTCTGGTTCTAGACACACTT	-25.2	Novel-mir-463-5p	-	TTCTAAACTCTGTCCAGTCGGCCC	-
Novel-mir-463-2	GTGTGTGTCTTTCTAAACTCTGTCCAGTCGGCCCAGTTTGCCACAGGTGGATCCAGTCTGGTTCTAGACACGTCAG	-22.4	Novel-mir-463-5p	-	TTCTAAACTCTGTCCAGTCGGCCC	-
Novel-mir-464	CAGATACTCATTCTGATCCTCGGCCTCTCTGTCGATGTCGTATGCCAGCTGTCAGATACACAGACAGGACAGATGTTGTTATGTTAAAATAGATTAACTGA	-18.5	Novel-mir-464-5p	-	TTCTGATCCTCGGCCTCTCTGTCG	-
Novel-mir-465	ATGGCAGTCCTTCTGTGTTATGGTGAACGATGGTTAGAGTGTACGATCTGCTCAGTGTTTGTCTGATCTGCAGAAGCAACATCATGTCATC	-24.3	Novel-mir-465-5p	Novel-mir-465-3p	TTCTGTGTTATGGTGAACGATGGT	CAGTGTTTGTCTGATCTGCAGAAGC
Novel-mir-466-1	GCTGAAGCCATTCTGTTGTGGACTTGCTCTGGTGTTTTGGGTCATTGTCCTGTTGCATCACCCAACTTCTACGATTTCAGCT	-24	Novel-mir-466-5p	-	TTCTGTTGTGGACTTGCTCTGG	-
Novel-mir-466-2	TCTGAAACCATTCTGTTGTGGACTTGCTCTGGTGTTTTGGGTCATTGTCCTGTTGCATCACCCAACCTCTACAGAGTTTCAGC	-24.9	Novel-mir-466-5p	-	TTCTGTTGTGGACTTGCTCTGG	-
Novel-mir-466-3	TCTGGAGCCATTCTGTTGTGGACTTGCTCTGGTGTTCTGGGTCATTGTCCTGTTGCATCACCCAACTTCTACAGAGTTTCAGC	-23.5	Novel-mir-466-5p	-	TTCTGTTGTGGACTTGCTCTGG	-
Novel-mir-467	TGACCTCTTCTTCTTAGGTGCTGGTGGTGGCAGTGGCACTGCCACTGTTTCAGGACTTCAGTGTTTTCTTCCG	-22.9	Novel-mir-467-5p	-	TTCTTAGGTGCTGGTGGTGGC	-
Novel-mir-468	GAGGGTCTTCTTCTTGGAAGTGGTCTCAAGCTGCATCACCTCATGTTGGGTATTGACCTCTTCACTGTGTCCCTCTGTGAT	-22.3	Novel-mir-468-5p	Novel-mir-468-3p	TTCTTGGAAGTGGTCTCAAGCTGC	TTGGGTATTGACCTCTTCACTGTGTC
Novel-mir-469-1	GGGGCGACCGTTCTTTTGCTGTGTATGGCCCCAGACTTTGGAACTCTCTACCGCAAGAGTTACAGTGCATAACCG	-18.4	Novel-mir-469-5p	-	TTCTTTTGCTGTGTATGGCCC	-

Novel-mir-469-2	GGGGCGACCGTTCTTTTGCTGTGTATGGCCCCAGACTTTGGAACTCTCTACCGCAAGAGTTACGGTGCATA	-22.3	Novel-mir-469-5p	-	TTCTTTTGCTGTGTATGGCCC	-
Novel-mir-469-3	GGGGCGACTGTTCTTTTGCTGTGTATGGCCCCAGACTTTGGAACTCTCTACCGCAAGAGATACGGTGCGTAACCA	-24.2	Novel-mir-469-5p	-	TTCTTTTGCTGTGTATGGCCC	-
Novel-mir-470	CCACTCTCTTTTGAAGATCTCTGTACAAAGTCCCGAGTACACAGACGCCTCTGAGACATCCAGACCATGCTGCTCCAGGTCTTCTTGGTAGAACAT	-22.1	Novel-mir-470-5p	-	TTGAAGATCTCTGTACAAAGTCC	-
Novel-mir-471	CAGTGATCTATTGAAGATCTGTGTGAAGATGGGGGCCAGCTGATCAGGACAGGTTTTTGGGCCGGAGGGGGTCACAC	-24.1	Novel-mir-471-5p	-	TTGAAGATCTGTGTGAAGATGGGG	-
Novel-mir-472-1	CAGTAACCTGTTGAAGATGAATGTGAAGATGGGGGCCAGCTGATCAGCACACACTTTCAGGCAGGAGGGGG	-19.5	Novel-mir-472-5p	-	TTGAAGATGAATGTGAAGATGGG	-
Novel-mir-472-2	CAGTGACCTGTTGAAGATGAATGTGAAGATGGGGGCCAGCTGATCAGCACACACTTTCAAGCAGGAGGTGTCACACC	-22	Novel-mir-472-5p	Novel-mir-472-3p	TTGAAGATGAATGTGAAGATGGG	GATCAGCACACACTTTCAAGCAGGAG
Novel-mir-472-3	CAGTGACCTGTTGAAGATGAATGTGAAGATGGGGGCCAGCTGATCAGCGCACACTTTCAAGCAGGAGGTGTCACACC	-21.6	Novel-mir-472-5p	Novel-mir-472-3p	TTGAAGATGAATGTGAAGATGGG	TCAGCGCACACTTTCAAGCAGGAG
Novel-mir-473	AGGGATAAGCTTGAAGTATCTTGGAAGGCCGGTCCTGCTCCTTAATGGCATCAGAGTCAATGAAGGCCCGTCTTGCTTGGAATTCAAGGTCCAT	-26.7	Novel-mir-473-5p	-	TTGAAGTATCTTGGAAGGCCGGTC	-
Novel-mir-474	ATCTGCAAACTTGACAATGTGGTTGGGGCTGTAGGTTGGCACACAGTCCTAGGTCAGCAGTGAGAAGAGCAATGGGCTGAGCACACACCCCTGCGGAG	-29.3	Novel-mir-474-5p	-	TTGACAATGTGGTTGGGGCTGTAG	-
Novel-mir-475	CTGCTTGCCCTTGAGAAATGTGGTAATGTAGCCTTTGAAGAGAGAATCGCTCTGTTCTTTGAAATGCCACACCTCGCTTATTTCAACGAGTTT	-20.7	Novel-mir-475-5p	-	TTGAGAAATGTGGTAATGTAGC	-
Novel-mir-476-1	CCTGTGCTTCTTGAGTAAGTTGGTGACTGAGGCCCACACCTCACCCCTCTGAGCTCTGGGTATACACT	-18	Novel-mir-476-5p	-	TTGAGTAAGTTGGTGACTGAGGC	-
Novel-mir-476-2	CCTGTGTTCCTTGAGTAAGTTGGTGACTGAGGCCCACACCTCACCCCTCTGAGCTCTGGGTATACACTTGAGGT	-22.6	Novel-mir-476-5p	-	TTGAGTAAGTTGGTGACTGAGGC	-
Novel-mir-476-3	TCTCTGTTCCTTGAGTAAGTTGGTGACTGAGGCCCACACCTCACCCCTCTGAGCTCTGGGTATACACTTGAGGT	-20.5	Novel-mir-476-5p	-	TTGAGTAAGTTGGTGACTGAGGC	-
Novel-mir-477	CTCAGTCCCTTTGATGGTCAGAGGCAGCAGAGGAGAGGGGTTCTTCCTGAAGTGTGCTATTATTTTTTTTTGTCTTGTTTATGTTGAGGATGAGG	-22.4	Novel-mir-477-5p	-	TTGATGGTCAGAGGCAGCAGAGGA	-
Novel-mir-478	CTCCGTCCCTTTGATGGTCAGAGGTAGCAGAGGGAAGGGGTTCTTCCTGACATCTACTGTTATTTCTGTTGTCTTGTTTATATTGAGGATGAGGT	-29.74	Novel-mir-478-5p	-	TTGATGGTCAGAGGTAGCAGAGGG	-
Novel-mir-479	GATGCAGCAGTTGATGTTGACTTTAAGCTGGACTGTGAAGATCTACTGGATTTTTTTGATGTCTGTGATCTAGCATCACTTGCTCCAAA	-23.5	Novel-mir-479-5p	-	TTGATGTTGACTTTAAGCTGGACT	-
Novel-mir-480-1	ACTTGCTGTTTTGCTATTTGCTGTGGACATCTGAGATAAAGAAATTCTCTGAAGGTGTAAGTCAGTAAATCTAAAATGTATAT	-19	Novel-mir-480-5p	-	TTGCTATTTGCTGTGGACATCTGA	-
Novel-mir-480-2	ACTTGCTGTTTTGCTATTTGCTGTGGACATCTGAGATAACGAAATTCTCTGAAGGTGTAAGTCAGTAAATCTAAAATGTATAT	-19	Novel-mir-480-5p	-	TTGCTATTTGCTGTGGACATCTGA	-
Novel-mir-481	CGCAGGCAGCTTGGAAAGATGAAAGACCCTGGACCAAGAAACCTAGATAATTTTAATCATTCTGGGCTAACCCCATACTACGCAGGCA	-19.21	Novel-mir-481-5p	-	TTGGAAAGATGAAAGACCCTGGAC	-
Novel-mir-482	CTAAGAGGATTTGGAAAGTCGCCAGATTTAGCGAGAAAGTCGCCAAGTTGGCAACACTGCCACAGTTCATGGGT	-19.2	Novel-mir-482-5p	-	TTGGAAAGTCGCCAGATTTAGC	-
Novel-mir-483-1	CTGGTGCATGTTGGAAGTCAATCTGGAATCGTGGTCTCCATTTCTTGGTCCGTGCCATCAGATCTCAGATGATTCAGTCACACAGCAGCTGTAACATA	-26.2	Novel-mir-483-5p	Novel-mir-483-3p	TTGGAAGTCAATCTGGAATCGTGG	TCTCAGATGATTCAGTCACACAGCAG
Novel-mir-483-2	GACTCGCATGTTGGAAGTCAATCTGGAATCGTGGTCTCCATTTCTTGGTCCGTGGCATCAGATCTCAGATGATTCAGTCACACAGCAGCTGTAACACA	-22.5	Novel-mir-483-5p	Novel-mir-483-3p	TTGGAAGTCAATCTGGAATCGTGG	TCTCAGATGATTCAGTCACACAGCAG
Novel-mir-484	TAGAAAGGTGTTGGACATACTAATAGAAACGGGGACTAACTTTCCTGTCTCTCTTCTTGTGTCCAGTGCTGAGTAT	-27.6	Novel-mir-484-5p	-	TTGGACATACTAATAGAAACGG	-
Novel-mir-485	GCCAACAGCATTGGACATCAATAAAGGCACATAAAACTCTCATTTAGTAGGCGAGTTCCTGATGATTTTTTGTTTGATGTCCACAAAAGCTTGTGTAG	-23	Novel-mir-485-5p	-	TTGGACATCAATAAAGGCACATAA	-
Novel-mir-486	CCGGTTCCTTTTGGACCTTCAATCTAAGGGCGGGGATCTACGTGTGCTTGGACGTGACGGACGAGGAACTGT	-27.3	Novel-mir-486-5p	Novel-mir-486-3p	TTGGACCTTCAATCTAAGGGCGGG	TGTGCTTGGACGTGACGGACGAGG
Novel-mir-487	CAGTGACATGTTGGAGATAAGTGTGAAGATGGGGGCCAGCTGATCAGCACACACTTTCAGGCAGGAGGGGGTCATGC	-20.9	Novel-mir-487-5p	-	TTGGAGATAAGTGTGAAGATGGG	-
Novel-mir-488	GTGAAAAAACTTGGAGTGATATTTGATAGCGGCCCTAATTTTGATATGCAAATCAGGGCCATGGTTAAAACCACCTTTTTTTACC	-23.1	Novel-mir-488-5p	-	TTGGAGTGATATTTGATAGCGGC	-
Novel-mir-489	TATCTGTATTTTGGATTGCATCTGGAGCAGGTTTATTGAGTGAACTGCTGTTTAACATTTGTTCCATGTATCTGTGTTTGTGCTGATAC	-20.7	Novel-mir-489-5p	-	TTGGATTGCATCTGGAGCAG	-

Novel-mir-490	GACAGTGTGCTTGGGTGCAGGGAGAAGATGTGGGGAGATCTTAAACACTCATCTATCCACTGCACTCTGACTTTCTGTCT	-30.2	Novel-mir-490-5p	-	TTGGGTGCAGGGAGAAGATG	-
Novel-mir-491	AGATGCTCAATTGGGTTTAAGTCGGGGCTCTGGCTGGGCCATTCAAGAACAGTCACAGAGTTGTTGTGAAGCCACTCCTTCGTTA	-20	Novel-mir-491-5p	-	TTGGGTTTAAGTCGGGGCTCTGGC	-
Novel-mir-492	CCAGTTGTGTTTGGTGATTTTTCTGTAGACCCACTGCTACGGCAGGTGCCTGTTCCTTGGTGTCATCTGTTGAGAGATGCTCATCACAGTGT	-23.5	Novel-mir-492-5p	Novel-mir-492-3p	TTGGTGATTTTTCTGTAGACC	TCTGTTGAGAGATGCTCATC
Novel-mir-493	TATAGTCCCTTTGGTGGTCAGAGGCAGCAGAGGGGAGGGGTTCTTTCTGAAGTCTATTATTATTTCTTTTGTCTTGTTTATGTTGAGGTCATTTT	-18.9	Novel-mir-493-5p	-	TTGGTGGTCAGAGGCAGCAGAGGG	-
Novel-mir-494	CACCCTGCGGTTGTAAATGCTCTGCAGAGAGGGCAGTGGAGTCCTGGTGATCTTCTCCGCAGTCTTGACCACTCTCTGCAGGCGATTGAGGTCCATAGCAG	-38.8	Novel-mir-494-5p	-	TTGTAAATGCTCTGCAGAGAGGGC	-
Novel-mir-495-1	GAGGTGGAGGTTGTAGAGCTGGTGGCAGAGCTTGCTTAGTTTCTGCCCCTGCTCCGCAACAACGTCGGGTGTGTCCGCGGG	-34.8	Novel-mir-495-5p	-	TTGTAGAGCTGGTGGCAGAGC	-
Novel-mir-495-2	GAGTTGGAGGTTGTAGAGCTGGTGGCAGAGCTTGCTAAGCTTCTGCCCCAGCTCCGCAACATCATCG	-37.4	Novel-mir-495-5p	-	TTGTAGAGCTGGTGGCAGAGC	-
Novel-mir-496	CAGTGACCTGTTGTAGATAAGTGTGAAGATGGGGGCCAGCTGATCAGCACAGACTTTCAGACAGGAGGGGGTCG	-18.4	Novel-mir-496-5p	-	TTGTAGATAAGTGTGAAGATGGG	-
Novel-mir-497	ACAGGCTCCATTGTAGGACCTCTGGGGTAGACGGGGCTTCTCAGTCTAGCCAGAAGCTAGGGGGACCCGGGAGGAACGCAGAGGAAGGGGACCAGGGGCCC	-41.4	Novel-mir-497-5p	-	TTGTAGGACCTCTGGGGTAGACGG	-
Novel-mir-498-1	CATATCTCATTTGTATTGTGTGGCATTCATGGCAAAGTAAGATTTACATTGTCCTAACTGTGCACATGATCTGTATAATATTTAGAGGCGTT	-23.2	Novel-mir-498-5p	-	TTGTATTGTGTGGCATTCATGGCA	-
Novel-mir-498-2	TATATGTCATTTGTATTGTGTGGCATTCATGGCAAAGTAAGATTTACATTGTCCTAACTGTGCACATGATCTGTATAATATTTAGAGGTGTT	-23.2	Novel-mir-498-5p	-	TTGTATTGTGTGGCATTCATGGCA	-
Novel-mir-498-3	TATATTTCATTTGTATTGTGTGGCATTCATGGCAAAGTAAGATTTACATTGTCCAAACTGTGCACATGATCTGTATAATATTTAGAGGTCTT	-21.5	Novel-mir-498-5p	-	TTGTATTGTGTGGCATTCATGGCA	-
Novel-mir-498-4	TATATTTCATTTGTATTGTGTGGCATTCATGGCAAAGTAAGATTTACATTGTCCTAACTGTGCACATGATCTGTATAATATTTAGAGGCGTT	-20.6	Novel-mir-498-5p	-	TTGTATTGTGTGGCATTCATGGCA	-
Novel-mir-498-5	TATATTTCATTTGTATTGTGTGGCATTCATGGCAAAGTAAGATTTACATTGTCCTAACTGTGCACATGATCTGTATAATATTTAGAGGTGTT	-23.9	Novel-mir-498-5p	-	TTGTATTGTGTGGCATTCATGGCA	-
Novel-mir-498-6	TATATTTTATTTGTATTGTGTGGCATTCATGGCAAAGATTTACATTTTCCTAACTGTGCACATGATCTGTATAATATTTAGAGGTGTT	-18.29	Novel-mir-498-5p	-	TTGTATTGTGTGGCATTCATGGCA	-
Novel-mir-499	TTTCCAGTTTTTGTGAATGAAGAATGCTGTGTGGACATTCTGTTTGGAGGTGCAGCTGCAATTTACAGGACCTGGAGAA	-26	Novel-mir-499-5p	-	TTGTGAATGAAGAATGCTGTGTG	-
Novel-mir-500	AGCCACTCCTTTGTTATCTTGGCTGTGGTCTTAGGGTCATTGTCCTGTTGAAAGATAAACTGTCGCCACAGTCTGAGGTCCAGAGTGACC	-27	Novel-mir-500-5p	-	TTGTTATCTTGGCTGTGGTCTTAG	-
Novel-mir-501	TACAGTACGGTTGTTATTCACGTCGGCGCTAATGACTCCCGACTTCGCCAGTCGGAGGTCACTAAGATTAATGTTGAGTCAGTGTGTAACTTCGCTAAAAC	-27.4	Novel-mir-501-5p	Novel-mir-501-3p	TTGTTATTCACGTCGGCGCTAATGAC	TTAATGTTGAGTCAGTGTGTAACT
Novel-mir-502	CTTATGACCTTTGTTGAACTTATAGCCAGGCATCTCAAGGATCACGGCTGCTGTGGTGTATATCAGCTTGTTGGTCTCAGT	-19	Novel-mir-502-5p	-	TTGTTGAACTTATAGCCAGGC	-
Novel-mir-503	TGGCATTGCATTGTTGTGTTCATTGTGCCAGGTTGACCTGTAAAATGACATGAACTGATGGACTGGAATGTTGTACAGTATGGACCTACTGCATAATGC	-22.3	Novel-mir-503-5p	-	TTGTTGTGTTCATTGTGCCAGG	-
Novel-mir-504	CAGTTCATGTTTGTTTTGACTGCTTGAGGCCGGTGTGTCTGCCGCATGTGATGTGTTGCTGGTTAACAAGCAGATTAATAATCCATGATGTC	-25.5	Novel-mir-504-5p	-	TTGTTTTGACTGCTTGAGGCCGGT	-
Novel-mir-505	GTATGGTACATTTACGTACTGGGGAACAGTGGGGCGTGGCTCGCTCTGCAGATTGGCCGACTGTAACTTCGGCGTTATGAACCTGAA	-27.1	Novel-mir-505-5p	-	TTTACGTACTGGGGAACAGTGGG	-
Novel-mir-506	GATTCAGCCCTTTAGTTCACATTTCCATCTGTGGCGCTCGATTGCCGGGTTGCTGCTCATCCACGATGGCTCTGTTGCTAGGGAGACTGCCGC	-26.3	Novel-mir-506-5p	-	TTTAGTTCACATTTCCATCTGTGG	-
Novel-mir-507	CAGAATCTGCTTTATTGGCCAGGTGTGTCGGGCACACGAGGAATTTGACTCTGGTACATCGGCTCTTAATGTGCATGTACAT	-20.1	Novel-mir-507-5p	-	TTTATTGGCCAGGTGTGTCGGGCA	-
Novel-mir-508	CAGAATCTGCTTTATTGGCCGGGTGTGTTGGACACATGAGGAATTTGACTCTGGTACTGTACATCGGCTGTCAATGTGCAAACAGTA	-21.5	Novel-mir-508-5p	-	TTTATTGGCCGGGTGTGTTGGACA	-
Novel-mir-509-1	GGGGGATAAGTTTCATGTCCATACTGAGGAACCCCTGCTTTAGGCTGTTTTTAATGTGGGGGTGGTGGTGAGTTGTTATATGGGCACTGAGCAGCCCAATGTC	-31.3	Novel-mir-509-5p	Novel-mir-509-3p	TTTCATGTCCATACTGAGGAACCC	TGAGTTGTTATATGGGCACTGAGCA
Novel-mir-509-2	GGGGGATAAGTTTCATGTCCATACTGAGGAACCCCTGCTTTAGGCTGTTTTTAATGTGGGGGTGGTGGTGAGTTGTTATATGGGCACTGAGCAGCCCAATGTCC	-31.3	Novel-mir-509-5p	Novel-mir-509-3p	TTTCATGTCCATACTGAGGAACCC	TGAGTTGTTATATGGGCACTGAGCAG
Novel-mir-510-1	AAGCAGCACTTTTCTGATTGGGCTTGTTGCTTGGGTGAATTCCGTACAAACCGGCTGGCTGTGTGAAGGGAGGTGGTGTTTA	-25.4	Novel-mir-510-5p	-	TTTCTGATTGGGCTTGTTGCTTGG	-

Novel-mir-510-2	AAGCCGCACTTTTCTGATTGGGCTTGTTGCTTGGGTGAATTCCATACCAACCAGCTGGCTGTTGAGAAGGGAGGTGATGTTTA	-26.8	Novel-mir-510-5p	-	TTTCTGATTGGGCTTGTTGCTTGG	-
Novel-mir-508-1	CTGTGGCCCCTTTGAAGATGTTCCAGTCTGTGGCCTCTAGACAGCCCCGCAGGCAGCTCTTGCACAGTCGTTCCAGG	-22.7	Novel-mir-508-3p	-	TTTGAAGATGTTCCAGTCTGTGGC	-
Novel-mir-508-2	CTGTGGCCCCTTTGAAGATGTTCCAGTCTGTGGCCTCTAGACAGCCCTACAGGCTGGCCTGTCGTTCCAGGTGTTAACAGT	-28.5	Novel-mir-508-3p	-	TTTGAAGATGTTCCAGTCTGTGGC	-
Novel-mir-508-3	CTGTGGCCCCTTTGAAGATGTTCCAGTCTGTGGCCTCTAGAGAGCCCTGCAGGCTGGCCTATGCACAGTTGTTCCAGT	-26.5	Novel-mir-508-3p	-	TTTGAAGATGTTCCAGTCTGTGGC	-
Novel-mir-511	CTCAACCCACTTTGAGAAGTAGTCAGTGGCTGTCAAAATATACTGAAAGCCATCAGCAGTTTTGGGTAAGGG	-18.1	Novel-mir-511-5p	-	TTTGAGAAGTAGTCAGTGGCTGT	-
Novel-mir-512	GGAGCGGAGATTTGCTAGGTGGATAGATGGGAGGTGCCTGCTCGTTTGCCACGGCAACATTTCTTCCGGGTCCC	-27.3	Novel-mir-512-5p	-	TTTGCTAGGTGGATAGATGGG	-
Novel-mir-513	GGAGCGAATATTTGCTAGGTGGATGGACGGGAGTGGAGTCCAGGTCCCTCTCTCTCGTAGCCTTACTAAGGCGCCCA	-24.6	Novel-mir-513-5p	-	TTTGCTAGGTGGATGGACGGGA	-
Novel-mir-514	ATGGCGAAGATTTGGACCAAAAGGAGACTGTTTATAGATATATTCATGTCTTTTATGTTTTCCTGAGTCACACAAGATTTGACCAA	-19.7	Novel-mir-514-5p	-	TTTGGACCAAAAGGAGACTGTTTA	-
Novel-mir-515-1	CCTGTCAGCATTTGGATTAACTGGAGACTTTTTAGGGAACTTTTGGGACATCCTGATAGTAAAGAGTTACAGTAGTCCAGCCTGGAAGT	-26.6	Novel-mir-515-5p	-	TTTGGATTAACTGGAGACTTTTTA	-
Novel-mir-515-2	GCTGCAGCAATTTGGATTAACTGGAGACTTTTTAGGGAATTATTGGGACATCCTGATAGTAAAGAGTTACAGTAGTCCAGCCTGGAAGTA	-27.9	Novel-mir-515-5p	-	TTTGGATTAACTGGAGACTTTTTA	-
Novel-mir-516	GATGAAGCTGTTTGTAAGTCTGGTGGAGTGGGTCTGAAGACTCCAGTACCTTCTCCCTGAAGGCAGGAGTCTGAAGTGGGA	-20.5	Novel-mir-516-5p	Novel-mir-516-3p	TTTGTAAGTCTGGTGGAGTGGGTC	TACCTTCTCCCTGAAGGCAGGAGTC
Novel-mir-517	TCTGGACATCTTTGTTGGTAGGTTTGTTGCGGAAATATTCTGTGATCACTTTCCTAGATTCCTCCATGGCAAGCTCCAGGAGACGTGGCACATT	-24	Novel-mir-517-5p	-	TTTGTTGGTAGGTTTGTTGCGG	-
Novel-mir-518	ATCCACTCTCTTTTGAAGATCTCTGTACAAAGTCCCGAGTACACAGACGCCTCTGAGACATCCAGACCATGCTGCTCCAGGTCTTCTTGGTAGAACATGA	-22.1	Novel-mir-518-5p	-	TTTTGAAGATCTCTGTACAAAGTC	-
Novel-mir-519	TGTTTCAAACTTTTGAGTGCGACGCAATGCGGCATCCACACAATTTGAGTGCAATGCAATGCGATGTCCTCCCCAGTTGGGTGCGGT	-24.7	Novel-mir-519-5p	Novel-mir-519-3p	TTTTGAGTGCGACGCAATGCGGC	TGCAATGCGATGTCCTCCCCAGT
Novel-mir-520-1	GTGCTGCAGTTTTTGGTTTGTTGCACCATGTCTTGATCGTGTTAATAATCAGACTACTGGAGGAGATGAGCTGAAGGCTGAATCAGTGCA	-31.8	Novel-mir-520-5p	-	TTTTGGTTTGTTGCACCATGTCT	-
Novel-mir-520-2	GTGCTGCAGTTTTTGGTTTGTTGCACCATGTCTTGATTGTGTTAATAATCAGACTACTGGAGGAGATGAGCTGAAGGCTGAATCAGTGCA	-35	Novel-mir-520-5p	-	TTTTGGTTTGTTGCACCATGTCT	-
Novel-mir-521	GAATGCCTGTTTTTGTTCACAGTGCCTACCAGGCTGATGTTCCTTACTAGGTGTTTATGAACAAGGGGGAGCGATGTA	-20.3	Novel-mir-521-5p	Novel-mir-521-3p	TTTTGTTCACAGTGCCTACCAGGCT	TACTAGGTGTTTATGAACAAGGGG
Novel-mir-522	AAGTTTCCTGTTTTTCAGTACAGGCTGTAGGGGGGTCCCAAAATAGAGAAGTGAGAAGGGTGGGGGCCC	-18.1	Novel-mir-522-5p	-	TTTTTCAGTACAGGCTGTAGG	-
Novel-mir-1042	CCCTATATTCCCACTCGTCACCCCCAGATACACATCCAACAATGGCGGTGTTGTCCAAGAACTTTTGTAGATGACAGTGGTGGGAGTTGTACTT	-23.22	-	Novel-mir-1042-3p	-	TTTTGTAGATGACAGTGGTGGG
Novel-mir-1041-1	AGGAAAGACAGTTTCACAGTGTTTTCTTCAATAAAAGTCCTAAACCAAAGAAGATTGACCAGGTTTTTGCTGAAGGGACCTGTTAGCTATCTGGCAA	-23.3	-	Novel-mir-1041-3p	-	TTTTGCTGAAGGGACCTGTTAGC
Novel-mir-1041-2	GTGCTGCAGTTTCACAGTGTTTTCTTAAATAAAAGTCCTAAATCGAAGAAGATTGACCAGCTTTTTGCTGAAGGGACCTGTTAGCTATCTGGCAA	-20.7	-	Novel-mir-1041-3p	-	TTTTGCTGAAGGGACCTGTTAGC
Novel-mir-1041-3	GTGCTGCAGTTTCACAGTGTTTTCTTCAATAAAAGTCCTAAACCAAAGAAGATTGACCAGGTTTTTGCTGAAGGGACCTGTTAGCTATCTGGCAA	-22.2	-	Novel-mir-1041-3p	-	TTTTGCTGAAGGGACCTGTTAGC
Novel-mir-1041-4	GTGCTGCAGTTTCACAGTGTTTTCTTCAATAAAAGTCCTAAATCAAAAAAGATTGACCAGGTTTTTGCTGAAGGGACCTGTTAGCTATCTGGCAA	-24.6	-	Novel-mir-1041-3p	-	TTTTGCTGAAGGGACCTGTTAGC
Novel-mir-1041-5	GTGCTGCAGTTTCACAGTGTTTTCTTCAATAAAAGTCCTAAATCAAAGAAGATTGACCAGGTTTTTGCTGAAGGGACCTGTTAGCTATCTGGCAA	-24.6	-	Novel-mir-1041-3p	-	TTTTGCTGAAGGGACCTGTTAGC
Novel-mir-1040	TGGCATGTGGGGCACACGTTTCTTCTAATACATAGAGATGCTGCAAATTTTCAGGTAGGGAGATGGCAGCCGCCACTCCT	-22.2	-	Novel-mir-1040-3p	-	TTTTCAGGTAGGGAGATGGCAGC
Novel-mir-1039	GCCGGCTCTGGTGAGAGCCCGACTGGGTTAGTAGGGCGAAGTTTTAGATAGTGTTTTACCGAAGGAGGCTCTGAGAAAGAGTAATC	-23.2	-	Novel-mir-1039-3p	-	TTTTACCGAAGGAGGCTCTGAGA
Novel-mir-1038	AAAGTCTAGTGTTGGAGGCTGCTCATGTCACAAAAATATCACGACTTTGGTCCTAGGATCATTTTGTTTGACTAGGACGGCATCTGTGGAGGCTT	-24.1	-	Novel-mir-1038-3p	-	TTTGTTTGACTAGGACGGCATCT
Novel-mir-1035	GTCCCTGGGGCTGCTTCAGGGTTGGGAGCTGTGTGTGTCGGTCCAGGCAGACTTTGTTCGGATGGTGGAGCCCACCCACCAGGGTG	-33.9	-	Novel-mir-1035-3p	-	TTTGTTCGGATGGTGGAGCCCACC

Novel-mir-516-2	ACAGTGTTCAGCATCCTCACAGCCTGGTGGATGAAGCTGTTTGTAAGTCTGGTGGAGTGGGTCTGAAGACTCC	-20.2	-	Novel-mir-516-5p	-	TTTGTAAGTCTGGTGGAGTGGGTC
Novel-mir-1034	ACAGTGTTCAGCATCCTCACAGCCTGGTGGATGAAGCTGTTTGTAAGTCTGATGGAGTGGGCCTGAAGGCAGG	-19.3	-	Novel-mir-1034-3p	-	TTTGTAAGTCTGATGGAGTGGGCC
Novel-mir-1031	GCACCTGTCGCAGCGCGGGTCTATGTCAGGGTAGTAGCGCGCCAGTCGAGATTTGGATATGTGAGACCTGTGCACAACTTCGAAC	-24.1	-	Novel-mir-1031-3p	-	TTTGGATATGTGAGACCTGTGCAC
Novel-mir-1030-1	ATGTAATCACTCACAGTCTCTGCATATTCATGGATATTTTGTCTGCAGCCTCTTTGAAGATGTTCCAGTCTGTGGCCTCTAGACAG	-18.22	-	Novel-mir-1030-3p	-	TTTGAAGATGTTCCAGTCTGTGGC
Novel-mir-1030-2	CAGTCACTCACAGTCTCTGCATATTCATGGATGTTGGCTGTGGCCCCTTTGAAGATGTTCCAGTCTGTGGCCTCTAGACAG	-20.9	-	Novel-mir-1030-3p	-	TTTGAAGATGTTCCAGTCTGTGGC
Novel-mir-1030-3	CCAGCTGATGTAATCAATCACAGTCTCTGCATATTCATGGCTGTCATCTATGGCCCCTTTGAAGATGTTCCAGTCTGTGGCCTTTAGGCTG	-24.7	-	Novel-mir-1030-3p	-	TTTGAAGATGTTCCAGTCTGTGGC
Novel-mir-1030-4	GCTGATGTAGTCACTCACAGCCTCTGCATATTCATGAATGTCGTCTGTGGCCCCTTTGAAGATGTTCCAGTCTGTGGCCTCTAGACAG	-19.7	-	Novel-mir-1030-3p	-	TTTGAAGATGTTCCAGTCTGTGGC
Novel-mir-510-2	ACAGTGTTAATAGTAGTATGCCAACCCTCTCAACAGTGTCTCAGAGAAGCAGTGCTTTTCTGATTGGGCTTGTTGCTTGGGTGAATTCTG	-26.29	-	Novel-mir-510-5p	-	TTTCTGATTGGGCTTGTTGCTTGG
Novel-mir-1029	AACGTTGTTCTAGCTGCATAAAACCAACCGTGTGTGCTGTCCTCGATACACGCCTACTTTCGTGACACTAGACAGCCAGCGAACAAATAA	-20	-	Novel-mir-1029-3p	-	TTTCGTGACACTAGACAGCCAGC
Novel-mir-1028	AAGGTACTCTCTGCAGTACTTTCACTGAAGGTACTACTATTACAGTACTTTCACTGAAGATACTGTAGCTTAACTACTT	-28.3	-	Novel-mir-1028-3p	-	TTTCACTGAAGATACTGTAGC
Novel-mir-1027	ACCTGTGTACCGACACCTATTGTCCTGGTGGGCATGTTTGGGCAGAGATTTCAAAACAGGATTGTAGATGGATGACAAATTG	-18.2	-	Novel-mir-1027-3p	-	TTTCAAAACAGGATTGTAGATGGA
Novel-mir-1026	AGCACTGATTTTGTGTCTAAAGGTAGTAAGTGCTATTTGTTGGGGTAGAGTGGCTGAACACTTTAGTTCTTGCTTCTGGGATACAGATCAAAAGA	-28.4	-	Novel-mir-1026-3p	-	TTTAGTTCTTGCTTCTGGGATACA
Novel-mir-1024	TTATCAGGAAATATTCAGCCAAATCCAGGCCCTCTGGAGAGCAACATCAGCACGCCTGATGAATTTAGAGCTAGATCAGGACTTGGGATCCTTCATC	-20.3	-	Novel-mir-1024-3p	-	TTTAGAGCTAGATCAGGACTTGGG
Novel-mir-1022-1	AGGGCAGGAGGGGCAAAGAGAAGCAGAGGGAGAAGAGGAGGGGATACTGCTTGGGTTTGTTTTGATTCTCACTTTGCCCTTAACGGTCAA	-33.4	-	Novel-mir-1022-3p	-	TTGTTTTGATTCTCACTTTGCCCT
Novel-mir-1022-2	AGGGCAGGAGGGGTGAAGAGGAAGGGATACTGCTTGAGTTTGTTTTGATTCTCACTTTGCCCTTCACAGTCAC	-23.3	-	Novel-mir-1022-3p	-	TTGTTTTGATTCTCACTTTGCCCT
Novel-mir-1022-3	CAGGGCAGGAGGGGCAAAGAGGAGCAGAGGGAGAAGAGGAGGGGATACTGTTTGAGTTTGTTTTGATTCTCACTTTGCCCTTAATGGTCGA	-32.4	-	Novel-mir-1022-3p	-	TTGTTTTGATTCTCACTTTGCCCT
Novel-mir-1022-4	GAGACGGGGCAGAAGGGGCAAAGAGAGAGGATGGGATACTGCTCATGTTTGTTTTGATTCTCACTTTGCCCTTAACGGTCAA	-28.7	-	Novel-mir-1022-3p	-	TTGTTTTGATTCTCACTTTGCCCT
Novel-mir-1022-5	GGACAGGAGGGGCAAAGAGGAGTGGAGGGAGGAGTGGAGGGAGAAGTGGAGGGGACGCTGCTTGAGTTTGTTTTGATTCTCACTTTGCCCTTAACGGTCAG	-33	-	Novel-mir-1022-3p	-	TTGTTTTGATTCTCACTTTGCCCT
Novel-mir-1022-6	TTTCATAAACAGGGCAGGAGGGAGAAGAGGAGGGAATACTGCTCGAGTTTGTTTTGATTCTCACTTTGCCCTTAACAGTCAA	-22.3	-	Novel-mir-1022-3p	-	TTGTTTTGATTCTCACTTTGCCCT
Novel-mir-1020	CTACCAAAGCATCCCTTGTCATTCAGATGGTGACAGACAGTGTGAGCTCACACTGTTGAATTTGAATTTGTTTGGAAGTTGATCAAGGTTCTTTATCCA	-25.7	-	Novel-mir-1020-3p	-	TTGTTTGGAAGTTGATCAAGGT
Novel-mir-1019-1	ACGGTACATGGGTTGCGAATCAAAAGCCTGGAGACTGTTGAGATAGTTGTCATGTTCACCATATTGTTTGATATCTGAGCTCGTGCCCAGAATAACA	-20.5	-	Novel-mir-1019-3p	-	TTGTTTGATATCTGAGCTCGTGCC
Novel-mir-1019-2	ACGGTACGTGGGTTGCGAATCAAAAGCCTGGAGACTGTTGAGATAGTTGTCATGTTCACCATATTGTTTGATATCTGAGCTCGTGCCCAGAATAATA	-20.7	-	Novel-mir-1019-3p	-	TTGTTTGATATCTGAGCTCGTGCC
Novel-mir-1018	GACACTTGGCATTCAGACCAGAGTTCAATCTTTGTTTCATCAGACCAGAGTTTTGTTTCTCGTGGTCTGAGAGTCCTTCAGGTGCC	-28.8	-	Novel-mir-1018-3p	-	TTGTTTCTCGTGGTCTGAGAGTCC
Novel-mir-1013	CTTTTGGTGTCCTCTGCATCTAATGGCAGCCTCATGTCTGACACATGTAGGATGGCTCATCTCTACATTGTCAAGTCTGGAGCTGTAACCTATTAAC	-29.6	-	Novel-mir-1013-3p	-	TTGTCAAGTCTGGAGCTGTA
Novel-mir-1005-1	AGTGCTACAAGTGCTGTGTCTTGGCAGAACCGACAAGCGCTCAGTTGGATTCTAGCAGAGAAAGGAGGTGTCTGTGCC	-22	-	Novel-mir-1005-3p	-	TTGGATTCTAGCAGAGAAAGGAGG
Novel-mir-1005-2	GAGTGCTACAAGTGCTGTGTCTTGGCAGAACCGACAAGTGCTCGATTGGATTCTAGCAGAGAAAGGAGGTGTGTGTGCT	-21.4	-	Novel-mir-1005-3p	-	TTGGATTCTAGCAGAGAAAGGAGG
Novel-mir-1004	GCCAAGGCCTTCTATTAAAGTGTACCTTGCTGCCACCATTGCCTTTACAGTTTTGCGGCTTGGAATGGAGTATGATGGGTCCAGGATTTTGAT	-21.6	-	Novel-mir-1004-3p	-	TTGGAATGGAGTATGATGGGTCCA
Novel-mir-1002-1	ACATTACACGTCCGGATCTCTCCCACCATCCACCTGCGCGCAGGCAGCTTGGAAAGATGAACAGAGACCGGACCCAGAAACC	-26.3	-	Novel-mir-1002-3p	-	TTGGAAAGATGAACAGAGACCGGA

Novel-mir-1002-2	ACATTACACGTTCGGATCTCTCCCACCATCCACCTGCGCGCAGGCAGCTTGGAAAGATGAACAGAGACCGGACCCAGAAACC	-23.4	-	Novel-mir-1002-3p	-	TTGGAAAGATGAACAGAGACCGGA
Novel-mir-999	GTGGACGACCAAATACAGACTACACCATTCAGTGTGAATCAGTGCACCATTGCTTGTCACTGTAGCCTATAGCCGGTGTCCTT	-20	-	Novel-mir-999-3p	-	TTGCTTGTCACTGTAGCCTATAGC
Novel-mir-996-1	ATATTCTGTCCACTGAGCTTCTGAGTCAGGTCCAGGACAACTCTCATCCCTTGATTTATCTCTGGGGCTCCTCCGTCAGGTTTC	-23.4	-	Novel-mir-996-3p	-	TTGATTTATCTCTGGGGCTCCTCC
Novel-mir-996-2	TGCATGTGATGTTGTGTCCATTAAGTTGCTGAGTCAGGTCTAGGACAACTCTCATCCCTTGATTTATCTCTGGGGCTCCTCCATCAGGTTTC	-20.4	-	Novel-mir-996-3p	-	TTGATTTATCTCTGGGGCTCCTCC
Novel-mir-992-1	GTGAAAGTACTGCAATAGTAGTACCGTCAGTGAAAGTACTGCAGACAGTACCTTGAGTGAAAGTACTGCAGTGGCAGTATCTTCAG	-37.1	-	Novel-mir-992-3p	-	TTGAGTGAAAGTACTGCAGTGGCA
Novel-mir-992-2	GTGAAAGTACTGCAGTGGCAGTATCTTCAGTGAAAGTACTGTAATAATAGTACCTTGAGTGAAAGTACTGCAGTGGCAGTATCTTCAG	-38.7	-	Novel-mir-992-3p	-	TTGAGTGAAAGTACTGCAGTGGCA
Novel-mir-989	AGCAGGTGGGGAGTTCACACAGCTTCAGTGACCTGTTGAAGATGAGTGTGAAGGTGGGGCCAGCTGAT	-19.7	-	Novel-mir-989-3p	-	TTGAAGATGAGTGTGAAGGTGGG
Novel-mir-988	CGGCAACAGTAAAAGCACGGTCACCCCAGCATCTGGCCCTAAGAAAGTGGTTTGAAGATCTGAGGGACTTTGTAGATTTGTGCCA	-27.5	-	Novel-mir-988-3p	-	TTGAAGATCTGAGGGACTTTGTAG
Novel-mir-987	ACCGCTGACTGAGGGAGTTCAACTTGCAGGTGATAGCAAGCTACCTTCTTGAGTTCTGACACTCCTTCCGATAGTGTGA	-23.9	-	Novel-mir-987-3p	-	TTCTTGAGTTCTGACACTCCTTCC
Novel-mir-985-1	AGGTCTGGGCTCTGACTCTCCAAAAGGCAGATTTAGTTTTTCTGAAGTCATTCTGTTGTGGACTTGCTCTGGTGGTTTGGGTC	-20.5	-	Novel-mir-985-3p	-	TTCTGTTGTGGACTTGCTCTGGT
Novel-mir-985-2	GGGCTCTGACTTGGCCACTCCAAAAGGCGGATTTTATTTTTCTGAAGCCATTCTGTTGTGGACTTGCTCTGGTATTTTGGGTC	-19.4	-	Novel-mir-985-3p	-	TTCTGTTGTGGACTTGCTCTGGT
Novel-mir-983	TTGCACCGGAGTCAGCAGGGTCTATGGTGGCCAGTTCTTTCTGTTAGATCCGCTCGGACACAGAGGTGAGC	-20.4	-	Novel-mir-983-3p	-	TTCTGTTAGATCCGCTCGGACAC
Novel-mir-978	GGGTGCCGTGAATACAAAAAACATGCTGAACTAGGAGCTGGGCGGTTTCTAGAGCAGTGGGGAGTTGTGGAAGGGCGAGG	-27.5	-	Novel-mir-978-3p	-	TTCTAGAGCAGTGGGGAGTTGTGG
Novel-mir-977	GATCATATCCCTGAGAGGTTGAGTGAGATCCACATACCCAGGAATGTGGTTTCTACTGTAGCCTGTGAGTCCCAGGAATGCCA	-24.7	-	Novel-mir-977-3p	-	TTCTACTGTAGCCTGTGAGTCCC
Novel-mir-976	TTTCAGACTATGCTGTCCCATTCTGATTGAGTTAGCGCTAGTTCGTTTAAATGTGGGCTTTAGCATATCTGGGC	-22.9	-	Novel-mir-976-3p	-	TTCGTTTAAATGTGGGCTTTAGC
Novel-mir-973	AGACATACCTGCATTCATGGTATGTGTTGTACGTCTTCCTGATTTTCGTGCTGACAGTTCCTGTGCTGTGGATTTCATTTTGTATGTT	-19.92	-	Novel-mir-973-3p	-	TTCCTGTGCTGTGGATTTCAT
Novel-mir-968	CTCCTTTTGCCATATAGGTCTTCCCTTGGGAATGAGCCAGGAGGGGGCAGTGCTATAGTGGTTTTCATGTGGAGTAGAAGATTGGGGGTAAGTGGTT	-24.7	-	Novel-mir-968-3p	-	TTCATGTGGAGTAGAAGATTGGGG
Novel-mir-967	GGGTGGGGGGGTGTTGAGTGTCAAACCTGCAGTAGAATATATTCAGGTCTTGTGCCAGTTGACGGTCCTCCTCAG	-28.3	-	Novel-mir-967-3p	-	TTCAGGTCTTGTGCCAGTTGACGG
Novel-mir-966	TTACAGAGCTCTGGGTTCCATCTCTATCTGAAGCCCCGACCTCAACAGCAAAACCGTTCAACCCGTCTTCAGGATGAGAAGGAACCCTGTCCTCCTCCA	-29.7	-	Novel-mir-966-3p	-	TTCAGGATGAGAAGGAACCCTG
Novel-mir-964	TGAGGAGATTAGGGGTTGCTCCTTCCTGAAGAAGCTGTGAACCATACTTCTGTAGTGGGGATAGGGCTTCAGATAGACGGAACCTAGAGCTCTGTATTGC	-28.8	-	Novel-mir-964-3p	-	TTCAGATAGACGGAACCTAGAGC
Novel-mir-963	AAACTGAGGTAGGCTCCTCAGATTGGTCTCTACGCAGGATTTCAACAACCTGTTCAGAGAGACTCATCACGGCGCTGTTCAATCG	-25.4	-	Novel-mir-963-3p	-	TTCAGAGAGACTCATCACGGCGC
Novel-mir-962-1	CAGTTGCTCAACAGTCCAGGGCCTGGGTTGTTGTATTTTGCACTTTATAATGTGCCACACATTTTCAATGGGAGACGGGTCTGGACTGCAGGCACTC	-30.99	-	Novel-mir-962-3p	-	TTCAATGGGAGACGGGTCTGGACT
Novel-mir-962-2	TACGACTTCAGTTCAGTCTGGGGTCTGCGTTGTCGTATTGTGCGCTTCATAATGCGCCACACATTTTCAATGGGAGACGGGTCTGGACTGCAGGCAGGT	-34.8	-	Novel-mir-962-3p	-	TTCAATGGGAGACGGGTCTGGACT
Novel-mir-961	TGTACCAGATGATAGGCATTCTGTAAGTCTAGTTTAGTGAAAATCGTGGCGTTATGGAGCATTTCAAAAGCAGACGACATGAGTGGTAATGGGTAT	-19.3	-	Novel-mir-961-3p	-	TTCAAAAGCAGACGACATGAGTGG
Novel-mir-959	TTGAACGTGTGCTCAGTGTTTGGAGAATGTCACGATGTCAAGTATAATGCTGTCAAAAGTGTCATTATGATATGCTGTACAAACGAGGATAGGTTCCT	-24.9	-	Novel-mir-959-3p	-	TTATGATATGCTGTACAAACGAGG
Novel-mir-958	GTTTTCAGTTCTTCACACTTACCTCTGATGTTGACAATCACACATTATGAGCGAGACTGTGAATAAATGAAAAA	-19.1	-	Novel-mir-958-3p	-	TTATGAGCGAGACTGTGAAT
Novel-mir-955	AGGTGGGGCATTTTTTCCAGTTGGCTCCTGGGCCTGATATTGCCCAAGTGTTGGAGGAGGGTAGGCATTATAAGCATACTGGGGGGTGCCTCCAGCT	-37.9	-	Novel-mir-955-3p	-	TTATAAGCATACTGGGGGGT
Novel-mir-954	TACGGTTGTCAGGTCACCTTGCCCTTGTTGTCCCTTCTTCTCGTTATAAGAAATGGGGAGGTCGGGCCTTTGCCGT	-22.5	-	Novel-mir-954-3p	-	TTATAAGAAATGGGGAGGTCGGG

Novel-mir-953	GTGATGACATTCTCAATGGTTCCCCGGTAGAGGGAGTGCATGATGGGGGGTGGGACACTAGCTCTTCTTAGTTTGCGGAGGAAGTAGGGGCATGTTTGAGC	-26.7	-	Novel-mir-953-3p	-	TTAGTTTGCGGAGGAAGTAGGGGC
Novel-mir-952-1	TTTGCCAAGTTAAAGCAGTACTTATTGTGCTTACGAGCATGTTTTTTTAGAGTGTAGGGAGTCGGTTTAGTGTAGAATTGCTCTGTTAAGGTCAAAA	-20.2	-	Novel-mir-952-3p	-	TTAGTGTAGAATTGCTCTGTT
Novel-mir-952-2	TTTTGCCATGTTAAAGCAGTACTTATTGTGCTTACGAGCATGTTTTTTTAGAGTATAGGGAGTTGGTTTAGTGTAGAATTGCTCTGTTAAGGTCAAAA	-19.2	-	Novel-mir-952-3p	-	TTAGTGTAGAATTGCTCTGTT
Novel-mir-949	ATACTGCAGGAAGACAGACACAAGTCAAAGTCCAGCTGGGTATGACTTGGTTTAGATTTAGAGGTTCTGTCAAACATATAGCC	-18.4	-	Novel-mir-949-3p	-	TTAGATTTAGAGGTTCTGTCAA
Novel-mir-947	CAGAGAGACAGAGCTCCTGCAGGAAATGTATTCTCTGAACCACGTGTTATATTTTCTGGTTACATTTATCTTCTGGGCTGAGTCCTTCTT	-18.74	-	Novel-mir-947-3p	-	TTACATTTATCTTCTGGGCTG
Novel-mir-946	AAAAGCAGACCCAGGTGGCTGGCTTCGGTGCAAGCAGTGAGGTTTATTTACAGTGAAGGTAGTGACAGGGGGTCCAGAGGA	-25.9	-	Novel-mir-946-3p	-	TTACAGTGAAGGTAGTGACAGGGG
Novel-mir-945-1	ACGTTGGCGCTAATGACTCCTGACTTCGCCAGTTGGAGGTCACTAAAATTAATGTTGAGTCAGTGTGTAACTTCGCTAAAAC	-20.3	-	Novel-mir-945-3p	-	TTAATGTTGAGTCAGTGTGTAACT
Novel-mir-945-2	TGGTGTAAGATTGTTGTTCACGTCCTCATTAATGACTCCCAACTTTGCCAGTCAGAGGTCACCAAAATTAATGTTGAGTCAGTGTGTAACTTCACTAAAAC	-18.17	-	Novel-mir-945-3p	-	TTAATGTTGAGTCAGTGTGTAACT
Novel-mir-943	TGGAGAGGGGGGCCGGTAGTCTTGAGTGGTTGTGTGCGCGAGCGCGGGTTAAGTTGTAGCTCTGCCATGGCCTCGTGTAAAC	-27.7	-	Novel-mir-943-3p	-	TTAAGTTGTAGCTCTGCCATGGCC
Novel-mir-936	TGACCTGAGACTTGAGAGCTGGTATCCCATAGTAAATAGCCTCCTGTTTGACTTATCTGTTTGAGCATCTGTTTTCTGTTTTAGTATG	-18.3	-	Novel-mir-936-3p	-	TGTTTGAGCATCTGTTTTCTG
Novel-mir-935	CCTGGTTGGATGACAACATCCTGGACGCTGCGGTGGAGCTGGAGGGACGCTTCTTGTTTCAAGCAGACAGGACTGCAGCATCTCCTAA	-31.9	-	Novel-mir-935-3p	-	TGTTTCAAGCAGACAGGACTGCAG
Novel-mir-933	TTAAGCATCTGTTTTCTTTGTTGTATGCTGAGTATAAGGGCAGTCACATTCTGTTCATTGAGCTTCCCTGCAACAGACTGGTTTA	-18.5	-	Novel-mir-933-3p	-	TGTTCATTGAGCTTCCCTGCAACA
Novel-mir-931-1	TCATCTTAAAACTCAGTGCTGCTTTTGACACCATTGATCACAGCATCTTACTGCAGAGACTTGAACATGTTATTGGGATTAAAGGAACTGTATTAAGTTGG	-21.2	-	Novel-mir-931-3p	-	TGTTATTGGGATTAAAGGAACTGT
Novel-mir-931-2	TCCTCTTAGTTCTCAGTGCTGCTTTTGACACCATTGATCACAGCATCTTACTAAAAAGACTTGAACATGTTATTGGGATTAAAGGAACTGTACTAAGTTGG	-24.9	-	Novel-mir-931-3p	-	TGTTATTGGGATTAAAGGAACTGT
Novel-mir-931-3	TGCTCTTAGATCTCAGTTCTGCATTTTGACACCACTGATCACACATCTTATTACAGAGACTTGAACATGTTATTGGGATTAAAGGAACTGTACTAAGTTAG	-20	-	Novel-mir-931-3p	-	TGTTATTGGGATTAAAGGAACTGT
Novel-mir-930	GATCTCAGTGCTGCTTTTGACACCATTGATCACACATTACAGAGACTTGAATGTGTTATTGGGATTAAAAGAACTGCACTAAGTTG	-24.7	-	Novel-mir-930-3p	-	TGTTATTGGGATTAAAAGAACTG
Novel-mir-927	GTGCTGTTGCCCAGGTTTCCAAGTTTGGTCGTTAACTTGGAGGGGATGACCATGTTAAACGCTGAGCTGAAGTCCACGAACAGCA	-24.8	-	Novel-mir-927-3p	-	TGTTAAACGCTGAGCTGAAGTCC
Novel-mir-926	TTTCACCTTCCCCGAAGCAGCAGTGCAGTCACCTCGATGTACAGAGTGTGTTTGTAGCTGAATCGTGGAATCTTGAAC	-26.4	-	Novel-mir-926-3p	-	TGTGTTTGTAGCTGAATCGTGG
Novel-mir-923	GAGTGTTACTTCTTGGGGCCTTCAGATAAAAGAACAGGTTGAATCTAAAATCAGCTATAAATGTGTCTAGATTGGTCTAAAGAAAAAATCTAA	-20.66	-	Novel-mir-923-3p	-	TGTGTCTAGATTGGTCTAAAGA
Novel-mir-921-1	AGATGCAGCAGTCCTCTGGCGATCACCCGGCCCCACTTGTAACTGCACGGTTCCACCTGTGGTGTGTAGTACAGCGTGGGGCACGTCCACCATCG	-37.4	-	Novel-mir-921-3p	-	TGTGTAGTACAGCGTGGGGCACG
Novel-mir-921-2	TGGCGATCACCCGGCCCCACTTGTAACTGCATGGTTCCACCGGTGGTGTGTAGTACAGCGTGGGGCACGTCCACCGTCG	-34.5	-	Novel-mir-921-3p	-	TGTGTAGTACAGCGTGGGGCACG
Novel-mir-919-1	CAATGTTCATCCGGGAGATATCTAGGATGTATGTTTACAATGAGATATGCTGGTCTGTTGAAAGTGTGTAGAGACAGTTCATCGGATGCCCATAA	-23.4	-	Novel-mir-919-3p	-	TGTGTAGAGACAGTTCATCGG
Novel-mir-919-2	CAGTGTTCACCCGGGAGATGTCTAGGATGTGTGTTTACGATAAGATACGTGAGTTTGTTGAAAGTGTGTAGAGACAGTTCATCGGATGCCCACAC	-24	-	Novel-mir-919-3p	-	TGTGTAGAGACAGTTCATCGG
Novel-mir-915	TCTGCTTTTGGCCAGGACATTGTCAAATGCATTTTTTCTGCAGAGAAACTGTGACAGAACAATGGAGATGGTGCACGACGCAG	-22.2	-	Novel-mir-915-3p	-	TGTGACAGAACAATGGAGATGGTG
Novel-mir-913	CGACAGAACTCATTCCAGAAGTGGGCCACAAACTGAGGGCCTCTGTCTGACACCACATCTCTGGGGAGGCCATGCA	-19.3	-	Novel-mir-913-3p	-	TGTCTGACACCACATCTCTGGGG
Novel-mir-912-1	TATTGCCTTTCCAATTGCAGCCACATGTCTGACACATGTAGGATGTCCCATTTCTACATTGTCAAGTCTGGAGCTGTAATACATCAGAACCTT	-27.5	-	Novel-mir-912-3p	-	TGTCAAGTCTGGAGCTGTAATACA
Novel-mir-912-2	TATTGCCTTTTCAATTGCAGCCACATGTCTGACACATGTAGGATGTCCCATTTCTACATTGTCAAGTCTGGAGCTGTAATACATCAAAACCTT	-27.5	-	Novel-mir-912-3p	-	TGTCAAGTCTGGAGCTGTAATACA
Novel-mir-911	TTGTTTCAGCGGATCTTCTGTATTGACTAAGTCTCTCTGCAAACGCTGTCAACAGCTGCTTGTCTACTGTATTCAACTCGTGAGAAGCTGAACCTTAATAT	-23.1	-	Novel-mir-911-3p	-	TGTATTCAACTCGTGAGAAGCTGA

Novel-mir-909-1	TGGCGATCACCCGGCCCCACTTGTAACTGCACGGTTCCACCTGTGGTGTGTAGTACAGCGTGGGGCACGTCCACCATCGCCC	-40.5	-	Novel-mir-909-3p	-	TGTAGTACAGCGTGGGGCACGTCC
Novel-mir-909-2	TGGCGATCACCCGGCCCCACTTGTAACTGCATGGTTCCACCGGTGGTGTGTAGTACAGCGTGGGGCACGTCCACCGTCGCCC	-39	-	Novel-mir-909-3p	-	TGTAGTACAGCGTGGGGCACGTCC
Novel-mir-907	TTCGGTGCAAGCAGTGCTAAATTTATTTACAGTGCAATGTGAACAAGTTCATACGATAACCAGTAAGTGTAGAATGATGAGAGTAACCGGGTGTCGATCAG	-19.9	-	Novel-mir-907-3p	-	TGTAGAATGATGAGAGTAACCGGG
Novel-mir-905-1	GGACGAGCAGCGTTGGGGATTTTGCGTGAATATTATGCTGGGAAAGGGAAACCAAGAATAATTAGTTTGTACACGGAGTTAACTTCACTGCAGAAGGAGTC	-21.41	-	Novel-mir-905-3p	-	TGTACACGGAGTTAACTTCACTGC
Novel-mir-905-2	GGACGAGCAGTGTTGGGGATTTTGCGTGAATATTATGCTGGGAAAGGAAAACCAAGAATAATTAGTTTGTACACGGAGTTAACTTCACTGCAGAAGGAGTC	-23.2	-	Novel-mir-905-3p	-	TGTACACGGAGTTAACTTCACTGC
Novel-mir-904	GGCTTCTCTGCCATGACTATTTAGATTTTCATAAATATGTTTTATTCGTCCGATCGAAGTGGCCATCTGTAAGTAGATTTGTGTAGTAGGCATAGAACCAG	-24.5	-	Novel-mir-904-3p	-	TGTAAGTAGATTTGTGTAGTAGGC
Novel-mir-903	AGGCAAAATTAATATTACAAGCTCAAGTATTTTGCACCTCCACACTGCACGCAGGCAGATGTAAGATGAAAAGACCCTGAGCCCACCAGGCC	-18.2	-	Novel-mir-903-3p	-	TGTAAGATGAAAAGACCCTGAGC
Novel-mir-897	AGACCTCAGTGCTGCTTTTGGCACCATTGATCACAACATCTTATTACAGAGACTTGAACATGGTATTGGGATTAAAGGAACTGCACTAAGTTGG	-20.6	-	Novel-mir-897-3p	-	TGGTATTGGGATTAAAGGAACTGC
Novel-mir-896	AGGACTGGTATATGACCATCTTTATTTTAGTCTGTATAATCCCATACTGGTCCCAAGGTGGTAAATGCTCTGCAGAGAGGGAGGTGGAGTCC	-24.2	-	Novel-mir-896-3p	-	TGGTAAATGCTCTGCAGAGAGGGA
Novel-mir-891	TAGGGGATGCCGTCAAGCTGAAGAAGGAGTCCTTTCGAACCTGGTTGGCTCGTGGGACACCTGAGGCAGCTGATGGGTATCGGCAT	-37	-	Novel-mir-891-3p	-	TGGGACACCTGAGGCAGCTGATGG
Novel-mir-890	AAAACCAGGATCCAAATCCCCATGTTCAAATGACGAGTGAACTACTGGGAATTAAACTGGTAGTTTGGCTCCATTTTGC	-20.9	-	Novel-mir-890-3p	-	TGGGAATTAAACTGGTAGTTTGGC
Novel-mir-887	GGTTCGATACAATTGCTTTATGAGCTACCACACATGTGTCGGAGCCTCAAGTCTCTGGATGTTGTGGGGTTGTCTTGGCTGATTCGGAT	-23.2	-	Novel-mir-887-3p	-	TGGATGTTGTGGGGTTGTCTTGGC
Novel-mir-886	GATTAGGAATATATGCTGGAGCAGTGCTTTCAATCTACAAATCGCAGAATTTAAACAAGTGGATGAAATGCTGGCTAATGGTATTACTGAGC	-18.15	-	Novel-mir-886-3p	-	TGGATGAAATGCTGGCTAATGGT
Novel-mir-885	GAGGGTTCATGGGAGTTTGCCCAACCAGTCCACATGTGTTTCGTGGATCTGGAGAAGGCATTCGTTCGTGTCCCT	-30.3	-	Novel-mir-885-3p	-	TGGATCTGGAGAAGGCATTCGT
Novel-mir-883-1	AAGGACCGCTCCTACCCCAGTGTCCGACGCGTCCACCTCTACCACGAACTGCCATGAGGGATCAAGTTGGAGGAGTATGGGGGCTGTGGTGAACCGGTCTT	-34.89	-	Novel-mir-883-3p	-	TGGAGGAGTATGGGGGCTGTGGTG
Novel-mir-883-2	GAGGGCCGCTCCCACCCCAGTGTCGGACGTGTCCACCTCAACCACAAACTGCCGTGTGGGGTCTGGTTGGAGGAGTATGGGGGCTGTGGTGAACCGGTCTT	-38.1	-	Novel-mir-883-3p	-	TGGAGGAGTATGGGGGCTGTGGTG
Novel-mir-881	TGTCTGTTGGCACAAATTTTGCCTCTTTCAATTCTCTCTACTACCATTGCAACTCCCTGCTTGCCAATGGAAGAAGACTGTATTTGAACCATCTCTGGCT	-21.4	-	Novel-mir-881-3p	-	TGGAAGAAGACTGTATTTGAACC
Novel-mir-321-2	TAACATCACACGTCCAGATCTCTCTCACCACCAAACCTGCGCGCAGGCAGCTTGGAAAGATGAAAGACCCTGGACCAAGAAACCTA	-21	-	Novel-mir-321-5p	-	TGGAAAGATGAAAGACCCTGGACC
Novel-mir-321-3	TAACATCACACGTCCAGATCTCTCTCACCACCAACACTGCGCGCAGGCAGCTTGGAAAGATGAAAGACCCTGGACCAAGAAACTTA	-21	-	Novel-mir-321-5p	-	TGGAAAGATGAAAGACCCTGGACC
Novel-mir-321-4	TAACATCACACGTCCAGATCTCTCTCACTACCAAAACCTGCGCGCAGGCAGCTTGGAAAGATGAAAGACCCTGGACCAAGAAACCTA	-21.7	-	Novel-mir-321-5p	-	TGGAAAGATGAAAGACCCTGGACC
Novel-mir-878	TTTTCAGCTTACTATGACCTGGATGACTGAGAATTTGCAACCTGAGAGTAGAGGTTGGAGTGCTTAATCAGAGGAGGCAGTAGAGAGGCTGGGA	-30.8	-	Novel-mir-878-3p	-	TGCTTAATCAGAGGAGGCAGTAGA
Novel-mir-875	CGGCAGAGCGTGCAAAGTCAGGGTTAGTCCTTGAAGTCTTCCGGTTCTTCTAGCATGCTAACTCTGAGAGGCGAGGCAGTTCAAGTA	-24.6	-	Novel-mir-875-3p	-	TGCTAACTCTGAGAGGCGAGGC
Novel-mir-872	TCAGTGTTATTAAAAATTAATCACATTTTGTTGGCTAGGGATGCTGGAGATCTAGTTCTGTTGGTGCCTTTAGACCTCTCTGCTGCCTTTGATACAGT	-19.1	-	Novel-mir-872-3p	-	TGCCTTTAGACCTCTCTGCTGCCT
Novel-mir-867	ATCCTGATTCTACGGTCATTACACAGTGAGGTCTTTCAATTATTATATGCTTTTTCCACTAGAGATGCATTAATACTGTGAAAACTGCCTAAGGGAGGA	-18.4	-	Novel-mir-867-3p	-	TGCATTAATACTGTGAAAACTGCC
Novel-mir-866	TGACTGGAGTAGCTTGGTTCAGATTACCGGCAGTAAGTCAGACCTGTTTCTGGTGCATGTTGGACTCTGCCAAGGCTGCCCTTTGT	-29.1	-	Novel-mir-866-3p	-	TGCATGTTGGACTCTGCCAAGGC
Novel-mir-857	GATGACCAGCATTAGTGAAGGGGTTATTGTCCATGGAGTGATGAGGTCGGGCTGCAAGGGAAGGCGACACTCCTGAGCTGGTCAAG	-29.3	-	Novel-mir-857-3p	-	TGCAAGGGAAGGCGACACTCCTGA
Novel-mir-856	CTCCCTTCCTCCTCGGAACAGTTCCTTTAGCACAAGACTGTTACATCCCCAGTGCAAGAAGGAGCGTTACTGTAGGTCGTTCCTCC	-20.57	-	Novel-mir-856-3p	-	TGCAAGAAGGAGCGTTACTGTAGG
Novel-mir-854	CGAGGCTCGAGGCTCTGAACAGTTCTTTCAGCACAAGACTGTTACATCCCCAGTGCAAGAAGGAGCGTTACCGTAGGTCGTTCCTCC	-23.47	-	Novel-mir-854-3p	-	TGCAAGAAGGAGCGTTACCGTAGG

Novel-mir-852	TTCCTAGCTCTCTCATTGCACTACAGTCACAGTCACAGTTGCACCACAGGTGCACCACAGGTGCAACTGTGACTGACGACATCTGGCTTGGA	-39.6	-	Novel-mir-852-3p	-	TGCAACTGTGACTGACGACAT
Novel-mir-849-1	TCTTTGGATCAAGTACGTTGCTGTTCCAAACCCATCCTCGCTTGCATCTGCAAAATGATGTAATTGGGCAGGCACTGGCTGACCAAAAT	-23.2	-	Novel-mir-849-3p	-	TGATGTAATTGGGCAGGCACTGGC
Novel-mir-839-2	AGGTCCTCTCTGTGCAGTGGGGAGTGAAATGTGTCATATGTTTTGATGCTGGAAAGCACCTTCTGAGTTATCATTTCTGTAGACAGTCAAGATT	-18.5	-	Novel-mir-839-3p	-	TGAGTTATCATTTCTGTAGAC
Novel-mir-835	GGGGAGTTGTGGAAGGGCGAAGACATGGGTGGCTTTGGAGAATCGGTCAACGATGGTGAGTATGACAGTGTTGCCCTGGGAGGGAGGAAG	-19.9	-	Novel-mir-835-3p	-	TGAGTATGACAGTGTTGCCCTGGG
Novel-mir-833-1	TGCGGACGCTGGGGTCACTCCTGGTCGTAGGTGAAGCGATGATGGCTGAGGAGACAGGAGCATGCATAGATAGTTAACG	-18.3	-	Novel-mir-833-3p	-	TGAGGAGACAGGAGCATGCATAG
Novel-mir-833-2	TGGTTATGGATCGGGCTGTGGACGCTGGGGTCACTCCTGAGCTAGTCGTGGTTGAAGCGATGATGGCTGAGGAGACAGGAGCATGCATAGATAGCTAATG	-33.2	-	Novel-mir-833-3p	-	TGAGGAGACAGGAGCATGCATAG
Novel-mir-830	ACAGAGTCACGTGTTTAAAAACAGGCCCTGGTGACACGGGAGCTGTGGCGCAGCTGTGTGAGCAGGGCTGTGTTTTTGAGCAGGTGTGCTG	-48.5	-	Novel-mir-830-3p	-	TGAGCAGGGCTGTGTTTTTGAGC
Novel-mir-823	AATATTTTCAAGTTGCATTTTTAGTTCTCTTAATTCATTTTGTGTTTCCTCGCTAGGGGTTTGAGAATGTAAATCTGTGAGTTGTTTTATCT	-18.6	-	Novel-mir-823-3p	-	TGAGAATGTAAATCTGTGAGT
Novel-mir-822	CCTGACGTTCCACCAGGTGGCGCATTAGGCGCGGTGATACATTGACTTGATTTTGCGGAGCCAGGGCTACTCTCAG	-19	-	Novel-mir-822-3p	-	TGACTTGATTTTGCGGAGCCAGGG
Novel-mir-821	TGGGTGTTTTGGCTTGTTGCATCTACAATTTCTGCCAGTCACTTGTCACTTATAGGGATGCTACTCATGACTGTGTGCACATGTGCTTCCATGCTCTC	-21.7	-	Novel-mir-821-3p	-	TGACTGTGTGCACATGTGCTT
Novel-mir-820	AGCTCAGAGAGGAATCTTCTGCGAACCACTGCTTCGAGGCTTGTATCGTTGCCAAAAAGACATGTGACTGATGATGTCCGACGCAGAAACAGCTGGCC	-24.8	-	Novel-mir-820-3p	-	TGACTGATGATGTCCGACGCAGAA
Novel-mir-819-1	AGTCGAGCCAGGAGAGGGATCTCCCGAACCACTGCCTCGGGGCTTGTCTCGTTGCCTAAAAGACTTGTGACTGATGACGTCCGACGCAGAAATGGCTGGCC	-35.2	-	Novel-mir-819-3p	-	TGACTGATGACGTCCGACGCAGAA
Novel-mir-819-2	CGCGAACCACTGCCTCGAGGCCTGTATTGTTGCCGAAAAGACACGTGACTGATGACGTCCGACGCAGAATCGGCTGGCC	-18.7	-	Novel-mir-819-3p	-	TGACTGATGACGTCCGACGCAGAA
Novel-mir-819-3	CGCGAACCACTGCTTCGAGGCTTGTATCGTTGCCAAAAAGGCATGTGACTGATGACGTCCGACGCAGAAACGGCTGGCC	-22.7	-	Novel-mir-819-3p	-	TGACTGATGACGTCCGACGCAGAA
Novel-mir-819-4	CTCCAGCGAACCACTGCCTCGAGGCTTGTATCATTGCCGAAAAGACACGTGACTGATGACGTCCGACGCAGAAACAGCTGGCC	-24.4	-	Novel-mir-819-3p	-	TGACTGATGACGTCCGACGCAGAA
Novel-mir-819-5	TCGAGCTGGGAGAGAGCTCTTCCGCGAACCACTGCCTCGAGGTTTGTATCGTTGCCCAAAAGACACGTGACTGATGACGTCCGACGCAGAAACGGCCTGAG	-26	-	Novel-mir-819-3p	-	TGACTGATGACGTCCGACGCAGAA
Novel-mir-819-6	TTCCACGAACCACTGCCTCGAGGCTTGTATCGTTGCCAAAAAGACGTGACTGATGACGTCCGACGCAGAAATGGCTGGCC	-20.2	-	Novel-mir-819-3p	-	TGACTGATGACGTCCGACGCAGAA
Novel-mir-817	AACTTGACTAGATTTCTAGTCTAGATTTTCAGTTGATTTTTTTCAGTTTACTATGACCTGGATGACTGAGAATCTGCACGGACATCTAATATTCTG	-25.1	-	Novel-mir-817-3p	-	TGACTGAGAATCTGCACGGACATC
Novel-mir-816	AAGTCCAAAGCATATGCCAGTGTTGTGAGTTTGTTTTGGATGCACAGTATACTGACTCAAGAAACTGGCATATGTTTTGGACAT	-44.3	-	Novel-mir-816-3p	-	TGACTCAAGAAACTGGCATATG
Novel-mir-815	AGTCCAAAGCATATGCCAGTGTTGTGAGTTTGTTTTGGATGCACAGTATACTGACTCAAGAAACTGGCATATGTTTTGGACA	-44.3	-	Novel-mir-815-3p	-	TGACTCAAGAAACTGGCATAT
Novel-mir-814	AGTCCAGTTTGTGGACCACAGGATCTCGTCTCTGCTTTTTGCAGATGACGTTGTCCTGTTAGCTCCATCAAACAAGGAC	-38	-	Novel-mir-814-3p	-	TGACGTTGTCCTGTTAGCTCCATC
Novel-mir-813	CGGGAAGTGATCCAGACAAGAGCTGAGGGAAGCAGGTTCCCCTGGATGAGGTTTGTCACCAACATGCTGACCGAGGACTTCTGGGTGACATCGGACA	-28.6	-	Novel-mir-813-3p	-	TGACCGAGGACTTCTGGGTG
Novel-mir-811	ACCATAAACATGAATTCTGTCCCTGACCACAATCGTCAGGTTGACAATCTCTGGGGCAGCATATATGCACCAAGC	-18.4	-	Novel-mir-811-3p	-	TGACAATCTCTGGGGCAGCATATA
Novel-mir-810-1	AGCTTTGGAAAAATGATCAACGTGAGAATGACAGGGTTGCCCTGGGAGGGAGGTAGGCCGGTGACAAAGTCTAGGGCGATATGAGTCCAGGGCC	-30.5	-	Novel-mir-810-3p	-	TGACAAAGTCTAGGGCGATATGA
Novel-mir-810-2	CGGTCGACGATGGTGAGAATGACCATGTTGCCCTGGGATGGAGGCAGGCCGGTGACAAAGTCTAGGGCGATATGAGACCAGGGCC	-29.5	-	Novel-mir-810-3p	-	TGACAAAGTCTAGGGCGATATGA
Novel-mir-808-1	AACAAAGACAGGTATCAGAATCAGAGTCAGCTTCACTGGCCAAGTATCTTGACCACACAATGAATTTGACTCTGGTACAGTGGCTCTTAGTGTG	-26	-	Novel-mir-808-3p	-	TGAATTTGACTCTGGTACAGTGGC
Novel-mir-808-2	ATTTCTGGATGCTAATTGTCTAAAGATCAGAATCAGAATTAGAACTGAATTTGACTCTGGTACAGTGGCTCTCAATGTG	-19.3	-	Novel-mir-808-3p	-	TGAATTTGACTCTGGTACAGTGGC
Novel-mir-808-3	TAATTCAAGGTCCTGTATTCAGAATCAGCTTTATTGGCCAGGTGTGTTGGACACACAATGAATTTGACTCTGGTACAGTGGCTCTTAATGTG	-28.7	-	Novel-mir-808-3p	-	TGAATTTGACTCTGGTACAGTGGC

Novel-mir-806-1	GATGATGGTGTTGTCATGGTTGGAGACACAGTAATGGGTGTACAGGGTGAAGAGTCTGGGGCTGAGACAGCAGCCCTGCGG	-20.3	-	Novel-mir-806-3p	-	TGAAGAGTCTGGGGCTGAGACAGC
Novel-mir-806-2	GGGTGAGAGGGTGACGGTTCAGCCCAGCTTTTCTGTGCTGCCTTCACAGTCGTGGGTGTACAGGGTGAAGAGTCTGGGGCTGAGACAGCAGCCGTGCGG	-37.5	-	Novel-mir-806-3p	-	TGAAGAGTCTGGGGCTGAGACAGC
Novel-mir-805	CGGGAGATTGACAGGCGGATCGGTGCAGCTTCTACAGTAATGCGGTTGATGTACCAGTCCGTCGTGGTGAAGAAGGAGCTGAGCTGTAAGTTGAAGCTCTC	-34.2	-	Novel-mir-805-3p	-	TGAAGAAGGAGCTGAGCTGTAAGT
Novel-mir-804	AAGTGGATTCGTGAGTAACTCATTTTGTTGTACTGGATGCACAGTGAACTTGTAATGTGTGGACACGCGTCAAAACAA	-19.3	-	Novel-mir-804-3p	-	TGAACTTGTAATGTGTGGACACGC
Novel-mir-802	AAATGCTTAAAATCGATTTTTTTGTGTGCAGTTTGTTGAGCTTTGCAGCTTCACCAGCGTTGAACTCGGGAAGAAATTTGACAGAAGCAATA	-20.1	-	Novel-mir-802-3p	-	TGAACTCGGGAAGAAATTTGAC
Novel-mir-801	AAGGGTGAGGGTTTGAGGTCTTTAGGGTCCGGGCCAGGGGACAGAAAAAGAGTACCCAGCTGAACAGAGGACTCAGGACAAGGGCAGCATCGGC	-29	-	Novel-mir-801-3p	-	TGAACAGAGGACTCAGGACAAGGG
Novel-mir-800	GTGATCATGGGCCTCTTGGCTGCATCTCTGATCAGTCTTCTCCTTGTATGAGCTGAAAGTTTAGAGGGACGGCCAAGTCTTAGTCA	-28.2	-	Novel-mir-800-3p	-	TGAAAGTTTAGAGGGACGGCCAA
Novel-mir-798	AGCCAGAGGGCAAAGAAGTGAACAGTCCGTGCCAGGGGTGGGTTGGATCTTTGAGGACGGAGGCAGCTTTCCTACGC	-21.4	-	Novel-mir-798-3p	-	TCTTTGAGGACGGAGGCAGC
Novel-mir-796	CCGATGTCCTGGCTGTAGATCCTAGTGGTGTGGATCAGTGTGAATCGATCTTGTTCACTCCTGGCATACAGCTTGATGTCAT	-30.4	-	Novel-mir-796-3p	-	TCTTGTTCACTCCTGGCATACAGC
Novel-mir-795-1	ATGCTGGTTTCCGAGCACTAATTCGGCCATTGAGACCATTGTGTGAGAGAATTTGACAAACTGTTCTTGTAGATACAGGGGTTTCTGGTGACCAGTTT	-29.5	-	Novel-mir-795-3p	-	TCTTGTAGATACAGGGGTTTCTGG
Novel-mir-795-2	ATGCTGGTTTCTGAGCACTAGTTCGGCCATTGAGACCACTGCATGAGAGAATTCGACAAACTGTTCTTGTAGATACAGGGGTTTCTGGTGACCAGTTT	-28.6	-	Novel-mir-795-3p	-	TCTTGTAGATACAGGGGTTTCTGG
Novel-mir-795-3	TAAATTGGTCATTGAGACCACTGCATGAGAGAATTCGACAAACTGTTCTTGTAGATACAGGGGTTTCTGGTGACCAGTTT	-27.8	-	Novel-mir-795-3p	-	TCTTGTAGATACAGGGGTTTCTGG
Novel-mir-794	TAACTATCTTGTCTGTCTGCATCTCTAAGTTAGTGCCTGTGCCTGTATGCCAGTCTTGTAGAGCGAAGCAGACACACAACAACACCC	-22.8	-	Novel-mir-794-3p	-	TCTTGTAGAGCGAAGCAGACACAC
Novel-mir-790	AAAATGTGTCCCAGCCAGGATAATTAAGGAGGTCATTGACACTGTGGGGCCCACTTTGCTCATATTTCTTAACTGCTGTCTTAGGCTGGGCATGGAAC	-33.4	-	Novel-mir-790-3p	-	TCTTAACTGCTGTCTTAGGCTG
Novel-mir-789	TTCAGGCAGGTGACCAGGCTCTTCTTGGGAAGACAATGTTTGTGGTCTTAAAGCAGGTGGGGATGATGCTCTGGCTGAG	-23.1	-	Novel-mir-789-3p	-	TCTTAAAGCAGGTGGGGATGATGC
Novel-mir-788	TTCAGGCAGGTCACCGGGCTCTTCTTGGGAAGGGGGACGATGGTTGTAGTCTTAAAGCAGGAGGGAACGATGCTCTGGCTGAG	-29.6	-	Novel-mir-788-3p	-	TCTTAAAGCAGGAGGGAACGATGC
Novel-mir-787	CACAGGTCAAAAGGAGGGCAGAGAGGATCATTGCAGACCCCTCACACCCTGGACACAATCTGTTTGAACTTCTCCCCTCTGGATGGCGTTAC	-29.2	-	Novel-mir-787-3p	-	TCTGTTTGAACTTCTCCCCTCTGG
Novel-mir-786	CACGGGTCAAAAGGAGGGTGGAGAGGATCACTGCAGACCCCTCACACCCAGGACACAATCTGTTTGAACTCCTTCCCTCTGGACGGCGCTAC	-27.1	-	Novel-mir-786-3p	-	TCTGTTTGAACTCCTTCCCTCTGG
Novel-mir-785-1	TGTACACCTCACAGGGCAAAAGGAGAGGGAAGATTTCTGCAGACCCCTCACACCCTGGACACAATCTGTTTGAACTCCTCCTCTCTGGCTGGTGCTAG	-25.1	-	Novel-mir-785-3p	-	TCTGTTTGAACTCCTCCTCTCTGG
Novel-mir-785-2	TGTACACCTCATGGGTCAGATGGGGGGCAGAGAAGATTTCTGCAGACCCTTCACACCCTGCACACAATCTGTTTGAACTCCTCCTCTCTGGACAGTGTCAC	-28.1	-	Novel-mir-785-3p	-	TCTGTTTGAACTCCTCCTCTCTGG
Novel-mir-784-1	GCCAGCGCCTGCTCGGCATCGTCGTGTACTGAGCCCTTCCACTGCCGGTCTGTTTATCACGGCGTTTGAACAGGCAATTGC	-26.6	-	Novel-mir-784-3p	-	TCTGTTTATCACGGCGTTTGAAC
Novel-mir-784-2	GGTGGTGGCCTGCGGGCGCCTGCCTGGCGCTGTCGGACACTGAACCCTTCCACTGCCGATCTGTTTATCACGGCGTTTGAACAGGCAATTGC	-32.9	-	Novel-mir-784-3p	-	TCTGTTTATCACGGCGTTTGAAC
Novel-mir-784-3	TGCCGGGGCCTGCTCGGCACCGTCATGTACTGAACCCTTCCACTGCCGGTCTGTTTATCACGGCGTTTGAACAGGCAACTGC	-24.6	-	Novel-mir-784-3p	-	TCTGTTTATCACGGCGTTTGAAC
Novel-mir-781	CATTTTGTGATACCACATACGTCCCATCCAGGCTGCCACCTGTCTGTGAGGATGTATGTATGGCCAAACTATTGT	-22.1	-	Novel-mir-781-3p	-	TCTGTGAGGATGTATGTATGGCC
Novel-mir-780	ATTTTGTGATACCACATACGTCCCATCCAGGCTGCCACCTGTCTGTGAGGATGTATGTATGGCCAAACTATTG	-22.1	-	Novel-mir-780-3p	-	TCTGTGAGGATGTATGTATGGC
Novel-mir-775	CCATTCGGCTTGCATGGAGCACCTGCAACCTTTCAGTGGTTGGTGGACGAGGTTCTGAGAGGAGCAGAGGACTATGCAGCTGCTTAC	-33.1	-	Novel-mir-775-3p	-	TCTGAGAGGAGCAGAGGACTATGC
Novel-mir-773	GGGCCTGAAGACTCTGATATCTTCTCCCTGAAGGCAGGAGTCTGAAGTGGGAGTGTGCAGGGTGAGAAGTGAC	-22.7	-	Novel-mir-773-3p	-	TCTGAAGTGGGAGTGTGCAGGGT
Novel-mir-772	CGGAGTGATATGGGCATGCCAACGAGTGTTTGTCAGCAGACGGGCAGCTGCGTTCTGAACAAGTTGGAGCCTGGAGAGTGATGAC	-22.8	-	Novel-mir-772-3p	-	TCTGAACAAGTTGGAGCCTGGA

Novel-mir-771	CCACGTTCACAGTGTACTACATCTATTATCGGCTTCTCAGGGGTTAAACAGATGAGTGTCCGTTCTCTGAAGAAGTTCCTTGTAGGTGGAACAATC	-24.8	-	Novel-mir-771-3p	-	TCTCTGAAGAAGTTCCTTGTAGG
Novel-mir-770-1	ACAACTAGCCATCCTCACTGCAGAACACCAGAGGGATTGGGACATGCACCTCCCTCTCGTCTTACTGGCCTACAGGTCGGCTGTACAG	-27.5	-	Novel-mir-770-3p	-	TCTCGTCTTACTGGCCTACAGGTC
Novel-mir-770-2	ACAACTCGCCATCCTCACTGCAGAACACCAGAGGGATTGGGACATGCACCTCCCTCTCGTCTTACTGGCCTACAGGTCGGCTGTACCG	-25.7	-	Novel-mir-770-3p	-	TCTCGTCTTACTGGCCTACAGGTC
Novel-mir-769	CAGAGAGCTGTCAGCAATGCTAAGTCTCAGTATCTATCCCAAATTATCTCGGAGAATAGGCATCAGCCGAGAGTTCTTTT	-21.9	-	Novel-mir-769-3p	-	TCTCGGAGAATAGGCATCAGCCGA
Novel-mir-768	TGGGTTATTTTGGTGCCGCTGCTGCGCTGAAGGACGGCTGTATATTCATGAGCTCTCCTTTGAGTCAGTCGGCCCCAGATCTGCCC	-36.1	-	Novel-mir-768-3p	-	TCTCCTTTGAGTCAGTCGGCCCC
Novel-mir-767	TGTGGTGCAACGTGGCGTTGGTGAATGGAGCGAGAGTCTGAGGATCTCATCTCGGTACCTAATGGCAGTCACGCTACC	-26	-	Novel-mir-767-3p	-	TCTCATCTCGGTACCTAATGGCAG
Novel-mir-764	CCTGTTGGCCGACGTCTATGGTCCTGATGGGCATGTTTGGGCAGAGATCTCAAAACAGGATTGTAGACGGATTACAAGTTG	-27.3	-	Novel-mir-764-3p	-	TCTCAAAACAGGATTGTAGACGGA
Novel-mir-760	TATAAGCTTGTTGGTCTCAGTGATGGTCGTAGTAGGGATTGTTCTTAGTGCTGCATTCACATCCTCTAGTAGACTGTCAGAAGGCACTTCACTTAGT	-21.8	-	Novel-mir-760-3p	-	TCTAGTAGACTGTCAGAAGGCAC
Novel-mir-252-2	CCTCATTGTAACATGAGGTTTAGCTCCTCCATGATCATATCTCTCTTCCTCCTCTTCCTCACTTCTTCTAGGAGTAGACACCTTACTGGCCACAATCAGA	-18.15	-	Novel-mir-252-5p	-	TCTAGGAGTAGACACCTTACTGGC
Novel-mir-758	GACTGTTGTATCATCTGTGAAGTTGAGGAGTTTAACACAGGGGTCCCCTGAGGTGCAATCGTTTAGGGAGAACAGCAGAGGGGAGAGCAAAC	-22.5	-	Novel-mir-758-3p	-	TCGTTTAGGGAGAACAGCAGAGGG
Novel-mir-755	ATAAAAATGAGCTAGAGCTAGCATCTGCTTCCGGGAGAGGGCCTTGGTCGGGTAGAGTGAGCAGCATGAGCACCACCAACA	-20.4	-	Novel-mir-755-3p	-	TCGGGTAGAGTGAGCAGCATGAGC
Novel-mir-754	ATTAGCACAGCTAACTTATTAGCAAGCGAGTTCCGACTTTGATTTCTCTGACTGCATCGCTTCGGCTGTGATTTAGCATCCGCTACT	-19.8	-	Novel-mir-754-3p	-	TCGCTTCGGCTGTGATTTAGC
Novel-mir-747	CGGACACGGCGGGCCCGTTCTTCAGGGGTCAGACGGGCATGCCCTAATTCCATGGGTTCATCCGTAGGTCTCTGGAAGGATGTGTTGGTCCC	-38.3	-	Novel-mir-747-3p	-	TCCGTAGGTCTCTGGAAGGATG
Novel-mir-746	TCTCCTGGGTTTTCCCTGGGGCCTTTTCCTGGTGGGACATGCCCGGAACACCTCCCTAGGAAGGCGCTCAGGAGGCATCCAAAACA	-34.8	-	Novel-mir-746-3p	-	TCCCTAGGAAGGCGCTCAGGAGGC
Novel-mir-743	AGACTTGAGAAGACAGACACAGACCCCAGAAACTCTGCAGAGAAATCCAGGTGGTTCTGTTTCTCTCCCTCTCAATCC	-22.8	-	Novel-mir-743-3p	-	TCCAGGTGGTTCTGTTTCTCTCC
Novel-mir-741	CCTGTTGAGAGATGCTCATCACAACATTGTTCAGTGTTTGTACATGTTCACACAAGGCCTCATGTTGTACTAATGCTGGTGGGGAATCTGCAG	-19.3	-	Novel-mir-741-3p	-	TCATGTTGTACTAATGCTGGTGGG
Novel-mir-736-1	GTCCTCCCACAGCCAAGTTCAGCTCAGATGGCTGAGCTCATTGCTCTCATACAAGCATGTAAGTTGGCAAAAGATAAAG	-20.8	-	Novel-mir-736-3p	-	TCATACAAGCATGTAAGTTGGCA
Novel-mir-736-2	GTGCTCCCACAGCCAAGTTCAGCTCAGATGGCGGAGCTCATTGCTCTCATACAAGCATGTAAGTTGGCAAAAGATAAAG	-22.3	-	Novel-mir-736-3p	-	TCATACAAGCATGTAAGTTGGCA
Novel-mir-736-3	GTGCTCCCACAGCCAAGTTCAGCTCAGATGGCTGAGCTCATTGCTCTCATACAAGCATGTAAGTTGGCAAAAGATAAAG	-21.7	-	Novel-mir-736-3p	-	TCATACAAGCATGTAAGTTGGCA
Novel-mir-735	CCAGTTCGGTTGCCAATGAGAAGGTGACCCTAAGGGATCAGTTTGTAGAAGGGGTAAGCGATCCGGCACTG	-20.2	-	Novel-mir-735-3p	-	TCAGTTTGTAGAAGGGGTAAGCGA
Novel-mir-733	AAAGGATTGCTGGCACAATTATCAAAGGACTTGTTGAGAGACATTCTTTTATGTGTACTCAGTTGTAATGTGTAGTCATGCTGTTTCCACC	-19.7	-	Novel-mir-733-3p	-	TCAGTTGTAATGTGTAGTCATGC
Novel-mir-732	CAGCCAGCACTAAAGAGTTATTTTAAAGTCTTACTCCCAAGACAGTGATCACGTTACCTCAGTTGGAATAACTTGTACTAGTGGTTGGTGC	-26.2	-	Novel-mir-732-3p	-	TCAGTTGGAATAACTTGTACTAG
Novel-mir-731	AGCTGTGACTGCTGCAAGCTCTAGCTGTTCTCTCGGGGACAGATCAGTGAGGACTTGGAGAGCCGGTCCCTCCAGGG	-25	-	Novel-mir-731-3p	-	TCAGTGAGGACTTGGAGAGCCGGT
Novel-mir-728	GTTTACGCTACGGGTTGACAACATGTCATTCCTTATTTATAAGTAGCTTGGCTAATTGAATTCAGAGAATACCGGGTATCAGCACGAGCACAGAT	-19.3	-	Novel-mir-728-3p	-	TCAGAGAATACCGGGTATCAGCAC
Novel-mir-726	CAGAAGGGGGCTCCGCTGACATTTGTACATTCTGACTTTTTTCTCCAAATTCTGACTTTAAAGTCAGAAGTATGGGTACCTGTAGGGATCCAAGTTC	-26	-	Novel-mir-726-3p	-	TCAGAAGTATGGGTACCTGTAGGG
Novel-mir-724	CCTTAGAAGGACACTGTTTTTGGAGATAAAAGTGTAACTTTCACTCAGTAATGGTGTGTTCACTTATGTACTTATGAGGATAGCCTTCATGCA	-23.8	-	Novel-mir-724-3p	-	TCACTTATGTACTTATGAGGATAG
Novel-mir-720	AAATGTGCTGTGTTGACCAGTCATACAGGGGATGATAGGGACATGGACATGCAGTCGATAAATTTCAATCAGGCTTTAAGACTCGTCATAGCACTGAA	-25.6	-	Novel-mir-720-3p	-	TCAATCAGGCTTTAAGACTCGTCA
Novel-mir-719	GGTAGTGGGTGGCGGTCTGGCACTGTTTTCTGGTTTAGCAAACGGTAATCAATACACAGTCTCAGCGTGCCATCTCGTTTT	-22.7	-	Novel-mir-719-3p	-	TCAATACACAGTCTCAGCGTGCC

Novel-mir-716	CTGGTGCCTTTGACATGGTGGACCATGACATTTTGATATCTCACCTTCAAGATTTAGTGGGCATTCGTGGTGCTGCCCTA	-18.2	-	Novel-mir-716-3p	-	TCAAGATTTAGTGGGCATTCGTGG
Novel-mir-711	GGCTGGAGATGGGCTGTTGCCAAAGACATGGATCTTCAGTTAGTAGTCAATAACCTCCTTATTGATGTCATTGTCCTTATACCATAAAAAGCA	-19.6	-	Novel-mir-711-3p	-	TATTGATGTCATTGTCCTTATACC
Novel-mir-709	ATGTCTCTCTGTTCTGCATTCTCCTCAACTTCTTGCCTGAGAGAAAACGGAAGATCTGTCTGCACAGTATTAAGTCTGGGAGGTCTGTTCACGACTGCTTT	-20.1	-	Novel-mir-709-3p	-	TATTAAGTCTGGGAGGTCTGTTCA
Novel-mir-708	GCACAAGACTGTTACATCCCCAGTGTAAAAAACCTAGCATATAAGTTATTAAGGCTGTCTGGGAGGGTGACGTCATGGGA	-20.7	-	Novel-mir-708-3p	-	TATTAAGGCTGTCTGGGAGGGTGA
Novel-mir-703	ACCGTGTTGCCGTGTGAGGGAGGCAGGCCGGTAACAAAGTCAAGGGCTATATGAGACCAGGGCCGACTAGGCGTAGTAAGCGG	-24.5	-	Novel-mir-703-3p	-	TATGAGACCAGGGCCGACTAGGCG
Novel-mir-702	ACTGAAAGGCTCTTCCAGGGGTGTCCTAGCCTCATAGTCTACACTGCTTGGACAGAGCAGACTATCGGTGATGTCAACCTGGCGCTGCTTTTCAG	-36.9	-	Novel-mir-702-3p	-	TATCGGTGATGTCAACCTGGCGC
Novel-mir-701	AGAGAGGACGGGGTTTAATTTTCTGAAGATGTATTTATAGACACCTGGTAGGTGTCCGGATTATCATCGTTGGGAGGACTGCTACTGTTACCAGT	-25.1	-	Novel-mir-701-3p	-	TATCATCGTTGGGAGGACTGCTAC
Novel-mir-699	AACAAATACAACTATGCACATTTATTTGACACAGAGGCATCAATTTGTGAAGTTGGGTGAATATCAATCGAGTGAGCTGGACATTGTATGTGGA	-20.1	-	Novel-mir-699-3p	-	TATCAATCGAGTGAGCTGGACAT
Novel-mir-698	ACAATCTATAGCTTTAGGTTATGACATTGTGAAAACATGTTTAAAAAACACACTTGCATGCAGTCTATAGAGGAAGAAGAACAAGAGGCTATGGAGATT	-21.16	-	Novel-mir-698-3p	-	TATAGAGGAAGAAGAACAAGAGGC
Novel-mir-695	GGCCGGAGCGTTGCAGAGCCCAAACGGCATCACCTGGAACTGCCACCCTTGTCCGATAGTGAATGCTGTTTTGGGGCGGGCCTCTGGTG	-35.8	-	Novel-mir-695-3p	-	TAGTGAATGCTGTTTTGGGGCGG
Novel-mir-693-1	GTTGGAATACTGCAATGCTCTGTATTTTGGAGTTCCTCAGTCTTTGCTCAACAAACTCCAATTAGTCCAAAATGCAGCAGCTCGGCTGCTATCTAA	-26.1	-	Novel-mir-693-3p	-	TAGTCCAAAATGCAGCAGCTCGGC
Novel-mir-693-2	GTTGGATTACTGCAATGCTCTGTATATTGGCGTTCCTCAGTCTTTGCTCAGCAAGCTCCAATTAGTCCAAAATGCAGCAGCTCGGCTGCTATCTAA	-25.14	-	Novel-mir-693-3p	-	TAGTCCAAAATGCAGCAGCTCGGC
Novel-mir-693-3	GTTGGATTACTGCAATGCTCTGTATTTTGGAGTTCCTCAGTCTTTGCTCAACAAACTCCAATTAGTCCAAAATGCAGCAGCTCGGCTGCTATCCAA	-30.6	-	Novel-mir-693-3p	-	TAGTCCAAAATGCAGCAGCTCGGC
Novel-mir-692	GTTTGGTTTTGGCGTATAGTGTTGGTTTGTACTGGCGGCATCCATACTGCAGTGGATGTAGTATTGGTCGATGAATGATTCCGGGTCCGATA	-26.3	-	Novel-mir-692-3p	-	TAGTATTGGTCGATGAATGATTCC
Novel-mir-690	TATCATAAGGATCAGTACCATGGTATAGTCTATACCTGTGTGTGTGTAGGCTGTGAGATGGTATTGATCCTTATGCTA	-43.8	-	Novel-mir-690-3p	-	TAGGCTGTGAGATGGTATTGAT
Novel-mir-689	TCTCTATCTCCCATGTCACAGAGGCAACCATACAGGAGGCGGGTAGGATGGTTTCTGGAGGGGTGTCCGAGGTAGAG	-26.8	-	Novel-mir-689-3p	-	TAGGATGGTTTCTGGAGGGGTGTC
Novel-mir-685	ATGTGCGGGCCCAGGATGGTGTTGTTGGCGGTCCAGGAGGATGTCTTTTAGCTCCTCTAGGAAGGTGAGGAGACGTTGGGTGTTGTAAGA	-28.1	-	Novel-mir-685-3p	-	TAGGAAGGTGAGGAGACGTTGGG
Novel-mir-684	AGGACCACATACATTTTGACCGCCTTTTCTGAATGGCCTTCGGGGAACACCTTTACGAGACATAGCTTAGAGCAGGAACGTGGAAGCTTGCTGTCT	-18.3	-	Novel-mir-684-3p	-	TAGCTTAGAGCAGGAACGTGGAAG
Novel-mir-682	CAAACAGGAACAAGGCCAGGTTGCATATCAACGCTGCCTGTCACAAGGGTAGATGAGACATGAGCCAGAGGTGTCCCTAAAAC	-20.1	-	Novel-mir-682-3p	-	TAGATGAGACATGAGCCAGAGGTG
Novel-mir-680	TAATTAAAAGGTGGGGCGACTGTTCATTTTCTGTTTGTGGCCCTAGACTATGGAATGCCCTGCCGGTTGAACT	-23.2	-	Novel-mir-680-3p	-	TAGACTATGGAATGCCCTGC
Novel-mir-676	TGCCATACTGTCCATTTGTGCGGTGATGTGTACAGGAGCAGAGGCGCTACTTGCACCGGAGCCAGCAGGGTCTATGATGGC	-25.5	-	Novel-mir-676-3p	-	TACTTGCACCGGAGCCAGCAGGGT
Novel-mir-675	ATTGACGTCACTTCCACTGTAGTGTGTGTAGTATGGGTGACGAGTGGTGCTTGTCTACTTAGTGATGGAGATATGTGGTTGTGCGTTAA	-28.2	-	Novel-mir-675-3p	-	TACTTAGTGATGGAGATATGTGGT
Novel-mir-675	CTGACGTCACTTCCACTGTAGTGTGTGTAGTATGGGTGACGAGTGGTGCTTGTCTACTTAGTGATGGAGATATGTGGTTGTGCGTTAA	-27.9	-	Novel-mir-675-3p	-	TACTTAGTGATGGAGATATGTGGT
Novel-mir-674-1	AACCAGTTGTTCCTGGAAGTGCCGAAGTCCAGACGGAAACATTTTTCAGTTCAGGCTCCTATACTGTGGAATGGTCTGCCATGGGAGATTAGATC	-22.8	-	Novel-mir-674-3p	-	TACTGTGGAATGGTCTGCCATGGG
Novel-mir-674-2	ACTCTATATTGTCCAGACTGACACACTGGGAAGACCGAGCTTTTTCAGTTCGGGCTCCTATACTGTGGAATGGTCTGCCATGGGAGATTAGATC	-31.1	-	Novel-mir-674-3p	-	TACTGTGGAATGGTCTGCCATGGG
Novel-mir-670-1	CCTCGTCACCTGGAACGCACCCTCACCTGTGCGGGGGCGGAGCTGCTGGAACTACCTGTGCCTGCTACCGAGGTACAGGGGGCGGCCCGGGTCACGGAC	-47.8	-	Novel-mir-670-3p	-	TACCGAGGTACAGGGGGCGGCCCG
Novel-mir-670-2	CCTCGTCACCTGGAATGCACCCTCACCTGTGCGGGGGCGGAGCTGCTGGAACTACCTGTGCCTGCTACCGAGGTACAGGGGGCGGCCCGGGTCACGGAC	-45.4	-	Novel-mir-670-3p	-	TACCGAGGTACAGGGGGCGGCCCG
Novel-mir-670-3	CCTCGTCGCCTGGAACGCACCCTCACCTGTACGGGGGGCGGAGCTGCTGTGCCTGCTACCGAGGTACAGGGGGCGGCCCGGGTCACGGAC	-45.5	-	Novel-mir-670-3p	-	TACCGAGGTACAGGGGGCGGCCCG

Novel-mir-670-4	CCTCGTCGCCTGGAATGCACCCTCACCTGTGCGGGGGCGGAGCTGCTGGAACTACCTGTGCCTGCTACCGAGGTACAGGGGGCGGCCCGGGTCACGGAC	-45.7	-	Novel-mir-670-3p	-	TACCGAGGTACAGGGGGCGGCCCG
Novel-mir-667	CCGCTGGAAGACTCAGTGTTTTGGACCGATAGCACAACAGTGCTAAAGTACATTGGCAACGAGACATCAAGGTTTCACACGT	-23.11	-	Novel-mir-667-3p	-	TACATTGGCAACGAGACATCAAGG
Novel-mir-663	AATTAGTCCACTTCAACGTGAGTTCTCAGTGTTCAGACTCTTTATTTTCTCGTCTCCATACAGAACAACTCTCTCTCTGAGGTGTACTACCT	-21.32	-	Novel-mir-663-3p	-	TACAGAACAACTCTCTCTCTGAGG
Novel-mir-662	GGGGTGTCAGATAAGGCGCACAGTGACACTTGTGGCTGTCTGTGAAACGAGCATCGGGTGACTCCTTTACACAAACAGCTGCAAGGACACACCAGTGAT	-37.1	-	Novel-mir-662-3p	-	TACACAAACAGCTGCAAGGACA
Novel-mir-659	AGGGGGTAATGGATATTTGTCCTTAACTGTTATAGCATTAAGTCCGCGGTAATCAATGCAAGGACGGAGGCCGCCGTCCA	-21.4	-	Novel-mir-659-3p	-	TAATCAATGCAAGGACGGAGG
Novel-mir-658	AGAGGAGAGGTAATGGGTATTTGTTTTTAATGGTTATGGCATTGATACCACAGTAATCAATACATGGACGGAGGCCACCGTCCTT	-20.1	-	Novel-mir-658-3p	-	TAATCAATACATGGACGGAGGC
Novel-mir-657	CACGTGCTTTCCACAGTGGCTTGTCCCTGAACTGGGTCTGGTACAACGACTCAGGAATAATAGTTCTGGCTGCCCCTGTGTCTATTTCCAT	-24.7	-	Novel-mir-657-3p	-	TAATAGTTCTGGCTGCCCCTGTGT
Novel-mir-656	ATTAGTATTGGTGACTGTCTCTATTCTAGTAGCTAGGGCTTTGGCTTGATTTTTAAGTCTGAATTAATAAGTGATAAGGGACGGTAACCAGAAGGGTG	-25.3	-	Novel-mir-656-3p	-	TAATAAGTGATAAGGGACGGTAAC
Novel-mir-655-1	CCATAGCTTTCCTTTTGTACCGCACGTATGATGACTTGGGACCCCATAAGTATGGTATCAGGTCCTAGAGGAAGCTTACA	-21.9	-	Novel-mir-655-3p	-	TAAGTATGGTATCAGGTCCTAGAG
Novel-mir-655-2	CCATAGCTTTCCTTTTGTACCGCATGTATAATGACTTGGGACCCCCTAAGTATGGTATCAGGTCCTAGAGGAAGCTTACA	-24.6	-	Novel-mir-655-3p	-	TAAGTATGGTATCAGGTCCTAGAG
Novel-mir-653	ACAGTAGATCCTCCGTACCTCATACTATTGATGGAGTCTCTGATCCTGATAAGATAGCTGAGTTTTGGCGGCGTCATTACTGC	-20.3	-	Novel-mir-653-3p	-	TAAGATAGCTGAGTTTTGGCGGCG
Novel-mir-651-1	ACTAGAGAGAAGAGTTGATGACTGTCAGCAGACAGGGTGTTTAATATTATTATTATGACATGTATAACTGTAGACTTAGCCATAATGTTCTTATGTTT	-18.1	-	Novel-mir-651-3p	-	TAACTGTAGACTTAGCCATAATGT
Novel-mir-651-2	CACCAGAGAGAAAAGTTGATGACTGTCAGCAGACAGGGTGTTCAATATTATTATTACGACCTGTATAACTGTAGACTTAGCCATAATGTCCCTATGCTC	-18.1	-	Novel-mir-651-3p	-	TAACTGTAGACTTAGCCATAATGT
Novel-mir-649	TGGAATGTGCTACAAGCCAGGTGTGTGAGTGTTGTGATGTCAGGCGGTAGATAACAATGAATACTGGACAATTGGTTGTCCG	-18.5	-	Novel-mir-649-3p	-	TAACAATGAATACTGGACAAT
Novel-mir-647	ATAATGCAATAATCACTCACATTGCAACATTTAGACACTGGTAGGAGACACTGGTATACTTGCCTCTTAAATGGATAGTAGTGGACTGGTTGATGTTTTGT	-25.6	-	Novel-mir-647-3p	-	TAAATGGATAGTAGTGGACTGGTT
Novel-mir-644	TTGGATCTGCACTTGGGCATTTTTGTTATGTCCGCTCGAACATGGAGGAGAGGGAGTAAAGAGAGACGGACCCAGGTAGCTGACTTCGG	-25.2	-	Novel-mir-644-3p	-	TAAAGAGAGACGGACCCAGGTAG
Novel-mir-642	ATCCGGGTTGAGGTAGTAGGTTGTATGGTTTAGAATTACACCCTGGGAGTTAACTGTACAACCTTCTAGCTTTCCTTGGA	-31.4	-	Novel-mir-642-3p	-	GTTAACTGTACAACCTTCTAGC
Novel-mir-639	ATGGTCCGTGTTTATCACGATTGCTCATCATTTGTGCTGCATCACGGTGCGTGTTACAGTATAATGTCTGTAGGTGGCGCTCGGCTTA	-21.3	-	Novel-mir-639-3p	-	GTATAATGTCTGTAGGTGGC
Novel-mir-636	GCTTCAAGTATACTTTACTTTAACTTGCAGTTTCTATTGAAAGCTTTTTTCGGTTCTTTTAACCAGTGGAACTCAGAGAGATGAAGCGGACAACTTGGATA	-18.2	-	Novel-mir-636-3p	-	GGAACTCAGAGAGATGAAGCGGAC
Novel-mir-635	TTCCTGTCTTCCCGGAATGGAGTGAGAAGGGGGGCCCTGGTAGACGTCAAGATGGACAGAGTCCAGCTGTTGAGAAGTAGCTAGTGGCTGAGCGGATA	-26.9	-	Novel-mir-635-3p	-	GCTGTTGAGAAGTAGCTAGTGGC
Novel-mir-634	ACTGCTTTTTTTCTCGCGATTGTTTCCCGTGTGGGTTAATGGCTCCTCCTGCTACAGACTGAGACGGAGCTGGCACGAAGCAGC	-24.21	-	Novel-mir-634-3p	-	GCTACAGACTGAGACGGAGCTGGC
Novel-mir-633-1	TAACGCTCCAGCCACCTTTGAGAGATTGATGGGGCGGGTCCTCAAAGACATTCCCAGAACCCGCTGCGTAGTTTACCTGGACGACTTGCTGGTGCACAC	-29.52	-	Novel-mir-633-3p	-	GCGTAGTTTACCTGGACGACTTGC
Novel-mir-633-2	TAACGCTCCAGCCACCTTTGAGAGATTGATGGGGCGGGTCCTCAAAGACATTCCCAGAACCCGCTGCGTAGTTTACCTGGACGACTTGCTGGTGCACGC	-29.52	-	Novel-mir-633-3p	-	GCGTAGTTTACCTGGACGACTTGC
Novel-mir-632	TGCCTGCCCTTAGAAACCAAGTGTGGGTGTGGCCTGAAAAACAGATATGGCGACCCATCCTTTGTTTCTGAGGTCCTGCA	-31.24	-	Novel-mir-632-3p	-	GCGACCCATCCTTTGTTTCTG
Novel-mir-631-1	TCTCTCCTCTATCCACTCACCCCAACCGGTCGAGGCAGAAGGCCGCCCACCAAGAGTCGGGGTCTGTCAGAGGTTTCT	-23.1	-	Novel-mir-631-3p	-	GCCCACCAAGAGTCGGGGTCTGTC
Novel-mir-631-2	TCTCTCCTCTATCCACTCACCCCAACCGGTCGAGGCAGAAGGCCGCCCACCAAGAGTCGGGGTCTGTCGGAGGTTTCT	-23.1	-	Novel-mir-631-3p	-	GCCCACCAAGAGTCGGGGTCTGTC
Novel-mir-631-3	TCTCTCCTCTATCCACTCACCCCAACCGGTTGAGGCAGAAGGCCGCCCACCAAGAGTCGGGGTCTGTCAGAGGTTTCT	-23.1	-	Novel-mir-631-3p	-	GCCCACCAAGAGTCGGGGTCTGTC
Novel-mir-631-4	TCTCTCCTCTATCCACTCACCCCAACCGGTTGAGGCAGAAGGCCGCCCACCAAGAGTCGGGGTCTGTCGGAGGTTTCA	-23.1	-	Novel-mir-631-3p	-	GCCCACCAAGAGTCGGGGTCTGTC

Novel-mir-631-5	TCTCTCCTCTATCCACTCACCCCAACTGGTCGAGGCAGAAGGCCGCCCACCAAGAGTCGGGGTCTGTCAGAGGTTTCT	-24.8	-	Novel-mir-631-3p	-	GCCCACCAAGAGTCGGGGTCTGTC
Novel-mir-631-6	TCTCTCCTCTATCCACTCAGCCCGACTGGTCGAGGCAGAAGGCCGCCCACCAAGAGTCGGGGTCTGTCGGAGGTTTCT	-26.2	-	Novel-mir-631-3p	-	GCCCACCAAGAGTCGGGGTCTGTC
Novel-mir-631-7	TCTTTCCTCTATCCACTCACCCCAACCGGTCAAGGCAGAAGGGCGCCCACCAAGAGTCGGGGTCTGTCAGAGGTTTCT	-21.5	-	Novel-mir-631-3p	-	GCCCACCAAGAGTCGGGGTCTGTC
Novel-mir-631-8	TGTCTCCTCTATCCACTCACCCCAACTGGTCGAGGCAGAAGGCTGCCCACCAAGAGTCGGGGTCTGTCGGAGGTTTTT	-28.4	-	Novel-mir-631-3p	-	GCCCACCAAGAGTCGGGGTCTGTC
Novel-mir-631-9	TTTCTCCTCTCTCTTCTTTATCCGCTCACCCCACCCGGTTGAGGCAGAAGGTCGCCCACCAAGAGTCGGGGTCTGTCAGAGGTTTCT	-20.34	-	Novel-mir-631-3p	-	GCCCACCAAGAGTCGGGGTCTGTC
Novel-mir-626-1	CGAGTGATCCACTCTTACCTTTTGTAGACTGATGTGCTACGTTGACCGAGGATATCAGGACAAAAATATAATCACTGT	-20.4	-	Novel-mir-626-3p	-	GAGGATATCAGGACAAAAATA
Novel-mir-626-2	CGAGTGATCCACTCTTACCTTTTGTAGACTGATGTGCTACGTTGACCGAGGATATCAGGACAAAAATATAATCATTGT	-20.4	-	Novel-mir-626-3p	-	GAGGATATCAGGACAAAAATA
Novel-mir-626-3	AGACACCGCTCCCATTCACCTGCGTTCCCACAGACTCCCCATCTCCAAACGCCAAGCTGCTGAGGAACTGATAGCGGACATGGCAGCTAATGGC	-20.67	-	Novel-mir-625-3p	-	GAGGAACTGATAGCGGACATGGC
Novel-mir-623	GACCGGGCGTGTGTTTTGGGGTTTTTGGGAGGGAGCAAGCAAGCCTGACAGAGACAGACAACTCATGACTAAATAAGCTCTGAGACACTCAAACTGAGTG	-25.4	-	Novel-mir-623-3p	-	GACTAAATAAGCTCTGAGACACTC
Novel-mir-621	CATCGAGTCAGTTCTCTGCTCATCCATCACTGTCTGGTTTGGCTCAGCAACCAAACAGGACAAGTACAGACTCCAGAGGACAGCCAAGACT	-24.3	-	Novel-mir-621-3p	-	GACAAGTACAGACTCCAGAGGAC
Novel-mir-620-1	GCAGTCTCTGGGGACTGACTCCCTTACGGTGCCAGCCTCTAGTGGCCGTTTGAGGAACTGCAGGTTTTGGCACTTCCACATTGGCTTC	-30	-	Novel-mir-620-3p	-	GAACTGCAGGTTTTGGCACTTCCA
Novel-mir-620-2	GGAATCTATGGGGACTGACTTCCTTTTGGAGCCAGCCTCAAGTGGCCATTTGAGGAACTGCAGGTTTTGGCACTTCCACACTGGATTC	-27.5	-	Novel-mir-620-3p	-	GAACTGCAGGTTTTGGCACTTCCA
Novel-mir-615-1	ACCCCACCTTGACTATAGCTCGCCTGAGCAACACGATAAGTACAAACATATACCAACTGCTATACAGAGACCTAAAGCACTGGGTGGGGG	-26.9	-	Novel-mir-615-3p	-	CTGCTATACAGAGACCTAAAGCAC
Novel-mir-615-2	ACCCCACCTTGACTATAGCTCGCCTGAGCAACACGATAAGTACGAACATATACCAACTGCTATACAGAGACCTAAAGCACTGGGTGGGGG	-26.9	-	Novel-mir-615-3p	-	CTGCTATACAGAGACCTAAAGCAC
Novel-mir-615-3	ACCCCACCTTGACTATAGCTCGCCTGAGCAACACGATAAGTACGAACATATACCAACTGCTATACAGAGACCTAAAGCACTTGGTGGGGG	-25.9	-	Novel-mir-615-3p	-	CTGCTATACAGAGACCTAAAGCAC
Novel-mir-615-4	AGCCCACCTCATCTTGACTATAGCTCGCCTTAGCAACATGATAAGTATAAACATATACAAACTGCTATACAGAGACCTAAAGCACTGGGTGGGGG	-23.8	-	Novel-mir-615-3p	-	CTGCTATACAGAGACCTAAAGCAC
Novel-mir-613	GTCAGTGTAGTGCCAAAGTGCTTACAGTGCAGGTAGTTTTATAAGATCTACTGCAGTGAAGGCACTTTCAGCACTGTTCTGA	-36.1	-	Novel-mir-613-3p	-	CTGCAGTGAAGGCACTTTCAGC
Novel-mir-609	ACCTGCTCTCCTAAAAGAGCTGCGGTCCACAGCCCCAGCAGTGCCGCTGGCCTCGTGCTTGGATCTGGGTCTGGAGGAGGAGGAGGA	-38.9	-	Novel-mir-609-3p	-	CGTGCTTGGATCTGGGTCTGGAGG
Novel-mir-607	CGAGCGGGGTCTTTGTGAAGCCGGATACGTCAGGCATTCTCACAGGATGGCGGACACTAACCTCTATACTTTGACCTCGTAGC	-26.6	-	Novel-mir-607-3p	-	CGGACACTAACCTCTATACTTTG
Novel-mir-605	TTTGCCCACTCAAGTCGGTTACAATGACGCACTGCAGTCAGGTCGAGACCCCGATAAGGATGACGAGCATGCAGATGAGCTTCCCTGAGACAGTTTC	-21.2	-	Novel-mir-605-3p	-	CGAGCATGCAGATGAGCTTCCCTG
Novel-mir-603	CCCGTGTTGGAGTTTACCCCGGTGAACGAGAGGGTCGCTTCCCTGCGCCTTTGGGTCGGGGATAGGTCTTTCACTGC	-27.7	-	Novel-mir-603-3p	-	CCTTTGGGTCGGGGATAGGT
Novel-mir-602	TGTTTATTATGGCGCACACATAGGAGGAGAGATGAATTCTGTACCCTGCATGCGCGCCTGTCTGCCTCTGTGTGTGTCTGTTCGTGTG	-26.7	-	Novel-mir-602-3p	-	CCTGTCTGCCTCTGTGTGTGTC
Novel-mir-595	TGTGTAGACATGGTCTAGGTGGGAGTGCGGGGTCATAAACAGAGAGTCTCCCCCATGTCTCTTGCTCTGCCTGCGTCTGTGC	-26.63	-	Novel-mir-595-3p	-	CCCCATGTCTCTTGCTCTGCCT
Novel-mir-592	TAGTGATCGGCTGGCTCTGATGTCCTTGCTGGCTGAGTTCATTTGGCCAGATTGTACTGTCAGGATCGGCGCGTGACGCT	-31.5	-	Novel-mir-592-3p	-	CCAGATTGTACTGTCAGGATCGGC
Novel-mir-590	GGGCTTCTGCTGTGATTAGCACGGGAGGAGTGGCTAATTACCACTTCTCCCGCGGAATCGCTATCAAGAGATT	-32.4	-	Novel-mir-590-3p	-	CCACTTCTCCCGCGGAATCGCTA
Novel-mir-589	CTCCTTTTTCTCCTCTCTTTCTCCTTTGTTTGACTTTGTTCTTCAGAGGCAGCCACAAAGGGAGAAGGAGAGGAGAAAGACAGA	-41.7	-	Novel-mir-589-3p	-	CCACAAAGGGAGAAGGAGAGGA
Novel-mir-586	TTCTCTGCTAGCTCTTTGTATTTTGGCTCCAGGTTCTTGCAGTGCCCACACCATGGAGCATAGAACTCGATCAGCACGTCTTTGC	-21.4	-	Novel-mir-586-3p	-	CATGGAGCATAGAACTCGATCAGC
Novel-mir-583	GTGTGAATTTCAAGTTGGTGGATCTTCTCCAGAAAGACCATGCAGAGCTCTACGGAGGTGCTCAGGTTGTCACTGTTGGAAGCTGTGGACTTCACA	-24	-	Novel-mir-583-3p	-	CAGGTTGTCACTGTTGGAAGCTGT

Novel-mir-582	CGGGTGTTTGTGCTGTGTCGCTGGAACTTGGCCCATCTGCGCTGCTGGGAAACAGTCCTGACAGGTTAACATCAGGGTCCGCAGGGTCTGCATCC	-29.1	-	Novel-mir-582-3p	-	CAGGTTAACATCAGGGTCCGCAGG
Novel-mir-581	ATCACATCTGTTGGTCAGCTATTAGTAGCCTGGGTAGTCCTCAGTATCCAGGTAACTGAGAGCCGACTGATTAATGTGAA	-39.2	-	Novel-mir-581-3p	-	CAGGTAACTGAGAGCCGACTGA
Novel-mir-573	CTCTACGCCCAGAAGTCCTGCACTGGGTCCATGGGGCAGCAGGAGCTGGTCACTATGGGAACTCCAAGACGGTGAGGCGACTTA	-29.1	-	Novel-mir-573-3p	-	CACTATGGGAACTCCAAGACGGTG
Novel-mir-571	TGGGGAATCCAGATGCTGCTGCAGTTGCTGTTGTTTTAGGATGAGACACACCAGGATCAGTAGACGGTGTGCTAGCTTGCCC	-21.5	-	Novel-mir-571-3p	-	CACCAGGATCAGTAGACGGTGTGC
Novel-mir-569	TCCCCTGTTTTCCTGGGGGCTATCGATGGGACTTGGTGCAGTCGCACAGTCCAGCACGACCACACATTGATTGGGCCATGGGCTCGCGGGGGG	-38.1	-	Novel-mir-569-3p	-	CACACATTGATTGGGCCATGGGC
Novel-mir-565	GGCAAGAGGCTGAGCTGTTTCTGCCCTGGCAGGATGAGCAGTGGGAAACTAGGGACAAGTCAGGCAAGAACCAGGCAGACCAAAGACC	-25.8	-	Novel-mir-565-3p	-	CAAGTCAGGCAAGAACCAGGCAG
Novel-mir-564	TAGTTGTCTATATGTACCCTGTAGAACCGAATTTGTGTGAAGTCCAAACAGTCGCAAATACGTCTCTACAGGAATACATGGGCAACT	-32.7	-	Novel-mir-564-3p	-	CAAATACGTCTCTACAGGAATAC
Novel-mir-560	CACATCGATACACTGCTTTGATAGATGTGACAGAAACAGACTGAATCTGTGTTCATGAGTCTACAGTATGTAAAACACTGAACAAGCAGGGTGTCTATGGC	-26.7	-	Novel-mir-560-3p	-	ATGTAAAACACTGAACAAGCAGGG
Novel-mir-557	GAACACAAAGTGTGCTTTAAAGAGTTCATCAAAGGCCGCCAATGATGTAGTTACCTGGCATGGGATGAGCTTCTTGTCCAGCACAATGTAGAA	-27.6	-	Novel-mir-557-3p	-	ATGGGATGAGCTTCTTGTCCAGCA
Novel-mir-555	CCATACCAGGGTTAGAGTTCAGCCTCAGCATTCCTTGTGTGGTCATTCTCATATTTTATGGAATGCTCAAGCTCATGTTACCTTCGTATG	-27.2	-	Novel-mir-555-3p	-	ATGGAATGCTCAAGCTCATGTTA
Novel-mir-553	CGGCATCAAGCTACGGTGCTAAAGAAAGATTCAAAAGACGCCAGTGCCATGAAACTATCGTTTGTAGAGGTTTTTCGCCCGTGTCTGCCT	-23.4	-	Novel-mir-553-3p	-	ATCGTTTGTAGAGGTTTTTCGCCC
Novel-mir-552	AGCGTGTAATCTTTCAGTGAAAACTCAGGCTTCCAGTTGTTGTACAGATTCTGTTAAAGGGAATAAAAAGCATGGTTGTCACAGGGCAGTGCAGGC	-21.1	-	Novel-mir-552-3p	-	ATAAAAAGCATGGTTGTCACAGGG
Novel-mir-551	GGCACCGGGCGATGACGTCACTTCCGGTTTAGCGATTTCCCGTTAGTAAGAAAGATGGCGGACGTAAGGCTCTTTTTT	-19.4	-	Novel-mir-551-3p	-	AGTAAGAAAGATGGCGGACGTAAG
Novel-mir-550	TCGGGCCACGCTCACATGCAGCAGTCAGTCCCACATATGCACATGTCAGCGTTGCAGCAGCAGCAGAAGGGAGTGGCTCTGCAGCAGAAGGTACAACAGAT	-33.5	-	Novel-mir-550-3p	-	AGGGAGTGGCTCTGCAGCAGAAGG
Novel-mir-549	AGAGATGTAGGATGTTGATGTTCTTTTTAGGAGGGACCAGGATGGACAGGATTAGGAATGAACACATCAGACGGACAGCTC	-18.4	-	Novel-mir-549-3p	-	AGGATTAGGAATGAACACATCAGA
Novel-mir-544	TATAGTGTACCGCCAGAGAGGCCACTGTACAAGTGTTGATAAAAGATGTGCGAAGTAGAGTTCCAAGATGGCGTTACTGAACACACAAGT	-18.5	-	Novel-mir-544-3p	-	AGAGTTCCAAGATGGCGTTACTGA
Novel-mir-543	AGAGACAAACGGATGCAGGTGAGATATCCATGGCTGCCACTCGGCAGAGTATGGAACCACTGGCATTCTGCTGTGGAT	-28.1	-	Novel-mir-543-3p	-	AGAGTATGGAACCACTGGCATTC
Novel-mir-542	GGCTGCTAAGACTATGTTGTGTCCTTTATGTTCATCCACAGGGCAGAGATAACAGATACACTGCTGGTCAGTGCG	-18.2	-	Novel-mir-542-3p	-	AGAGATAACAGATACACTGCT
Novel-mir-541	CAGGGCTCGACCCAGGCAGACACTCCTCATTCCTGTTCATCATATGAGACTGAAGACGTGTCTGTGGAGTCGGCCCCT	-33.3	-	Novel-mir-541-3p	-	AGACTGAAGACGTGTCTGTGGA
Novel-mir-540	AGGTGCATCACTGTGTGGTACGGAGCCTGTACTGCCTCCTGCAGAAGGACTCTGCAACACATAGTGAATACAGCC	-32.6	-	Novel-mir-540-3p	-	AGAAGGACTCTGCAACACATAGT
Novel-mir-539	GGCTGCATCACTGTGTGGTATGGAGCCTGTACTGCCTCCTGCAGAAGGACTCTGCAACACATAGTGAAAACAGC	-32.4	-	Novel-mir-539-3p	-	AGAAGGACTCTGCAACACATAG
Novel-mir-538-1	ACCTCAAGTGTATACCCAGAGCTCAGAGGGGTGAGGTGTGGGCCTCAGTCACCAACTTACTCAAGGAACACAGGGTTGTTGCAGAGCA	-22.9	-	Novel-mir-538-3p	-	ACTTACTCAAGGAACACAGGGTTG
Novel-mir-538-2	TCAAGTGTATTACCTAGAGCTCAGAGGGGTGAGGTGTGAGGTGTGGGCCTCAGTCACCAACTTACTCAAGGAACACAGGGTTGTTGCACAGAA	-30.1	-	Novel-mir-538-3p	-	ACTTACTCAAGGAACACAGGGTTG
Novel-mir-536	ATCCTGACTAAATGTTTAGCTGGACGGCTCAGATCCGTCATAGTCACCATTGTAGGCGACACACAGACCTGTAGCATTCCAGGCAGAGATGTCACAGA	-25.8	-	Novel-mir-536-3p	-	ACCTGTAGCATTCCAGGCAGAG
Novel-mir-534	GAAAGGTAAAGGAGTGTTACATGGACATTTTCAACTTAAATCTCAGAGTTGATTGAACCAAAGTCATTTGTAGCCACTGCAAAGCTGAAT	-18.51	-	Novel-mir-534-3p	-	ACCAAAGTCATTTGTAGCCACTGC
Novel-mir-531-1	CCAGCCAGCCTAACCAATGTGCAGACTACTGTACAACCACTACTTCCTGAACAGGTAGTCTGAACACTGGGCAGGCGGGCTG	-38.2	-	Novel-mir-531-3p	-	ACAGGTAGTCTGAACACTGGGC
Novel-mir-531-2	TCCCAGCCCAGCCTAACCAATGTGCAGACTACTGTTCAGCTGTAGCCTGATGAACAGGTAGTCTGAACACTGGGCAGGCGGGGGG	-42.5	-	Novel-mir-531-3p	-	ACAGGTAGTCTGAACACTGGGC
Novel-mir-6-2	GGCATTTATGCCAGCACAGCGGACCTGAGTGGAGACAGCCCAGTGCAGGGCACAGGGTGGGTAAAGAAATCTCTTCAGGACTGTGCATGCGTAAGGGATAA	-36.1	-	Novel-mir-6-5p	-	AATCTCTTCAGGACTGTGCATGCG

Novel-mir-528	TGGCTGCATCACTATGTGGTACGGAGCCTGTACTGCCTCCTGCAGAAGGACTCTGCAGCACATAGTTAATACAGCCA	-35.5	-	Novel-mir-528-3p	-	AAGGACTCTGCAGCACATAGTT
Novel-mir-526	CAAACTCAGTTCTATGTTTGCTGGAAAGGATGGTGTTGCAGCCTATTGTGAACTTTCTGAAGAACAAAAGAGCTGGTTCG	-21.4	-	Novel-mir-526-3p	-	AACTTTCTGAAGAACAAAAG
Novel-mir-525	ACTACGGGCCTTAATCTTTGGCCCCAGTTCTTTGGCTTGGTGCCCTGGATGAAGGGCAAGGGGAACATGTGGAACAGAGACAGTGTCCATCCT	-33.6	-	Novel-mir-525-3p	-	AACATGTGGAACAGAGACAG
Novel-mir-524	TATTATTTGTGCGTTGCCCACAACCTGCTTGACTGTAAAAGACAAAACAGTAGTCAGTTTCAGGAGCGCACTCTTTGCC	-18.5	-	Novel-mir-524-3p	-	AACAGTAGTCAGTTTCAGGAGCGC
Novel-mir-580	GATGATGAACCGTTTCCTGTGAGTTCAGCCTGGCCATGTGACCTGATGAAGGCCAGGGGTGATGATGCTGAGTAAGGTTTCGCATG	-23.86	-	Novel-mir-580-3p	-	CAGGGGTGATGATGCTGAGTAAG
Novel-mir-803	AGGTCTAGACCTTTGTAAAGTGTCCTGAGATTACTTTTGTTAAGCTTTGCAGCTTCATCAGCGTTGAACTCGGGAAGAATTTGACGGAGGCAATAATA	-26.5	-	Novel-mir-803-3p	-	TGAACTCGGGAAGAATTTGACGGA
Novel-mir-548-1	CCTAGGGCTGAATGGGAGGACCCAGAGCGCATTTTGGCTGTCTTGCAGGAGTTGTATGGGTGTTCCAAATCTTATG	-22.3	-	Novel-mir-548-3p	-	AGGAGTTGTATGGGTGTTCC
Novel-mir-548-2	TGAACGAGAGGACCCAGAGCGCATTTTGGCTGTCTTGCAGGAGTTGTATGGGTGTTCCAAATCGTATG	-19.9	-	Novel-mir-548-3p	-	AGGAGTTGTATGGGTGTTCC
Novel-mir-877	GAGGGCAGCCCCGATGCCAGGCAGGAGTCCGTGGTTGCGGTGGCGGCTGCTGCTGAAGGACCGGCTGGGACCTGGGCTGCAGGG	-38.7	-	Novel-mir-877-3p	-	TGCTGAAGGACCGGCTGGGACCTG
Novel-mir-573	CTCTCCGCCCAGAAGTCCTGCACTGGGTCCATGGGGCAGCAGGAGCTGGTCACTATGGGAACTCCAAGACGGTGAGGCGGCTTA	-31.3	-	Novel-mir-573-3p	-	CACTATGGGAACTCCAAGACGGTG
Novel-mir-679-1	TATGCATTTGTAGCACCCTTCTCACTCTACCTAGGAGGGGCACCAACTAGACGAGTTAGAATGAGGGGTGCTTGGGTTCGT	-29.5	-	Novel-mir-679-3p	-	TAGACGAGTTAGAATGAGGGGTGC
Novel-mir-679-2	TATTATTGTAGTACCCTTCTCACTCTACCTAGGAGGGGCACCAACTAGACGAGTTAGAATGAGGGGTGCTTGGGTTCGT	-25.4	-	Novel-mir-679-3p	-	TAGACGAGTTAGAATGAGGGGTGC
Novel-mir-848	GCTATGTGAGAACAGGGCCGGCTAGGCGTAGTCAGCGGCCGAAGGAGACCAGCGGGTGACTGATGTAAGGGCTTGGACTGAGCACAGGGA	-31.2	-	Novel-mir-848-3p	-	TGATGTAAGGGCTTGGACTG
Novel-mir-916	ATTGTGTCAGTTCTGATGTGCATGAGCGGCTTCTCAGAGGGAAGTGAAACGATGTGTGAGTGAGTGACTGGTGGAGCAGAAACACCAT	-20.6	-	Novel-mir-916-3p	-	TGTGAGTGAGTGACTGGTGGAGCA
Novel-mir-979	AACCGATTTACAACCTGTGTAATAGATGTTTTCTTGTTAGCCTCACGAGGTGTACATATTTTCTGAAGCTGAGTGGAAGGAGAGTCATTGGTA	-22.6	-	Novel-mir-979-3p	-	TTCTGAAGCTGAGTGGAAGGAGA
Novel-mir-554	AAGTGCTCTACTATGAGAACTGAATTCCATAGATGGTGGCTACTTCAGGTGTCATCTATGGGCTCAGTTCTTTTGGCATGGTACT	-37.2	-	Novel-mir-554-3p	-	ATCTATGGGCTCAGTTCTTTTG
Novel-mir-481-2	AACATCACACGTCCAGATCTCTCTCACCACCAAACCTGCGCGCAGGCAGCTTGGAAAGATGAAAGACCCTGGACCAAGAAACCT	-21	-	Novel-mir-481-5p	-	TTGGAAAGATGAAAGACCCTGGAC
Novel-mir-481-3	AACATCACACGTCCAGATCTCTCTCACTACCAAAACCTGCGCGCAGGCAGCTTGGAAAGATGAAAGACCCTGGACCAAGAAACCT	-21.7	-	Novel-mir-481-5p	-	TTGGAAAGATGAAAGACCCTGGAC
Novel-mir-481-4	AACATCACATGTCCAGATCTCTCTCACCACCAACCCGCGCGCAGGCAGCTTGGAAAGATGAAAGACCCTGGACCAAGAAACCT	-18	-	Novel-mir-481-5p	-	TTGGAAAGATGAAAGACCCTGGAC
Novel-mir-759	AGCCTCGTATTTCACTGGATGCTGGTCATGAGACCATAGATGAGTTATTAGCTTGGTTTAGCTCTACATGCACGGACATCCTTGACTCGTGTG	-22.5	-	Novel-mir-759-3p	-	TCTACATGCACGGACATCCTT
Novel-mir-725-1	ACAAAGGCCTTGTGGAGAGAGCAAAATTCAGTGCTGAATCGAAAGAATTTCACTTGATAGGACCTCTGCACGCAGATATTCGT	-19.2	-	Novel-mir-725-3p	-	TCACTTGATAGGACCTCTGCACGC
Novel-mir-725-2	ACAAAGGCCTTGTGGAGAGAGCAAAATTCAGTGCTGAATCGAAAGAATTTCACTTGATAGGACCTCTGCACGCAGATATTCTT	-19.2	-	Novel-mir-725-3p	-	TCACTTGATAGGACCTCTGCACGC
Novel-mir-718	TGGTCACTCTCTTGGCATTCACCGCGTGCCTTAATTGTATGGACATTTAAATCAAGGTCCGCTGTGAACACGAGGAGAGACGA	-29.1	-	Novel-mir-718-3p	-	TCAAGGTCCGCTGTGAACACGA
Novel-mir-738	CTGCAGTGTGCCACATTAAAGTGCTGCTGGGGCCCTACTGCAGGTTCTGGGTAAGGGGTCATCAGTGTGGGCTGGCATGGGTAACC	-27.2	-	Novel-mir-738-3p	-	TCATCAGTGTGGGCTGGCATGGG
Novel-mir-956	GGCATCTGTGTTTTATCCTCGTCGCCACTCTTGTTGTGTCTGTAGTATTTCTTCTATTTATACTAGACACACAAGAACAATCGGAGC	-19.44	-	Novel-mir-956-3p	-	TTATACTAGACACACAAGAA
Novel-mir-922	ATGGACAAGTCCGCTCCTGAGTCCCCAGCTCTGGCTTCCTTTATGGAAGGTGTGTCAGAGGGATTGAGGAGATCTTCAAAGT	-32.7	-	Novel-mir-922-3p	-	TGTGTCAGAGGGATTGAGGAGA
Novel-mir-1011	TGCGTCGTTTTGCTGGCCAGTTGTTCCTGTGTTTCTCACACACAGCTTGTAACACATTGTCAGCTGCAGTTGCTTCCTT	-18.7	-	Novel-mir-1011-3p	-	TTGTAACACATTGTCAGCTGCAG
Novel-mir-1000	CTTGGGAGGGTCTGCTCTCGCTATGGCCCGGTTGCATTGCTCCCTTGCTTTATAGGGAGGCCAGAAGCTTCTTCCATT	-27.5	-	Novel-mir-1000-3p	-	TTGCTTTATAGGGAGGCCAGAAGC

Novel-mir-978	GGGTGCCGTGAATACGAAACACATGCTGAACCAGGAGCTGGGTGGTTTCTAGAGCAGTGGGGAGTTGTGGAAGGGCAAGA	-23.4	-	Novel-mir-978-3p	-	TTCTAGAGCAGTGGGGAGTTGTGG
Novel-mir-766	CGGGAGCAGGGTCCTGTAGGGCTTTGGCGGTCCAGCCAAAGGGGCAGGATCTCATCGGCACACTGTAGACCTGGTACCCT	-33.2	-	Novel-mir-766-3p	-	TCTCATCGGCACACTGTAGAC
Novel-mir-906	CCGCCCCCCCATATACATCCAACAATGGCAGTGTCATCAGAGAACTTTTGTAGGTGATGGTGGGAGTTGTACTTGAAGTCTGATGTGTAGAGGGTGAACAG	-26.9	-	Novel-mir-906-3p	-	TGTACTTGAAGTCTGATGTGTAGA
Novel-mir-454-2	GTGGATGAAAGAGAAGCACCACATTTGGGAGTGGGTGTCACATCAGTCACACAGGTTAAAAAGCCGGTTATCAGGTTGGGAGCTGTCGAGCATGCAGCTGC	-25.9	-	Novel-mir-454-5p	-	TTATCAGGTTGGGAGCTGTCGAGC
Novel-mir-390-2	CTGCAGATATAATAATAAATCATCTGCATACAGGCAAACTTTATGTTGTAAGTTAGTATCTTGTATTCCTTTGATGCTATTATTCTGTCATATT	-18.4	-	Novel-mir-390-5p	-	TGTATTCCTTTGATGCTATTATT
Novel-mir-677	GCTCTTGCCTGAGCGGGACACACTAGCTTTGCAGCCGTGGATAAGCATTCTTTAATAAAGTCCCTTGTAGAAAATGAACGTCCACTCCTGGCAATTAGAGC	-27.3	-	Novel-mir-677-3p	-	TAGAAAATGAACGTCCACTCCTGG
Novel-mir-900	GAGAACTGTACAGCCAACCTGTAGGCCAGTAAGACGAGAGGAAGGTGCATATCCCAATCCCTCTGGTGTTCTGCAGTGAGGATGGCTAGTTGTTTT	-29.2	-	Novel-mir-900-3p	-	TGGTGTTCTGCAGTGAGGATGGC
Novel-mir-347-2	GGATGGTAAACGGCTCCAGGAACCGGGGAGACTATTTGGGGCATGCCCCTTTGATGGGAATGTGGGTGGCCGAGAGCCAAACCTGTT	-31.6	-	Novel-mir-347-5p	-	TGGGAATGTGGGTGGCCGAGAGC
Novel-mir-837-1	TAACACCAGACATACAGAAAATGGTGAAAGAAGGGAAATACCAGTTTTCCCAATGAGTCTTTCAAATGTTGTAGTTATCGATCC	-18.3	-	Novel-mir-837-3p	-	TGAGTCTTTCAAATGTTGTAG
Novel-mir-837-2	TAACACCAGACATACAGAAAATGGTGAAAGAAGGGAAATACCAGTTTTCCCACTGAGTCTTTCAAATGTTGTAGTTATCGATCC	-18.3	-	Novel-mir-837-3p	-	TGAGTCTTTCAAATGTTGTAG
Novel-mir-837-3	TAACACCAGACATACAGAAAATGGTGAAAGAAGGGAAATACCAGTTTTTCCCACTGAGTCTTTCAAATGTTGTAGTTATCGATCC	-18.6	-	Novel-mir-837-3p	-	TGAGTCTTTCAAATGTTGTAG
Novel-mir-712	GCAGGCAGAATGGGCTCAGGGGTATCTTCAGCAGTTGGGGGGTTATATTGTCGGGAGAAGGCATCTGGTTTCTGGTTG	-19.1	-	Novel-mir-712-3p	-	TATTGTCGGGAGAAGGCATCTGG
Novel-mir-710	ACTTTAACTGTACTGCTGTTAGCCACAAGGCTGATATTTCTTGCATGTATTCAAATATTATTCGGGTGCTGATAGAGGCTAGTTTGCCT	-20.8	-	Novel-mir-710-3p	-	TATTATTCGGGTGCTGATAGAGGC
Novel-mir-706	GCTATCATGCCCGCGTCTTCTCCAAGTCCCATAGATGGCCTCTGTCTATGGGACTTGGAGAAGACGCAGGAGGGTGG	-55.4	-	Novel-mir-706-3p	-	TATGGGACTTGGAGAAGACGC
Novel-mir-855	CCTTCCTCCTCGGAACAGTTCCTTTAGCACAAGACTGTTACATCCCCAGTGCAAGAAGGAGCGTTACTGTAGGTCGTTCC	-20.57	-	Novel-mir-855-3p	-	TGCAAGAAGGAGCGTTACTGT
Novel-mir-837-1	TAACACCAGACATACAGAAAATGGTGAAAGAAGGGAAATACCAGTTTTCCCACTGAGTCTTTCAAATGTTGTAGTTATCAATTC	-18.3	-	Novel-mir-837-3p	-	TGAGTCTTTCAAATGTTGTAG
Novel-mir-837-2	TAACACCAGACATACAGAAAATGGTGAAAGAAGGGAAATACCGATTTTCCCACTGAGTCTTTCAAATGTTGTAGTTATCGATCT	-18.3	-	Novel-mir-837-3p	-	TGAGTCTTTCAAATGTTGTAG
Novel-mir-837-3	TAACGCCAGACATACAGAAAATGGTGAAAGAAGGGAAATACCAGTTTTCCCACTGAGTCTTTCAAATGTTGTAGTTATCAATAC	-18.3	-	Novel-mir-837-3p	-	TGAGTCTTTCAAATGTTGTAG
Novel-mir-791	TCTGTGTCTAGGAGGAATGGCAGGTTGGGGGCTGGGGGGGTGAGAATAGGAGACTCCGTCAAAGCCTTCTTCAGCAATCTAAAGGCATGGTCGCATT	-28.7	-	Novel-mir-791-3p	-	TCTTCAGCAATCTAAAGGCA
Novel-mir-762-1	TGGGCCCAGTCGAGGGTCTGAGCCCTAGCAGCCAAGGGGACATATAATCTATCTGGTGGTCCACCCCCTGGATTGGGTTCC	-34.4	-	Novel-mir-762-3p	-	TCTATCTGGTGGTCCACCCCCTGG
Novel-mir-762-2	TGGGCCCAGTTGAGGGTCTGGGCCCTAGCAGCCTGGGGGACGTATAATCTATCTGGTGGTCCACCCCCTGGATCGGGTTCC	-34.2	-	Novel-mir-762-3p	-	TCTATCTGGTGGTCCACCCCCTGG
Novel-mir-762-3	TGGGTCCAGTCGAGGGTCTGAACCCTAGCAGCCAAGGGGACGTATAATCTATCTGGTGGTCCACCCCCTGGATCGGGTTCC	-27.6	-	Novel-mir-762-3p	-	TCTATCTGGTGGTCCACCCCCTGG
Novel-mir-710	CGGACTTTAACTGTACTGCTGTTAGCCACAAGGCTGATATTTCTTGCATGTATTCTAATATTATTCGGGTGCTGATAGAGGCTAGTTTGCCT	-22.8	-	Novel-mir-710-3p	-	TATTATTCGGGTGCTGATAGAGGC
Novel-mir-608	ACCTGCTCTCCTAAAAGAGCTGCGGTCCACAGCCCCAGCAGTGCCGCTGGCCTCGTGCTTGGATCTGGGTCTGGAGGAGGAGGAGG	-37.8	-	Novel-mir-608-3p	-	CGTGCTTGGATCTGGGTCTGGAG
Novel-mir-756	TCCATCTGTGCAAGAAGTTTCATTTCTGGAATAGGAATTGAGGAGAAAGCTTCATTCTTCCTCGGTCTGTGGAAGAGAGGTTTGTTGGTCGTGGC	-23.1	-	Novel-mir-756-3p	-	TCGGTCTGTGGAAGAGAGGTTTGT
Novel-mir-687	TTTGCGAGTGCTGGGATCTTTGTCTTTCCGCCTGCAGTATCCAAACACAGCCAGTCGATCACCATAGGACGGTAGATAATGGCTTAGCTGGTCATCTG	-24	-	Novel-mir-687-3p	-	TAGGACGGTAGATAATGGCTTAGC
Novel-mir-664	CGGTAGCCATAATGCTGGCCTGGTCCCTTCAGCTCTTTGTGGATGGCAGCACGTACAGTAGCAAGAGGAGAGTCATTTTTTTACC	-19.7	-	Novel-mir-664-3p	-	TACAGTAGCAAGAGGAGAGTCA
Novel-mir-1021-1	GCAGAAGGGGCAAAGAGAGAGGATGGGATACTGCTCATGTTTGTTTTGATTCTCACTTTGCCCTTAACGG	-25.2	-	Novel-mir-1021-3p	-	TTGTTTTGATTCTCACTTTG

Novel-mir-1021-2	GCAGGAGGGGCAAAGAGAAGCAGAGGGAGAAGAGGAGGGGATACTGCTTGGGTTTGTTTTGATTCTCACTTTGCCCTTAACGG	-29.5	-	Novel-mir-1021-3p	-	TTGTTTTGATTCTCACTTTG
Novel-mir-1021-3	GCAGGAGGGGCAAAGAGGAGCAGAGGGAGAAGAGGAGGGGATACTGTTTGAGTTTGTTTTGATTCTCACTTTGCCCTTAATGG	-29.5	-	Novel-mir-1021-3p	-	TTGTTTTGATTCTCACTTTG
Novel-mir-928-1	CGAAATGCAGCAGAGGAGGCAGCAGTTAATGGCTCCTGTGTTGTATGTGCTGGACCCCGTCCTGCCCTGTTAACTGTACCTTCTCTCCTCTCAGATC	-29.9	-	Novel-mir-928-3p	-	TGTTAACTGTACCTTCTCTC
Novel-mir-928-2	CGAAATGCGGCAGAGGAGGCATCAGTTAATACCTCCTGTGTTGTATGTGCTGGACCCCGTCCTGCCCTGTTAACTGTACCTTCTCTCCTCTCAGATC	-27.5	-	Novel-mir-928-3p	-	TGTTAACTGTACCTTCTCTC
Novel-mir-975	TCCCTCAAGGACCAGTTCACCACAGCCCTCGTACTCCTCCAACCTGACCCCTCACTGCAGTTCGTGGTAGAGGTGGACGCGTCCAACACTGGGG	-26.61	-	Novel-mir-975-3p	-	TTCGTGGTAGAGGTGGACGCGTCC
Novel-mir-851	AAGGTCCCACAGTTCGTGCTGAAATGCGATCGAAACCAGGTGTGTGTGTGTTGTCATGTGATTTTCCAGGCGGCAGCTGAACAATGGGTCCT	-29	-	Novel-mir-851-3p	-	TGATTTTCCAGGCGGCAGCTGAAC
Novel-mir-669	TACCAGTGCCAGGGCTCTGTTTCTGATCTCTTTGGTTTTCAAAGAGTATACCATAGGATTGATGCAGGGTGGGGTGCAAGAT	-26.4	-	Novel-mir-669-3p	-	TACCATAGGATTGATGCAGGGTGG
Novel-mir-660	GAGGGGGTAATGGATATTTGTCCTTAACTGTTATAGCATTAAGTCCGCGGTAATCAATGCAAGGACGGAGGCCGCCGTCCAT	-21.4	-	Novel-mir-660-3p	-	TAATCAATGCAAGGACGGAGGC
Novel-mir-1014-1	TGGCCTAGATCTGATTGCAGCCACATGACTGACACATGTAGGATGGCACACCTCTACATTGTCAAGTCTGGAGCTGTAACACATCAAAACAT	-31.9	-	Novel-mir-1014-3p	-	TTGTCAAGTCTGGAGCTGTAACAC
Novel-mir-1014-2	TGGCCTAGATCTGATTGCAGCCACATGACTGACACATGTAGGATGGCACACCTCTACATTGTCAAGTCTGGAGCTGTAACACATCAAAACCT	-32.2	-	Novel-mir-1014-3p	-	TTGTCAAGTCTGGAGCTGTAACAC
Novel-mir-928	AAAATGCAGCAGAGGAAGTGTCAGTTAACACCTCCTGTGTTGTATGTGCTGGACCTCGTGATGCTCTGTTAACTGTACCTTCTCTCCTCTCAGATC	-23.5	-	Novel-mir-928-3p	-	TGTTAACTGTACCTTCTCTC
Novel-mir-792	ACTTGGGGTGTTTTGCTTTGGACAGCAACTGTTCTTTCATTTCAATTTGGCAGCGATCAATATGTTCTTGAATCTCACTGGCTGTCCACTTATCTGGGG	-21.4	-	Novel-mir-792-3p	-	TCTTGAATCTCACTGGCTGTCCAC
Novel-mir-669	TACCAGTGCCAGGGCTCTGTTTCTGATTTCTTTGGTTTTCAAAGAGTATACCATAGGATTGATGCAGGGTGGGGTGCAAGAG	-25	-	Novel-mir-669-3p	-	TACCATAGGATTGATGCAGGGTGG
Novel-mir-1017	CATACCTGTGGGTAAGGAGAGAGGACATGTTAACCAGGCCATAGCTTGTTTAATTCTGAGCCCGTGCCTGCACTTAAT	-21.9	-	Novel-mir-1017-3p	-	TTGTTTAATTCTGAGCCCGTGCC
Novel-mir-997	ATTTTTTTTTTGGCTAGAGCAGGTAAATTAGGTGTCATGCTCCCTAACAGGTGAATGTGTGGTGTGTTTGATTTGGGCTCTGTCAGGGTGAGGTAGTA	-25.3	-	Novel-mir-997-3p	-	TTGATTTGGGCTCTGTCAGGG
Novel-mir-727-1	AGGGCATAAATAGTTGAGCAAAAGCATTTTACTTATGTGTGCGCTCAAAAAACATCCCGTGCTCAGAATGTCAGATGCGCGCGCTCAAAATGTCCC	-27.54	-	Novel-mir-727-3p	-	TCAGAATGTCAGATGCGCGCGCTC
Novel-mir-727-2	ATAAATAGTTGAGCAAAAGCATTTGACCTATGCGTGCGCTCAAAAAAAACATCCCGCGCTCAGAATGTCAGATGCGCGCGCTCAAAAAAATAT	-28.59	-	Novel-mir-727-3p	-	TCAGAATGTCAGATGCGCGCGCTC
Novel-mir-706	CCACCCTCCTGCGTCTTCTCCAAGTCCCATAGACAGAGGCCATCTATGGGACTTGGAGAAGACGCGGGCATGATA	-49.9	-	Novel-mir-706-3p	-	TATGGGACTTGGAGAAGACGC
Novel-mir-579-1	AGGCAGTGTTCCTCTCTCTCAGTAGCCACTTGACCTCCCATTTCATCCAGGGCTTCTGGTTGGGGTAGACCCTGATT	-20.4	-	Novel-mir-579-3p	-	CAGGGCTTCTGGTTGGGGTA
Novel-mir-579-2	CTGAAGGCAGTGTTCCTCTCTTTCAGTAGCTGCTTGGCCTCCCAGTTCATCCAGGGCTTCTGGTTGGGGTACACCCTGATG	-22.22	-	Novel-mir-579-3p	-	CAGGGCTTCTGGTTGGGGTA
Novel-mir-579-3	TCTGAAGGCAGTCTTCCTCTAATTCAGTAGCCACTTGGCCTCTCATTTCATCCAGGGCTTCTGGTTGGGGTAGACCCTCATT	-24.02	-	Novel-mir-579-3p	-	CAGGGCTTCTGGTTGGGGTA
Novel-mir-579-4	TGTCAGTGTTACTCTCTCTCAGTAGTCGCTTGACCTCCCAGTTCATCCAGGGCTTCTGGTTGGGGTAGACCCTGATG	-21.3	-	Novel-mir-579-3p	-	CAGGGCTTCTGGTTGGGGTA
Novel-mir-981	GGAGACCTTGAAGCCTCCCTGTGCCAAATGTGTCAACAATGTGTCTGTAGCAATCATTATGCTTTCTGAGTCAGGAGCAGCTAACAACAGGTCATC	-18.5	-	Novel-mir-981-3p	-	TTCTGAGTCAGGAGCAGCTAACA
Novel-mir-980-1	ACCCAGCTCACTGTGCCTGTCCCCACCTGTCAGTGGATTACTAGCTTTCTGACAGACAAGGAGCAGCAGGTGAGGCTGGG	-35.9	-	Novel-mir-980-3p	-	TTCTGACAGACAAGGAGCAGCAGG
Novel-mir-980-2	CCCAGCTCACTGTGCCTGCCCCCACCTGTCAGTGGATTACTAGCTTTCTGACAGACAAGGAGCAGCAGGTGAGGCTGTG	-31	-	Novel-mir-980-3p	-	TTCTGACAGACAAGGAGCAGCAGG
Novel-mir-893	TCAAGCTGTTTGTAGCAGCCTTCCACATCCCCATTTTTCGTTTATAATATATGAAAAATGGGGATGTGGAAGTCTGCTACAAACAGCTTG	-66.6	-	Novel-mir-893-3p	-	TGGGGATGTGGAAGTCTGCTAC
Novel-mir-591	TGCCTCCTTGAGAGAAGACAAGGAGCTAGGGGAAAGCTGTTATCTGCATCCCACCTACATCGCAGGACCAGAAGAGAAGGCAGGCCCCAGAGGAATGGCTA	-26.7	-	Novel-mir-591-3p	-	CCAGAAGAGAAGGCAGGCCCCAGA
Novel-mir-1037	GTAGGGATGTTGGGGTCGGCCAGTCCTGCACCTTCTGGACTTTGTTGGGATCTGTAGCCACGCCACACTCCGAC	-24.6	-	Novel-mir-1037-3p	-	TTTGTTGGGATCTGTAGCCACGCC

Novel-mir-1021	GACAAAGGGGCAAAGAGGAGCAGAGGAAGAAGAGGAGGGAGAAAAGGAGGGGATACGATTGAGTTTGTTTTGATTCTCACTTTGCCCATAAACG	-20.7	-	Novel-mir-1021-3p	-	TTGTTTTGATTCTCACTTTG
Novel-mir-1001-1	CATTACACGTCCGGATCTCTCCCACCATCCACCTGCGCGCAGGCAGCTTGGAAAGATGAACAGAGACCGGACCCAGAAAC	-26.3	-	Novel-mir-1001-3p	-	TTGGAAAGATGAACAGAGACCGG
Novel-mir-1001-2	CATTACACGTTCGGATCTCTCCCACCATCCACCTGCGCGCAGGCAGCTTGGAAAGATGAACAGAGACCGGACCCAGAAAC	-23.4	-	Novel-mir-1001-3p	-	TTGGAAAGATGAACAGAGACCGG
Novel-mir-941-1	GGTGAAATAAACCTGCTGAAGCGTGTCCTCTGTGTACCCTCTATCCCAGGTTGGTAAACACTGAATGTTAAGAGTTAGATGCAAACGGTAGGAGTTGCATG	-22.1	-	Novel-mir-941-3p	-	TTAAGAGTTAGATGCAAACGGTAG
Novel-mir-941-2	TGTGAAATAAACCTGCTGAAGCGTGTCCTCTGTGTACCCTCTATCCCAGGTTGGTAAACACTGAATGTTAAGAGTTAGATGCAAACGGTAGGAGTTGCATG	-22.4	-	Novel-mir-941-3p	-	TTAAGAGTTAGATGCAAACGGTAG
Novel-mir-950	GTGTCGACATAGCACGTTAGCTCTGGGTAAGAGTCCAATGCGCTGAAAAGACTACGTTCTCTTAGCTGTAGTAACGTCGCCCGGTCGTAAA	-24.6	-	Novel-mir-950-3p	-	TTAGCTGTAGTAACGTCGCC
Novel-mir-941	TGAAATAAACCTGCTGAAGCGTGTCCTCTGTGTACCCTCTATCCCAGGTTGGTAAACACTGAAGGTTAAGAGTTAGATGCAAACGGTAGGAGTTGTATG	-21.9	-	Novel-mir-941-3p	-	TTAAGAGTTAGATGCAAACGGTAG
Novel-mir-876	GGGCACTCTACTTAAATATTTTCATGGTACTCACTATGTCCCTTCACAGATTGCTAGAAAATTTCTGATGTGGAGAGAATTGCCA	-20.4	-	Novel-mir-876-3p	-	TGCTAGAAAATTTCTGATGTGGAG
Novel-mir-827	GATGTGGAAGCTGATGTCCTCATGATGGTTTCCAGCGATGGTGAGATGAAGTGGTGGCGTCCGGTGGGTGATGT	-18.4	-	Novel-mir-827-3p	-	TGAGATGAAGTGGTGGCGTCCGG
Novel-mir-818	TCAAGCCGAGAGAGGGATCTCCCCTGAAACACTGCGTAGAGGGTTGTATCGTTGCCGAAAAGACACGTGACTGATGACGTCCGACACAGAAATGGCTGGCC	-25.2	-	Novel-mir-818-3p	-	TGACTGATGACGTCCGACACAGAA
Novel-mir-777	TGAACAGTGTTCAGCGTCCTCACAGCCTGGTGGATGAAGCTGTCTGTAAGTCTGGTGGAGTGGGCCTGAAGA	-20.5	-	Novel-mir-777-3p	-	TCTGTAAGTCTGGTGGAGTG
Novel-mir-730	GACAAGAAGACTCTTTTGGTTCTTCCATGCTGGACACCTTTTCCCTCAGGGGTGCAACCTCAGTCTGGAGATCAGAGCTCAAACTCTTGAG	-24.52	-	Novel-mir-730-3p	-	TCAGTCTGGAGATCAGAGCTCA
Novel-mir-721	ATGTTGACTGTTAGTCTCTCCAAAGTTTTGTGTGTTCATGTTACACTCTTCACACGGAACTGATGGAGCCCTTTAGTCACTTG	-26.3	-	Novel-mir-721-3p	-	TCACACGGAACTGATGGAGCCCTT
Novel-mir-600	CCTCATCTTCTGTCTCTGAATTGAACAGCTGGGCAATTTCGCCATCAAACATGCCGGGTTCCCTCTCATCATTGTCGGAGTCAGTCACGTTGC	-21.2	-	Novel-mir-600-3p	-	CCCTCTCATCATTGTCGGAGTCA
Novel-mir-545	CAGCCCCCCGAGTTGGTGTTCATGTTAGATCAGCTGTCTAACACCCGGGTGACGGCCAAGCAGATTAAGAGGTGGACTGAGAGGGATCCAG	-21.6	-	Novel-mir-545-3p	-	AGCAGATTAAGAGGTGGACTGAG
Novel-mir-533	ATGTGCAGCTCAAGCATGCAGTGTGTGAGTGTTGGACCTGAGAGGAGAGAAGGTACAGTTAACAGGGCAGGACGGGAGGGGTGCCA	-21.2	-	Novel-mir-533-3p	-	ACAGTTAACAGGGCAGGACGGG
Novel-mir-948	AGCAGTCAATGGAGCGATGATGGCTTGGAGATGAGGATGGCTGAGGAGGGAATATCAGAGAGACAGATTACTGGAGACTGTGCATCGACCAGTGAAGGCG	-25.4	-	Novel-mir-948-3p	-	TTACTGGAGACTGTGCATCGACC
Novel-mir-466	AATTCTGGGCTCAGACTTGGCCGCTCCAAAAGGCCCATTTTGTTTTTCTGATGCCATTCTGTTGTGGACTTGCTCTGGTGTTTTGGGT	-18.47	-	Novel-mir-466-5p	-	TTCTGTTGTGGACTTGCTCTGG
Novel-mir-863	AGCTCTGGGTTCTGTTTCTATCTGAAGCCCTATCACACAGAGAAGTCAGTGCATAGCTGCTGCAGGAAGGCGGAACCACGAGT	-27.7	-	Novel-mir-863-3p	-	TGCATAGCTGCTGCAGGAAGGCGG
Novel-mir-846	TGACCGGGTGTCGATCAGCGGCAGCAGAAACACAGTCAATGGTATGATGGGTGATGATGGGCCGGGCTGCGGACGTTGGGATCA	-24.8	-	Novel-mir-846-3p	-	TGATGATGGGCCGGGCTGCGGAC
Novel-mir-714-1	AGCGTGTAGACTGGTCAGATGCGCAGTGACAGTTATTTTCAGGGCATTTCTGTATTTAGCTCTCCTGCTGCGTGGCAAGTTGCGAA	-25	-	Novel-mir-714-3p	-	TATTTAGCTCTCCTGCTGCGTGGC
Novel-mir-714-2	ATGTAGACTGGTCAGATGCGCAGTGACAGTTATTTTCAGGGCATTCTTGGCCGCTGTATTTAGCTCTCCTGCTGCGTGGCAAGTGTTGGG	-28.1	-	Novel-mir-714-3p	-	TATTTAGCTCTCCTGCTGCGTGGC
Novel-mir-714-3	GTGTGTAGACTGGTCAGATGCACAGTGACAGTTATTTTCAGGGCATTTTTGGCCGCTGTATTTAGCTCTCCTGCTGCGTGGCAAGTGAAACA	-27	-	Novel-mir-714-3p	-	TATTTAGCTCTCCTGCTGCGTGGC
Novel-mir-579	CTGAAGGTAGTGTTCCTCTCTTTCAGTAGCCACTTGACCTCCCAGTTCACCCAGGGCTTCTGGTTGGGGTAGACCCTTATG	-20.5	-	Novel-mir-579-3p	-	CAGGGCTTCTGGTTGGGGTA
Novel-mir-546	TTAATTCATTATTAAAATCAGAATCAGCTTTATTGGCCAGGTGTGTTGGGCACATGAGGAATTTGACTCTGGTACATGATAGAATAAT	-20.8	-	Novel-mir-546-3p	-	AGGAATTTGACTCTGGTACATG
Novel-mir-527	TGGCTGCATCACTATGTGGTACGGAGCCTGTACTGCCTCCTGCAGAAGGACTCTGCAGCACATAGTTAATACAGCC	-33.8	-	Novel-mir-527-3p	-	AAGGACTCTGCAGCACATAGT
Novel-mir-1021-1	ATACACAGGGCAGGAAGAGGAACGGAGGGAGAAGAGGAGGGGATACTGCATGAGTTTGTTTTGATTCTCACTTTGCCTTTACGAA	-20.5	-	Novel-mir-1021-3p	-	TTGTTTTGATTCTCACTTTG
Novel-mir-1021-2	GCATGAGGGGCAAGGAGGAGCGGAGGGAGAAGAGGAGGGGATACTGCTCGACTTTGTTTTGATTCTCACTTTGCCTTTAATGG	-29.3	-	Novel-mir-1021-3p	-	TTGTTTTGATTCTCACTTTG

Novel-mir-1016	TCTCTCCTGGCAGCCAGGGAGACATAATTTATCTGGTGGTCCACCCCCTGGATCGGGTTCCTGTTGTTGTGCCTCCCGTATGGTGTCCTCGATCTT	-28.3	-	Novel-mir-1016-3p	-	TTGTTGTGCCTCCCGTATGGTGT
Novel-mir-938	ATAGCTAGCTTTATTTACCGGTTAAAAAGACAAATTCTTGTGTTGGGCCATTGCTTGAATGTTTTGAGGACATTAAATCTTGGCTTGCACT	-18.2	-	Novel-mir-938-3p	-	TGTTTTGAGGACATTAAATCTT
Novel-mir-888	GCCCCGTGCACACTGATGTGTTTGTAGTGGTTTAAGTTACATGTTAATATGGATTATGTAATAGAATAGTGTTTATGGGTT	-22.2	-	Novel-mir-888-3p	-	TGGATTATGTAATAGAATAGTG
Novel-mir-774	AATTCTGGGACCCAGTGTCAGAAAGTTGGGTCTCCGTCAGAGGTCCTGGGAGAAGGAAGCCTTCAAATCTGAGAGACCTGGAGGAGTTGGCAAAAGAAAAG	-33	-	Novel-mir-774-3p	-	TCTGAGAGACCTGGAGGAGTTGGC
Novel-mir-717	ATCATCTCTTGGCATTCACCGCGTGCCTTAATTGTAAGACATTAAATCAAGGTCCGCTGTGAACACGGAGAGACACA	-26.3	-	Novel-mir-717-3p	-	TCAAGGTCCGCTGTGAACACG
Novel-mir-714-1	ATGTAGACTGGTCAGATGCGCAGTGATAGTTATTTTAAGGGCATTTTTGGACGCTGTATTTAGCTCTCCTGCTGCGTGGCAAGTGTTGGG	-21.2	-	Novel-mir-714-3p	-	TATTTAGCTCTCCTGCTGCGTGGC
Novel-mir-714-2	ATGTAGACTGGTCAGATGCGCAGTGATAGTTATTTTCAGGGCATTTTTGGCCACTATATTTAGCTCTCCTGCTGCGTGGCAAGTGTTGGG	-25.1	-	Novel-mir-714-3p	-	TATTTAGCTCTCCTGCTGCGTGGC
Novel-mir-666-1	AGTAAGGTTAATAGGTTACAGCTCCAGACTTGACAATGTAGAGATGAGCCATCCTACATGTGTCAGACATGAGGCTGCCATTAGATGC	-26.7	-	Novel-mir-666-3p	-	TACATGTGTCAGACATGAGGCTGC
Novel-mir-666-2	GGTTTTAATGTGTTAAAGCTCCAGACTTGACAATGTAAAGGTGAGCCATCCTACATGTGTCAGACATGAGGCTGCAATTAGATGC	-19.3	-	Novel-mir-666-3p	-	TACATGTGTCAGACATGAGGCTGC
Novel-mir-666-3	GGTTTTAATGTGTTACAGCTCCAGACATGACAATGTAGAGGCGAGCCATCCTACATGTGTCAGACATGAGGCTGCAATTAGATGC	-23.5	-	Novel-mir-666-3p	-	TACATGTGTCAGACATGAGGCTGC
Novel-mir-666-4	TAATGTGTTACAGCTCCAGACTTGACAATGTAGAGGTGAGCCATCCTACATGTGTCAGACATGAGGCTGCAATCAGATGC	-25.4	-	Novel-mir-666-3p	-	TACATGTGTCAGACATGAGGCTGC
Novel-mir-1012	TCGTCACAGTAACAGCTGGGGTAATCATGTGATAACAGCTCAGCGTAGGATGGAGTTGTATCGAAAGAAGCCATAGCTGTCAGATG	-25	-	Novel-mir-1012-3p	-	TTGTATCGAAAGAAGCCATAG
Novel-mir-863	AGCTCTGGATTCCGTCTCTATCTGAAGCACTATTACAAGTATAGTGCATAGCTGCTGCAGGAAGGCGGAACCACGAGT	-31.3	-	Novel-mir-863-3p	-	TGCATAGCTGCTGCAGGAAGGCGG
Novel-mir-859	AGCTCAGGAGACCGCTTTCCAGCAGGTCAAGGGACTACTGCACTCTGACCGAGTGCTAGTGCACTTTGATCCAGAACGGGAGGTGGTACTGGC	-41.7	-	Novel-mir-859-3p	-	TGCACTTTGATCCAGAACGGGAGG
Novel-mir-752	TGGGTGCTGGTATTGGAGACCCCCAGCTGAGGAAGAGCCCGGGCCTCGACTGGTGGTTCTGCTGTGGCACGGGGCCGG	-31	-	Novel-mir-752-3p	-	TCGACTGGTGGTTCTGCTGTGGC
Novel-mir-575	TAGGCTTAATCACAGGTACGCAGTTTTTCCATAGGGTCTGGGCATGGATTCCAAGCAAAGTCACTGGTGGACTTACAAGTAGTGATGGCCAT	-26.7	-	Novel-mir-575-3p	-	CACTGGTGGACTTACAAGTAG
Novel-mir-567	CTGGAGAGCCTGTAGGGGGCCGGTGCATTGTGTTTTGGGCAGTGGCCAGGGCCAAGTGCTAGGCTGACCTGAACCTCGGATG	-29	-	Novel-mir-567-3p	-	CAAGTGCTAGGCTGACCTGA
Novel-mir-1008	TCCTTGGTCAAGACACTGAACCCCCAAGTTTCCCCCGATGGTCAGAGCCATTGGTTGAGTTGGTGCTGCAGATGTCTGCCTTGG	-30.4	-	Novel-mir-1008-3p	-	TTGGTTGAGTTGGTGCTGCAGATG
Novel-mir-1003	CAGGTAAAATTAACATTACACGTCCAGATCTCTCCCACCACCAACCTGCGCGCAGGCAGCTTGGAAGATGCAAGACCCTGGACCATGAAACCTA	-22.5	-	Novel-mir-1003-3p	-	TTGGAAGATGCAAGACCCTGGACC
Novel-mir-990	CAGAGGCAACTTGGGGGTTCAGTGTCTTGCCCAAGGACAATTTGACATGTGGACTGCCGGCACTGCAATTC	-20.3	-	Novel-mir-990-3p	-	TTGACATGTGGACTGCCGGC
Novel-mir-986	GCCTTGCGGTCTTCGATTGGTTTGTCCCTGAACAACTTACATTTTTTGAGTGGGTGTGGTTTCTTATGGATTGGGCATTCACGGTCCGGTTCTT	-20.7	-	Novel-mir-986-3p	-	TTCTTATGGATTGGGCATTCACGG
Novel-mir-942	GTTCTTCTGCAGCAAAGTGAATTTAGTGTCTAGGGTGCCACACGACGGAGCCTTAAGGATTATGGAGGCCATTGTGCTCTTAGGAA	-20.8	-	Novel-mir-942-3p	-	TTAAGGATTATGGAGGCCATTGTG
Novel-mir-934	ATTATCCATGTGCAAACTGAGAATGTAAACAGTACATGATGTATTTCCTGGTAATAAACTCCTCAGCTGTTTAGAGTCTGATGGCAGAGGGAAAGAAAGA	-19.1	-	Novel-mir-934-3p	-	TGTTTAGAGTCTGATGGCAGAGG
Novel-mir-419	GCCTATGTATTAAGGCCTATTATGGTCTGAACCAGGACAAGTTACTGTGTTTGACTGTAAGGATGGTATTAATAAGG	-20.8	-	Novel-mir-419-5p	-	TGTGTTTGACTGTAAGGATGGT
Novel-mir-894	AGCACGTGGAGGCCACAGCTGGCAGGCTGGACGGGACAGACGAGGTGACTGCAGCATGGAACCAGGTTGGGGCTGAAGCTGCAGCGATGGCTTGGACTGCT	-36.2	-	Novel-mir-894-3p	-	TGGGGCTGAAGCTGCAGCGATGGC
Novel-mir-338	AGGTAGTTGAGAAGCTTCTCGCTGGCAAGGCAGATGAGATCTGCCCTGAGTACCTTAAGTCTCTGGATGTTGTGGGGCTGGCTTGGCTGACACACCG	-30.6	-	Novel-mir-338-5p	-	TGGATGTTGTGGGGCTGGCTTGGC
Novel-mir-882-1	AGGATCACAGGTAAAATTAACATTACACGCTCAGGTATCTTGCACCGCCAAACTGCTCGCAGGCAGATGGAAGATGAAAGACCCTGAACCCACCAGGCCTA	-21.5	-	Novel-mir-882-3p	-	TGGAAGATGAAAGACCCTGAACCC
Novel-mir-882-2	CAGGTAAAAATTAACATTACACGTTCAGGTATCTTGCACCTCCAAACTGCTCGCAGGCAGCTGGAAGATGAAAGACCCTGAACCCACCAGGCCTA	-24.9	-	Novel-mir-882-3p	-	TGGAAGATGAAAGACCCTGAACCC

Novel-mir-882-3	CAGGTAAAATTAACATTACACGCTCAGGTATCTTGCACCTCCAAACTGCTCGCAGGCAGCTGGAAGATGAAAGACCCTGAACCCACCAGGCCTA	-21.1	-	Novel-mir-882-3p	-	TGGAAGATGAAAGACCCTGAACCC
Novel-mir-880	GACATGTTGTGGGTGAAGAGGAAACAATTGTAAGCTAATTCTCAGTTCTTTTAACCAGTGGAACTCAGAGAGACGAAGCGGACCACTTGGA	-19	-	Novel-mir-880-3p	-	TGGAACTCAGAGAGACGAAGCGG
Novel-mir-865	TTGCTTCTCCGTGAGGGCAGCCTTGCCCTGTATTTCTCTTATAGTCTGCAGACTAAATTCTGTTGCATGTACATGACCGCTCTCAGCGGATTTTTGA	-24.23	-	Novel-mir-865-3p	-	TGCATGTACATGACCGCTCTCAGC
Novel-mir-844-1	ACCTCTTCCCCGTGGTCTGCCACGGTACTGAAGTCCACAGTACATGGGAGCAGAATCCCACTGATAAGGTGGTGCGGTCGGCTGTTGAACAAAGG	-31.5	-	Novel-mir-844-3p	-	TGATAAGGTGGTGCGGTCGGCTGT
Novel-mir-844-2	ACCTCTTCCTCATGGCCTGCCACGGTACATGGGAGCAGAATCCTACTGATAAGGTGGTGCGGTCGGCTGTTGAACAAAGG	-23.1	-	Novel-mir-844-3p	-	TGATAAGGTGGTGCGGTCGGCTGT
Novel-mir-812	TGCTGGAAAGTGCTTCGACTGGGGACTCCATTGTTCTACTGGGGTACTTCAATACTCACATGGGCGATGACAGTGATACCTGGAGGGGCGTGATTGGCAGG	-30.1	-	Novel-mir-812-3p	-	TGACAGTGATACCTGGAGGGGCGT
Novel-mir-143-1	ATGATAATTAAAAGGTGGGGCGACTGTTCATTTTCTGTTTGTGGCCCTAGACTATGGAATGCCCTGCCGGTTGAACTACGG	-23.2	-	Novel-mir-143-5p	-	TAGACTATGGAATGCCCTGCCGGT
Novel-mir-143-2	TAGTGGTCCCGCGATCTCGCTATAAAAAGTGGGGTGACTGTTCGTTTTCTATCTGTGGCCCTAGACTATGGAATGCCCTGCCGGTTGTACTACGG	-22.6	-	Novel-mir-143-5p	-	TAGACTATGGAATGCCCTGCCGGT
Novel-mir-619	CCAGTGAATAACAAGGACGTTTTCTGATCTCAGCAGGTTCCTTTTGTTCTGCTTTTGAAGGATGTGTTTGTTCTGCTCGGTGC	-19.4	-	Novel-mir-619-3p	-	CTTTTGAAGGATGTGTTTGTTC
Novel-mir-594	ATACCGGGGGTCCTAACACGGTGGAGAAGAGATGCTCACTTTATTGTGCTCCCATCAGTCACATTGGTTTGTGTCCTCGGCTG	-25.6	-	Novel-mir-594-3p	-	CCCATCAGTCACATTGGTTTGTG
Novel-mir-562	CAGGGTGCACTCTAGCAGCACATCATGTTTTGCAGATATGTACTGAATCACTCCAAAACATTATGCGCTGCTACTGTGAGCTCAA	-32.1	-	Novel-mir-562-3p	-	CAAAACATTATGCGCTGCTACT
Novel-mir-556	GTGTGTGTGTGTGTGTGTCTGTGTCGTGTAGCGCCGATGTAGTGTGAAATGGAGTCAAGCCAAGACGCACAGTCACTGTCAG	-22.6	-	Novel-mir-556-3p	-	ATGGAGTCAAGCCAAGACGCACAG
Novel-mir-1023	TGGAAAAATATGGACTGTATTTTTTATATAGCGCTATTCTCACAGTGTGTTGCACTGGCCAAAGCGCTTTACAGAGGATCATACACCATTCACCCAT	-24.7	-	Novel-mir-1023-3p	-	TTTACAGAGGATCATACACC
Novel-mir-984	TGATCATAGACTTTCCACCATCCTGCAGAGAAAAACTCTTCTGTTGGTTTGAGGAAAGGGCTGTATGGTGG	-19.5	-	Novel-mir-984-3p	-	TTCTGTTGGTTTGAGGAAAGGGC
Novel-mir-970	AAAGGGATAACGTGGTTGGCCTCCGTTGGGACTGACAACACACCGCCATGAACGGCGCGCAATTCCAATGCACTGACCCGCTTGATCGTATC	-23.5	-	Novel-mir-970-3p	-	TTCCAATGCACTGACCCGCT
Novel-mir-951	GCCTAAACACTACAGCAGCTGCTTTTACTGATCTAAACACTTTGGGGACTTGTTAGTGAAATCAGCTGCTGTGCTGTTTGTCT	-33.22	-	Novel-mir-951-3p	-	TTAGTGAAATCAGCTGCTGTG
Novel-mir-937	CATCGGCTGTTGTTGCTGCGCTGAGGCCACACTGTGTGAGTCATGGAGGTGTTTGTCGAGGCGGGCAAGGGCTTTCTTCT	-24.8	-	Novel-mir-937-3p	-	TGTTTGTCGAGGCGGGCAAGG
Novel-mir-917-1	AACCTAAGGCAGGGATGAATCCAGCCGCCCCATGAGCAAGATGTTGGGTGTGATTGGATCCGGGTCTGCAGCATCTGAGGAG	-27.1	-	Novel-mir-917-3p	-	TGTGATTGGATCCGGGTCTGCAGC
Novel-mir-917-2	ACTCTGGGTACACAACCTGAGGCAGGGATGAATCCAGCCGCTCCCTGAGCCAGATGTTGGGTGTGATTGGATCCGGGTCTGCAGCATTTGAGGAG	-30.42	-	Novel-mir-917-3p	-	TGTGATTGGATCCGGGTCTGCAGC
Novel-mir-914	TGACAGTGTGGTTGGTACTGTAGGTTGGCACACGGTCGTGGGTCAGCAGTGTGAAGAGCAATGGACTGAGCACATGCCCC	-23.8	-	Novel-mir-914-3p	-	TGTGAAGAGCAATGGACTGAG
Novel-mir-908	GCCTGACACCCTGTCTGGTCCTGCGGCTTTGTTGGTGTTGATCCTTCTCAGGGTGGAGCTCACCTGCTGTAGCTGCAGGACGAGAGGCTGGTGCTGTTC	-44.1	-	Novel-mir-908-3p	-	TGTAGCTGCAGGACGAGAGGCT
Novel-mir-313	GACGTGAGATCTCACTGCGTACGTCAGCAGGTGCGTAACATGTGGGTTTGTCGGTGTAGTAGGTGTGTGCGTAGTTGCTGTTGGTATGATCATTGCTGCTG	-27.46	-	Novel-mir-313-5p	-	TGCGTAGTTGCTGTTGGTATGATC
Novel-mir-871	TACTTGTTGTGTGAGCGTCGACAGGTCATGTAGCTGCTGCTGCAGTGCCTGAGCCAGTTGAGCGTTTGACGGTGAACT	-26.6	-	Novel-mir-871-3p	-	TGCCTGAGCCAGTTGAGCGTTTG
Novel-mir-797	TCAGAGGGAGGAATACCAGGAGAGCATGTGGGATGAAACACAGGACAGTCTCTTTCATCTTCTTCACTGCTCATTCTCGCTGC	-22.9	-	Novel-mir-797-3p	-	TCTTTCATCTTCTTCACTGCTCA
Novel-mir-793	GATGCACACAGATGGATAAAACATTAAAATACATACAAAATGCACACTGTTGAGAGCCCTCAACACCTCTTGCATTATTTGTCCTGTAAAATGCGTTCCC	-22.9	-	Novel-mir-793-3p	-	TCTTGCATTATTTGTCCTGTAAA
Novel-mir-713	ACTTCAAGTACAACTCCCAACAGTGTCATCTACAAAAGTTCTCAGACGACACTGCTATTGTTGGATGTGCATCTGGGGGGGATGAGGA	-24.5	-	Novel-mir-713-3p	-	TATTGTTGGATGTGCATCTGGGG
Novel-mir-162	GCCAGGGCTGGGGAATCGGGAGGGGATTCGTCCATTACCCTGGCTGAAGTTGCCAAGGTAGTTAAGGAACTCCTCGGTGGCACGGCTCTGGG	-43.2	-	Novel-mir-162-5p	-	TAGTTAAGGAACTCCTCGGTGGCA
Novel-mir-683-1	CTGACCTAAAGTGCTCTGCCAGTACTGCATCAGACTGCAGGTCAATGACTTCTAGCTGAACATCACTGGGTGCATTATCTACACT	-21.3	-	Novel-mir-683-3p	-	TAGCTGAACATCACTGGGTGCAT

Novel-mir-683-2	CTGACTTAAAGTGCTCTGCCAGTATTGCATCAGACTGCAGGTCAATGACTTCTAGCTGAACATCACTGGGTGCATTATCCACACT	-22.1	-	Novel-mir-683-3p	-	TAGCTGAACATCACTGGGTGCAT
Novel-mir-596-1	AAACTTAGGGGTGACAAGGCTTTTGCTGTGGGGGCTCCAAAGTTGTGGATTTCATTGCCCCTATATATTAGGCAGGCCCCCTTATTGT	-30.31	-	Novel-mir-596-3p	-	CCCCTATATATTAGGCAGGCC
Novel-mir-596-2	AAGACTTGGGGGGACAGGGCTTTTGCTGTGGGGGCTCCAAAGTTGTGGAATACATTGCCCCTATATATTAGGCAGGCCCCTTCGTTGT	-32.51	-	Novel-mir-596-3p	-	CCCCTATATATTAGGCAGGCC
Novel-mir-587	GTAAAGGACCTAAGCAAGCTACTTGTTTCTATCGCTGCTGGCGATGGCAGACAGCGGCTGGGTCATGTGAAGGTCAGCAGCGGAGGAGGTTCATGGA	-35.6	-	Novel-mir-587-3p	-	CATGTGAAGGTCAGCAGCGGAGGA
Novel-mir-578-1	TTAACTACAATGTGTATTCACTGTGATGTGCCTTGGGAGTGATGCTCAGCACTGCTTCCCATGCAGGGAGTTACAGGAAAAGGCAAAATGACTGA	-27.3	-	Novel-mir-578-3p	-	CAGGGAGTTACAGGAAAAGGCA
Novel-mir-578-2	TTAACTACAATGTGTATTCACTGTGATGTGCCTTGGGAGTGATGCTCAGCACTGCTTCCCATGCAGGGAGTTACAGGAAAAGGCAAAATGATTGA	-27.3	-	Novel-mir-578-3p	-	CAGGGAGTTACAGGAAAAGGCA
Novel-mir-578-3	TTAATTAACTACAATGTGTATTCACTGTGATGTGCCTTGGGAGTGATGCTCAGCACTGCTTCCCATGCAGGGAGTTACAGGAAAAGGCAGAATGATTGA	-28	-	Novel-mir-578-3p	-	CAGGGAGTTACAGGAAAAGGCA
Novel-mir-576	ACTGTCTGCTGAAGTCTGTACTTGTCCTGCTTTGGTTGCTGAGCCAAACCAGACAGTGATAGAGGTGCAGAGAACAGACTCG	-28.3	-	Novel-mir-576-3p	-	CAGACAGTGATAGAGGTGCAGAG
Novel-mir-568	GCTGGAGCTGTGGTGCATTGTAGTTGCATTGCATGTTATGCTAACACACTGCAATGGATCTGCTTTGCAACACAGAACTTGG	-30.7	-	Novel-mir-568-3p	-	CAATGGATCTGCTTTGCAACA
Novel-mir-535	CAGTCTACTTCGTCTTATTGTGAGTTCCTGGTTTAAAAGAACCGGAAGAAGCTACAGAGAAATAGTAGCT	-19.6	-	Novel-mir-535-3p	-	ACCGGAAGAAGCTACAGAGA
Novel-mir-1036	GCTTGATACGCCCGTCCTCGTCCCCGGCTGGGGCAGGGTTCTTTTTTGTTGGGAAAAAGGATGGGGGTCTTAGAC	-29.1	-	Novel-mir-1036-3p	-	TTTGTTGGGAAAAAGGATGGG
Novel-mir-1025	AGGATGCTTCGTCCCCACCAAAGATGCGACCTATCACTGCGGTGTCTTCTGCGAACTTTAGGATGATGTTGTGGGAGGCGACACAGTCGT	-27.2	-	Novel-mir-1025-3p	-	TTTAGGATGATGTTGTGGGAGGCG
Novel-mir-1021	GCTGTTGTGACCACATATGACCTAGTTTCACAGACGAGGCGAGGGGGAAGGGATACTGCCCGAGTTTGTTTTGATTCTCACTTTGTCCTTAACAG	-21.6	-	Novel-mir-1021-3p	-	TTGTTTTGATTCTCACTTTG
Novel-mir-1007	TCTGTGACTGGCTGCTGGACTTTCTGACTGGCAGGCCCCAGACTGTCAGGATTGGTAATAGGACTTCAGCCAGCATTGTTACAAA	-29.5	-	Novel-mir-1007-3p	-	TTGGTAATAGGACTTCAGCCAGCA
Novel-mir-994	TGACAACTAATGATGTTCCAGTGTTTTCAGTCTCAGTAGAGGCACGCAATGGCATTTGATGTTGAACATTATTTTGAAAGTCTGTTG	-20.4	-	Novel-mir-994-3p	-	TTGATGTTGAACATTATTTTGA
Novel-mir-991	CTACAGGTTCCATACTGAGACATGGCACGATCATTTAGGCTGTATACTTAATTGTGTATTCTACTGTTTGACTTGGTCTGCTCCTGCATGCGTACGTGTGT	-25.2	-	Novel-mir-991-3p	-	TTGACTTGGTCTGCTCCTGCATGC
Novel-mir-982	CCCAGCTCACTGTGCCTGCCCTGACCTGTCAGTGGATCACCAGTTTTCTGATAGACAGGAAGCAGCAGGTGAGGCTGGA	-36.3	-	Novel-mir-982-3p	-	TTCTGATAGACAGGAAGCAGCAGG
Novel-mir-971	TGTCCTTCCGTGCAGGCATTGTTACCTGAGCTGTGAAGGCTTTTAGACTTTCCATAGCTTGTGTCAGTGCTTCTACTTGGGCA	-30.6	-	Novel-mir-971-3p	-	TTCCATAGCTTGTGTCAGTGCTTC
Novel-mir-932	AGACCAGCTTTCCTGAGTTGCCTGGTGAAGTATAGCTGTTGCTGGGCTTTCTTTATTGTGGCAGCTGTGTTCAGTGTCCAGCTCAGGTCAGGTGTCACCTG	-40.06	-	Novel-mir-932-3p	-	TGTTCAGTGTCCAGCTCAGGTCAG
Novel-mir-902	GCTGCTATCTTGTTTAGCATTAGCTCGCAGTGCCACACACGCCTCCCATTCAGCATTGTGGTTTTGTTTGAGGTGCTGGAATAAATTCGTCG	-21.2	-	Novel-mir-902-3p	-	TGGTTTTGTTTGAGGTGCTGGAAT
Novel-mir-895	CGGGCATGGGACCGCATCTTCATGGCTCCGGCCATGCAGGGCTGTGTGAGTATGAGCAGATAAAGGCTGGGTTAGAGCAGAAGAAGAGGGAGAGAGGATGA	-26.7	-	Novel-mir-895-3p	-	TGGGTTAGAGCAGAAGAAGAGGGA
Novel-mir-884	TGTTTGGTGACAGAGTTTGTTTGTTTCAGTCTTTGTCTGTAAGCTGGATACAGGAACAAAGAAACGTGGTCTGAGC	-18.6	-	Novel-mir-884-3p	-	TGGATACAGGAACAAAGAAACG
Novel-mir-884	TGTTTGGTGACAGGGTTTGTTTGTTTCAGTCTTTGCCTGTAAGCTGGATACAGGAACAAAGAAACGTGGTCTGAGC	-19.9	-	Novel-mir-884-3p	-	TGGATACAGGAACAAAGAAACG
Novel-mir-313	AAGCCACCCAAGATGATGCGCAACATGTGGGTTTGTTAGTGTAGTAGGTGTGTGCGTAGTTGCTGTTGGTATGATCATTGTTGCTG	-18.9	-	Novel-mir-313-5p	-	TGCGTAGTTGCTGTTGGTATGATC
Novel-mir-860	ACTGGGGAACACCCCAGTGCACAAAGTCCATGATCGGCTGGTACTGTTTGTTTATGATGGGCGTGCATGCAGGACTAGAGATCGGCTGGCTCCGACTTCC	-33.6	-	Novel-mir-860-3p	-	TGCAGGACTAGAGATCGGCTGGC
Novel-mir-846	AGACCGGGTGTCTATCAGCGGCAGCAGAAACACAGTCAATGGTATGATGGGTGATGATGGGCCGGGCTGCGGACGTTGGGATCA	-26.4	-	Novel-mir-846-3p	-	TGATGATGGGCCGGGCTGCGGAC
Novel-mir-838	TGGGCTCAGGCAGGTCCCCAAGGGCAGGGACTCAGGAGCAAGGGAGTACGCCTCTGCCCTGAGTGTAGTGTCCCCGGGAGAATCGTCCG	-37.4	-	Novel-mir-838-3p	-	TGAGTGTAGTGTCCCCGGGA
Novel-mir-831	GATGACCAGCACCCTTCCTAGAGCTGGCCGTCCGGCCAAACTGAGCTATCGGGGGAGAGGAGCCTTGGTGAGA	-28.3	-	Novel-mir-831-3p	-	TGAGCTATCGGGGGAGAGGAGC

Novel-mir-826	GTCTCTGCTCCTGTACTTGGTCAAAGCTCCTCAGTGTTGGATAGATGCCTGAGAGGTTCTGAACAAGTATAGGATCTCTGA	-30.7	-	Novel-mir-826-3p	-	TGAGAGGTTCTGAACAAGTATA
Novel-mir-824-1	ATGGTCACGGGGGCACCACACTCGATATCTTTCTCGCTTCACTTTACAGAGGGAAATGAGACGAAACAGGCACTGTGGTTACGTGAAGGC	-21	-	Novel-mir-824-3p	-	TGAGACGAAACAGGCACTGTGGTT
Novel-mir-824-2	GGTCGCGGGGGCACCGCACTCGACATCTTTCTCGCTTCACTTTACAGAGGGAAATGAGACGAAACAGGCACTGTGGTTACGTGCGGCG	-26.3	-	Novel-mir-824-3p	-	TGAGACGAAACAGGCACTGTGGTT
Novel-mir-807	AAGCGGAGGCGATGATGGCTGTGAACACAGGGACATGGAGAACAGAGACAGGTGAAGTGGAGACTGTGCATCGACCTGTACGGCTG	-21.5	-	Novel-mir-807-3p	-	TGAAGTGGAGACTGTGCATCGACC
Novel-mir-761	AGCTCAGCAGACATCTGCCCTCCCATTCTAACTACCTGTGGTCTTTCAGTCAGGAAGTCCAAAATCTAGTTACAGGTGGGAGTCGGGACACTGAGGT	-32.5	-	Novel-mir-761-3p	-	TCTAGTTACAGGTGGGAGTCGGG
Novel-mir-734	TCTGTCAGAAAGCTAGTAATCCACTGACAGGTGGGGGCAGGCACAGTGAGCTGGGTCAGTTTGGAGTGGAGGATTTCCGGGATGATGG	-23.9	-	Novel-mir-734-3p	-	TCAGTTTGGAGTGGAGGATTTCC
Novel-mir-715	GCCCTGCCAAAACTGTTTACAGTGGAGGTGAGCAGCTGTTGACCTCAACTGGGGACATTGTCAGACGGTGGAAAGAAT	-27.3	-	Novel-mir-715-3p	-	TCAACTGGGGACATTGTCAGACGG
Novel-mir-707-1	TTTATTGATTTTCAAAAGGCCTTTGACACTATTAATTTTGAAATTTTACTTGATAAATTGGAAAGGTATGGTATTAGAGGTTTGGTGCTGAAATGGATAA	-23.5	-	Novel-mir-707-3p	-	TATGGTATTAGAGGTTTGGTGCTG
Novel-mir-707-2	TTTATTGATTTTCAAAAGGCCTTTGGCACTATTCATTTTGAAATTTTACTTGATAAATTGGAAAGGTATGGTATTAGAGGTTTGGTGCTGAAATGGATAA	-22.7	-	Novel-mir-707-3p	-	TATGGTATTAGAGGTTTGGTGCTG
Novel-mir-707-3	TTTATTGATTTTCAAAAGGCTTTTGATACTATTTTACTTGATAAATTGGAAAGGTATGGTATTAGAGGTTTGGTGCTGAAATGGATAA	-22.4	-	Novel-mir-707-3p	-	TATGGTATTAGAGGTTTGGTGCTG
Novel-mir-696	TGACAGGCTTGTTGGAGGTGTTTAATACTCTAACCGGGATCCATCTGTCACCGTTTATAGTTGTTACAGACCTGCAGACAATGACGTCG	-23.2	-	Novel-mir-696-3p	-	TAGTTGTTACAGACCTGCAGAC
Novel-mir-665	TGTGTTACAGCTCCAGACTTGACAATGTAGAGGTGAGCCATCTTACATGTGTCAGACATGAGGCTGAAATTAG	-22.7	-	Novel-mir-665-3p	-	TACATGTGTCAGACATGAGG
Novel-mir-637	AAGAGGGCAGCTGACGGACAGGATGTTCTTGTGAACCAGTTCGATGGGTCCTGGTCTGTGAGAGATCTTGTCGTTGAGGTCGTCGG	-33.3	-	Novel-mir-637-3p	-	GGTCTGTGAGAGATCTTGTCGTTG
Novel-mir-601	GCCGAGGCGTGCTGAGGCAAAGTGGATAACGGCCCATTCTCAATTGCAGAGTCATTTGTCCCGGGTCATTTGTCCCAGTAGTTGCATTCCCACT	-23.7	-	Novel-mir-601-3p	-	CCGGGTCATTTGTCCCAGTAGTTG
Novel-mir-599	AATGCCGCCTTGCTGTCTGGCCAATGGCCTGCTTTGGGACTGACCCCAAGACTTTTGTTGCCTCCCTAGACTATGCCAGCCACAAAATCGTATG	-20.7	-	Novel-mir-599-3p	-	CCCTAGACTATGCCAGCCACA
Novel-mir-593	TGTACTGATCAGTGGGTATTTACTATGTTCATATTGCACTCTATGGTCATTTTAAGTGCTGAGGACCATGAGTAAAGTATCCACGTACCTCTCGTATG	-24.3	-	Novel-mir-593-3p	-	CCATGAGTAAAGTATCCACGTAC
Novel-mir-561	TCTTGTACTGCCATGGCATAGACCTCCTCTCTGCTGTGGAATCATGGTATTGGGAGAGAAACTCTGGACTTTGGCACAGG	-24.9	-	Novel-mir-561-3p	-	ATTGGGAGAGAAACTCTGGACT
Novel-mir-537	TGTATAGAGACACCTGTAAGCCAACAGTGTGAGTGTGGGTATCGGGGACTGTGGTTACTGTGGATGCAGCAGGCTGTGAATTCGGTAT	-19.6	-	Novel-mir-537-3p	-	ACTGTGGATGCAGCAGGCTGTG
Novel-mir-532-1	GAGTCTTTCTTCACATGTTCATGTGCTGCTACTGTTTACTTCCTGAACTCAGACGTTCAGACATGAAACAGTAACTGTGGCCTGTGCAACAGTTCAGGCTC	-30.7	-	Novel-mir-532-3p	-	ACAGTAACTGTGGCCTGTGCAACA
Novel-mir-532-2	GAGTCTTTCTTCACATGTTCATGTGCTGCTACTGTTTATTTCTTGAACTCAGATGTTCAGACATGAAACAGTAACTGTGGCCTGTGCAACAGTTCAGGCTC	-29.1	-	Novel-mir-532-3p	-	ACAGTAACTGTGGCCTGTGCAACA
Novel-mir-532-3	GAGTCTTTTTTCACATGCTCATGTGCTGCTACTGTTTATTTCCTGAGCTCAGATGTTCAGACATGAAACAGTAACTGTGGCCTGTGCAACAGTTCAGGCTC	-30.89	-	Novel-mir-532-3p	-	ACAGTAACTGTGGCCTGTGCAACA
Novel-mir-539	AACACTTTGTGTGTCTCCTGCAGCTACCGTTACTGCAAGAACAAGCCCTACCCCAAGTCCCGCTTCTGCAGGGGTGTGCCTGGTAAG	-21.7	-	Novel-mir-529-3p	-	AAGTCCCGCTTCTGCAGGGGTGT

Supplementary Table S4 | The primers used in the study

Primers for qRT-PCR and making constructs, Sites for restriction enzymes are underlined.

Primer name	Primer sequence (5′-3′)	PCR conditions
eel-U6	F: CAAATTCGTGAAGCGTTCCTCAT	94 °C, 15 s; 60 °C, 20s; 72 °C, 20s; 40 cycles
dre-U6	F: CAAATTCGTGAAGCGTTCCATCT	94 °C, 15 s; 60 °C, 20s; 72 °C, 20s; 40 cycles
dre/eel-miR-17a-5p	F: CAAAGTGCTTACAGTGCAGGTA	94 °C, 15 s; 60 °C, 20s; 72 °C, 20s; 40 cycles
dre/eel-miR-18a-5p	F: TAAGGTGCATCTAGTGCAGATA	94 °C, 15 s; 60 °C, 20s; 72 °C, 20s; 40 cycles
dre/eel-miR-19a-3p	F: TGTGCAAATCTATGCAAAACTGA	94 °C, 15 s; 60 °C, 20s; 72 °C, 20s; 40 cycles
dre/eel-miR-19b-3p	F: TGTGCAAATCCATGCAAAACTGA	94 °C, 15 s; 60 °C, 20s; 72 °C, 20s; 40 cycles
dre/eel-miR-20a-5p	F: TAAAGTGCTTATAGTGCAGGTAG	94 °C, 15 s; 60 °C, 20s; 72 °C, 20s; 40 cycles
dre/eel-miR-92a-3p	F: TATTGCACTTGTCCCGGCCTGTA	94 °C, 15 s; 60 °C, 20s; 72 °C, 20s; 40 cycles
dre-miR-17a-3p	F：ACTGCAGTGGAGGCACTTCTAG	94 °C, 15 s; 60 °C, 20s; 72 °C, 20s; 40 cycles

dre-miR-19a-5p	F:CTAGTTTTGCATAGTTGCACTAA	94 °C, 15 s; 60 °C, 20s; 72 °C, 20s; 40 cycles
dre-miR-19b-5p	F:AGTTTTGCTGGTTTGCATTCAGA	94 °C, 15 s; 60 °C, 20s; 72 °C, 20s; 40 cycles
dre-miR-20a-3p	F:ACTGCAGTGTGAGCACTTGAAG	94 °C, 15 s; 60 °C, 20s; 72 °C, 20s; 40 cycles
eel-miR-17a-3p	F:ACTGCAGTGAAGGCACTTTCAA	94 °C, 15 s; 60 °C, 20s; 72 °C, 20s; 40 cycles
eel-miR-18a-3p	F:ACTGCCCTAAGTGCTCCTTCTA	94 °C, 15 s; 60 °C, 20s; 72 °C, 20s; 40 cycles
eel-miR-19a-5p	F:CTAGTTTTGCATAGCTGCACTA	94 °C, 15 s; 60 °C, 20s; 72 °C, 20s; 40 cycles
eel-miR-19b-5p	F:AGTTTTGCTGGTTTGCATTCCGC	94 °C, 15 s; 60 °C, 20s; 72 °C, 20s; 40 cycles
eel-miR-20a-1-3p	F:ACTGCATTGTGAGCACTTAAAA	94 °C, 15 s; 60 °C, 20s; 72 °C, 20s; 40 cycles
eel-miR-20a-3-3p	F: CTGCAATGTGAGCACTTGAAGT	94 °C, 15 s; 60 °C, 20s; 72 °C, 20s; 40 cycles
Psi-dmrt1-3'UTR	F: CCGCTCGAGGGGGTCGTTCTATAAGGTTTT<br>R: AATGCGGCCGCGCTTTGGTCTTGTTTTGTAAT	94 °C, 30 s; 62 °C, 30s; 72 °C, 1 min; 35 cycles
Psi-dmrt1-3'UTR Mut-1	F: TCATTCTTTCTGTACTGGAACTCAGTAT<br>R: TCGCAGTAGTACTTACTGTCAAATCTTA	94 °C, 30 s; 56 °C, 30s; 72 °C, 6 min; 30 cycles

Psi-dmrt1-3'UTR Mut-2	F: TCATGTTTAGTAAGACTAACAGTTAATT<br>R: TCGCCTGTTGAATATGTGCATTTTCTAA	94 °C, 30 s; 56 °C, 30s; 72 °C, 6 min; 30 cycles
pcDNA6.2-EmGFP-miR-19a	F: CACTGACTGACTCAGTTTTATAGATTTGCA CACAGGACACAAGGCCTGTTACTAGCAC<br>R: GCCAAAACTCAGTTTTGCATAGATTTGCA CACAGCATACAGCCTTCAGCAAGCCTCCA	94 °C, 30 s; 55 °C, 30s; 68 °C, 6 min; 30 cycles
pcDNA6.2-EmGFP-miR-19b	F: CACTGACTGACTCAGTTTTATGGATTTGCA CACAGAGCACAAGGCCTGTTACTAGCAC<br>R: GCCAAAACTCAGTTTTGCATGGATTTGCA CACAGCATACAGCCTTCAGCAAGCCTCCA	94 °C, 30 s; 55 °C, 30s; 68 °C, 6 min; 30 cycles
pcDNA6.2-EmGFP-miR-17a	F: CACTGACTGAC TACCTGCAGTAAGCACTT TGCAGGACACAAGGCCTGTTACTAGCAC<br>R: GCCAAAACTACCTGCACTGTAAGCACTTT GCAGCATACAGCCTTCAGCAAGCCTCCA	94 °C, 30 s; 55 °C, 30s; 68 °C, 6 min; 30 cycles
pcDNA6.2-EmGFP-miR-20a	F: CACTGACTGACCTACCTGCTATAAGCACTT TACAGGACACAAGGCCTGTTACTAGCAC<br>R:GCCAAAACCTACCTGCACTATAAGCACTT TACAGCATACAGCCTTCAGCAAGCCTCCA	94 °C, 30 s; 55 °C, 30s; 68 °C, 6 min; 30 cycles

Supplementary Figure S1

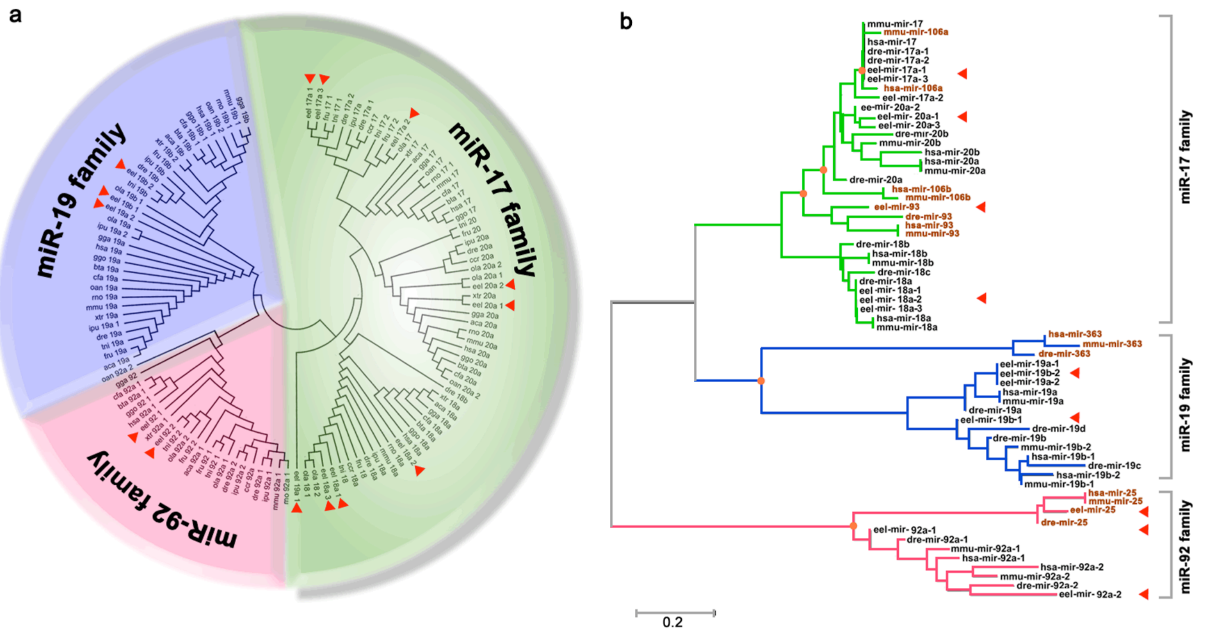

Supplementary Figure S1 | Phylogenetic analysis of miR-17-92 cluster and its paralogues. (a) Phylogenetic tree shows three miRNA families including miR-17, miR-19 and miR-92. The tree was constructed using miRNA precursors from fourteen vertebrate species including the swamp eel with MAGA 6.0. Red triangles indicate miRNAs in swamp eel. (b) Phylogenetic analysis highlights the relationship of miR-17-92 with its paralogous clusters. Red dots show branch points. miR-17-92 clusters are labeled in black color, while paralogous clusters are labeled in brown color. Red triangles indicate miRNAs in swamp eel.

Supplementary Figure S2

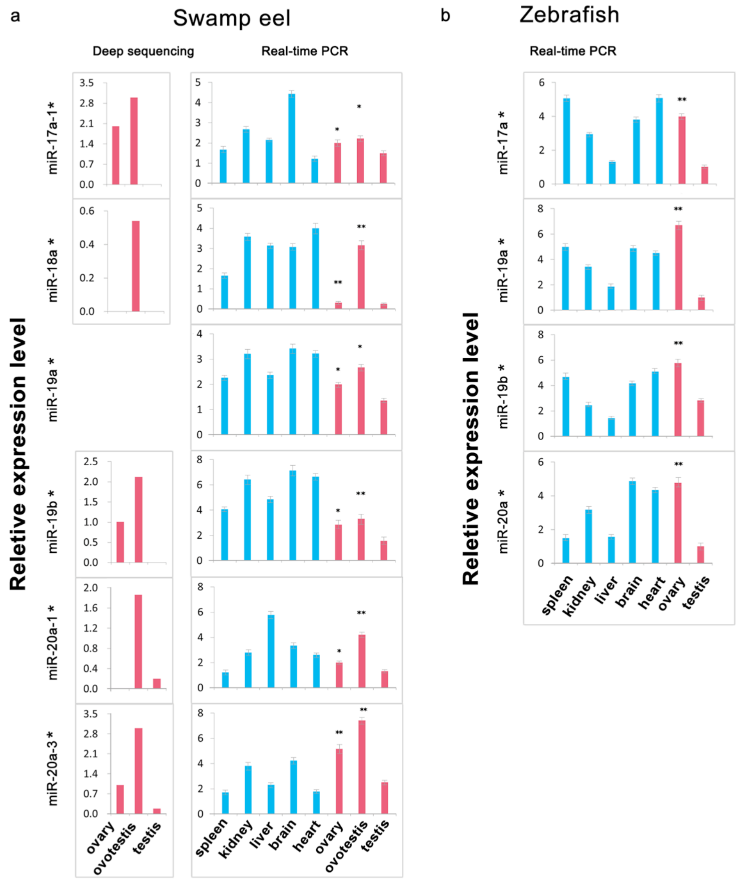

Supplementary Figure S2 | Tissue distributions of star miRNAs of miR-17-92 cluster in both zebrafish and swamp eel. (a) Six star miRNAs were expressed in adult tissues of swamp eel. The expression level in gonads was detected by q-PCR in comparison with deep sequencing (red bars). miR-19a* was not detected by deep sequencing. However, by searching the reference genome, we did find the mature and typical stem loop of miR-19a, and detected its expression by q-PCR. (b) miR-17-92 cluster zebrafish generated six miRNAs guide strands and four star miRNAs. All of the star miRNAs were expressed in adult tissues of zebrafish. The expression was analyzed by q-PCR. The statistical significance was assessed by Student's t-test with p value < 0.05(*), p value < 0.01(**). The y axis indicates the log2 fold change.

Supplementary Figure S3

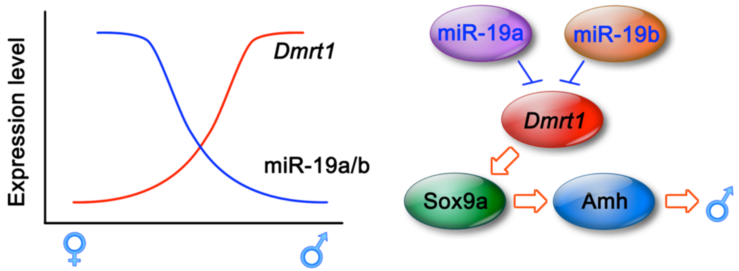

Supplementary Figure S3 | The schematic diagram of the *Dmrt1* expression regulation by miR19a/b. A negative correlation between *Dmrt1* and miR19a/b expressions supports the regulation pathway in gonads. Through direct repression on *Dmrt1*, miR-19a/b inhibits the expression levels of *Sox9a* and its downstream gene, *Amh*, thus leading to testis differentiation.
